# Switching the site-selectivity of C–H activation in aryl sulfonamides containing strongly coordinating N-heterocycles†
†Electronic supplementary information (ESI) available. CCDC 1874184 and 1874186. For ESI and crystallographic data in CIF or other electronic format see DOI: 10.1039/c9sc03691a


**DOI:** 10.1039/c9sc03691a

**Published:** 2019-08-12

**Authors:** Yi Dong, XuePeng Zhang, Jiajing Chen, Wenxing Zou, Songwen Lin, Heng Xu

**Affiliations:** a State Key Laboratory of Bioactive Substance and Function of Natural Medicines , Institute of Materia Medica , Chinese Academy of Medical Sciences , Peking Union Medical College , Beijing 100050 , China . Email: xuheng@imm.ac.cn; b Beijing Key Laboratory of Active Substances Discovery and Druggability Evaluation , Institute of Materia Medica , Chinese Academy of Medical Sciences , Peking Union Medical College , Beijing 100050 , China; c Lab of Computational and Drug Design , Peking University Shenzhen Graduate School , Shenzhen 518055 , China

## Abstract

The limitations of arene C–H functionalization of aryl sulfonamides containing strongly coordinating N-heterocycles were overcome using a Rh(iii) catalyst. The site-selectivity of C–H carbenoid functionalization at the *ortho* position relative to either the sulfonamide or N-heterocycle directing groups was elegantly switched using solvents of different polarities and different additive concentrations. Importantly, sulfonamide-group-directed *ortho*-C–H carbenoid functionalization tolerated strongly coordinating N-heterocycles, including pyridine, pyrrole, thiazole, pyrimidine, and pyrazine. Density functional theory (DFT) calculations were performed to rationalize the reaction mechanisms and the influence of reaction polarity.

## Abstract 2

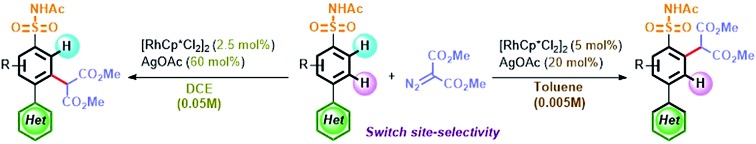
Switching the site-selectivity of C–H activation in aryl sulfonamides containing strongly coordinating N-heterocycles was achieved using a Rh^III^-catalyst.

## Introduction

**Fig. 1:**
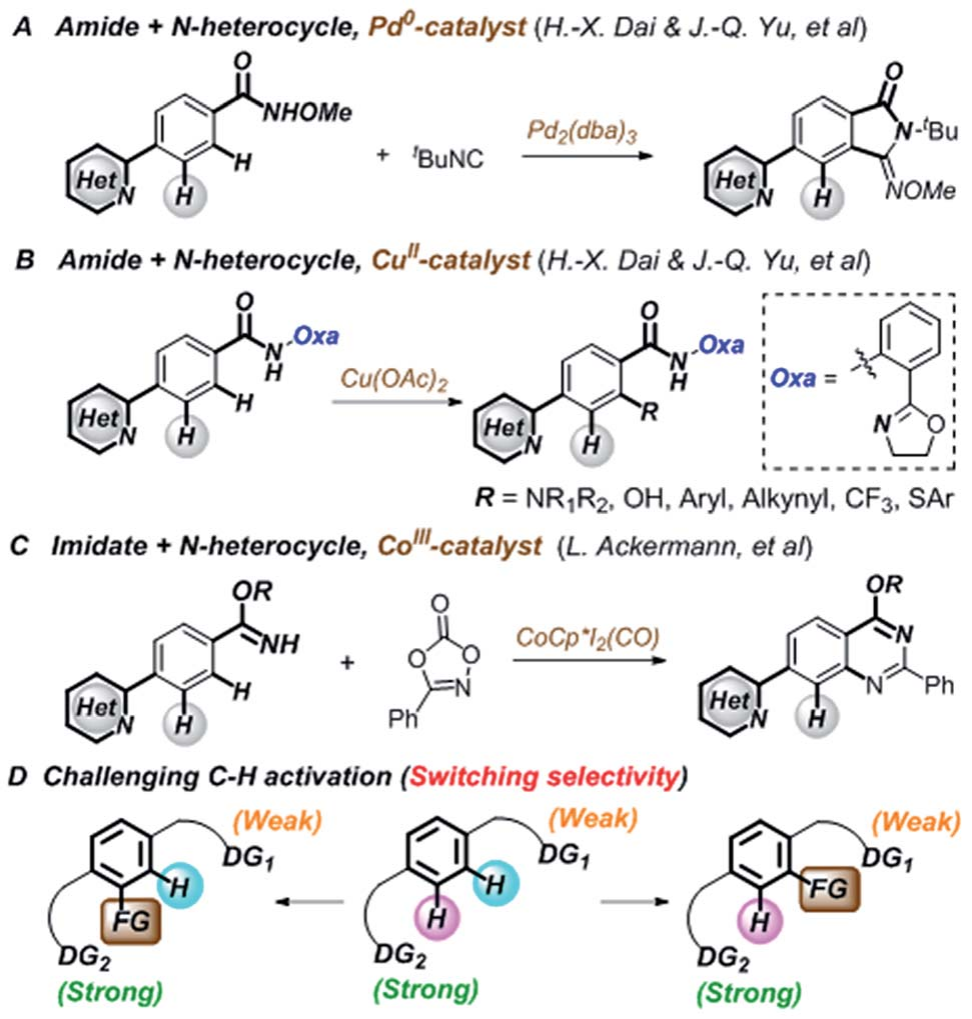
Overcoming the limitation of directed C–H functionalization.

Transition-metal-catalyzed C–H functionalization is an important and efficient synthetic tool in organic synthesis owing to its straightforward and economical features.^1^–^6^ Although directing-group-oriented aryl C–H functionalization has developed rapidly in the past decade, some limitations have remained difficult to overcome.^7^–^9^ For example, C–H activation often selectively occurs at the *ortho* position relative to the directing group.^10^–^12^ However, when two or more directing groups coexist in one molecule, C–H activation directed by weakly coordinating groups is difficult to achieve owing to competing strongly coordinating directing groups, such as N-heterocycles.^13^–^21^ These groups often preferentially bind with transition metals and interfere with C–H functionalization through the desired directing group, which hinders late-stage modification in drug discovery.^22^–^24^ Recently, Dai, Yu, and Ackermann *et al.* reported pioneering examples of palladium-,^25^ copper-,^26^ and cobalt-catalyzed^27^ site-selective C–H functionalizations of amides and imidates that tolerated strongly coordinating N-heterocycles (Fig. 1A–C). However, switching the C–H functionalization selectivity of substrates containing two directing groups remains challenging (Fig. 1D).^28^–^38^

**Fig. 2:**
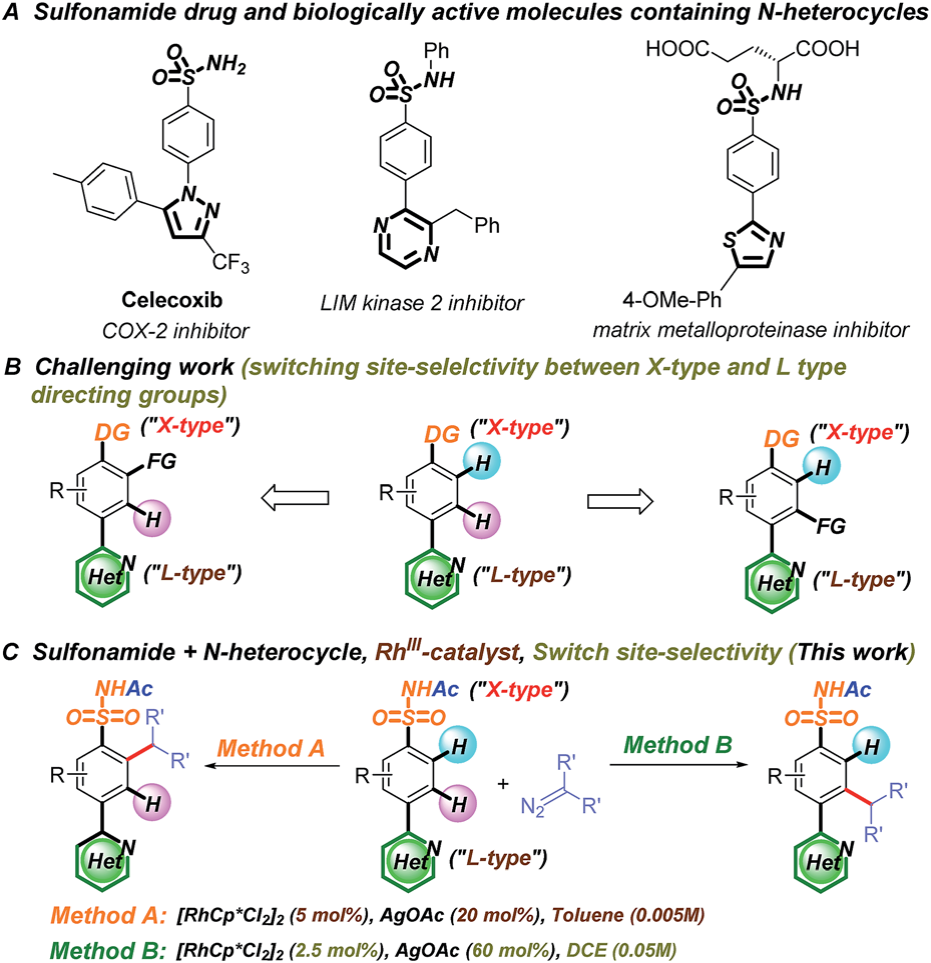
Site-selective C–H functionalization of sulfonamides containing N-heterocycles.

Both N-heterocycles and sulfonamides are ubiquitous in biologically active molecules owing to their hydrogen bond forming abilities and favorable drug-like properties.^39^–^43^ Considering their dual pharmacological effects, switching the selectivity of the C–H functionalization of aryl sulfonamides containing strongly coordinating N-heterocycles would be invaluable in the late-stage modification of potential drug molecules (Fig. 2A).^44^–^46^ In this study, we successfully switched the selectivity of the C–H carbenoid functionalization^47^–^66^ of sulfonamides^67^–^80^ containing N-heterocycles^81^ using a rhodium catalyst. The selectivity was precisely controlled by changing the reaction medium polarity and additive concentration, affording C–H activation at the *ortho* position relative to the N-heterocycle (charge-neutral forms, denoted as L-type ligands) or sulfonamide (easily deprotonated to charge-negative forms, denoted as X-type ligands) directing groups.

## Results and discussion

Initially, this Rh(iii)-catalyzed site-selective C–H carbenoid functionalization was investigated for solvent effects (0.05 M concentration) by using AgOAc as an additive at 60 °C, with the results summarized in Table 1 (entries 1 to 5). When 3-methyl-4-thiazole-*N*-acetyl sulfonamide (**1a**) was used as a model substrate, C–H carbenoid functionalization product **3′** at the *ortho*-position of thiazole was only observed and isolated in 79% yield when MeOH (a polar protonic solvent) was used as the reaction solvent (Table 1, entry 1). Other tested polar solvents, such as DMF and MeCN, also favored generation of the C–H activated product at the *ortho*-position of the thiazole group (Table 1, entries 2 and 3). When non-polar solvents were used, such as DCE and toluene, the thiazole-directed C–H activated product was decreased significantly (Table 1, entries 4 and 5). It was notable that the amount of sulfonamide directed C–H activated product **3** was close to thiazole directed C–H activated product **3′** (**3**/**3′** ratio, 1 : 1.6) when less-polar toluene was used as the reaction solvent (Table 1, entry 5). These results suggested that the polarity of the solvent strongly determined the site-selectivity of this rhodium catalyzed C–H carbenoid functionalization: the less polar the solvent, the more favorable was the sulfonamide-directed C–H activation product. Importantly, this phenomenon was more obvious at low concentrations (Table 1, entries 6–8), and when the reaction concentration was reduced to 0.01 M in DCE or toluene, an increasing amount of sulfonamide-directed activation product **3** was observed. It was worth noting that product **3** was isolated as the major product and the **3**/**3′** ratio could reach 33 : 1 when the reaction was carried out in toluene at 0.01 M concentration (Table 1, entry 7). The result given by a solvent mixture of DCE and toluene (v/v = 1 : 1) more clearly reflected the solvent polarity effect on this Rh(iii)-catalyzed site-selective C–H carbenoid functionalization (Table 1, entry 8). Further decreasing the concentration to 0.005 M resulted in an excellent site-selectivity of **3** (**3**/**3′** ratio, >99 : 1, Table 1, entry 9). Reducing the amount of [RhCp*Cl_2_]_2_ to 2.5 mol% resulted in a decreased **3** : **3′** ratio of 28 : 1, which showed that 5 mol% of the catalyst loading amount was necessary for this selective sulfonamide directed C–H carbenoid functionalization (entry 10). In contrast, increasing the reaction concentration led to increase of the thiazole-directed product **3′**, which also well reflected the significant solvent effect of this site-selective C–H carbenoid functionalization (Table 1, entries 11 and 12). On the other hand, the effect of the amount of AgOAc on site-selectivity was also considered and systematically investigated in DCE (Table 1, entries 13 to 16). Increasing the amount of AgOAc resulted in a lower **3**/**3′** ratio (entries 13 and 14), which reached <1 : 99 when the amount of AgOAc was 8- and 12-fold that of [RhCp*Cl_2_]_2_ respectively. It is worth noting that 2.5 mol% [RhCp*Cl_2_]_2_ and 60 mol% AgOAc loading amount could guarantee an excellent site-selectivity of **3′** (**3**/**3′** ratio, <1 : 99, entry 15). These results showed that increasing the amount of AgOAc favored C–H functionalization at the thiazole *ortho*-position. It may be possible that changing the AgOAc concentration affected the polarity of the reaction environment, influencing the site-selectivity. It is also possible that AgOAc plays the role of a Lewis acid which could bind to an N-heterocycle or diazo compound and affect the site-selectivity.^82^ When a more economic acetate salt tetrabutylammonium acetate was used as an additive instead of AgOAc, similar yields were obtained. However, the site-selectivities of the C–H carbenoid functionalization were significantly reduced (entries 17 and 18). Therefore, two optimal conditions to switch site-selectivity at the *ortho*-position of sulfonamide and N-heterocycle were chosen respectively (entries 9 and 15). These two methods not only gave good yields, but more importantly excellent site-selectivities.

**Table 1:** Optimization of the site-selective C–H carbenoid functionalization^*a*^

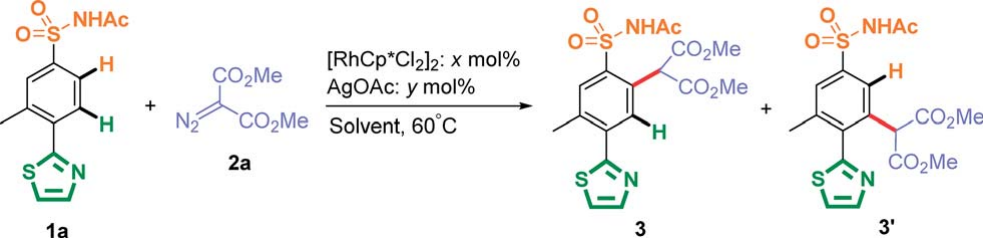					
Entry	*x*/*y*	Solvent	Conc.	Yield/%^*b*^	**3**/**3′**^*c*^
1	5/20	MeOH	0.05 M	**3′**/79	Only **3′**
2	5/20	DMF	0.05 M	**3′**/77	1 : 48
3	5/20	MeCN	0.05 M	**3′**/78	1 : 11
4	5/20	DCE	0.05 M	**3′**/65	1 : 4
5	5/20	Toluene	0.05 M	**3** + **3′**/26 + 50	1 : 1.6
6	5/20	DCE	0.01 M	**3** + **3′**/40 + 44	1 : 1
7	5/20	Toluene	0.01 M	**3**/80	33 : 1
8	5/20	Toluene/DCE = 1 : 1	0.01 M	**3**/62	4 : 1
**9**	**5/20**	**Toluene**	**0.005 M**	****3**** **/81**	**>99 : 1**
10	2.5/10	Toluene	0.005 M	**3**/80	28 : 1
11	5/20	DMF	0.25 M	**3′**/81	<1 : 99
12	5/20	MeCN	0.25 M	**3′**/80	1 : 47
13	5/40	DCE	0.05 M	**3′**/88	<1 : 99
14	5/60	DCE	0.05 M	**3′**/87	<1 : 99
**15**	**2.5/60**	**DCE**	**0.05 M**	****3′**** **/88**	**<1 : 99**
16	2.5/20	DCE	0.05 M	**3′**/83	1 : 30
17^*d*^	5/20	Toluene	0.005 M	**3**/79	26 : 1
18^*d*^	2.5/60	DCE	0.05 M	**3** + **3′**/29 + 46	1 : 1.5

^*a*^Reaction conditions: [RhCp*Cl_2_]_2_ (*x* mol%), AgOAc (*y* mol%), **1a** (0.125 mmol), **2a** (1.1 equiv.), solvent, 60 °C.

^*b*^Isolated yield.

^*c*^Determined by ^1^H NMR analysis of the crude reaction mixture before separation.

^*d*^Tetrabutylammonium acetate instead of AgOAc.

**Scheme 1:**
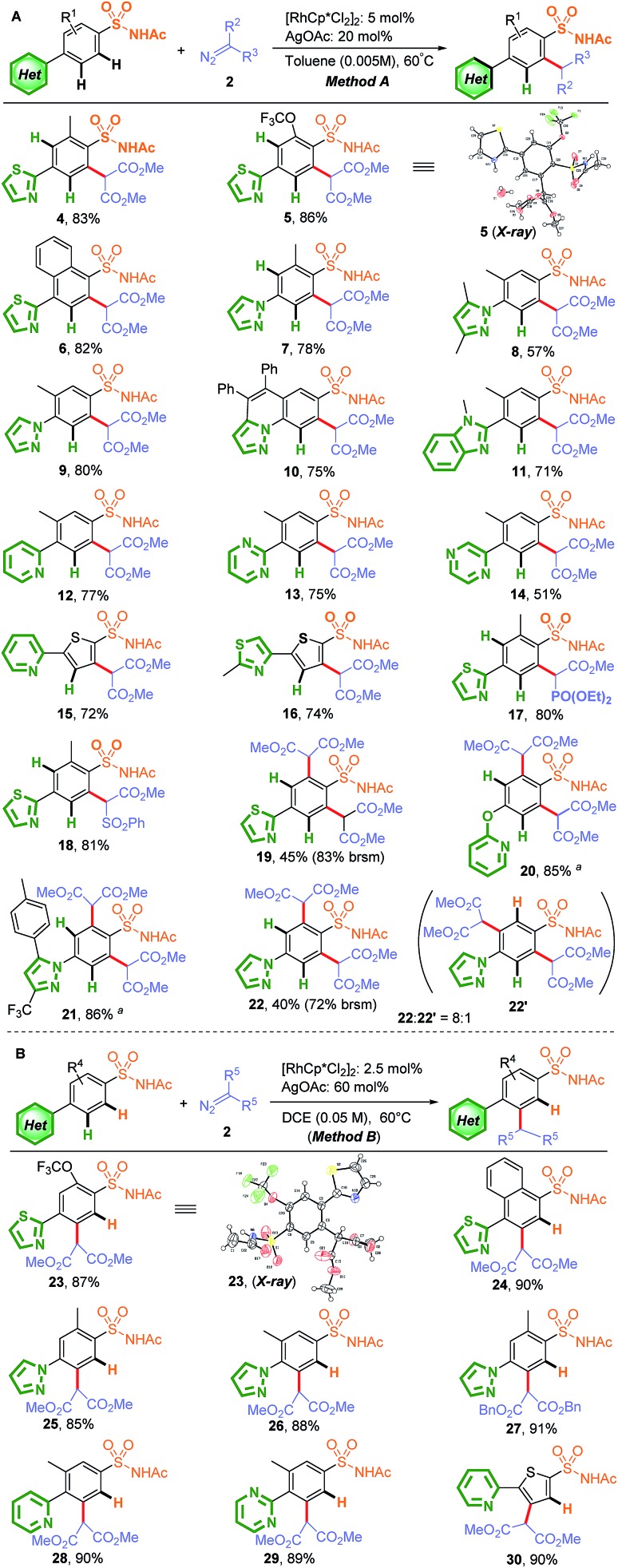
Site-selective *ortho* C–H carbenoid functionalization of sulfonamides containing N-heterocycles. (A) Method A: [RhCp*Cl_2_]_2_ (5 mol%), AgOAc (20 mol%), sulfonamides containing N-heterocycles (0.125 mmol), **2a** (1.1 equiv.), toluene (25 mL), 60 °C. brsm = based on the recovered starting material. ^*a*^2.1 equiv. of **2a** was used. (B) Method B: [RhCp*Cl_2_]_2_ (2.5 mol%), AgOAc (60 mol%), sulfonamides containing N-heterocycles (0.125 mmol), **2a** (1.1 equiv.), DCE (2.5 mL), 60 °C.

With the two optimal conditions in hand, sulfonamide substrates containing various N-heterocycles in a competitive position were further investigated, including thiazole, pyrazole, imidazole, pyridine, pyrimidine, and pyrazine substrates (Scheme 1). As expected, sulfonamide-group-directed *ortho*-C–H carbenoid functionalization proceeded in the presence of 5.0 mol% [RhCp*Cl_2_]_2_ catalyst and 20 mol% AgOAc as the additive in less-polar toluene (0.005 M) at 60 °C, affording excellent site-selectivity (Scheme 1A, Method A, **4–14**). In addition to phenyl sulfonamides, thienyl sulfonamides also showed excellent site-selectivity (**15** and **16**), while substrates containing phosphate ester and sulfone ester diazo groups instead of dimethyl 2-diazomalonate also gave satisfactory results (**17** and **18**). Notably, C–H di-activation at the *ortho*-positions relative to the sulfonamide group afforded the major product when *para*-heterocycle-substituted benzenesulfonamide was used (**19–22**). As shown in Scheme 1B, when the reaction was conducted in DCE (0.05 M), the N-heterocycle-directed C–H carbenoid functionalization of this difunctional compound performed well in the presence of 2.5 mol% [RhCp*Cl_2_]_2_ catalyst and 60 mol% AgOAc as the additive at 60 °C, with good yields and excellent site-selectivity (Method B, **23–30**). A 1,2-disubstituted substrate was also tried, but no desired carbenoid functionalization product was obtained, which may be the result of a bidentate coordination, impeding the free coordination site for cyclometallation.

**Scheme 2:**
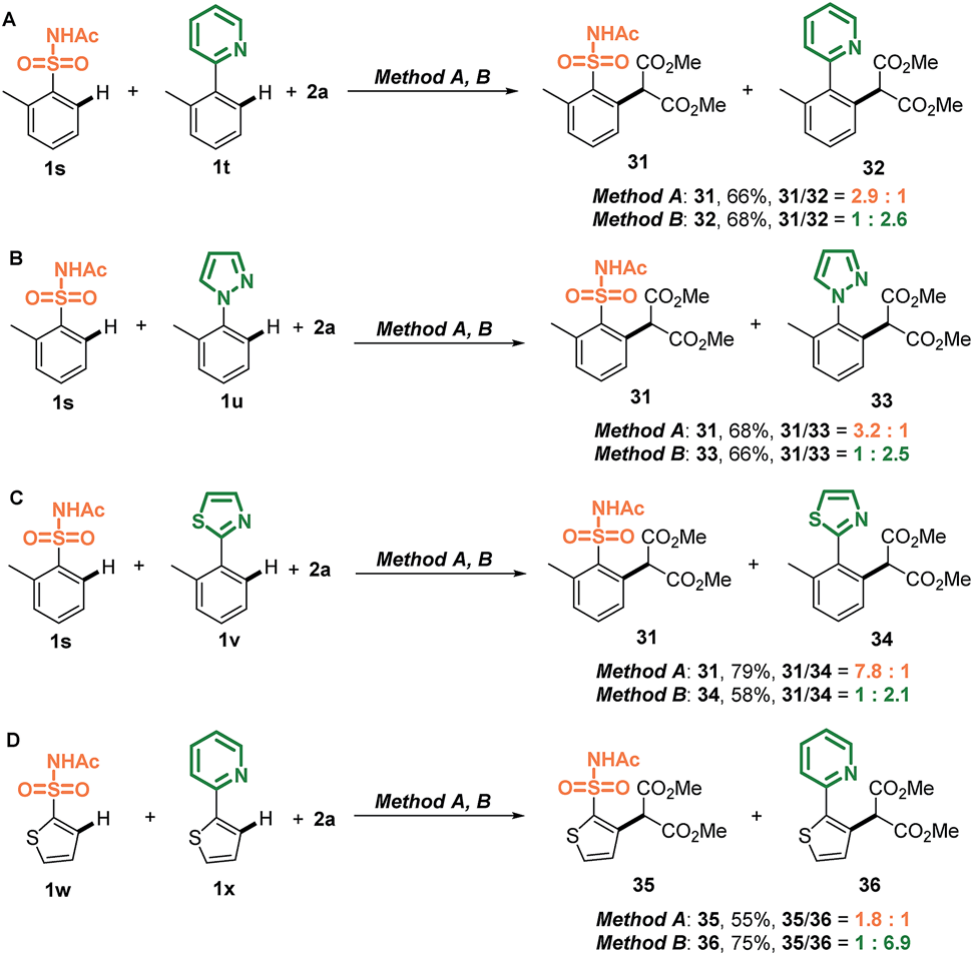
Intermolecular competition experiments between X-type sulfonamides and L-type N-heterocycles.

Intermolecular competition experiments were conducted to explore the unique chemoselectivity of difunctional sulfonamides containing N-heterocycle substrates (Scheme 2). Notably, X-type sulfonamides outcompeted strongly coordinating L-type thiazole, pyrazole, and pyridine substrates using method A. In contrast, when the competition reaction was conducted using method B, N-heterocycle-oriented C–H carbenoid functionalization was the major pathway. These intermolecular competition results implied that changing the C–H carbenoid functionalization reaction conditions could affect the competitive reactivity between X-type and L-type directing groups. More importantly, this strategy could prevent interference from L-type N-heterocycles to afford X-type sulfonamide-oriented C–H activation, despite N-heterocycles possessing strongly coordinating properties with metal catalysts.

**Scheme 3:**
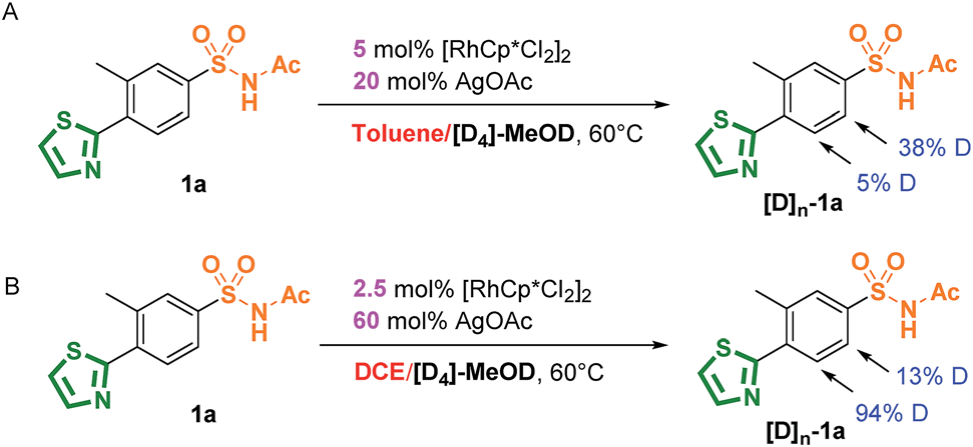
Isotope-labelling experiments.

**Scheme 4:**
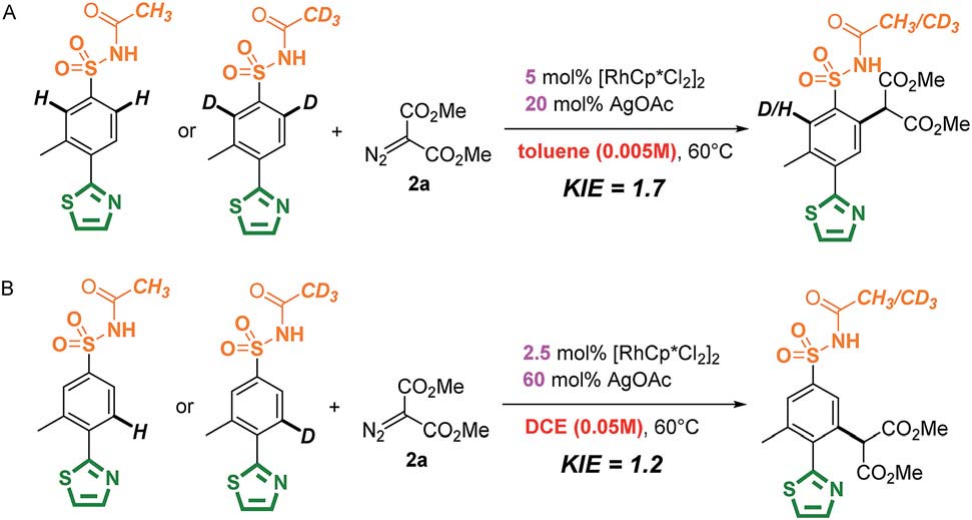
Kinetic isotope effect experiments.

The mechanism of the site-selective aryl C–H carbenoid functionalization between sulfonamide and N-heterocycles was further investigated by experimental studies and theoretical calculations. The mechanism of directing group oriented aryl C–H carbenoid functionalization has been illustrated in the previous literature, and three major steps including C–H activation, metal–carbene formation and final C–C bond formation process were proposed to take place.^50^ Firstly, as shown in Scheme 3, isotope-labelling experiments of 3-methyl-4-thiazole-*N*-acetyl sulfonamide (**1a**) by method A and B in the presence of [D_4_]-MeOD showed that reversible processes for sulfonamide and thiazole directed C–H activation were involved under method A and B conditions.^83^–^85^ Notably, there was a relatively slight H/D exchange (5%) at the *ortho* position of the thiazole group using [D_4_]-MeOD as a co-solvent in the method A catalytic system, implying weak reversible properties. As shown in Scheme 4, kinetic isotope effect (KIE) experimental results showed a relatively small value, indicating that C–H bond cleavage was likely not the rate-determining step in the catalytic cycle.^86^,^87^ These results suggested that the selectivity does not appear in the first C–H activation step, but in the subsequent steps possibly.

In order to delve into our conjecture about the mechanism on selectivity, density functional theory (DFT) calculations were performed. The RhCp*(OAc)_2_ complex was commonly considered as the active catalyst under the conditions of [RhCp*Cl_2_]_2_ with an abundant AgOAc additive.^51^,^54^,^57^ As implied in our experiments, the results of site-selective C–H carbenoid functionalization catalyzed by RhCp*(OAc)_2_ were similar to those catalyzed by RhCp*Cl_2_/AgOAc, which proved that RhCp*(OAc)_2_ was an active catalyst for the C–H carbenoid functionalization (details in the ESI†). An easier N–H deprotonation of the acetyl-substituted sulfonamide was predicted, and the active catalyst RhCp*(OAc)_2_ would firstly dissociate one OAc^–^ ligand to coordinate with the deprotonated sulfonamide substrate to form the reactant complex **RC**. Similar to previous reports, domino C–H activation, metal–carbene formation, and finally C–C bond formation processes were predicted to take place.^88^–^90^ The deprotonated sulfonamide moiety or *para*-thiazole group would act as a directing group to locate the Rh(iii) center adjacent to the *ortho*-C–H bond of either the sulfonamide or thiazole group, which guaranteed facilitation of the *ortho*-C–H activation processes in the transition state **TS1** or **TS1′**. Both aryl C–H deprotonation to the mono-coordinated OAc^–^ ligand and Rh–C bond formation processes were observed in the concerted-metalation–deprotonation (CMD)^91^–^93^ type C–H activation transition state **TS1** or **TS1′**. Subsequently, the Rh(iii)-bound neutral HOAc molecule would be easily replaced by the diazo compound **2a**, and metal–carbene formation through clear nitrogen extrusion processes was observed in both **TS2** and **TS2′**. The active metal–carbene would attack an adjacent aryl carbon center to generate C–C bond formation transition state **TS3** or **TS3′**. Finally, transient product **PC** or **PC′** was obtained through site-selective carbenoid functionalization at the *ortho*-position of either the sulfonamide or thiazole moiety. Alternatively, the active catalyst RhCp*(OAc)_2_ might directly react with diazo compound **2a** to form a metal–carbene complex *via* a similar nitrogen extrusion process in **TS_carbene_**.

**Fig. 3:**
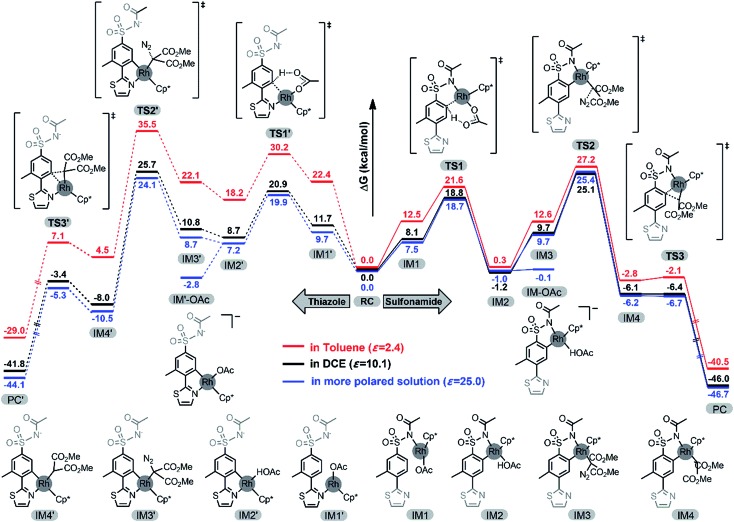
Profile of the potential energy surface (PES) of site-selective carbenoid functionalization in aryl sulfonamide **1a** catalyzed by the RhCp*(OAc)_2_ complex. The energy of reactant **RC** is referred to as the total energy of dissociative reactants (**1a**, **2a**, RhCp*(OAc)_2_).

**Scheme 5:**
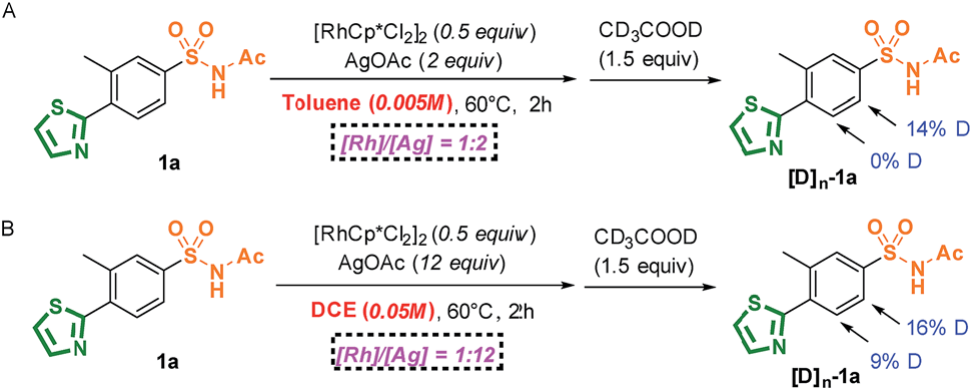
Stoichiometric C–H rhodation experiment.

The profiles of the potential energy surface proposed above are shown in Fig. 3. It can be noted that the energies of the direct metal–carbene formation transition state **TS_carbene_** (31.2 and 34.2 kcal mol^–1^ in toluene and DCE, respectively) were higher than the barriers depicted in Fig. 3, which reconfirmed the fact that the active Rh(iii) catalysts would preferentially react with aryl substrates (details in the ESI†). As depicted in Fig. 3, the free energy barrier in the metal–carbene formation was higher than those of the C–H activation process and C–C formation step. The calculated results demonstrated that the C–H activation step was not rate-determining, which was consistent with experimental KIE results. In addition, stoichiometric C–H rhodation experiments were conducted (Scheme 5). As shown in Scheme 5A, 14% and 0% D, respectively, were incorporated at the *ortho*-positions of the sulfonamide and thiazole groups at a lower reaction concentration (0.005 M) in toluene, which was supported by the energy barrier of the *ortho*-sulfonamide C–H activation in toluene (**TS1** is 8.6 kcal mol^–1^ lower than **TS1′**). Furthermore, the competitive C–H activation results shown in Scheme 5B confirmed the calculated narrow free energy gap between **TS1** and **TS1′** (2.1 kcal mol^–1^) in DCE. The concentration factor was considered in Scheme 3, which implied that the increased concentration of reaction complexes and more polarized solvent would favour thiazole-directed C–H activation. The experimental observations were in consistence with calculated C–H activation barriers, especially in the pathway of the more polarized solution (*ε* = 25.0) wherein a narrower free energy gap between **TS1** and **TS1′** was observed (1.2 kcal mol^–1^). More importantly, a stable thiazole-directed intermediate **IM′-OAc** was generated, which was responsible for more deuteration at the thiazole side in Scheme 3B.

**Fig. 4:**
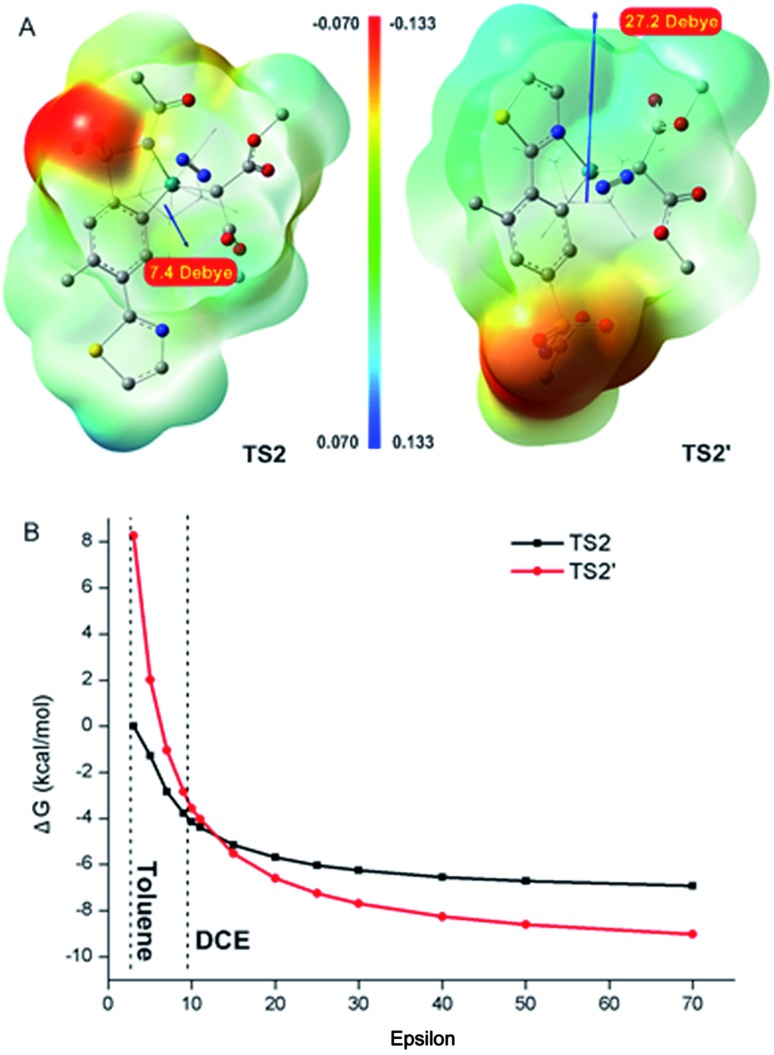
(A) Electrostatic potential (ESP) and dipole moment of **TS2** and **TS2′** in DCE. (B) Relative energy changes of **TS2** and **TS2′** with increasing solvent dielectric constant. The energy of **TS2** in toluene (*ε* = 2.4) was set to zero.

The reaction selectivities were further rationalized. As depicted in Fig. 3, the sulfonamide-directed pathway was more energetically favourable than the thiazole-directed pathway in toluene (red pathway). The calculated results were in good agreement with the experimental observations that *ortho*-sulfonamide C–H carbenoid functionalization product **3** was the major product in toluene (Table 1, entries 7, 9 and 10). Furthermore, close relative free energy barriers of C–H activation transition states **TS1** (18.8 kcal mol^–1^) and **TS1′** (20.9 kcal mol^–1^), and of metal–carbene formation transition states **TS2** (25.1 kcal mol^–1^) and **TS2′** (25.7 kcal mol^–1^) in DCE were observed (black pathway). The calculated competitive reaction pathways in DCE were in good agreement with experimental findings that both *ortho*-sulfonamide product **3** and *ortho*-thiazole product **3′** were obtained in DCE at a lower AgOAc concentration (Table 1, entry 6). The use of a more polarized solvent and/or high concentration of reaction complexes was also investigated, which together contributed to the increasing polarity of the reaction environment. As described above, the metal–carbene formation process was the rate-determining step, and therefore, the energy alterations of **TS2** and **TS2′** under various polarization conditions were investigated. As depicted in Fig. 4A, the thiazole-directed **TS2′** was more polarized than the sulfonamide-directed **TS2**, which would rationalize the free energy changes in Fig. 4B wherein the thiazole-directed **TS2′** was better stabilized in high polarity surroundings. Therefore, PES profiles in a more polarized solution are also provided in Fig. 3 (blue pathway). As demonstrated in Fig. 3, the energies of the thiazole-directed reaction species were decreased and the free energy of the rate-determining **TS2′** was lower than that of **TS2**. Meanwhile, as mentioned above, the introduction of excess OAc^–^ ligand would result in a more stable thiazole-directed intermediate **IM2′-OAc**. Thereby, the decreased thiazole-directed energy barriers and the important OAc^–^ ligand participation demonstrated in Fig. 3 (blue), as well as the better stabilization of **TS2′** in more polarized surroundings shown in Fig. 4B, would reasonably explain the reaction selectivities under the conditions of more polarized solvent and/or high concentration of reaction complexes demonstrated in Table 1.

## Conclusions

In summary, we have developed a Rh(iii)-catalyzed arene C–H carbenoid functionalization with good yields and excellent site-selectivity switching for aryl sulfonamides containing strongly coordinating N-heterocycles. The polarity of the reaction environment was an important factor influencing the site-selectivity. Notably, the combination of a less-polar solvent, such as toluene, and a lower additive concentration favored C–H functionalization at the *ortho*-position relative to the sulfonamide group with excellent site-selectivity. Furthermore, strongly coordinating N-heterocycles, including pyridine, pyrrole, thiazole, pyrimidine, and pyrazine, were tolerated. This switchable site-selectivity carbenoid functionalization methodology is suitable for the late-stage modification of N-heterocycle-derived sulfonamide drugs.

## Conflicts of interest

There are no conflicts to declare.

## Supplementary Material

Supplementary information

Crystal structure data

## References

[cit1] Ackermann L. (2011). Chem. Rev..

[cit2] Kuhl N., Schoöder S., Glorius F. (2014). Adv. Synth. Catal..

[cit3] Cernak T., Dykstra K. D., Tyagarajan S., Vachal P., Krska S. W. (2016). Chem. Soc. Rev..

[cit4] He J., Wasa M., Chan K. S. L., Shao Q., Yu J.-Q. (2017). Chem. Rev..

[cit5] Park Y., Kim Y., Chang S. (2017). Chem. Rev..

[cit6] Newton C. G., Wang S.-G., Oliveira C. C., Cramer N. (2017). Chem. Rev..

[cit7] Lyons T. W., Sanford M. S. (2010). Chem. Rev..

[cit8] Colby D. A., Bergman R. G., Ellman J. A. (2010). Chem. Rev..

[cit9] Arockiam P. B., Bruneau C., Dixneuf P. H. (2012). Chem. Rev..

[cit10] Hummel J. R., Boerth J. A., Ellman J. A. (2017). Chem. Rev..

[cit11] Ackermann L. (2007). Top. Organomet. Chem..

[cit12] Omae I. (2004). Coord. Chem. Rev..

[cit13] Wasa M., Worrell B. T., Yu J.-Q. (2010). Angew. Chem., Int. Ed..

[cit14] Malik H. A., Taylor B. L. H., Kerrigan J. R., Grob J. E., Houk K. N., Du Bois J., Hamann L. G., Patterson A. W. (2014). Chem. Sci..

[cit15] Shen Q., Hartwig J. F. (2007). J. Am. Chem. Soc..

[cit16] Kuhl N., Hopkinson M. N., Wencel-Delord J., Glorius F. (2012). Angew. Chem., Int. Ed..

[cit17] Duez S., Steib A. K., Manolikakes S. M., Knochel P. (2011). Angew. Chem., Int. Ed..

[cit18] Campeau L.-C., Rousseaux S., Fagnou K. (2005). J. Am. Chem. Soc..

[cit19] Leclerc J.-P., Fagnou K. (2006). Angew. Chem., Int. Ed..

[cit20] Kanyiva K. S., Nakao Y., Hiyama R. (2007). Angew. Chem., Int. Ed..

[cit21] Segawa Y., Maekawa T., Itami K. (2015). Angew. Chem., Int. Ed..

[cit22] Bryan M. C., Dillon B., Hamann L. G., Hughes G. J., Kopach M. E., Peterson E. A., Pourashral M., Raheem I., Richardson P., Richter D., Sneddon H. F. (2013). J. Med. Chem..

[cit23] Schönherr H., Cernak T. (2013). Angew. Chem., Int. Ed..

[cit24] Meanwell N. A. (2011). Chem. Res. Toxicol..

[cit25] Liu Y.-J., Xu H., Kong W.-J., Shang M., Dai H.-X., Yu J.-Q. (2014). Nature.

[cit26] Shang M., Wang M.-M., Saint-Denis T. G., Li M.-H., Dai H.-X., Yu J.-Q. (2017). Angew. Chem., Int. Ed..

[cit27] Wang H., Lorion M. M., Ackermann L. (2016). Angew. Chem., Int. Ed..

[cit28] Campeau L. C., Schipper D. J., Fagnou K. (2008). J. Am. Chem. Soc..

[cit29] Lapointe D., Markiewicz T., Whipp C. J., Toderian A., Fagnou K. (2011). J. Org. Chem..

[cit30] Alderson J. M., Phelps A. M., Scamp R. J., Dolan N. S., Schomaker J. M. (2014). J. Am. Chem. Soc..

[cit31] Gu Y., Shen Y., Zarate C., Martin R. (2019). J. Am. Chem. Soc..

[cit32] Dolewski R. D., Fricke P. J., McNally A. (2018). J. Am. Chem. Soc..

[cit33] Kim H. J., Kim J., Cho S. H., Chang S. (2011). J. Am. Chem. Soc..

[cit34] Zhang J., Sha S.-C., Bellomo A., Trongsiriwat N., Gao F., Tomson N. C., Walsh P. J. (2016). J. Am. Chem. Soc..

[cit35] Tiwari V. K., Kamal N., Kapur M. (2017). Org. Lett..

[cit36] Lee S., Mah S., Hong S. (2015). Org. Lett..

[cit37] Boyaala R., Touzani R., Roisnel T., Dorcet V., Caytan E., Jacquemin D., Boixel J., Guerchais V., Doucet H., Soulé J.-F. (2019). ACS Catal..

[cit38] Kerr W. J., Reid M., Tuttle T. (2015). ACS Catal..

[cit39] Barraza S. J., Denmark S. E. (2018). J. Am. Chem. Soc..

[cit40] Vitaku E., Smith D. T., Niardarson J. T. (2014). J. Med. Chem..

[cit41] Ammazzalorso A., De Filippis B., Giampietro L., Amoroso R. (2017). Chem. Biol. Drug Des..

[cit42] Carta F., Supuran C. T., Scozzafava A. (2014). Future Med. Chem..

[cit43] Chinthakindi P. K., Naicker T., Thota N., Govender T., Kruger H. G., Arvidsson P. I. (2017). Angew. Chem., Int. Ed..

[cit44] Pal M., Madan M., Padakanti S., Pattabiraman V. R., Kalleda S., Vanguri A., Mullangi R., Rao Mamidi N. V. S., Casturi S. R., Malde A., Gopalakrishnan B., Yeleswarapu K. R. (2003). J. Med. Chem..

[cit45] Delbecq F., Cordonnier G., Pommery N., Barbry D., Hénichart J.-P. (2004). Bioorg. Med. Chem. Lett..

[cit46] Goodwin N. C., Cianchetta G., Burgoon H. A., Healy J., Mabon R., Strobel E. D., Allen J., Wang S., Hamman B. D., Rawlins D. B. (2015). ACS Med. Chem. Lett..

[cit47] Davies H. M. L., Morton D. (2011). Chem. Soc. Rev..

[cit48] Xia Y., Zhang Y., Wang J. (2013). ACS Catal..

[cit49] Hu F., Xia Y., Ma C., Zhang Y., Wang J. (2015). Chem. Commun..

[cit50] Xia Y., Qiu D., Wang J. (2017). Chem. Rev..

[cit51] Chan W.-W., Lo S.-F., Zhou Z., Yu W.-Y. (2012). J. Am. Chem. Soc..

[cit52] Shi Z., Koester D. C., Boultadakis-Arapinis M., Glorius F. (2013). J. Am. Chem. Soc..

[cit53] Hyster T. K., Ruhl K. E., Rovis T. (2013). J. Am. Chem. Soc..

[cit54] Hu F., Xia Y., Ye F., Liu Z., Ma C., Zhang Y., Wang J. (2014). Angew. Chem., Int. Ed..

[cit55] Ye B., Cramer N. (2014). Angew. Chem., Int. Ed..

[cit56] Liang Y., Yu K., Li B., Xu S., Song H., Wang B. (2014). Chem. Commun..

[cit57] Zhou B., Chen Z., Yang Y., Ai W., Tang H., Wu Y., Zhu W., Li Y. (2015). Angew. Chem., Int. Ed..

[cit58] Xia Y., Liu Z., Feng S., Zhang Y., Wang J. (2015). J. Org. Chem..

[cit59] Gutiérrez-Bonet Á., Juliá-Hernández F., de Luis B., Martin R. (2016). J. Am. Chem. Soc..

[cit60] Kim J. H., Greβies S., Glorius F. (2016). Angew. Chem., Int. Ed..

[cit61] Dateer R. B., Chang S. (2016). Org. Lett..

[cit62] Zhou Q., Li S., Zhang Y., Wang J. (2017). Angew. Chem., Int. Ed..

[cit63] Long Z., Wang Z., Zhou D., Wan D., You J. (2017). Org. Lett..

[cit64] Sun Y., Cramer N. (2018). Angew. Chem., Int. Ed..

[cit65] Shen B., Wan B., Li X. (2018). Angew. Chem., Int. Ed..

[cit66] Dong Y., Chen J., Xu H. (2019). Chem. Commun..

[cit67] Dai H.-X., Stepan A. F., Plummer M. S., Zhang Y.-H., Yu J.-Q. (2011). J. Am. Chem. Soc..

[cit68] Pham M. V., Ye B., Cramer N. (2012). Angew. Chem., Int. Ed..

[cit69] Xie W., Yang J., Wang B., Li B. (2014). J. Org. Chem..

[cit70] Ding Q., Liu T., Zheng Q., Zhang Y., Long L., Peng Y. (2014). RSC Adv..

[cit71] Li X., Dong Y., Qu F., Liu G. (2015). J. Org. Chem..

[cit72] Kalsi D., Sundararaju B. (2015). Org. Lett..

[cit73] Planas O., Whiteoak C. J., Company A., Ribas X. (2015). Adv. Synth. Catal..

[cit74] Liu W., Wang D., Zhao Y., Yi F., Chen J. (2016). Adv. Synth. Catal..

[cit75] Vanjari R., Guntreddi T., Singh K. N. (2016). Chem.–Asian J..

[cit76] Lan T., Wang L., Rao Y. (2017). Org. Lett..

[cit77] Thrimurtulu N., Nallagonda R., Volla C. M. R. (2017). Chem. Commun..

[cit78] Nguyen T. T., Grigorjeva L., Daugulis O. (2017). Chem. Commun..

[cit79] Barsu N., Kalsi D., Sundararaju B. (2018). Catal. Sci. Technol..

[cit80] Ran Y., Yang Y., You H., You J. (2018). ACS Catal..

[cit81] Yang L., Huang H. (2015). Chem. Rev..

[cit82] Zhao P., Wang F., Han K., Li X. (2012). Org. Lett..

[cit83] Li Y., Qi Z., Wang H., Yang X., Li X. (2016). Angew. Chem., Int. Ed..

[cit84] Wang J., Wang M., Chen K., Zha S., Song C., Zhu J. (2016). Org. Lett..

[cit85] Yang Y., Wang X., Li Y., Zhou B. (2015). Angew. Chem., Int. Ed..

[cit86] Jiang H., Gao S., Xu J., Wu X., Lin A., Yao H. (2016). Adv. Synth. Catal..

[cit87] Li Y., Wang F., Yu S., Li X. (2016). Adv. Synth. Catal..

[cit88] Zhou T., Guo W., Xia Y. (2015). Chem.–Eur. J..

[cit89] Xu H., Zhang X., Ke Z., Zhao C. (2016). RSC Adv..

[cit90] Wang C., Zhou Y., Bao X. (2017). J. Org. Chem..

[cit91] Gorelsky S. I., Lapointe D., Fagnou K. (2008). J. Am. Chem. Soc..

[cit92] Hyster T. K., Knörr L., Ward T. R., Rovis T. (2012). Science.

[cit93] Gorelsky S. I. (2013). Coord. Chem. Rev..

