# Supplementary material for: Switching the site-selectivity of C–H activation in aryl sulfonamides containing strongly coordinating N-heterocycles
Source: Chem Sci. 2019 Aug 12;10(38):8744–51. doi: 10.1039/c9sc03691a (PMC6857669; doi:10.1039/c9sc03691a)

Electronic Supplementary Material (ESI) for Chemical Science.
This journal is © The Royal Society of Chemistry 2019

Switching the site-selectivity of C–H activation in aryl sulfonamides containing strongly coordinating *N*-heterocycles

Yi Dong,[†,‡,#] Xuepeng Zhang,[§,#] Jiajing Chen,[†,‡] Wenxing Zou,[†,‡] Songwen Lin,[†,‡] Heng Xu*,[†,‡]

[†]State Key Laboratory of Bioactive Substance and Function of Natural Medicines, Institute of Materia Medica, Chinese Academy of Medical Sciences and Peking Union Medical College, Beijing 100050, China.

[‡]Beijing Key Laboratory of Active Substances Discovery and Druggability Evaluation, Institute of Materia Medica, Chinese Academy of Medical Sciences and Peking Union Medical College, Beijing 100050, China.

[§]Lab of Computational and Drug Design, Peking University Shenzhen Graduate School, Shenzhen 518055, China.

Table of cotent

General methods……S1

Optimization of the site-selective C–H carbenoid functionalization………………………………………......S1

Copies of ^{1}HNMR analysis for Table 1……………………………………………………………………..……..…...S4

Synthesis of [RhCp*$(OAc)_2$] and its catalytic process of C–H carbenoid functionalization………S13

Computational Details………S14

Reference…….S16

Copies of ^{1}HNMR analysis for the ratio of **22** and **22'** by method A (Scheme 1) ………………………..….S17

Intermolecular competition experiments………………………………………………………………………………S18

Isotope-labelling experiments…………………………………………………………………………………………….S26

Stoichiometric C–H rhodation experiments……………………………………………………………….…….…S28

kinetic isotope effect (KIE) experiments………………………………………………………….………………S30

Synthetic procedure, NMR and HRMS data of products……………………………………………….………...S32

HSQC and HMBC of compound **3** and **3'** ………………………………………………… ……………………….S50

Copies of NMR spectra of products………………………………………………… …. …. ………………………….S57

X-ray analysis of compound **5** (CCDC1874186)………………………. …………………………………….…...S92

X-ray analysis of compound **23** (CCDC1874184)…………………………………. ………………………….S100

General methods

DCE and toluene were purchased from domestic corporations and used without purification, $[RhCp^*Cl_2]_2$ was purchased from TCI. Analytical thin layer chromatography (TLC) plates, preparative TLC and the silica gel for column chromatography were phased from Qingdao Haiyang Chemical and Special Silica Gel Co, Ltd.

High-resolution LC-MS was carried out by Agilent LC/MSD TOF using a column of Agilent ZORBAX SB-C18 (rapid resolution, 3.5 μm, 2.1 × 30 mm) at a flow of 0.40 mL/min. The solvent was MeOH/water (75:25 (v/v)), containing 5 mmol/L ammonium formate. The ion source is electrospray ionization (ESI).

Proton nuclear magnetic resonance (^1H NMR) and carbon nuclear magnetic resonance (^{13}C NMR) spectroscopy were performed on Bruker Advance 400M NMR and 600M NMR spectrometers. Chemical shifts of ^1H NMR spectra are reported as in units of parts per million (ppm) downfield from SiMe4 (δ 0.0) and relative to the signal of chloroform-*d* (δ = 7.260, singlet) and DMSO-*d6* (δ = 2.500, quintet). Multiplicities were given as: s (singlet); d (doublet); t (triplet); q (quartet); dd (doublet of doublets); m (multiplets), etc. The number of protons (n) for a given resonance is indicated by nH. Carbon nuclear magnetic resonance spectra (^{13}C NMR) are reported as in units of parts per million (ppm) downfield from SiMe4 (δ 0.0) and relative to the signal of chloroform-*d* (δ = 77.230, triplet) and DMSO-*d6* (δ = 39.510, septet).

Optimization of the site-selective C–H carbenoid functionalization (Table 1)

General procedure for this site-selective C-H carbenoid functionalization: A tube equipped with a magnetic stir bar was charged with $[RhCp^*Cl_2]_2$ (*x* mol%), AgOAc (*y* mol%), sulfonamide containing *N*-heterocycle (0.125 mmol) and solvent, then diazo compound **2a** (1.1 equivlent) was added. The tube was sealed, and the reaction mixture was stirred at 60°C over night. Solvent was removed under *vacuo*, and 20 mL DCM was added. The mixture was then filtered, the filtrate was concentrated, the ratio of **3** and **3’** was determined by ^1H NMR analysis of the crude reaction mixture before separation and the residue was purified by preparative TLC on silica gel to afford desired compound **3** and **3’**.

Table S1. Solvent effect between different solvents

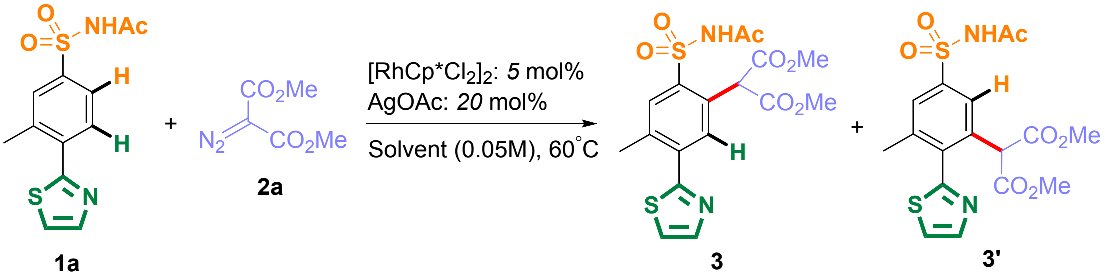

Entry		Solvent	Yield/%	3/3'
1	polarity	MeOH	**3'**/79	Only **3'**
2		DMF	**3'**/77	1:48
3		MeCN	**3'**/78	1:11
4		DCE	**3'**/65	1:4
5		toluene	**3+3'**/26+50	1:1.6

Table S2. Solvent effect by using toluene, DCE and their mixture

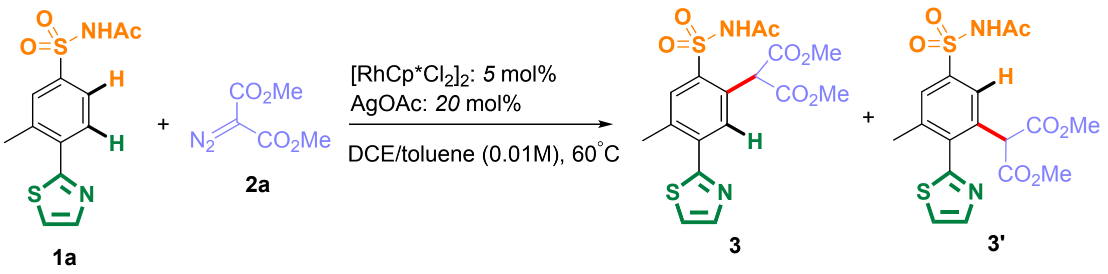

Entry	Solvent		Ratio of DCE/ toluene	Yield/%	3/3'
1		polarity	100%/0	**3+3'**/40+44	1:1
2	DCE/toluene		50%/50%	**3**/62	4:1
3			0/100%	**3**/80	33:1

Table S3. Reaction concentration effect

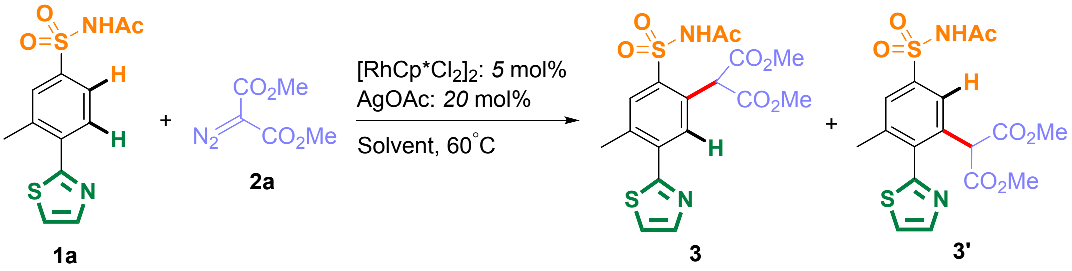

Entry	Solvent	Concentration	Yield/%	3/3'
1	DMF	0.05M	3'/77	1:48
2		0.25M	3'/81	<1:99
3	MeCN	0.05M	3'/78	1:11
4		0.25M	3'/80	1:47
5	DCE	0.05M	3'/65	1:4
6		0.01M	3+3'/40+44	1:1
7	toluene	0.05M	3+3'/26+50	1:1.6
8		0.01M	3/80	33:1
9		0.005M	3/81	>99:1

Table S4. AgOAc effect

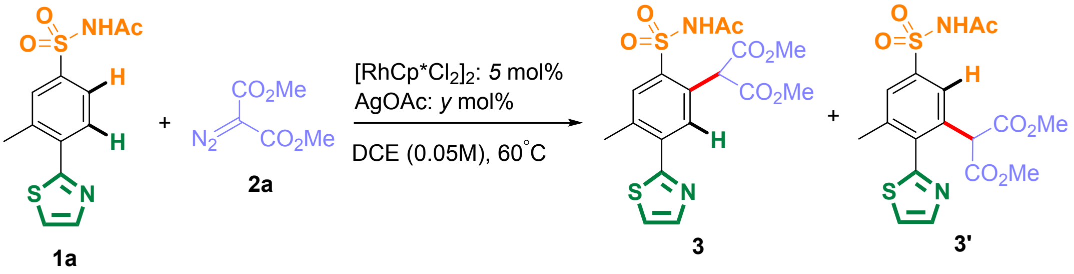

entry	*y*	Yield/%	3/3'
1	20	3'/65	1:4
2	40	3'/88	<1:99
3	60	3'/87	<1:99

__Table 1, entry 1 (Rh:5%, AgOAc:20%, MeOH, 0.05M)__

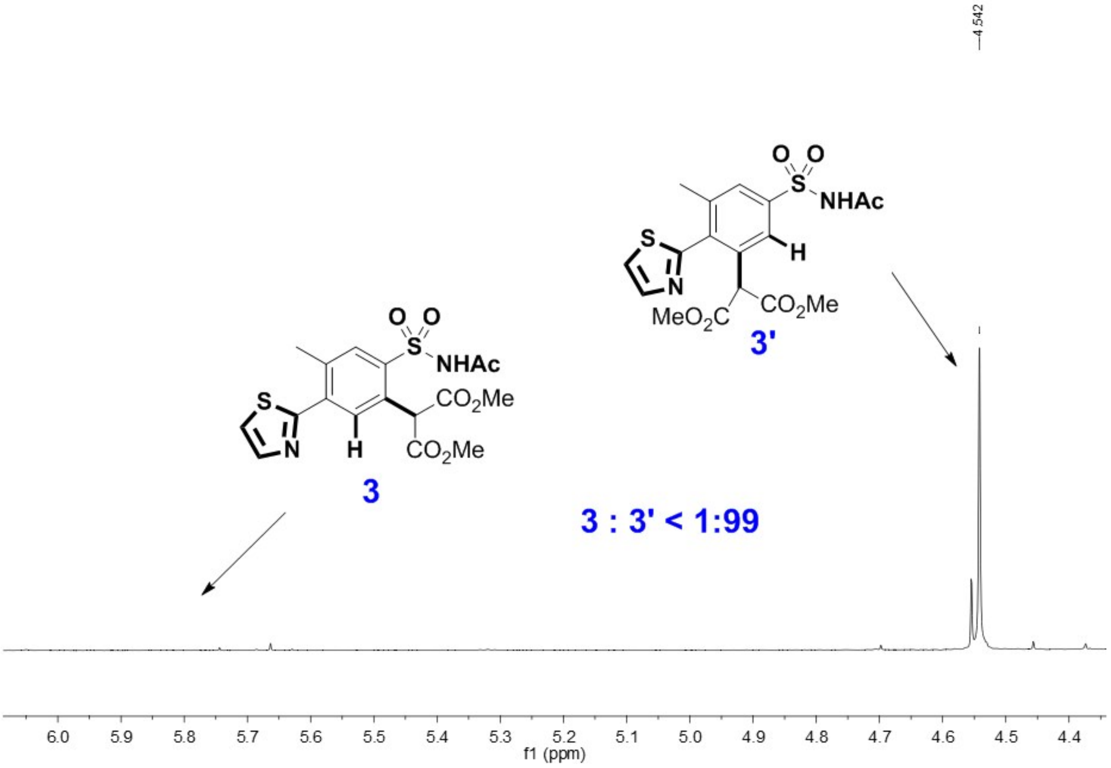

__Table 1, entry 2 (Rh:5%, AgOAc:20%, DMF, 0.05M)__

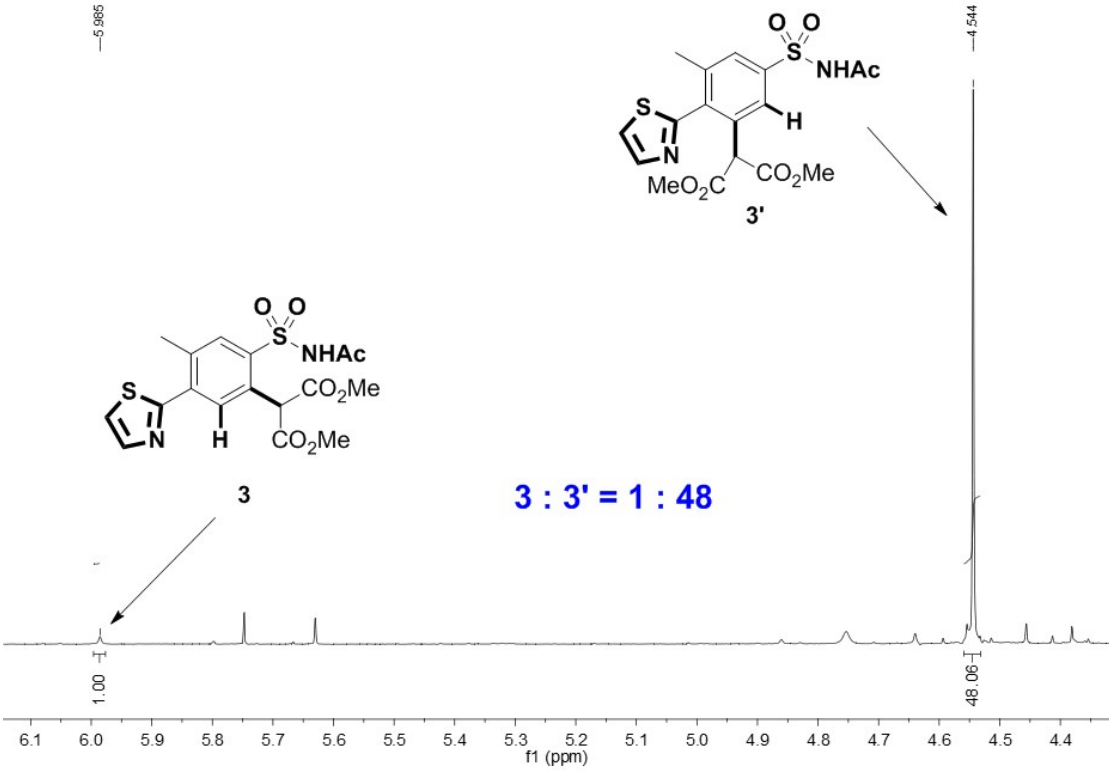

Table 1, entry 3 (Rh:5%, AgOAc:20%, MeCN, 0.05M)

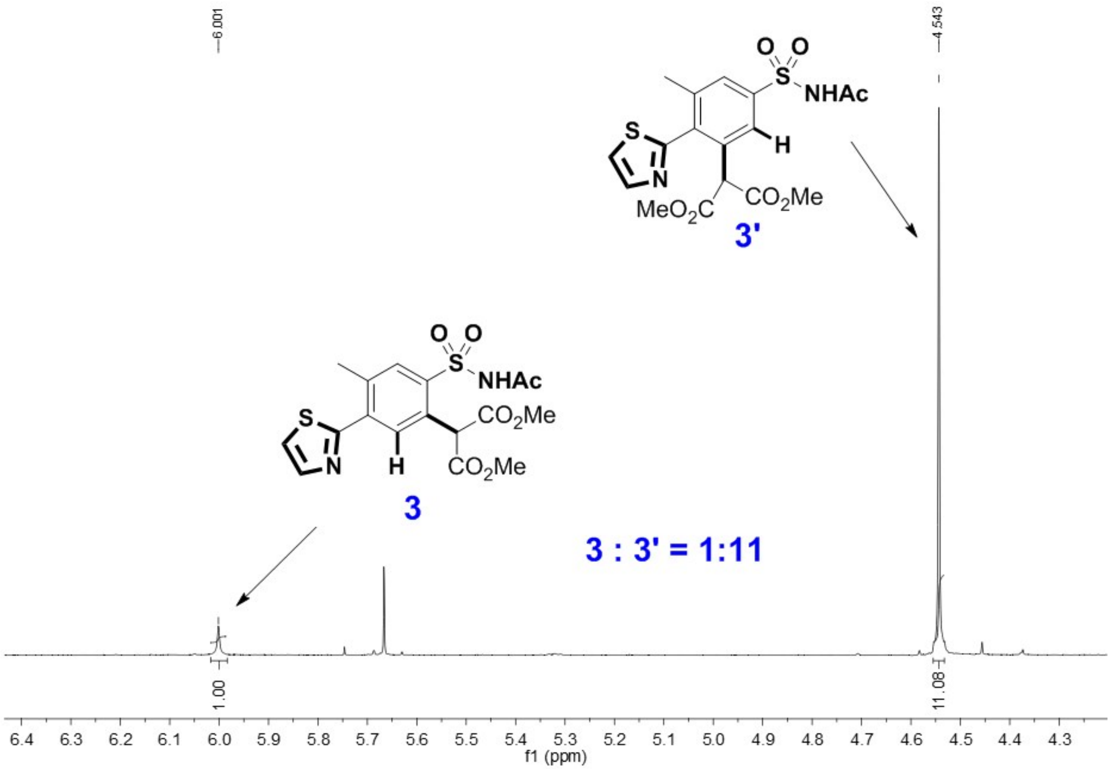

Table 1, entry 4 (Rh:5%, AgOAc:20%, DCE, 0.05M)

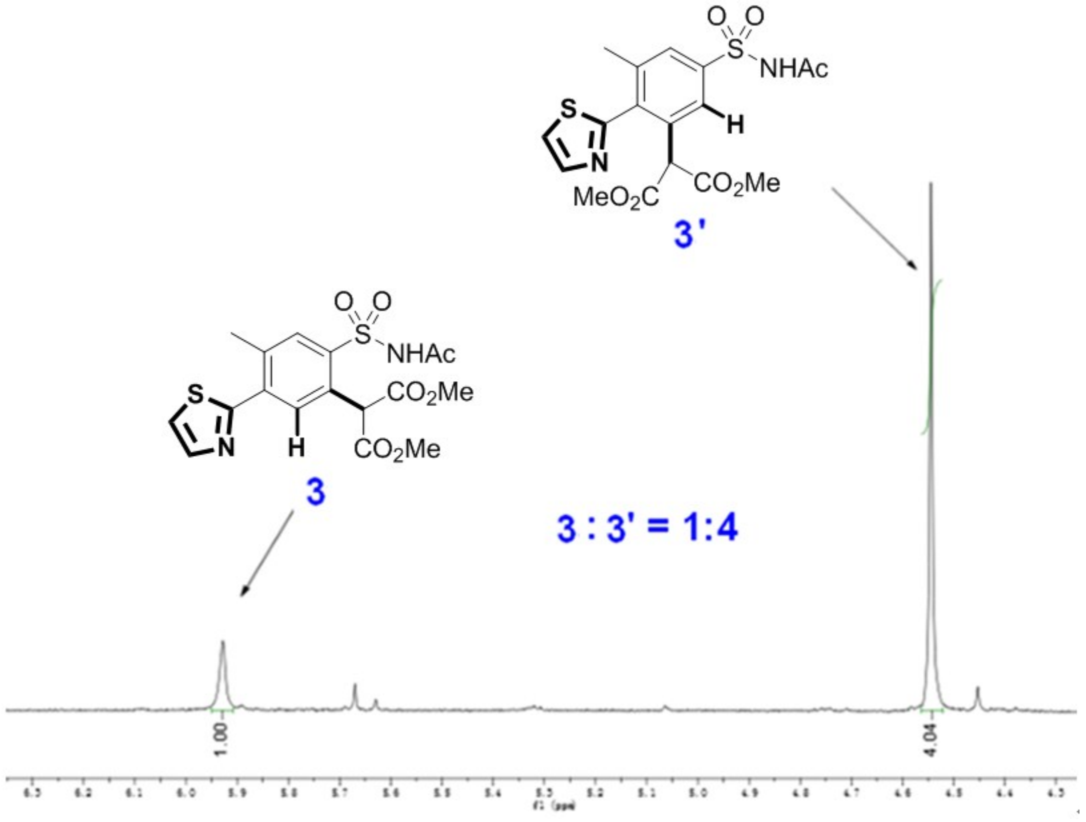

__Table 1, entry 5 (Rh:5%, AgOAc:20%, toluene, 0.05M)__

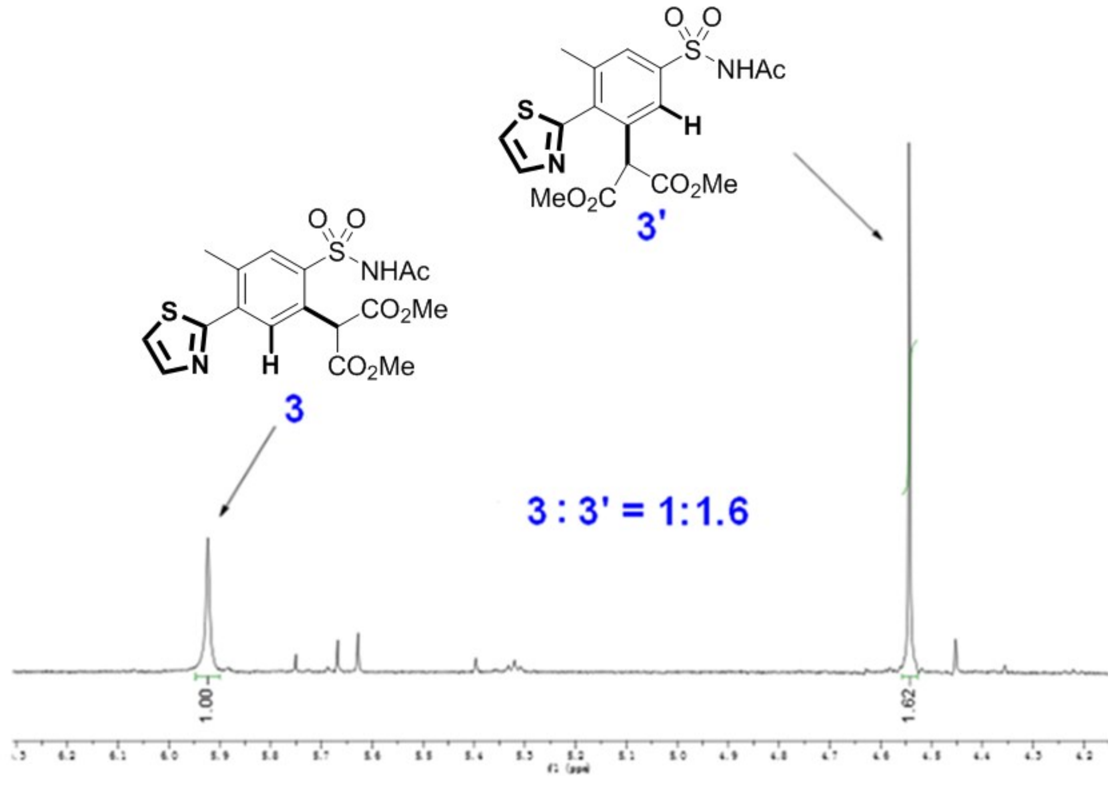

__Table 1, entry 6 (Rh:5%, AgOAc:20%, DCE, 0.01M)__

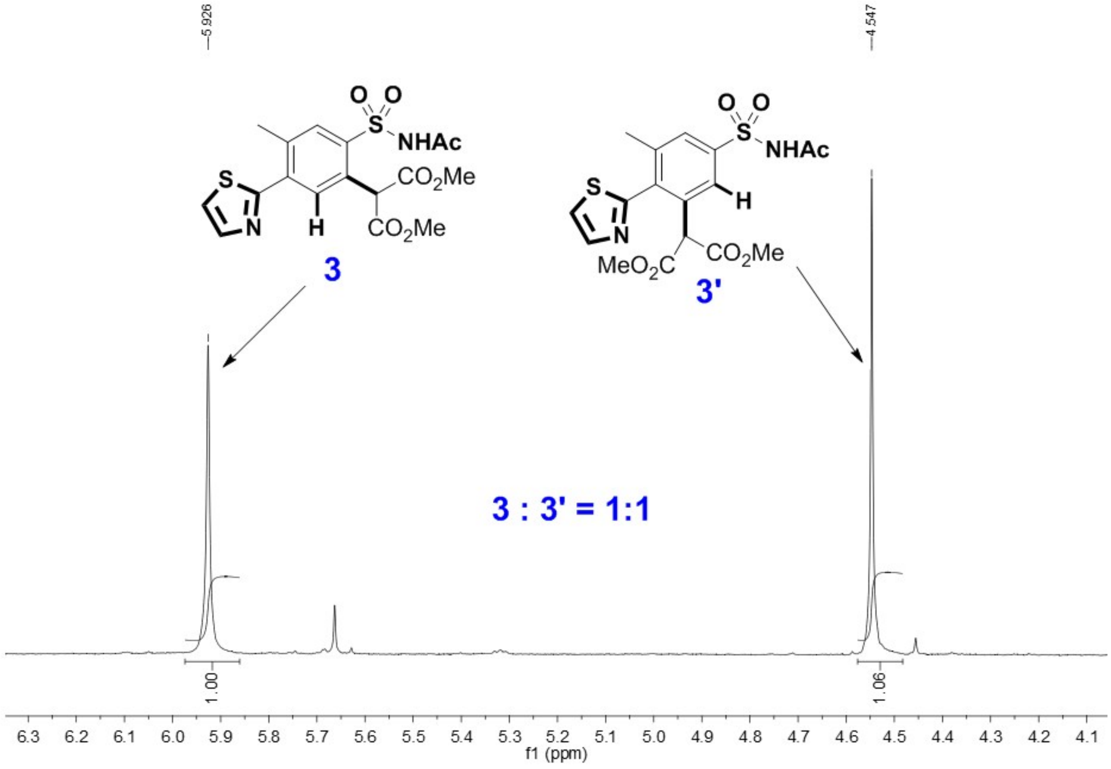

Table 1, entry 7 (Rh:5%, AgOAc:20%, Toluene, 0.01M)

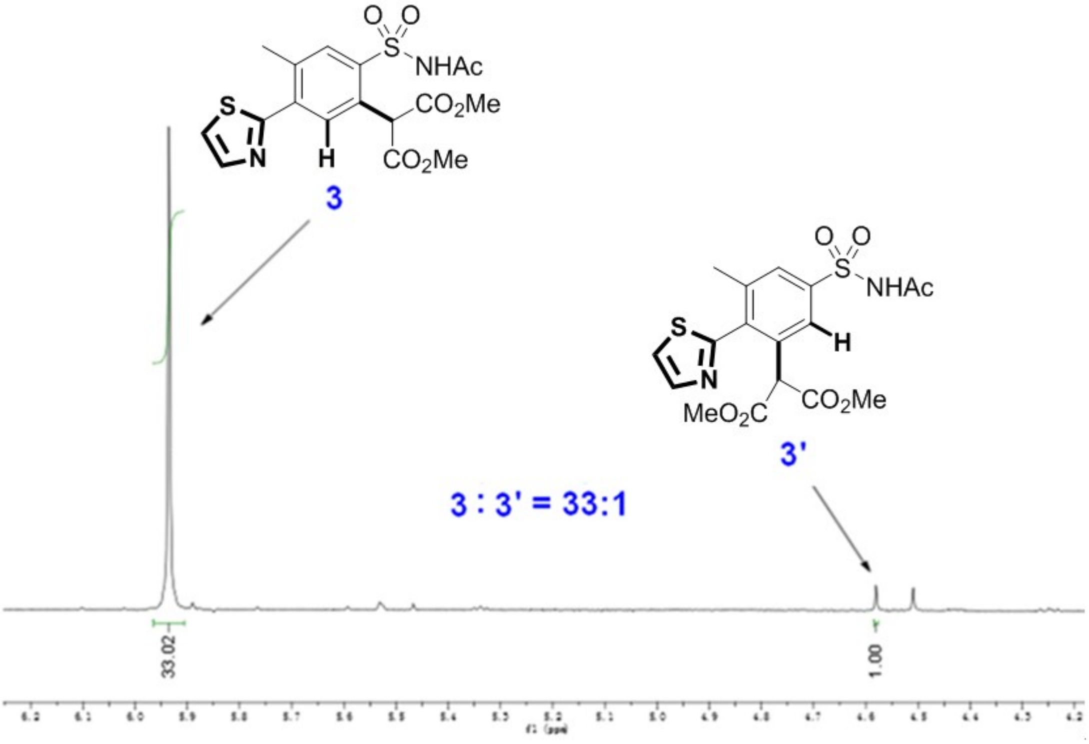

Table 1, entry 8 (Rh:5%, AgOAc:20%, Toluene/DCE=1:1, 0.01M)

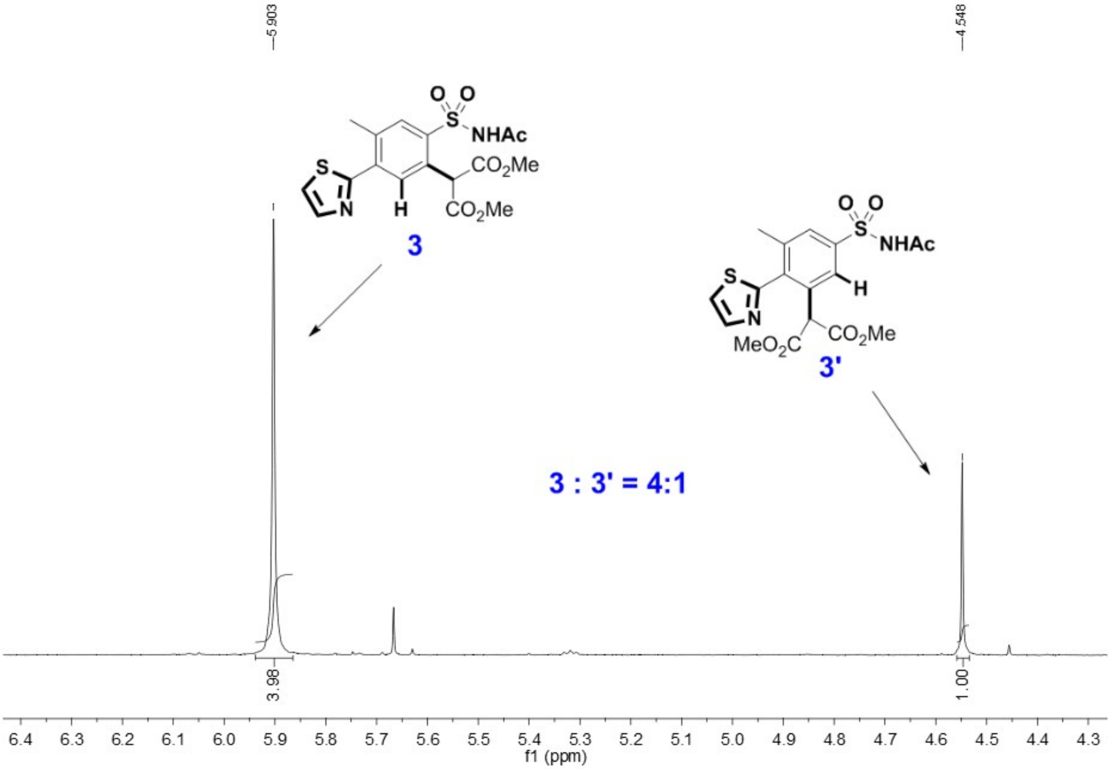

Table 1, entry 9 (Rh:5%, AgOAc:20%, Toluene, 0.005M)

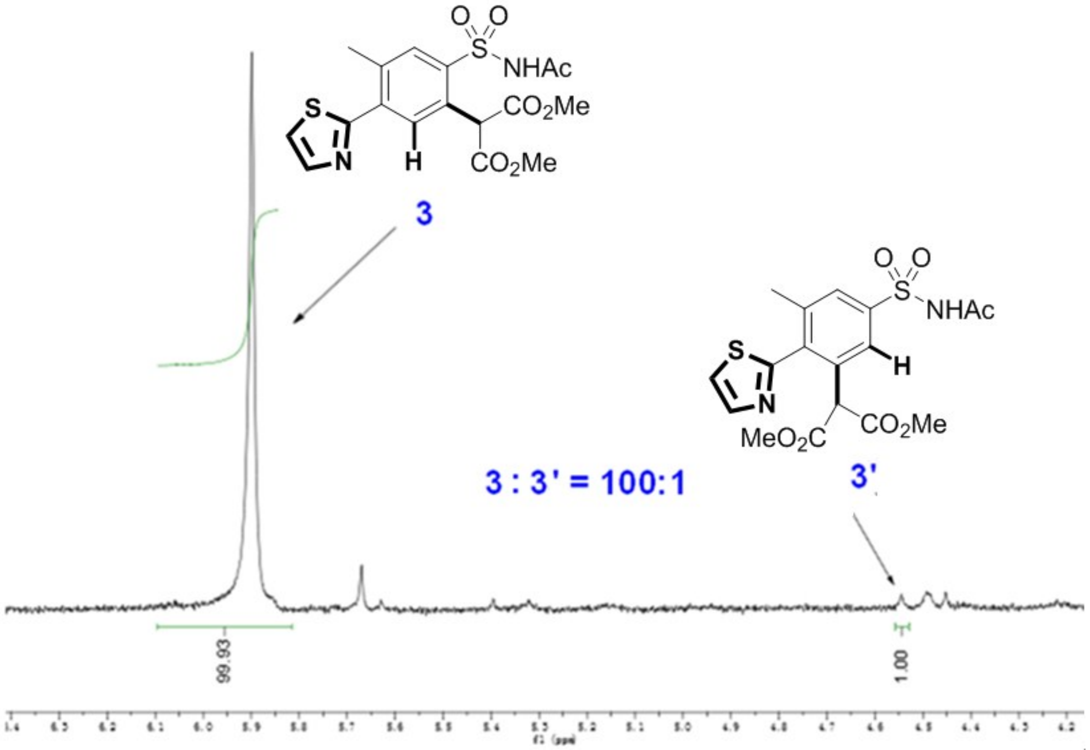

Table 1, entry 10 (Rh:2.5%, AgOAc:10%, Toluene, 0.005M)

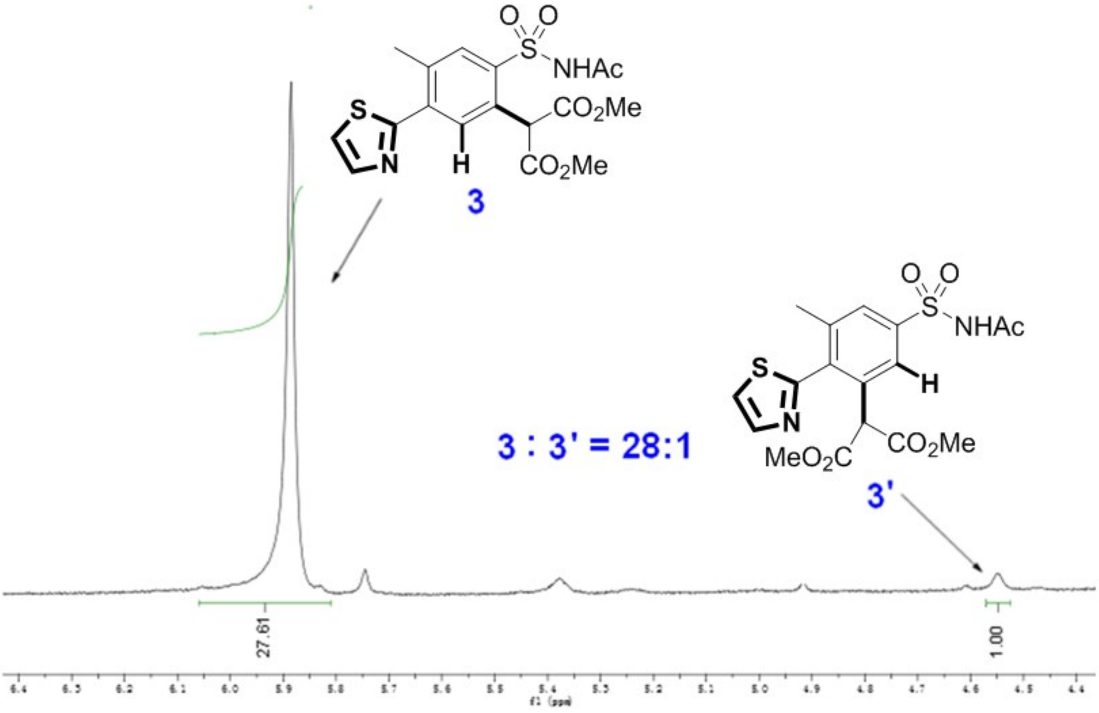

Table 1, entry 11 (Rh: 5%, AgOAc:20%, DMF, 0.25M)

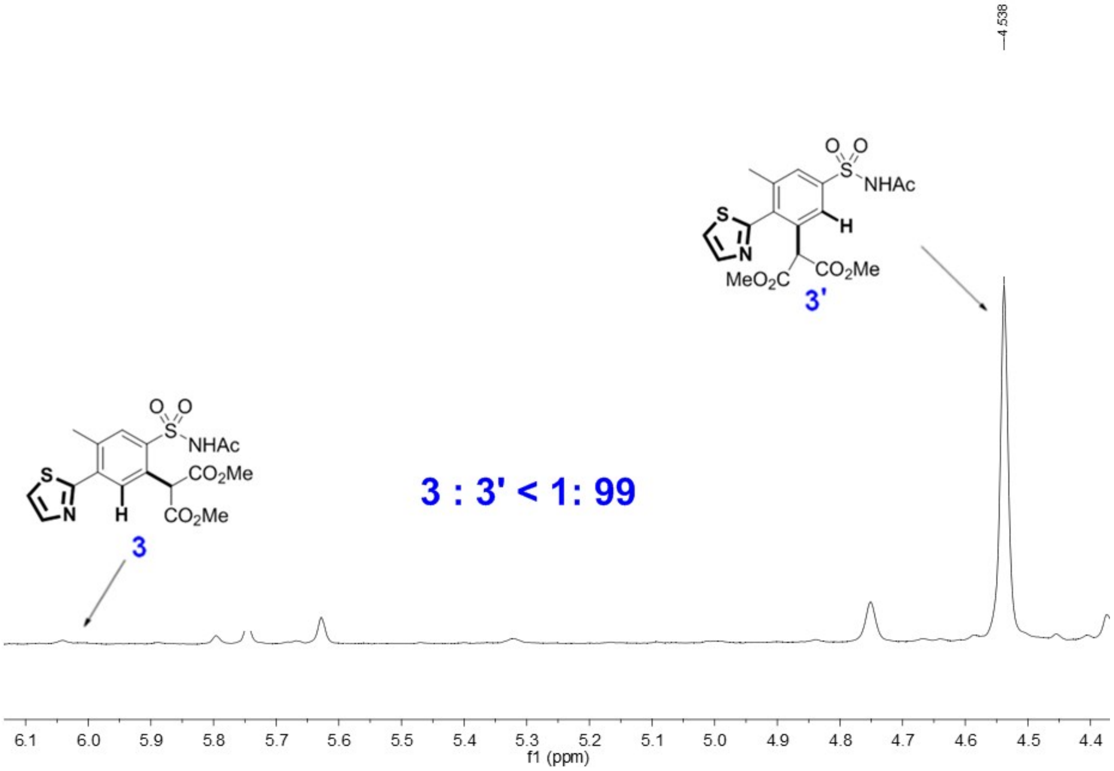

Table 1, entry 12 (Rh: 5%, AgOAc:20%, MeCN, 0.25M)

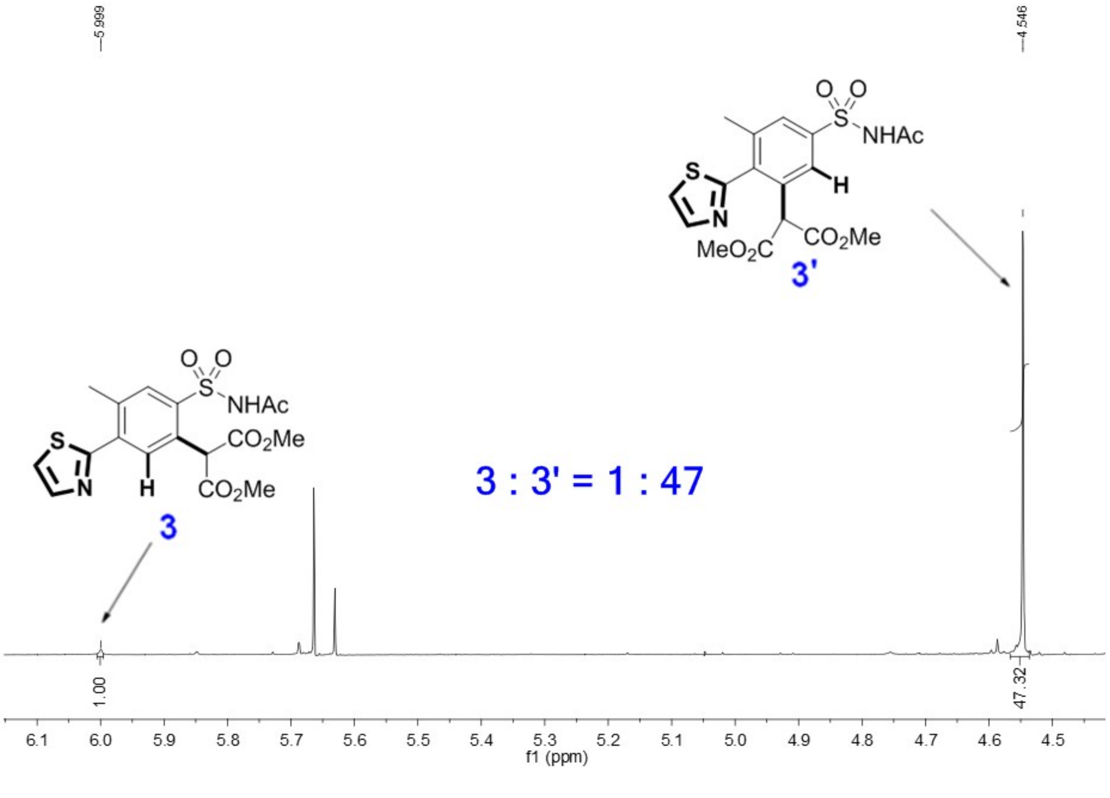

__Table 1, entry 13 (Rh: 5%, AgOAc:40%, DCE, 0.25M)__

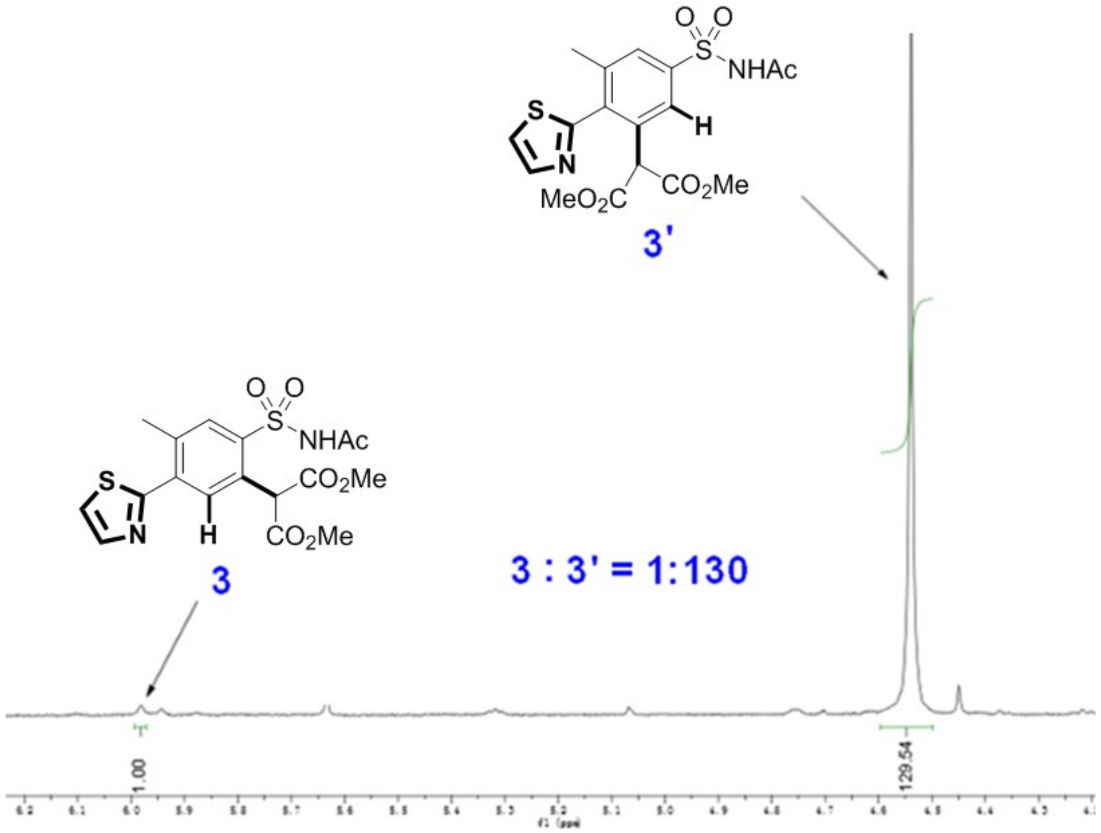

__Table 1, entry 14 (Rh:5%, AgOAc:60%, DCE, 0.05M)__

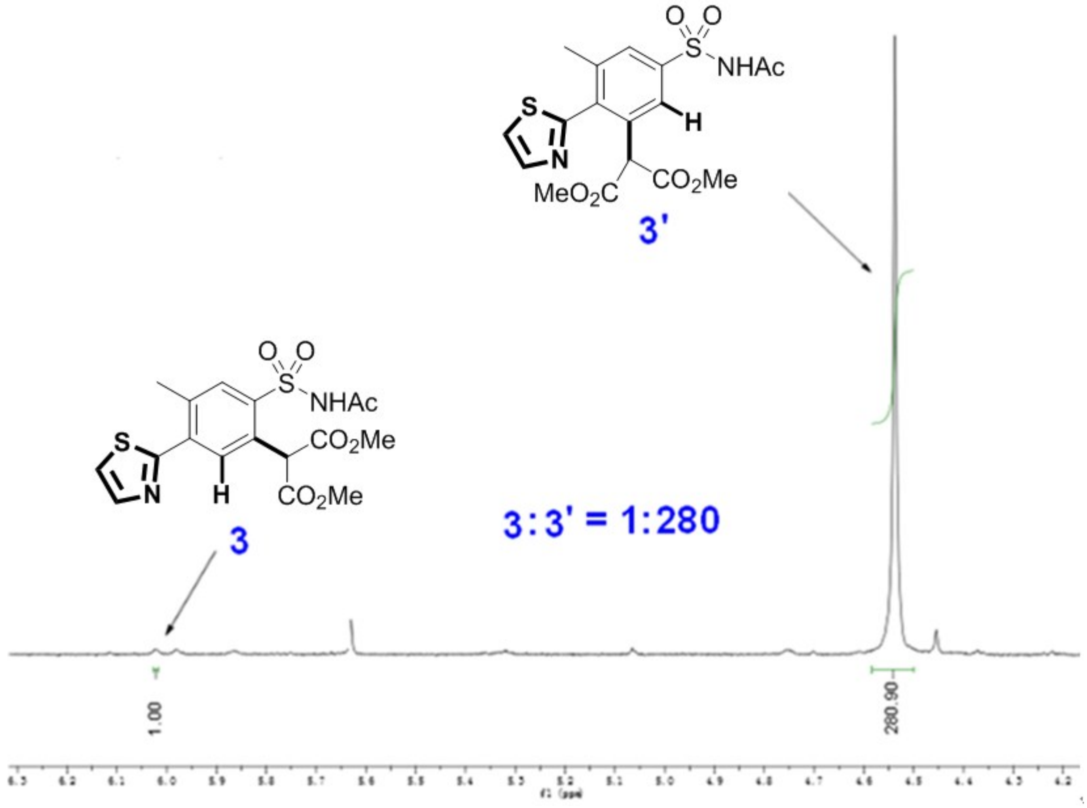

Table 1, entry 15 (Rh:2.5%, AgOAc:60%, DCE, 0.05M)

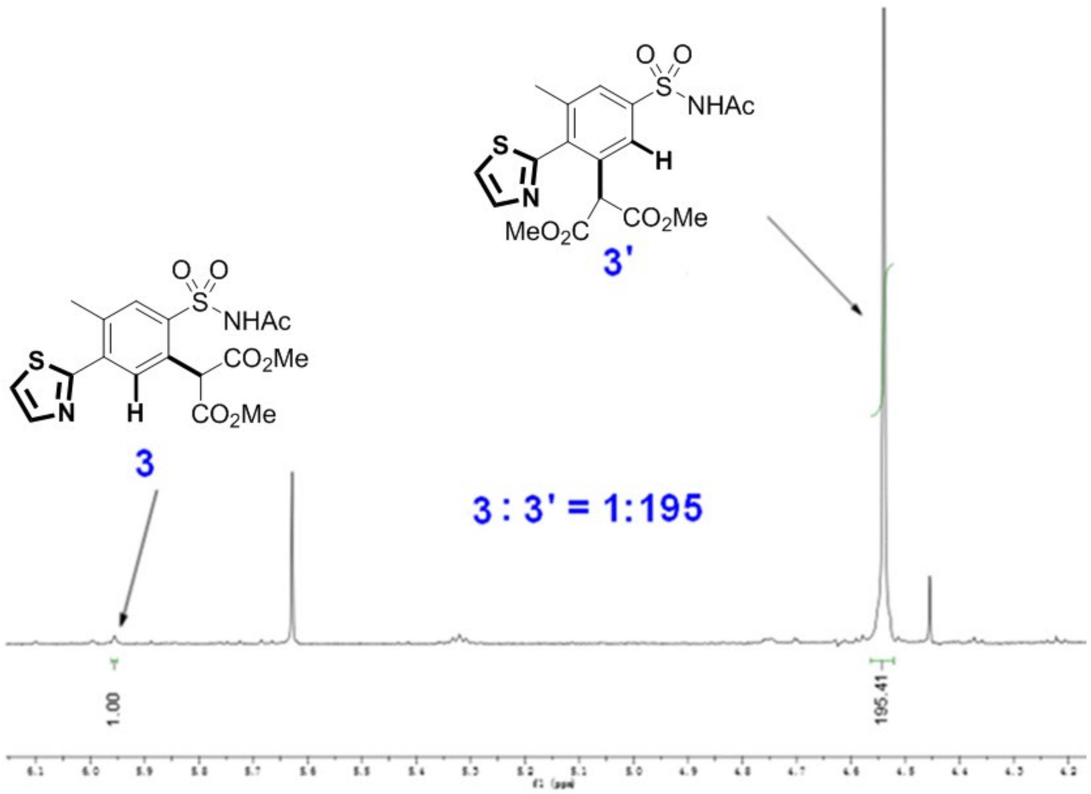

Table 1, entry 16 (Rh:2.5%, AgOAc:20%, DCE, 0.05M)

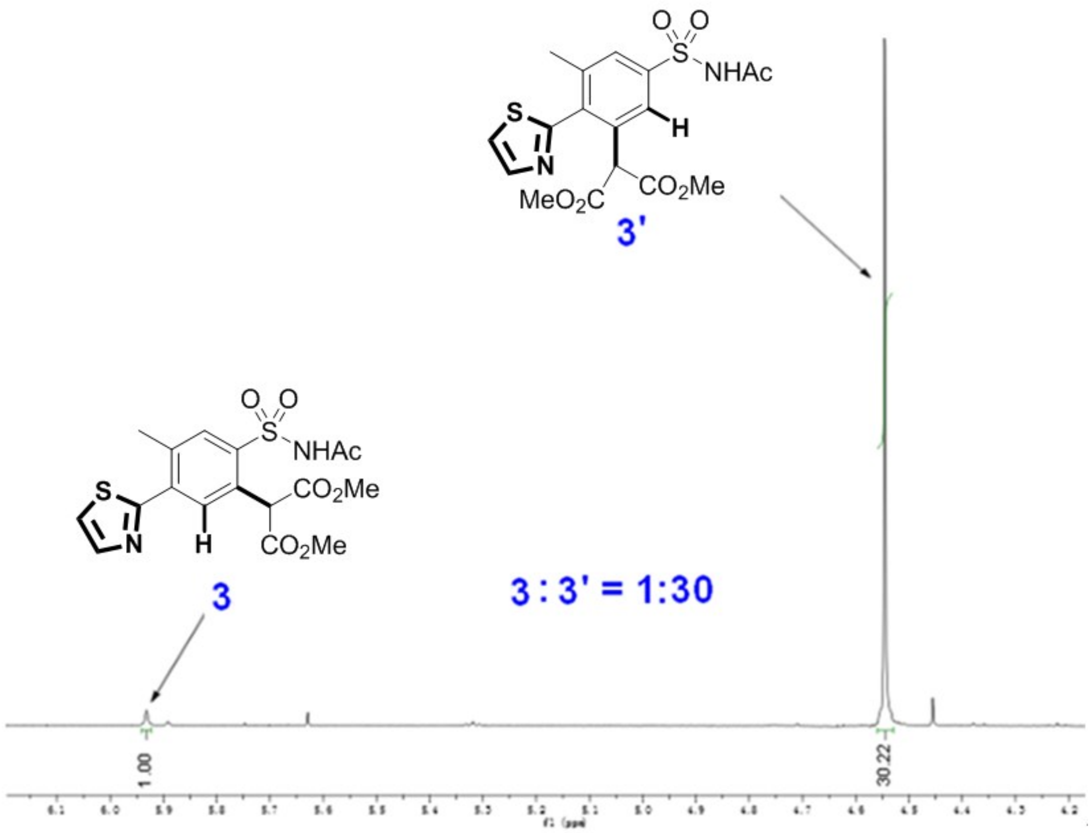

Table 1, entry 17 (Rh:5%, $^{n}Bu_4NOAc$:20%, toluene, 0.005M)

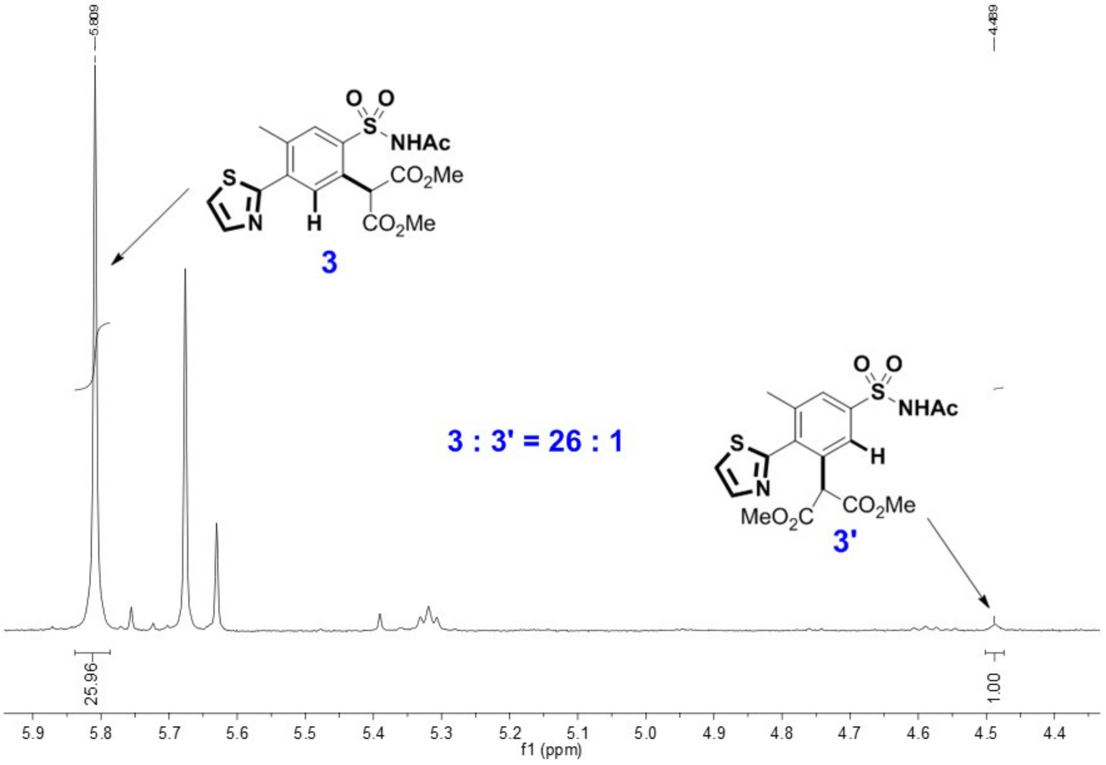

Table 1, entry 18 (Rh:2.5%, $^{n}Bu_4NOAc$:60%, DCE, 0.05M)

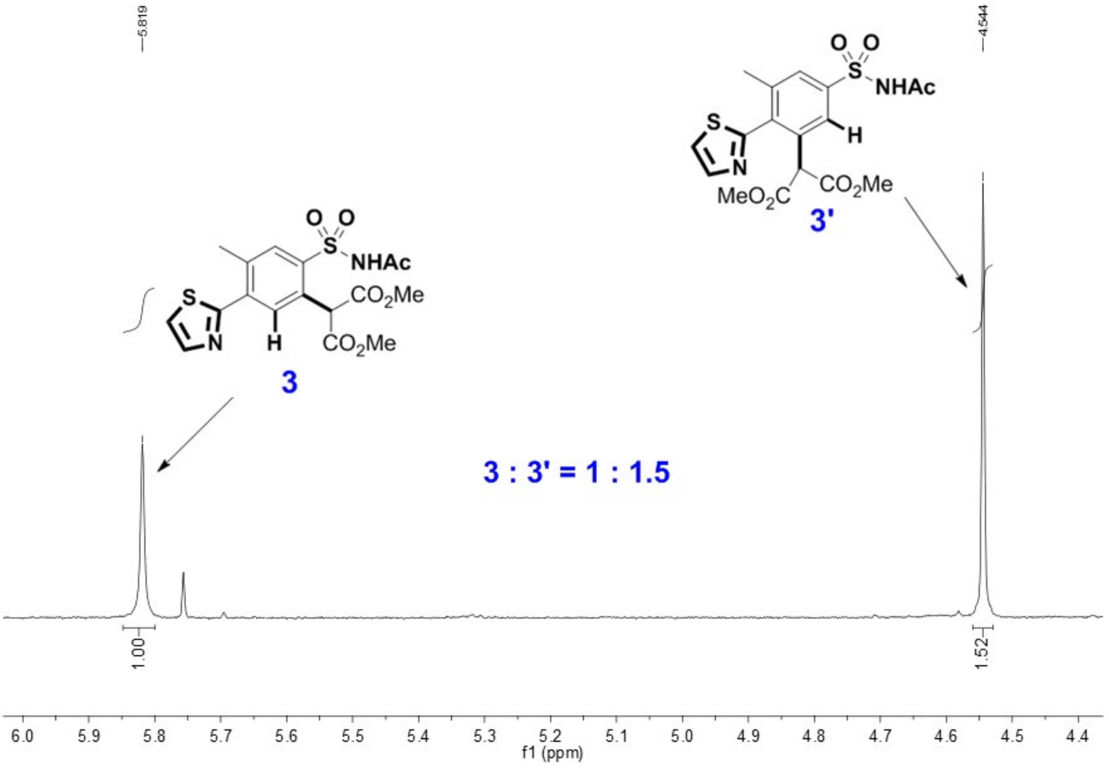

The active catalyst of this C–H carbenoid functionalization reaction: [RhCp*(OAc)$_2$]

In the selected literatures[1-5], [RhCp*(OAc)$_2$] was commonly recognized as an active catalyst in the [RhCp*Cl$_2$]$_2$/OAc$^-$ catalytic system. Moreover, in our experiments, RhCp*(OAc)$_2$ could be prepared in DCM at room temperature in good yield from [RhCp*Cl$_2$]$_2$ and AgOAc. The Rh(III)-catalyzed site-selective C–H carbenoid functionalization of N-((2-methyl-4-(1H-pyrazol-1-yl)phenyl)sulfonyl)acetamide was catalyzed by RhCp*(OAc)$_2$ in DCE (0.05M concentration) at 60 °C, and the corresponding C-H activated products were obtained in 86% yield (**25** + **7** = 66% + 20%, **25**/**7** = 3:1). These results were consistent with the results of the [RhCp*Cl$_2$]$_2$/AgOAc (5%/20%) catalytic system, which further indicated that RhCp*(OAc)$_2$ was the active catalyst under this [RhCp*Cl$_2$]$_2$/AgOAc catalytic system.

Synthesis of [RhCp*(OAc)$_2$]

$$[\mathrm{RhCp^*Cl_2}]_2 + \mathrm{AgOAc} \xrightarrow[\mathrm{DCM,\ rt}]{} \mathrm{RhCp^*(OAc)_2}$$

AgOAc (4.7 mmol, 778 mg) was added to a solution of [RhCp*Cl$_2$]$_2$ (0.97 mmol, 600 mg) in anhydrous DCM (20 mL). th deep-red solution was stirred at room temperature for 4 days. The precipitate was filtered off and washed with DCM. The filtrate was concentrated in *vacuo* to afford RhCp*(OAc)$_2$ as an orange oil (553 mg, 80% yield).

[RhCp*(OAc)$_2$] catalyzed C–H carbenoid functionalization reaction:

A 15 mL tube equipped with a magnetic stir bar was charged with RhCp*(OAc)$_2$ (10 mol%, 4.5 mg), N-((2-methyl-4-(1H-pyrazol-1-yl)phenyl)sulfonyl)acetamide (0.125 mmol, 35 mg), and 2.5 mL DCE, then diazo compound **2a** (1.1 equivlent) was added. The tube was sealed, and the reaction mixture was stirred at 60°C over night. DCE was removed under *vacuo*, and 20 mL DCM was added. The mixture was then filtered, the filtrate was concentrated, the ratio of **25** and **7** was determined by ^{1}H NMR analysis of the crude reaction mixture before separation and the residue was purified by preparative TLC on silica gel to afford **25** (33.5 mg, 66%) and **7** (10.1 mg, 20%).

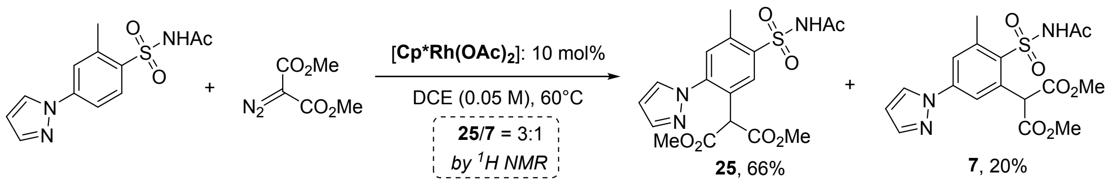

Computational Details

Density functional theory (DFT) was employed using Gaussian 16[6] program package. Pure DFT functionals were believed to appropriately describe the singlet-triplet energy difference of metal-carbene complexes.[7] Therefore, the pure DFT functionals BPW91[8] in combination with basis sets I (denoted as BSI: SDD[9] with Stuttgart/Dresden effective core potentials (ECPs) for Rh atoms, 6-31+G* for others) were employed for geometry optimization and frequency analysis. Both M06L[10] and ωB97XD[11] functionals were utilized for single-point energy calculation in liquid phase. As provided in Table S1, the ωB97XD-reported ΔG value in DCE was 2.1 kcal/mol, which is agreeable with the experimental deuteration facts that both ortho-sulfonamide and ortho-thiazole C-H functionalization products were obtained. Therefore, the solvation single-point energy refinement was conducted at the level of ωB97XD/BSII (BSII: SDD with ECPs for Rh atoms, 6-311++G** for others). The solvation effects were estimated by using polarized continuum model (PCM)[12] with SMD-Coulomb[12b] radii. All of the thermodynamic data were obtained at 298.15K.

Table S5. Calculated free energy gap (denoted as ΔG (in kcal/mol)) between TS1 and TS1' in the cases of DCE and toluene solvents mediated by RhCp*(OAc)2 complex.

Solvent	DCE	toluene
ΔG (M06L)a	4.3	12.5
ΔG (ωB97XD)b	2.1	8.6

a. Calculated at the level of M06L/BSII// BPW91/BSI. b. Calculated at the level of ωB97XD/BSII// BPW91/BSI.

Computational Results

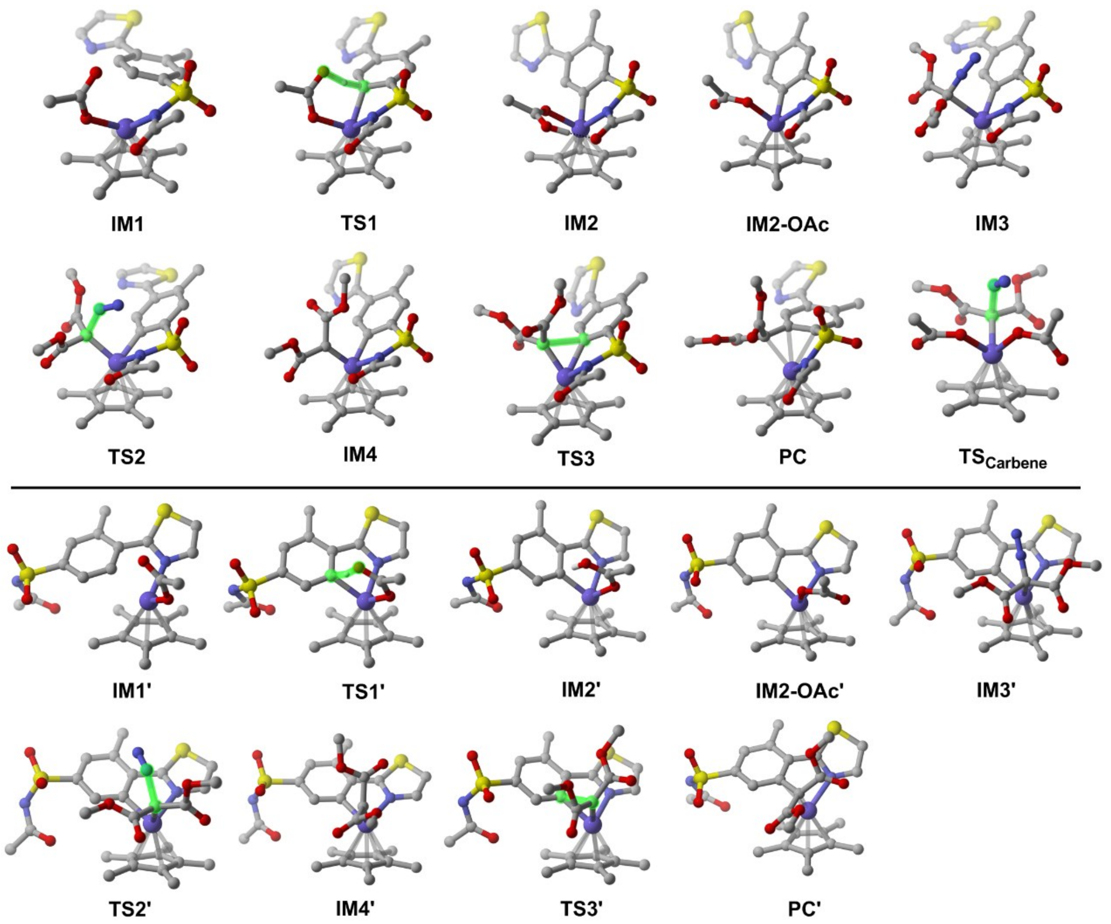

Figure S1. Depicted are the snapshots of optimized structures. All C-bound hydrogens were omitted for clarity. Key transition bonds were highlighted in light green.

Another metal-carbene formation transition state TSCarbene was also located, in which the active catalyst RhCp*(OAc)2 directly react with carbene-precursor 2a and two OAc- ligands were mono-coordinated to the rhodium(Ⅲ) center. The free energy of TSCarbene was obviously higher than C-H activation transition states TS1 and TS1’ in either toluene or DCE, which was consistent with the experimental results that O-carbenoid functionalization products were not obtained.

References:

1. F. Hu, Y. Xia, F. Ye, Z. Liu, C. Ma, Y. Zhang, J. Wang, *Angew. Chem. Int. Ed.,* **2014**, *53*, 1364−1367.
2. Y. Liang, K. Yu, B. Li, S. Xu, H. Song, B. Wang, *Chem. Commun.*, **2014**, *50*, 6130−6133.
3. N. K. Mishra, M. Choi, H. Jo, Y. Oh, S. Sharma, S. H. Han, T. Jeong, S. Han, S.-Y. Lee, I. S. Kim, *Chem. Commun.*, **2015**, *51*, 17229−17232.
4. B. Zhou, Z. Chen, Y. Yang, W. Ai, H. Tang, Y. Wu, W. Zhu, Y. Li, *Angew. Chem. Int. Ed.,* **2015**, *54*, 12121−12126.
5. W.-W. Chan, S.-F. Lo, Z. Zhou, W.-Y. Yu, *J. Am. Chem. Soc.*, **2012**, *134*, 13565−13568
6. M. J. Frisch, G. W. T., H. B. Schlegel, G. E. Scuseria, ; M. A. Robb, J. R. C., G. Scalmani, V. Barone, ; G. A. Petersson, H. N., X. Li, M. Caricato, A. V. Marenich, ; J. Bloino, B. G. J., R. Gomperts, B. Mennucci, H. P. Hratchian, ; J. V. Ortiz, A. F. I., J. L. Sonnenberg, D. Williams-Young, ; F. Ding, F. L., F. Egidi, J. Goings, B. Peng, A. Petrone, ; T. Henderson, D. R., V. G. Zakrzewski, J. Gao, N. Rega, ; G. Zheng, W. L., M. Hada, M. Ehara, K. Toyota, R. Fukuda, ; J. Hasegawa, M. I., T. Nakajima, Y. Honda, O. Kitao, H. Nakai, ; T. Vreven, K. T., J. A. Montgomery, Jr., J. E. Peralta, ; F. Ogliaro, M. J. B., J. J. Heyd, E. N. Brothers, K. N. Kudin, ; V. N. Staroverov, T. A. K., R. Kobayashi, J. Normand, ; K. Raghavachari, A. P. R., J. C. Burant, S. S. Iyengar, ; J. Tomasi, M. C., J. M. Millam, M. Klene, C. Adamo, R. Cammi, ; J. W. Ochterski, R. L. M., K. Morokuma, O. Farkas, ; J. B. Foresman, a. D. J. F. Gaussian, Inc.: Wallingford CT, 2016.
7. a) X. Lin, C. Zhao, C.-M. Che, Z. Ke, D. L. Phillips, *Chem. - Asian J.* **2007,** *2*, 1101-1108; b) X. Lin, C.-M. Che, D. L. Phillips, *J. Org. Chem.* **2007,** *73*, 529-537; c) X. Lin, Y. Xi, J. Sun, *Comput. Theor. Chem.* **2012,** *999*, 74-82; d) X. Zhang, Z. Ke, N. J. DeYonker, H. Xu, Z.-F. Li, X. Xu, X. Zhang, C.-Y. Su, D. L. Phillips, C. Zhao, *J. Org. Chem.* **2013,** *78*, 12460-12468; e) X. Zhang, H. Xu, C. Zhao, *J. Org. Chem.* **2014,** *79*, 9799-9811.
8. a) A. D. Becke, *Phys. Rev. A* **1988,** *38*, 3098; b) J. P. Perdew, J. A. Chevary, S. H. Vosko, K. A. Jackson, M. R. Pederson, D. J. Singh, C. Fiolhais, *Phys. Rev. B* **1992,** *46*, 6671-6687.
9. D. Andrae, U. Häußermann, M. Dolg, H. Stoll, H. Preuß, *Theor. Chem. Acc.* **1990,** *77*, 123-141.
10. Y. Zhao, D. G. Truhlar , *Theor Chem Acc.* **2008,** *120*, 215-241.
11. J.-D. Chai, M. Head-Gordon, *Phys. Chem. Chem. Phys.* **2008,** *10*, 6615-6620.
12. a) J. Tomasi, B. Mennucci, R. Cammi, *Chem. Rev.* **2005,** *105*, 2999-3094; b) A. V. Marenich, C. J. Cramer, D. G. Truhlar, *J. Phys. Chem. B* **2009,** *113* (18), 6378-6396.

The ratio of 22 and 22' by method A (Scheme 1)

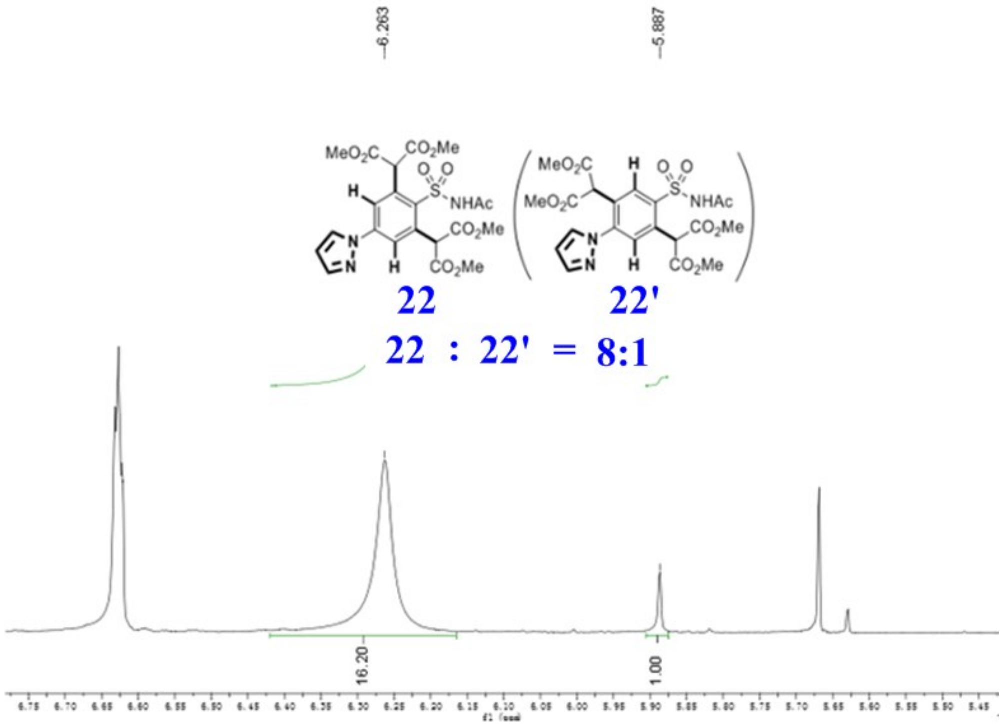

Intermolecular competition between sulfonamide and pyridine group by method A (Scheme 2, A)

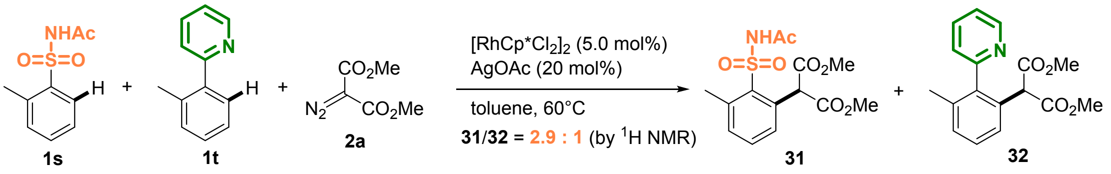

A 50 mL tube equipped with a magnetic stir bar was charged with $[RhCp^*Cl_2]_2$ (5.0 mol%), AgOAc (20 mol%), **1s** (0.125 mmol), **1t** (0.125 mmol) and 25 mL toluene, then diazo compound **2a** (1.1 equivlent) was added. The tube was sealed, and the reaction mixture was stirred at 60°C over night. Toluene was removed under *vacuo*, and 20 mL DCM was added. The mixture was then filtered, the filtrate was concentrated, the ratio of **31** and **32** was determined by ^{1}H NMR analysis of the crude reaction mixture before separation and the residue was purified by preparative TLC on silica gel to afford **31** as a major product (28 mg, 66%).

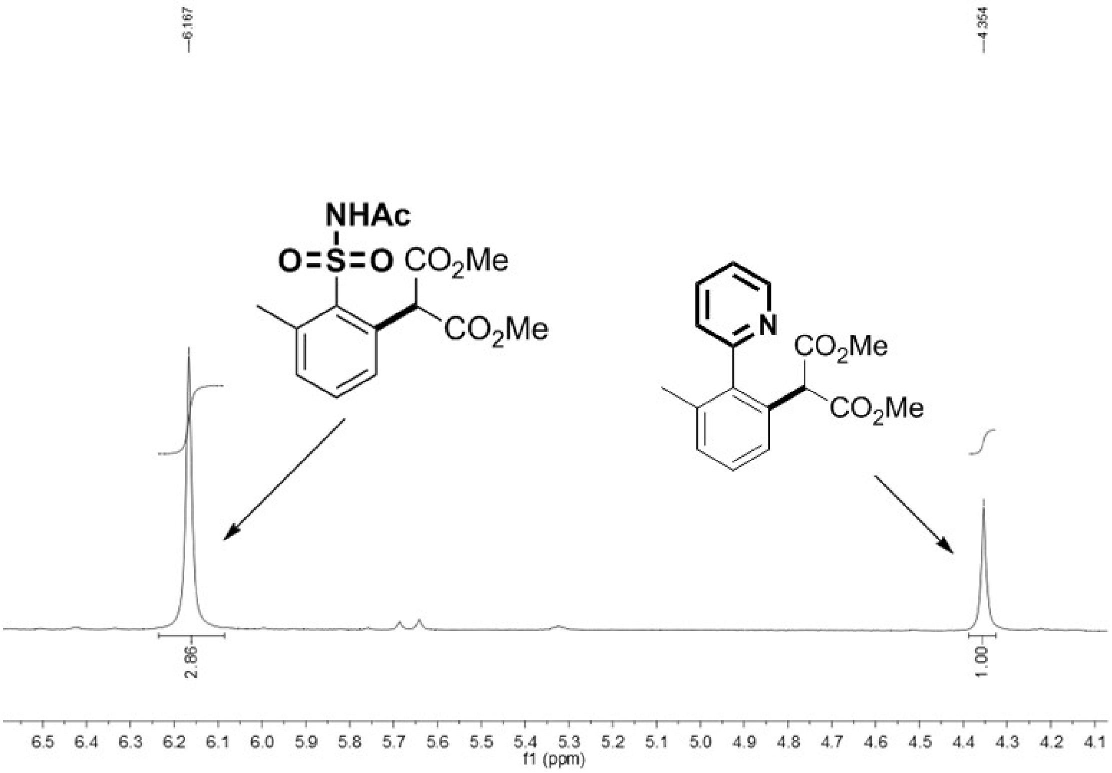

Intermolecular competition between sulfonamide and pyridine group by method B (Scheme 2, A)

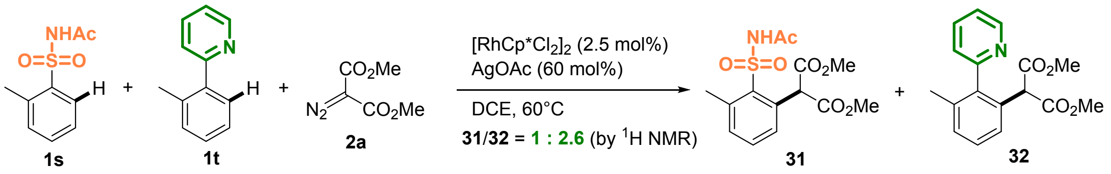

A 15 mL tube equipped with a magnetic stir bar was charged with [RhCp*Cl$_2$]$_2$ (2.5 mol%), AgOAc (60 mol%), **1s** (0.125 mmol), **1t** (0.125 mmol) and 2.5 mL DCE, then diazo compound **2a** (1.1 equivlent) was added. The tube was sealed, and the reaction mixture was stirred at 60°C over night. DCE was removed under *vacuo*, and 20 mL DCM was added. The mixture was then filtered, the filtrate was concentrated, the ratio of **31** and **32** was determined by ^{1}H NMR analysis of the crude reaction mixture before separation and the residue was purified by preparative TLC on silica gel to afford **32** as a major product (25.2 mg, 68%).

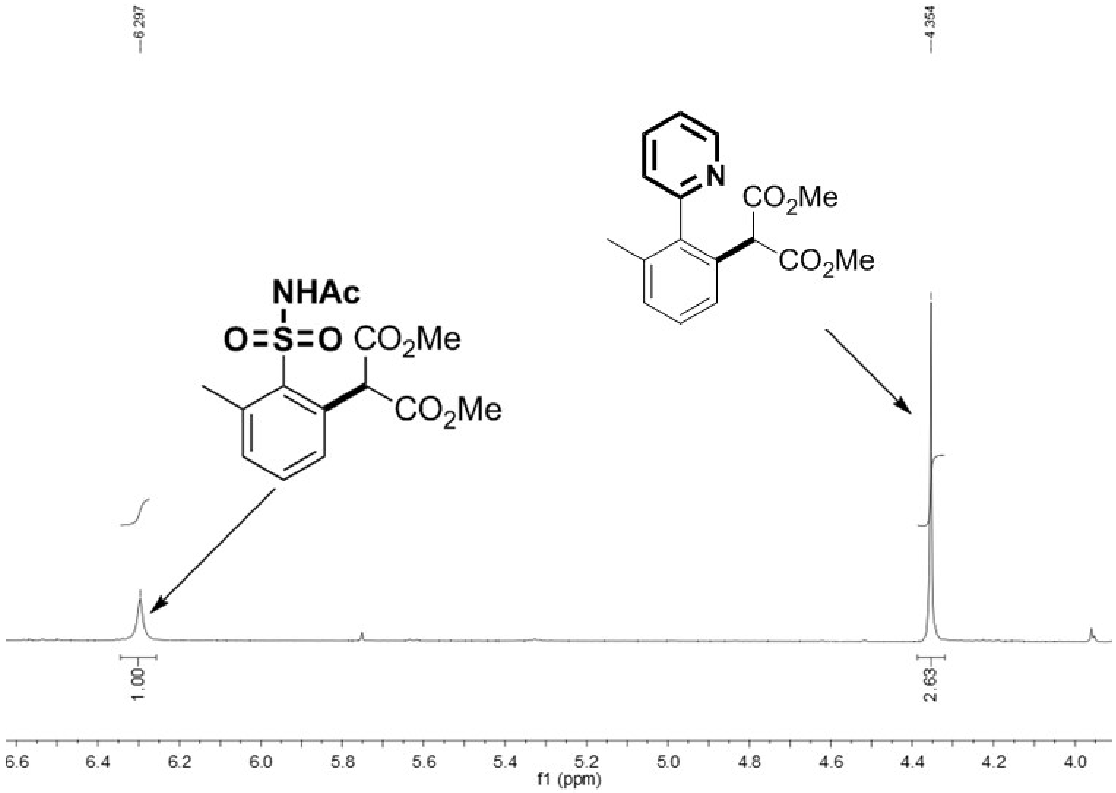

Intermolecular competition between sulfonamide and pyridine group by method A (Scheme 2, B)

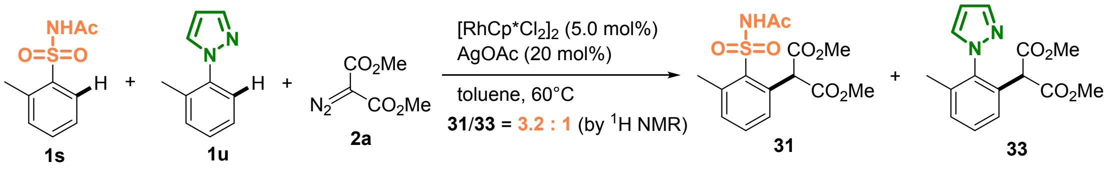

A 50 mL tube equipped with a magnetic stir bar was charged with $[RhCp^*Cl_2]_2$ (5.0 mol%), AgOAc (20 mol%), **1s** (0.125 mmol), **1u** (0.125 mmol) and 25 mL toluene, then diazo compound **2a** (1.1 equivlent) was added. The tube was sealed, and the reaction mixture was stirred at 60°C over night. Toluene was removed under *vacuo*, and 20 mL DCM was added. The mixture was then filtered, the filtrate was concentrated, the ratio of **31** and **33** was determined by ^{1}H NMR analysis of the crude reaction mixture before separation and the residue was purified by preparative TLC on silica gel to afford **31** as a major product (29.3 mg, 68%).

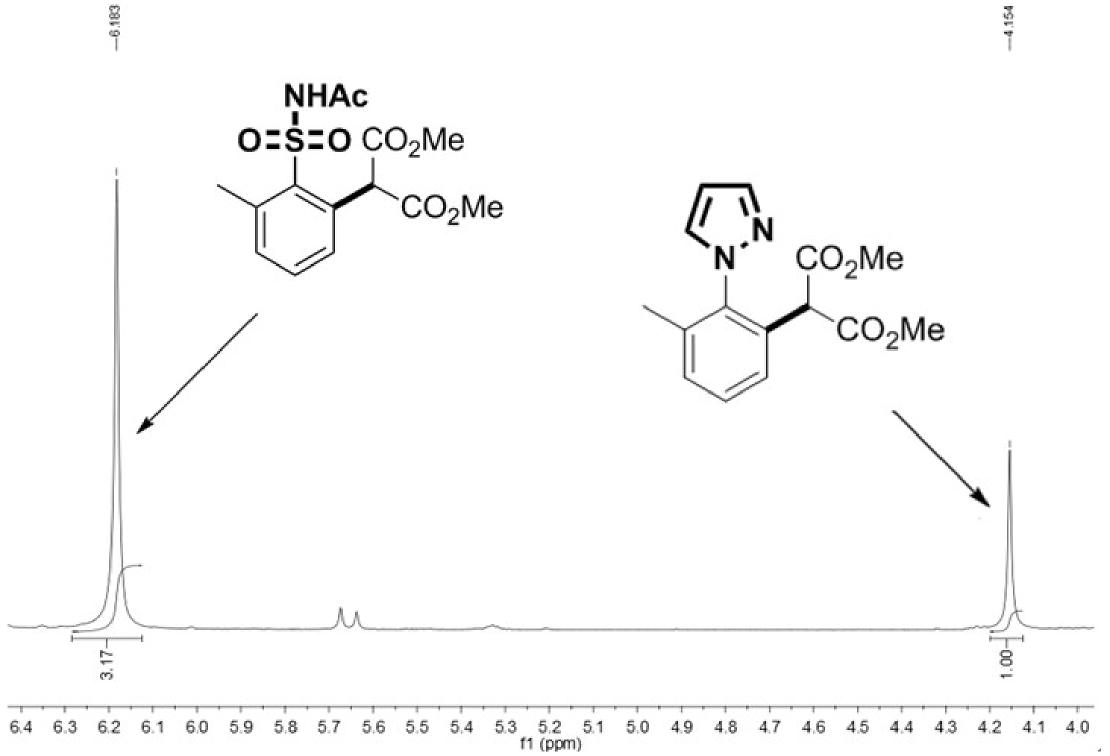

Intermolecular competition between sulfonamide and pyridine group by method B (Scheme 2, B)

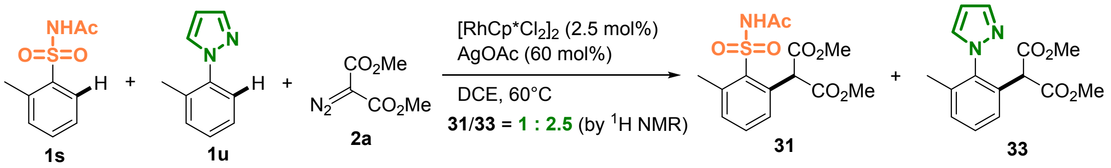

A 15 mL tube equipped with a magnetic stir bar was charged with [RhCp*Cl2]2 (2.5 mol%), AgOAc (60 mol%), **1s** (0.125 mmol), **1u** (0.125 mmol) and 2.5 mL DCE, then diazo compound **2a** (1.1 equivlent) was added. The tube was sealed, and the reaction mixture was stirred at 60°C over night. DCE was removed under *vacuo*, and 20 mL DCM was added. The mixture was then filtered, the filtrate was concentrated, the ratio of **31** and **33** was determined by ^{1}H NMR analysis of the crude reaction mixture before separation and the residue was purified by preparative TLC on silica gel to afford **33** as a major product (23.9 mg, 66%).

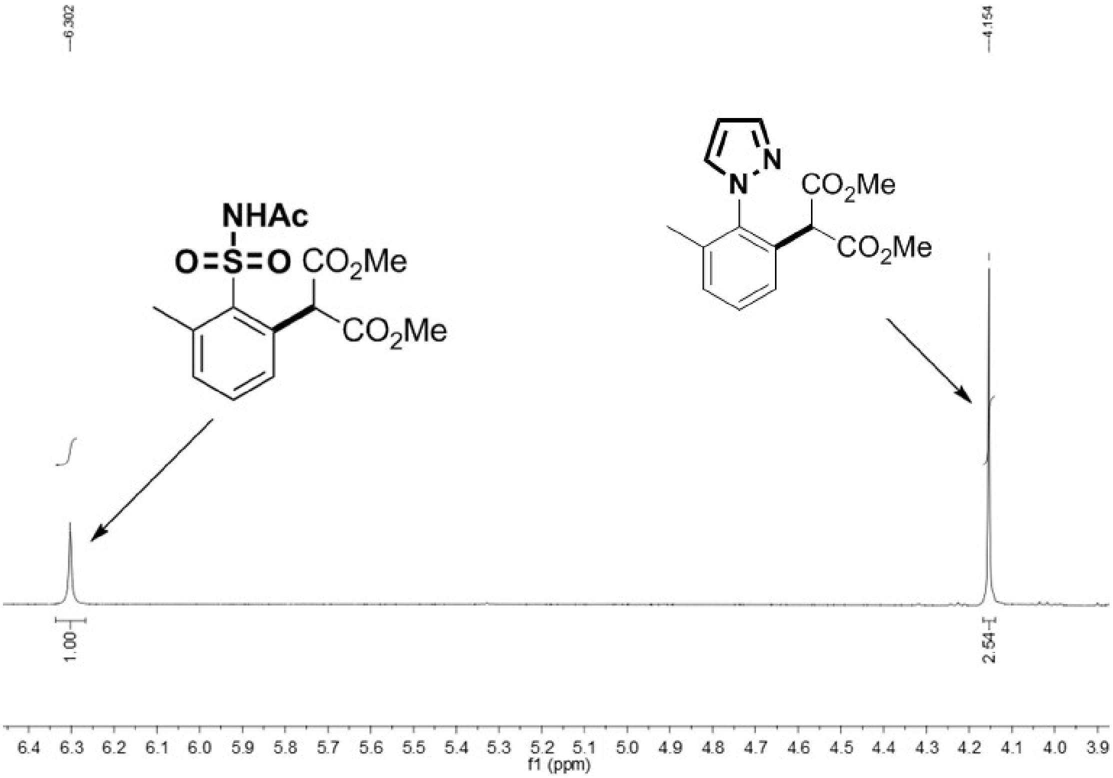

Intermolecular competition between sulfonamide and pyridine group by method A (Scheme 2, C)

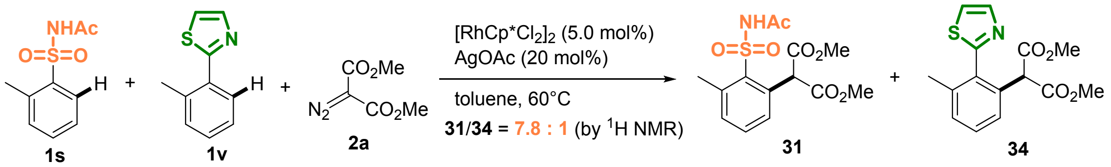

1s 1v 2a 31 34

A 50 mL tube equipped with a magnetic stir bar was charged with $[RhCp^*Cl_2]_2$ (5.0 mol%), AgOAc (20 mol%), **1s** (0.125 mmol), **1v** (0.125 mmol) and 25 mL toluene, then diazo compound **2a** (1.1 equivlent) was added. The tube was sealed, and the reaction mixture was stirred at 60°C over night. Toluene was removed under *vacuo*, and 20 mL DCM was added. The mixture was then filtered, the filtrate was concentrated, the ratio of **31** and **34** was determined by ^{1}H NMR analysis of the crude reaction mixture before separation and the residue was purified by preparative TLC on silica gel to afford **31** as a major product (34 mg, 79%).

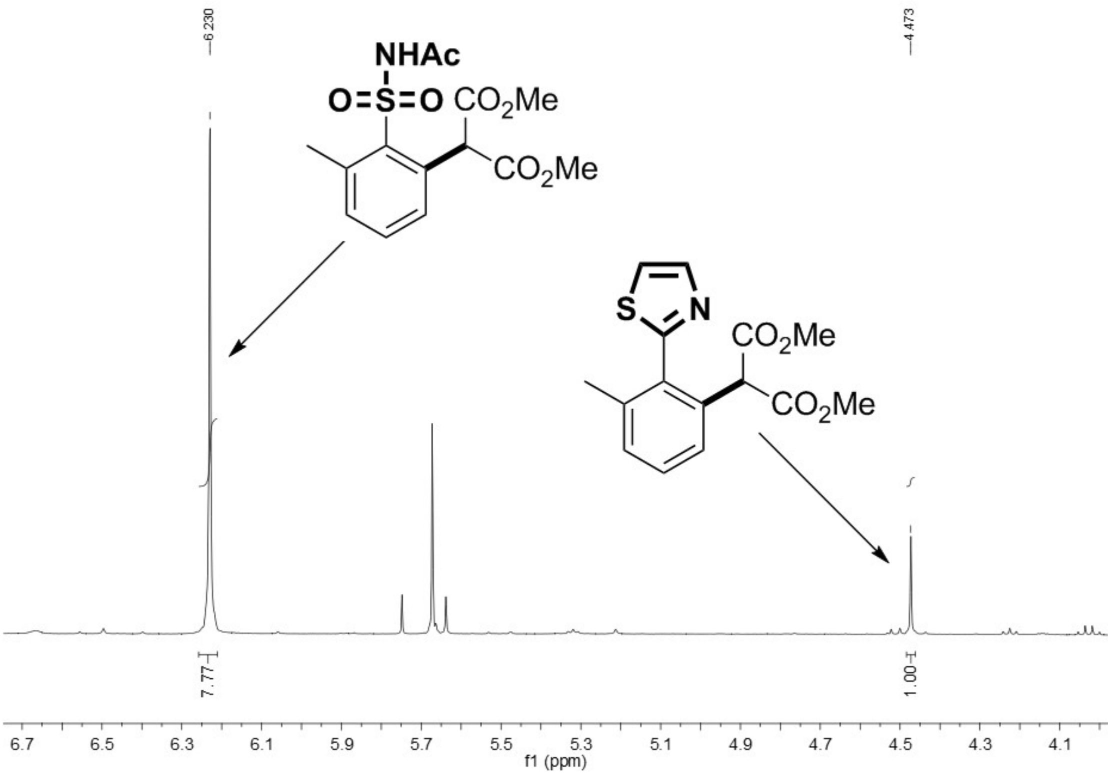

Intermolecular competition between sulfonamide and pyridine group by method B (Scheme 2, C)

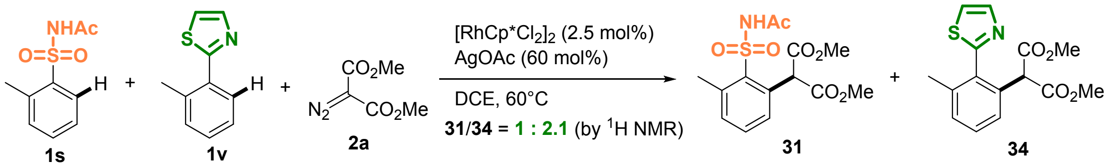

A 15 mL tube equipped with a magnetic stir bar was charged with $[RhCp^*Cl_2]_2$ (2.5 mol%), AgOAc (60 mol%), **1s** (0.125 mmol), **1v** (0.125 mmol) and 2.5 mL DCE, then diazo compound **2a** (1.1 equivlent) was added. The tube was sealed, and the reaction mixture was stirred at 60°C over night. DCE was removed under *vacuo*, and 20 mL DCM was added. The mixture was then filtered, the filtrate was concentrated, the ratio of **31** and **34** was determined by ^{1}H NMR analysis of the crude reaction mixture before separation and the residue was purified by preparative TLC on silica gel to afford **34** as a major product (22mg, 58%).

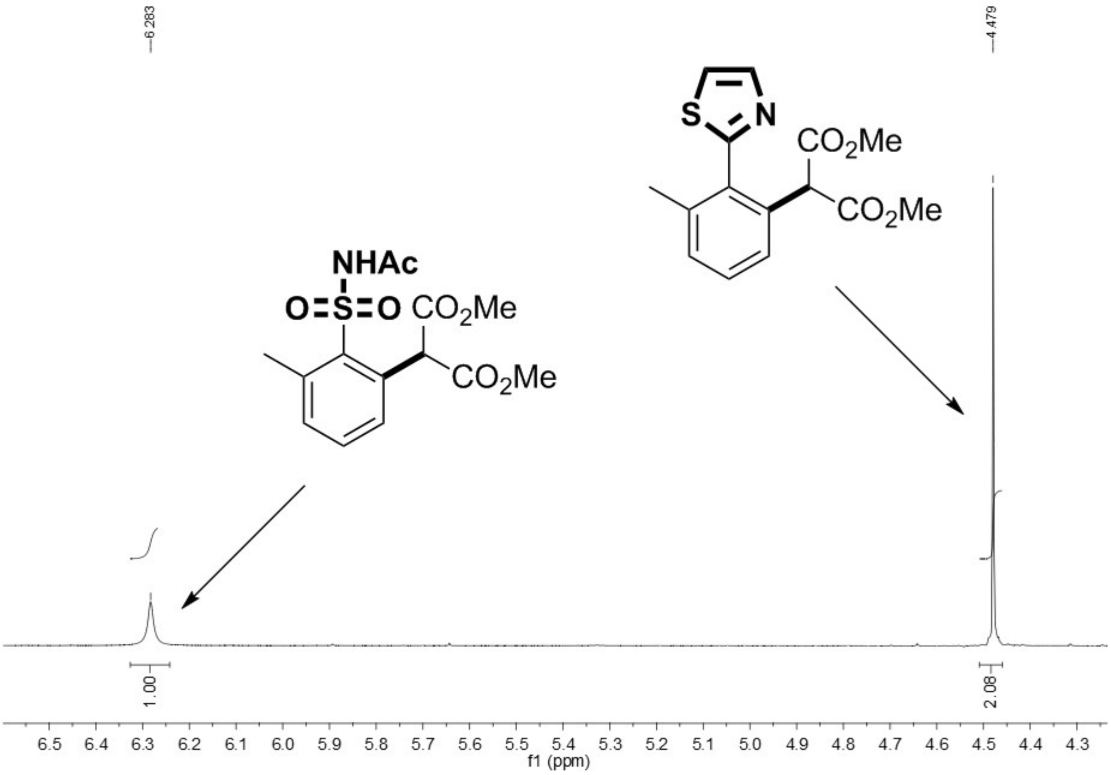

Intermolecular competition between sulfonamide and pyridine group by method A (Scheme 2, D)

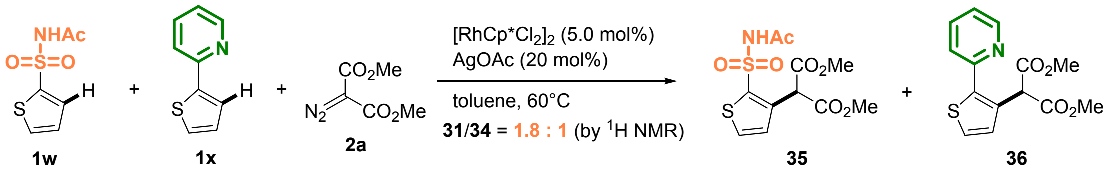

A 50 mL tube equipped with a magnetic stir bar was charged with [RhCp*Cl2]2 (5.0 mol%), AgOAc (20 mol%), **1w** (0.125 mmol), **1x** (0.125 mmol) and 25 mL toluene, then diazo compound **2a** (1.1 equivlent) was added. The tube was sealed, and the reaction mixture was stirred at 60°C over night. Toluene was removed under *vacuo*, and 20 mL DCM was added. The mixture was then filtered, the filtrate was concentrated, the ratio of **35** and **36** was determined by ^{1}H NMR analysis of the crude reaction mixture before separation and the residue was purified by preparative TLC on silica gel to afford **35** as a major product (23 mg, 55%).

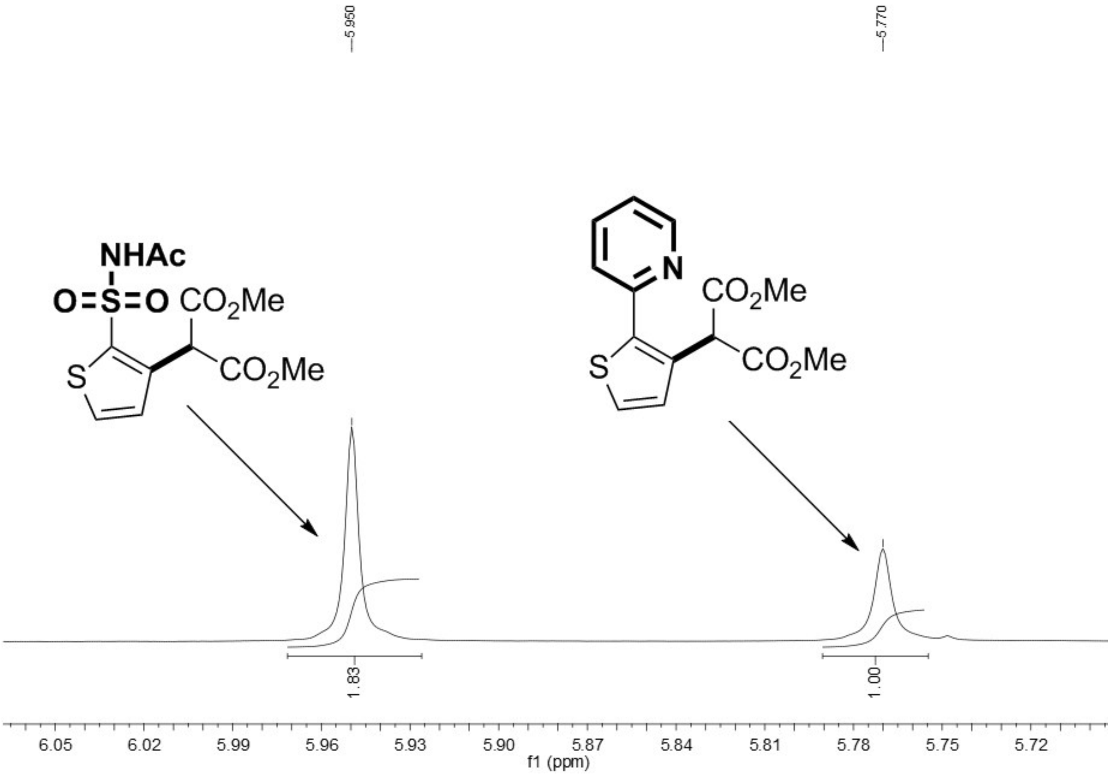

Intermolecular competition between sulfonamide and pyridine group by method B (Scheme 2, D)

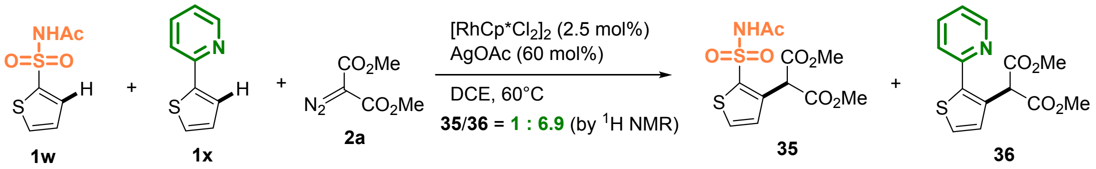

A 15 mL tube equipped with a magnetic stir bar was charged with $[RhCp^*Cl_2]_2$ (2.5 mol%), AgOAc (60 mol%), **1w** (0.125 mmol), **1x** (0.125 mmol) and 2.5 mL DCE, then diazo compound **2a** (1.1 equivlent) was added. The tube was sealed, and the reaction mixture was stirred at 60°C over night. DCE was removed under *vacuo*, and 20 mL DCM was added. The mixture was then filtered, the filtrate was concentrated, the ratio of **35** and **36** was determined by ^{1}H NMR analysis of the crude reaction mixture before separation and the residue was purified by preparative TLC on silica gel to afford **36** as a major product (27.4 mg, 75%).

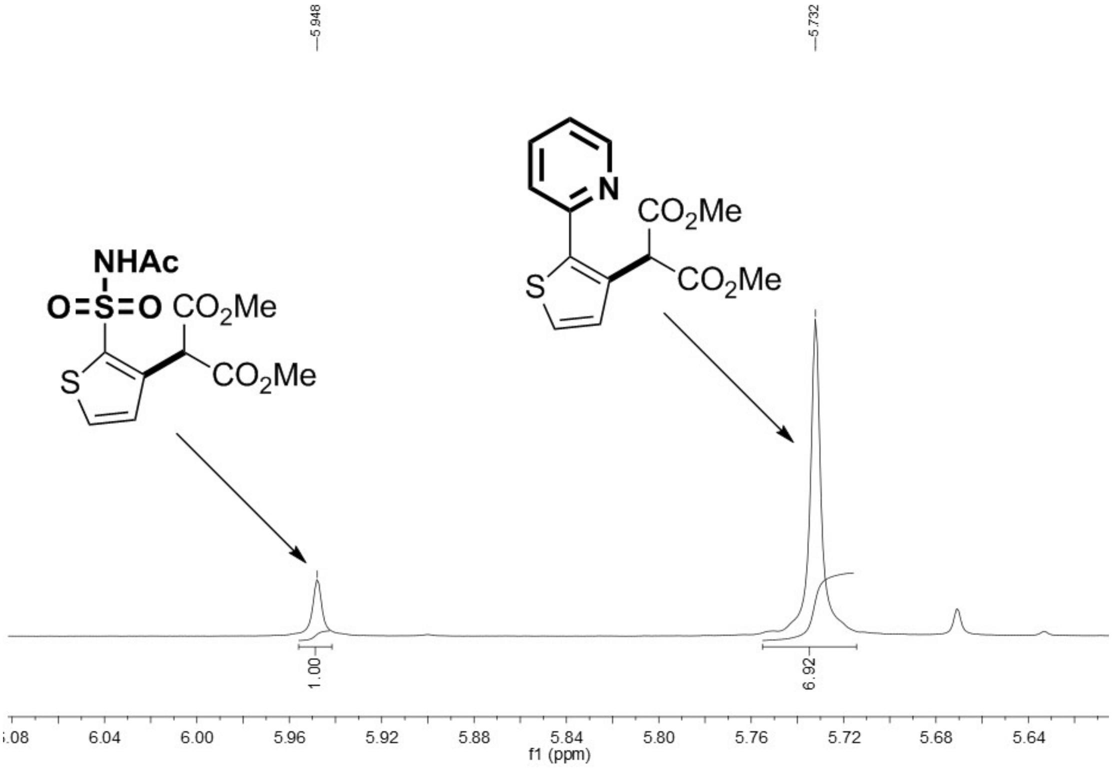

Isotope-labelling experiments

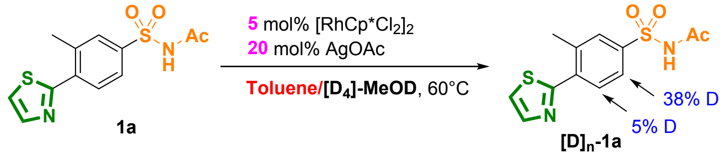

A 50 mL tube equipped with a magnetic stir bar was charged with $[RhCp^*Cl_2]_2$ (5.0 mol%), AgOAc (20 mol%), **1a** (0.125 mmol) and 25 mL toluene, then $[D_4]$-MeOH (0.5mL) was added. The tube was sealed, and the reaction mixture was stirred at 60°C over night. Toluene was removed under *vacuo*, and 20 mL DCM was added. The mixture was then filtered, the filtrate was concentrated, and the residue was purified by preparative TLC on silica gel to afford **$[D]_4$-1a**.

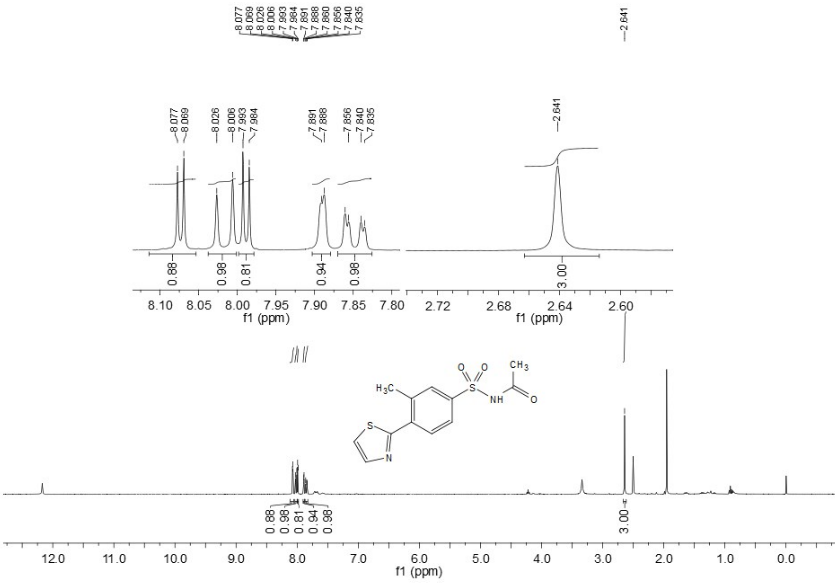

Scheme 3, A

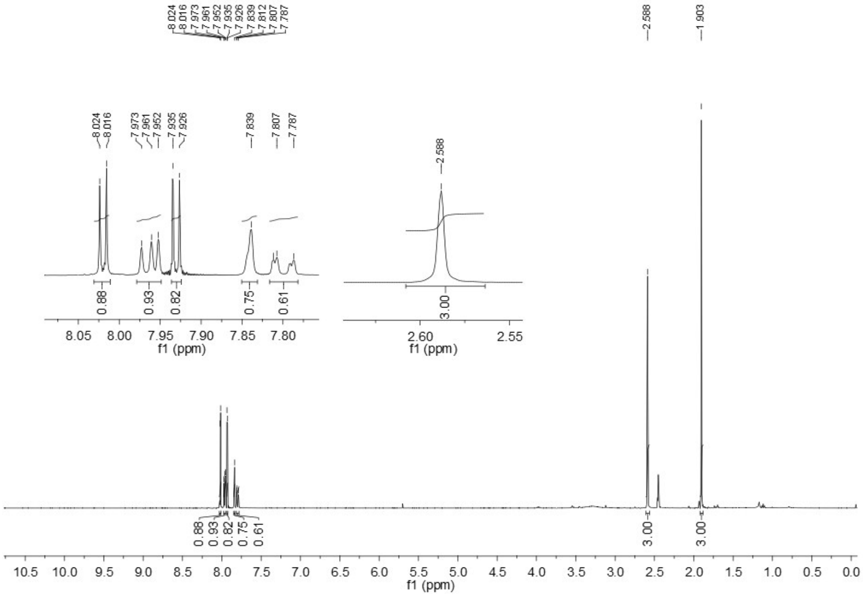

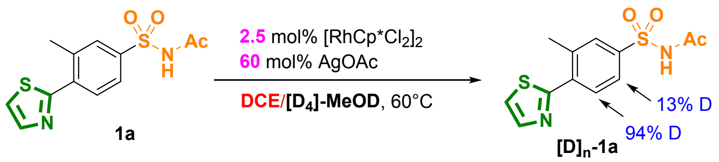

A 15 mL tube equipped with a magnetic stir bar was charged with [RhCp*Cl_2]$_2$ (2.5 mol%), AgOAc (60 mol%), **1a** (0.125 mmol) and 2.5 mL DCE, then [D_4]-MeOH (0.5mL) was added. The tube was sealed, and the reaction mixture was stirred at 60°C over night. DCE was removed under *vacuo*, and 20 mL DCM was added. The mixture was then filtered, the filtrate was concentrated, and the residue was purified by preparative TLC on silica gel to afford **[D]$_4$-1a**.

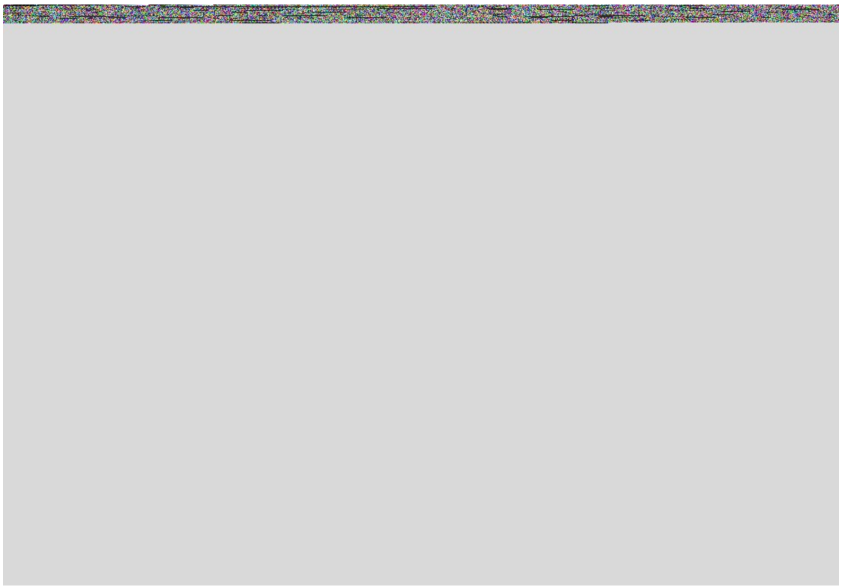

Scheme 3, B

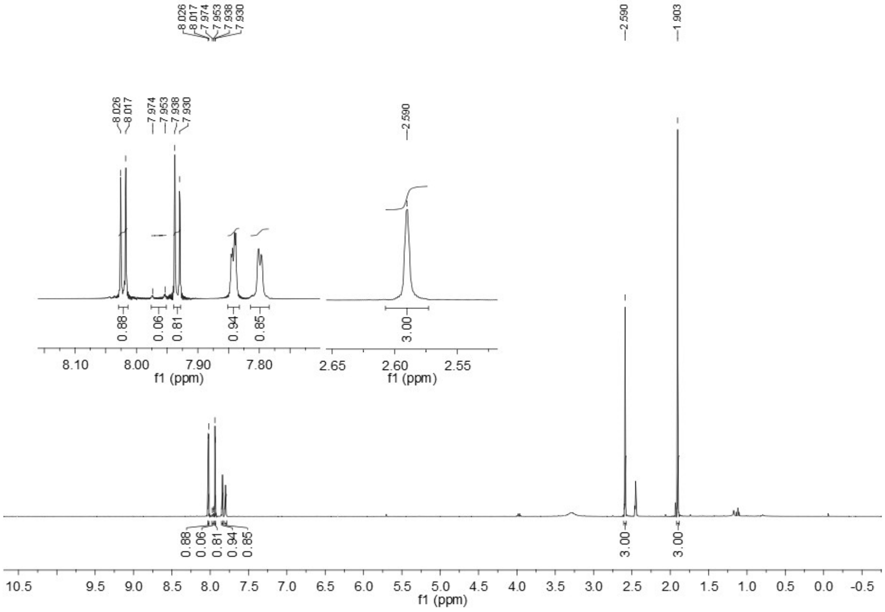

Stoichiometric C–H rhodation experiments

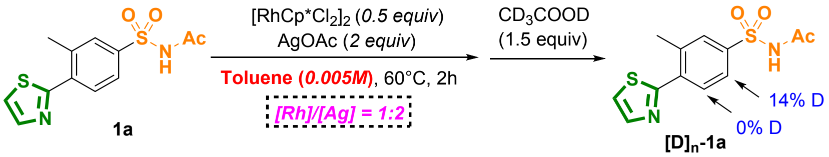

3-methyl-4-thiazole-*N*-acetyl sulfonamide (**1a**) (0.125 mmol), [Cp*$RhCl_2$]$_2$ (0.5 equiv), AgOAc (2.0 equiv) and toluene (25 mL) were placed in a 50 mL sealed tube and the mixture was stirred at 60 °C for 3 h. CD3COOD (9.0 µl, 1.5 equiv) was then added and the reaction was stirred for 5 minutes. After cooling to ambient temperature, toluene was removed under *vacuo*. The reaction mixture was purified by flash column chromatography on neutralized silica gel (*n*-hexane/EtOAc: 2/1) to afford the product [D]n-**1a**.

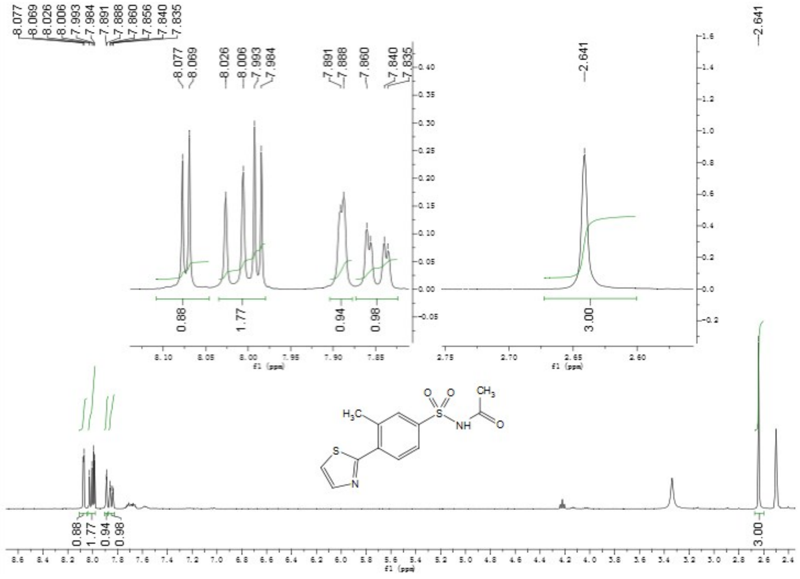

Scheme 4A, ^{1}H NMR of compound **[D]$_n$-1a**

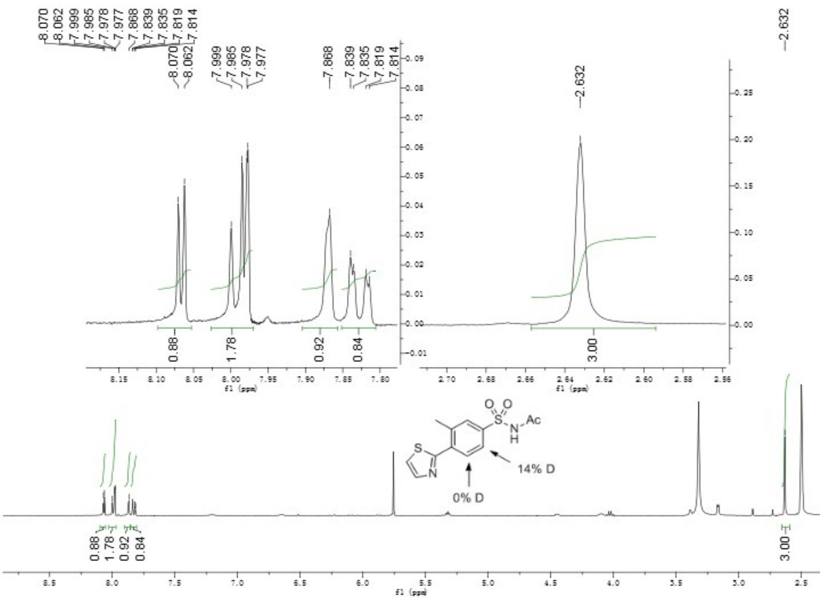

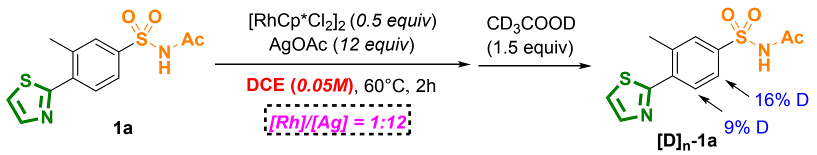

3-methyl-4-thiazole-*N*-acetyl sulfonamide (**1a**) (0.125 mmol), $[Cp^*RhCl_2]_2$ (0.5 equiv), AgOAc (12 equiv) and DCE (2.5 mL) were placed in a 10 mL sealed tube and the mixture was stirred at 60 °C for 3 h. CD3COOD (9.0 µl, 1.5 equiv) was then added and the reaction was stirred for 5 minutes. After cooling to ambient temperature, DCE was removed under *vacuo*. The reaction mixture was purified by flash column chromatography on neutralized silica gel (*n*-hexane/EtOAc: 2/1) to afford the product [D]n-**1a**.

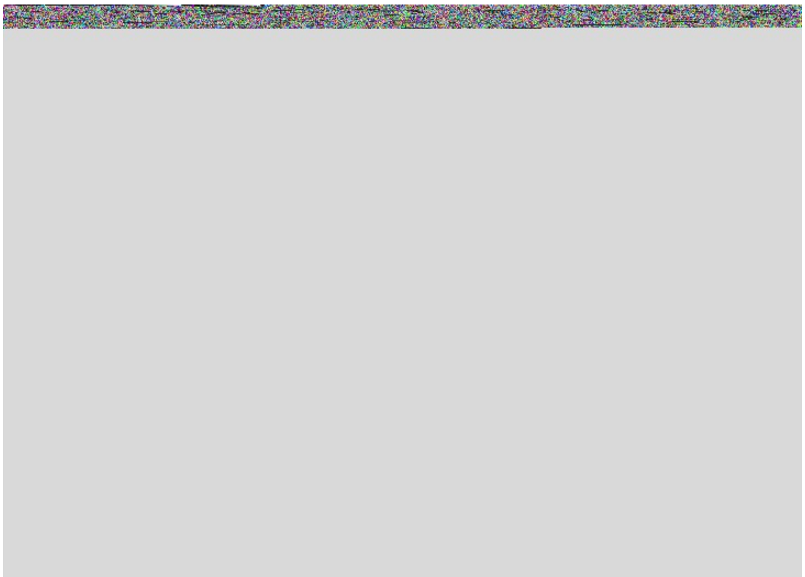

Scheme 4B, ^{1}H NMR of compound **$[D]_n$-1a**

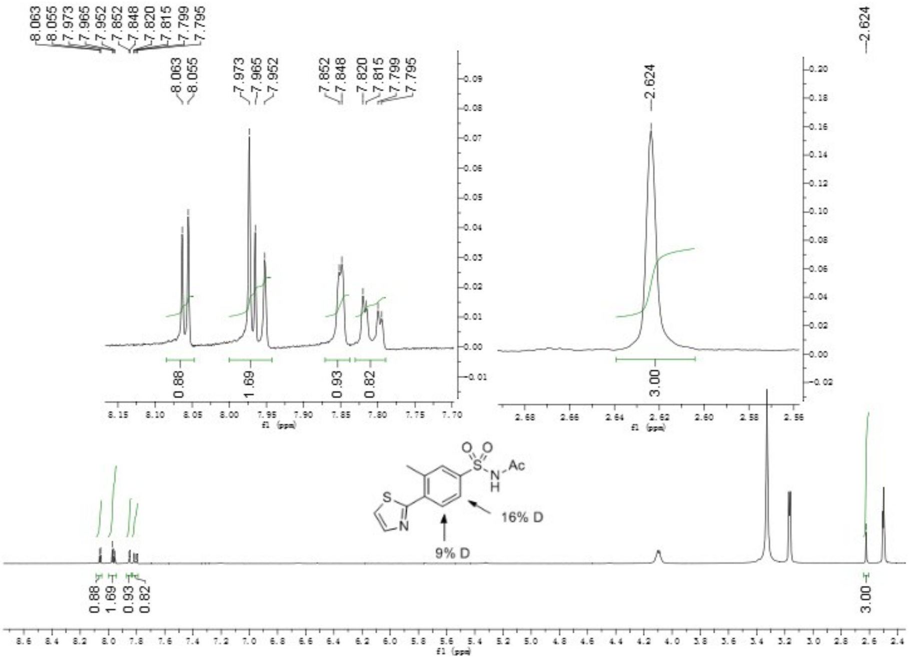

kinetic isotope effect (KIE) experiments

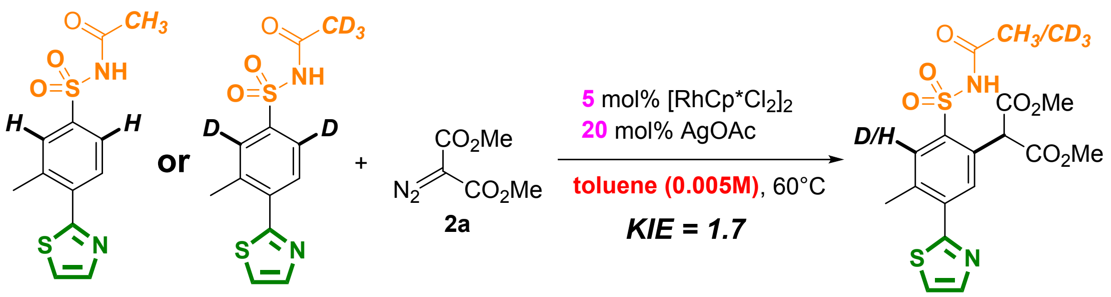

1a **[D$_4$]-1a**

Two parallel reactions with **1a** and **[D4]-1a** proceeded under Method A conditions: A suspension of [RhCp*Cl$_2$]$_2$ (5%), AgOAc (20%), **1a** (0.125 mmol) or **[D4]-1a** (0.125 mmol), diazo compound (1.1 euqiv) and 25 mL toluene was stirred at 60°C for 2h, then cooled to room temperature. The two reaction mixtures were combined and filtered through a short plug of silica gel. Solvent was removed under *vacuo* and the residue was purified by preparative TLC on silica gel to afford desired compound.

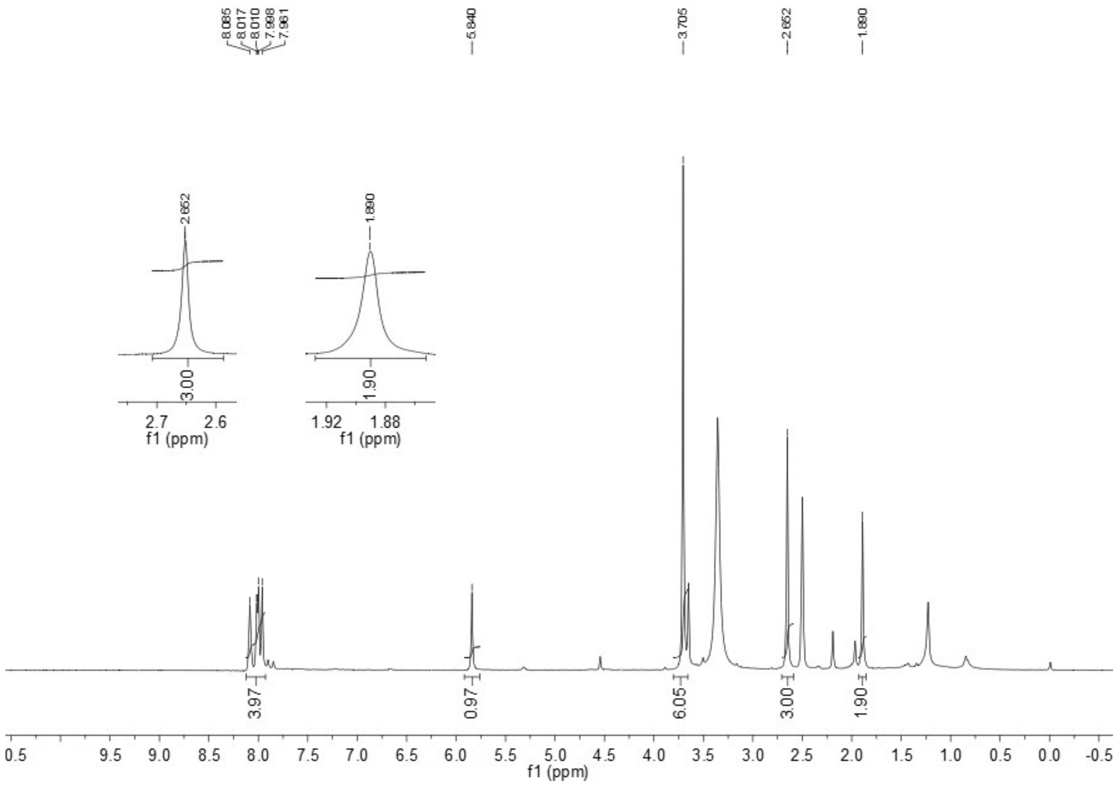

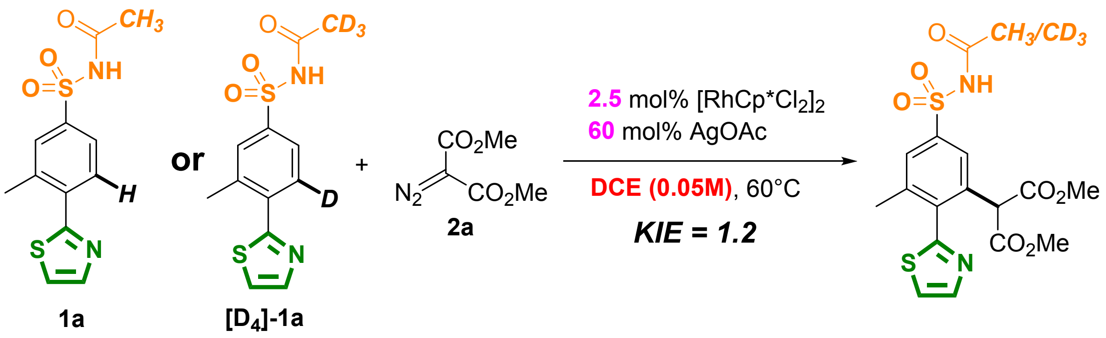

Two parallel reactions with **1a** and **[D4]-1a** proceeded under Method B conditions: A suspension of [RhCp*Cl2]2 (2.5%), AgOAc (60%), **1a** (0.125 mmol) or **[D4]-1a** (0.125 mmol), diazo compound (1.1 euqiv) and 2.5 mL DCE was stirred at 60°C for 20min, then cooled to room temperature. The two reaction mixtures were combined and filtered through a short plug of silica gel. Solvent was removed under *vacuo* and the residue was purified by preparative TLC on silica gel to afford desired compound.

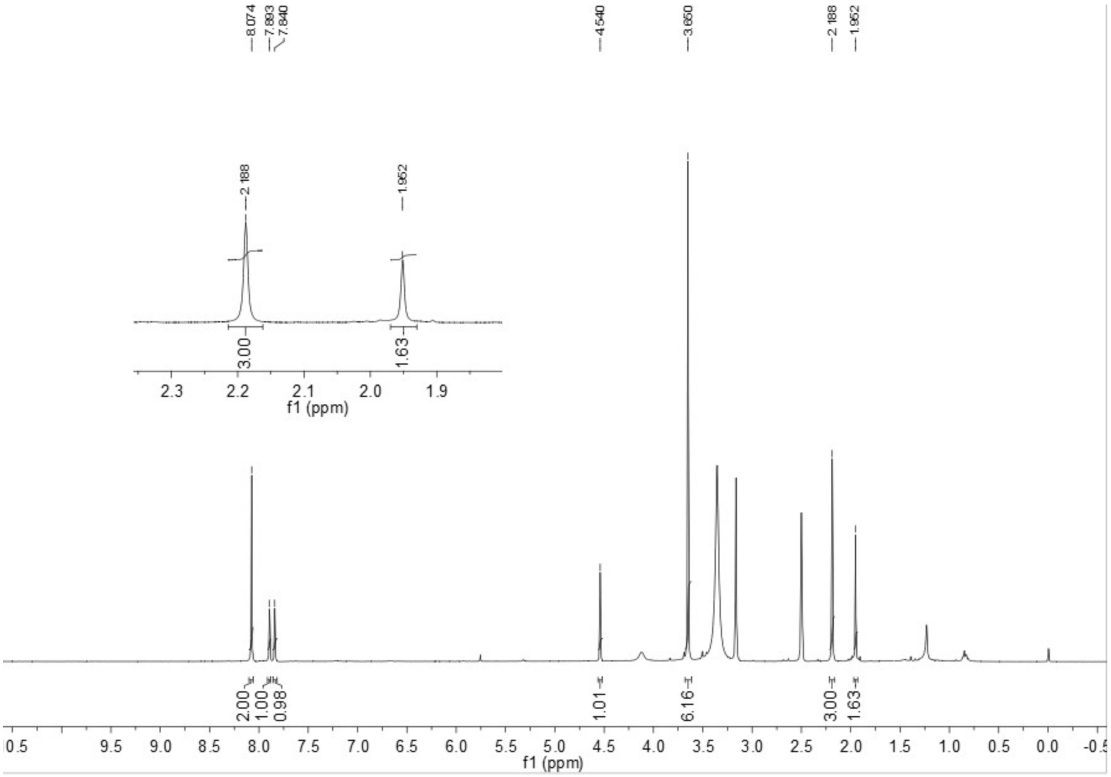

Synthetic procedure

Stille reaction:

$Pd(PPh_3)_2Cl_2$ (5 mol%), aryl bromide (5 mmol) and heterocycle stannyl reagent (7.5 mmol) were dissolved in dry DMF (10 mL), and the mixture was stirred at 90℃ under nitrogen atmosphere overnight. Then solvent was removed and 100mL water was added, the aqueous phase was extracted by EtOAc (4 X 100mL). Organic layer was combined and washed with saturated NaCl solution, dried over anhydrous Na_2SO_4, concentrated in *vacuo*, and the residue was purified by silica gel chromatography to afford the desired compounds. (This method was applied for thiazole, pyridine, pyrimidine and pyrazine derivatives)

Suzuki reaction:

$Pd(PPh_3)_4$ (5 mol%), aryl bromide (5 mmol) and quinolin-8-ylboronic acid (10 mmol) were dissolved in a mixed solvent (toluene/EtOH/H_2O = 2:1:1) (60 mL), and the mixture was stirred at 100℃ under nitrogen atmosphere for 6h. Then solvent was removed and 100mL water was added, the aqueous phase was extracted by EtOAc (4 X 100mL). Organic layer was combined and washed with saturated NaCl solution, dried over anhydrous Na_2SO_4, concentrated in *vacuo*, and the residue was purified by silica gel chromatography to afford the desired compounds. (This method was applied for quinolone derivatives)

Synthesis of Pyrazole derivatives:

Cu_2O (15 mmol), Cs_2CO_3 (15 mol), aryl bromide (5 mmol) and Pyrazole (10 mmol) were dissolved in dry DMF (10 mL), and the mixture was stirred at 100℃ under nitrogen atmosphere overnight. Then solvent was removed and 100mL water was added, the aqueous phase was extracted by EtOAc (4 X 100mL). Organic layer was combined and washed with saturated NaCl solution, dried over anhydrous Na_2SO_4, concentrated in *vacuo*, and the residue was purified by silica gel chromatography to afford the desired compounds.

Acetylation of sulfonamide derivatives:

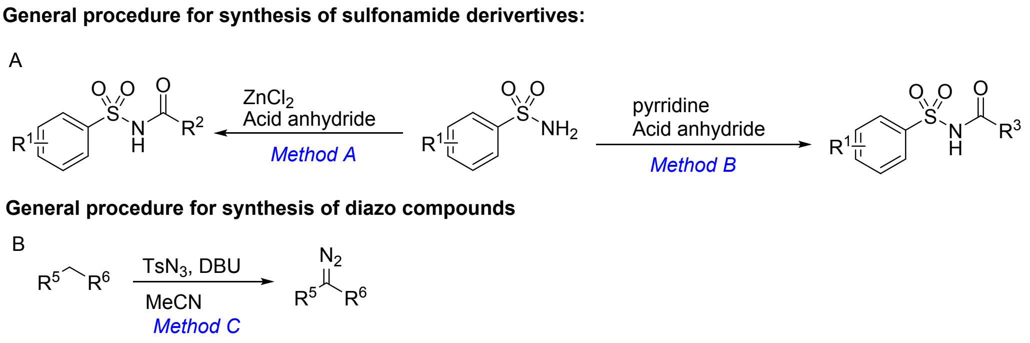

Figure S1. General procedure for synthesis of sulfonamide derivertives and diazo compounds

Method A:

Sulfonamide (5 mmol) was dissolved in 5 mL acid anhydride, 0.1eq~1eq anhydrous $ZnCl_2$ was added, the reaction mixture was stirred at room temperature and monitored by TLC until the free sulfonamide was consumed completely, then poured into a mixture of EtOAc and water (100mL, *v*/*v* = 1:1). The organic layer was separated and the aqueous phase was extracted by EtOAc (50 mL). The organic layers were combined and washed with saturated NaCl solution, dried over anhydrous Na_2SO_4, concentrated in *vacuo* to afford solid powder and washed with cold toluene to give the acetyl or propionyl sulfonamide without further purification, the purity was detected by ^{1}HNMR.

Method B:

Sulfonamide (5 mmol) and DMAP (61 mg, 0.5 mmol) were dissolved in 5 mL pyridine, then Ac_2O (4.7 mL, 50 mmol, 10 equiv) was added. The reaction mixture was stirred at room temperature overnight, and concentrated. The residue was dissolved in EtOAc (50 mL) and washed with saturated NH_4Cl (50 mL). The organic layer was dried over Na_2SO_4, concentrated again in *vacu*o, the residue was purified by silica gel chromatography.

Method C:

1,3-dicarbonyl compound (50 mmol) and tosyl azide (55 mmol) were dissolved in acetonitrile (100 mL), the mixture was cooled to 0°C. DBU (55 mmol) was added dropwise, and the reaction mixture was stirred for 3h. Solvent was removed and the residue was dissolved in DCM, washed with water, the aqueous layer was extracted by DCM, and the organic layers were combined and washed with brine, and dried over Na_2SO_4, and concentrated in *vacuo*. The residue was purified by silica gel chromatography.

<u>*N*-((3-methyl-4-(thiazol-2-yl)phenyl)sulfonyl)acetamide (1a)</u>

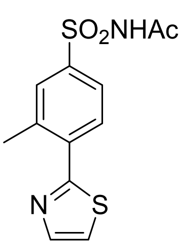

Method B, (87%, white powder), R_f = 0.5 (EtOAc/Petroleum ether = 1:1). **m.p.**: 170-171°C. **^{1}H NMR** (400 MHz, DMSO-*d6*) δ 12.18 (s, 1H), 8.07 (t, *J* = 3.4 Hz, 1H), 8.01 (d, *J* = 8.2 Hz, 1H), 7.98 (d, *J* = 3.3 Hz, 1H), 7.89 (d, *J* = 1.3 Hz, 1H), 7.85 (dd, *J* = 8.2, 1.7 Hz, 1H), 2.64 (s, 3H), 1.95 (s, 3H). **^{13}C NMR** (101 MHz, DMSO-*d6*) δ 168.9, 164.8, 143.7, 139.5, 137.0, 136.8, 130.3, 130.2, 125.5, 122.4, 23.3, 21.4. **HRMS** (ESI): m/z (M + H$^+$) calcd for $C_{12}H_{13}O_3N_2S_2$, 297.0362, found: 297.0355.

***N*-((2-methyl-4-(thiazol-2-yl)phenyl)sulfonyl)acetamide (1b)**

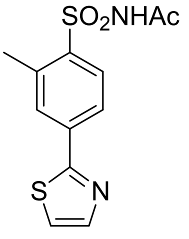

Method B, (85%, white powder), R_f = 0.5 (EtOAc/Petroleum ether = 1:1). **m.p.**: 162-163°C. **^{1}H NMR** (400 MHz, DMSO-*d6*) δ 12.29 (s, 1H), 8.07 – 8.04 (m, 1H), 8.02 (d, *J* = 3.2 Hz, 1H), 8.00 – 7.96 (m, 2H), 7.92 (d, *J* = 3.2 Hz, 1H), 2.66 (s, 3H), 1.96 (s, 3H). **^{13}C NMR** (101 MHz, DMSO-*d6*) δ 168.8, 165.1, 144.4, 138.2, 138.1, 137.1, 131.5, 129.6, 123.7, 122.3, 23.2, 19.5. **HRMS** (ESI): m/z (M + H$^+$) calcd for $C_{12}H_{13}O_3N_2S_2$, 297.0362, found: 297.0357

***N*-((4-(thiazol-2-yl)-2-(trifluoromethoxy)phenyl)sulfonyl)acetamide (1c)**

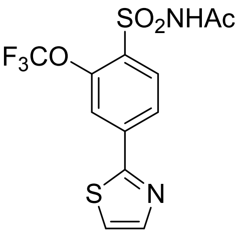

Method B, (88%, white powder), R_f = 0.3 (EtOAc/Petroleum ether = 1:1). **m.p.**: 191-193°C. **^{1}H NMR** (400 MHz, DMSO-d6) δ 12.61 (s, 1H), 8.18 (d, *J* = 8.3 Hz, 1H), 8.13 (dd, *J* = 8.3, 1.5 Hz, 1H), 8.08 – 8.05 (m, 2H), 8.02 (d, *J* = 3.2 Hz, 1H), 1.97 (s, 3H). **^{13}C NMR** (101 MHz, DMSO-d6) δ 169.0, 163.4, 145.5, 144.7, 139.4, 133.6, 131.7, 125.0, 123.5, 119.9 (q, *J* = 261.0 Hz), 117.3, 23.0. **HRMS** (ESI): m/z (M + H$^+$) calcd for $C_{12}H_{10}O_4N_2F_3S_2$, 367.0029, found: 367.0018.

***N*-((4-(thiazol-2-yl)naphthalen-1-yl)sulfonyl)acetamide (1d)**

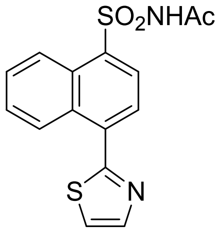

Method B, (82%, white powder), R_f = 0.5 (EtOAc/Petroleum ether = 1:1). **m.p.**: 214-216°C. **^{1}H NMR** (400 MHz, DMSO-*d6*) δ 12.58 (s, 1H), 8.88 (dd, *J* = 8.4, 0.5 Hz, 1H), 8.70 (d, *J* = 8.4 Hz, 1H), 8.40 (d, *J* = 7.8 Hz, 1H), 8.18 (d, *J* = 3.3 Hz, 1H), 8.07 (d, *J* = 2.8 Hz, 1H), 8.06 (d, *J* = 1.6 Hz, 1H), 7.85 (ddd, *J* = 8.5, 6.9, 1.3 Hz, 1H), 7.78 (ddd, *J* = 8.2, 6.9, 1.2 Hz, 1H), 1.92 (s, 3H). **^{13}C NMR** (101 MHz, DMSO-*d6*) δ 168.8, 164.9, 144.2, 136.0, 135.2, 130.5 (X2), 128.7, 128.1, 128.0, 127.0, 126.9, 124.0, 122.8, 23.2. **HRMS** (ESI): m/z (M + H$^+$) calcd for $C_{15}H_{13}O_3N_2S_2$, 333.0362, found: 333.0353.

***N*-((2-methyl-4-(1H-pyrazol-1-yl)phenyl)sulfonyl)acetamide (1e)**

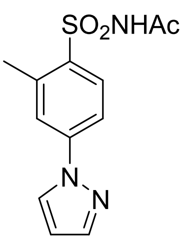

Method B, (79%, white powder), R_f = 0.3 (EtOAc/Petroleum ether = 1:1). **m.p.**: 133-135°C. **^{1}H NMR** (400 MHz, DMSO-*d6*) δ 12.22 (s, 1H), 8.62 (d, *J* = 2.6 Hz, 1H), 8.04 (d, *J* = 8.7 Hz, 1H), 7.94 (d, *J* = 2.1 Hz, 1H), 7.89 (dd, *J* = 8.7, 2.3 Hz, 1H), 7.83 (d, *J* = 1.7 Hz, 1H), 6.61 (dd, *J* = 2.5, 1.8 Hz, 1H), 2.65 (s, 3H), 1.95 (s, 3H). **^{13}C NMR** (101 MHz, DMSO-*d6*) δ 168.7, 142.7, 142.2, 139.1, 134.3, 132.3, 128.4, 121.2, 115.2, 108.8, 23.2, 19.7. **HRMS** (ESI): m/z (M + H$^+$) calcd for $C_{12}H_{14}O_3N_3S$, 280.0750, found: 280.0744.

N-((4-(3,5-dimethyl-1H-pyrazol-1-yl)-3-methylphenyl)sulfonyl)acetamide (1f)

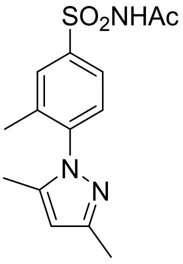

Method B, (82%, white powder), ***R*f** = 0.4 (EtOAc/Petroleum ether = 1:1). **m.p.**: 220-222°C. **^{1}H NMR** (400 MHz, DMSO-*d6*) δ 12.42 (s, 1H), 7.84 (s, 1H), 7.77 (d, *J* = 8.2 Hz, 1H), 7.40 (d, *J* = 8.2 Hz, 1H), 6.05 (s, 1H), 2.16 (s, 3H), 2.06 (s, 3H), 2.03 (s, 3H), 1.87 (s, 3H). **^{13}C NMR** (101 MHz, DMSO-*d6*) δ 171.6, 147.9, 142.0, 141.3, 140.0, 136.1, 129.4, 127.9, 125.5, 105.6, 24.6, 17.2, 13.4, 11.0. **HRMS** (ESI): m/z (M + H$^+$) calcd for $C_{14}H_{18}O_3N_3S$, 308.1063, found: 308.1053.

N-((3-methyl-4-(1H-pyrazol-1-yl)phenyl)sulfonyl)acetamide (1g)

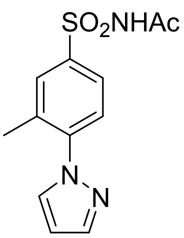

Method B, (75%, light yellow powder), ***R*f** = 0.3 (EtOAc/Petroleum ether = 1:1). **m.p.**: 148-150°C. **^{1}H NMR** (400 MHz, DMSO-*d6*) δ 12.21 (s, 1H), 8.20 (d, *J* = 2.4 Hz, 1H), 7.92 (d, *J* = 1.8 Hz, 1H), 7.86 (dd, *J* = 8.4, 2.1 Hz, 1H), 7.80 (d, *J* = 1.6 Hz, 1H), 7.63 (d, *J* = 8.4 Hz, 1H), 6.57 – 6.55 (m, 1H), 2.36 (s, 3H), 1.95 (s, 3H). **^{13}C NMR** (101 MHz, DMSO-*d6*) δ 168.9, 143.2, 141.1, 138.1, 133.2, 131.6, 130.5, 126.2, 126.1, 107.2, 23.3, 18.5. **HRMS** (ESI): m/z (M + H$^+$) calcd for $C_{12}H_{14}O_3N_3S$, 280.0750, found: 280.0745.

N-((4,5-diphenylpyrazolo[1,5-a]quinolin-7-yl)sulfonyl)acetamide (1h)

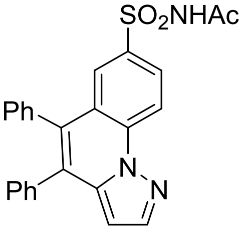

Method B, (85%, white powder), ***R*f** = 0.5 (EtOAc/Petroleum ether = 1:1). **m.p.**: 237-239°C. **^{1}H NMR** (400 MHz, DMSO-*d6*) δ 12.54 (s, 1H), 8.71 (d, *J* = 8.8 Hz, 1H), 8.21 (d, *J* = 1.8 Hz, 1H), 7.92 (d, *J* = 1.8 Hz, 1H), 7.39 – 7.24 (m, 10H), 6.86 (d, *J* = 5.1 Hz, 1H), 6.35 (d, *J* = 2.0 Hz, 1H), 1.77 (s, 3H). **^{13}C NMR** (101 MHz, DMSO-*d6*) δ 171.1, 142.7, 141.9, 139.1, 138.3, 136.1, 135.5, 134.8, 133.3, 130.8, 129.9, 129.7, 128.2, 128.1, 127.7, 127.2, 122.9, 115.4, 106.9, 101.6, 24.5. **HRMS** (ESI): m/z (M + H$^+$) calcd for $C_{25}H_{20}O_3N_3S$, 442.1220, found: 442.1206.

N-((3-methyl-4-(1-methyl-1H-benzo[d]imidazol-2-yl)phenyl)sulfonyl)acetamide (1i)

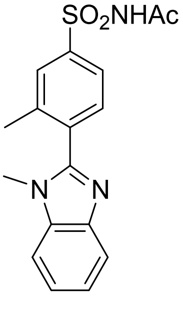

Method B, (80%, light yellow powder), ***R*f** = 0.4 (EtOAc/Petroleum ether = 1:1). **m.p.**: 203 – 205°C. **^{1}H NMR** (400 MHz, DMSO-*d6*) δ 12.23 (s, 1H), 7.95 (d, *J* = 1.4 Hz, 1H), 7.89 (dd, *J* = 8.0, 1.8 Hz, 1H), 7.74 (d, *J* = 8.1 Hz, 1H), 7.71 (d, *J* = 7.9 Hz, 1H), 7.65 (d, *J* = 7.8 Hz, 1H), 7.37 – 7.31 (m, 1H), 7.31 – 7.26 (m, 1H), 3.65 (s, 3H), 2.33 (s, 3H). **^{13}C NMR** (101 MHz, DMSO-*d6*) δ 169.0, 151.2, 142.3, 140.3, 139.2, 135.5, 134.9, 131.2, 129.0, 124.8, 122.6, 122.1, 119.1, 110.7, 30.8, 23.3, 19.5. **HRMS** (ESI): m/z (M + H$^+$) calcd for $C_{17}H_{18}O_3N_3S$, 344.1063, found: 344.1064.

N-((3-methyl-4-(pyridin-2-yl)phenyl)sulfonyl)acetamide (1j)

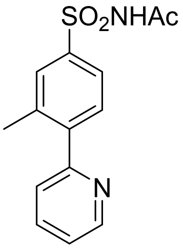

Method B, (80%, white powder), ***R*f** = 0.3 (EtOAc/Petroleum ether = 1:1). **m.p.**: 188 – 190°C.**^{1}H NMR** (400 MHz, DMSO-*d6*) δ 12.17 (s, 1H), 8.70 (ddd, *J* = 4.8, 1.7, 0.9 Hz, 1H), 7.93 (td, *J* = 7.7, 1.8 Hz, 1H), 7.84 – 7.80 (m, 2H), 7.60 (ddd, *J* = 7.8, 2.3, 1.2 Hz, 2H), 7.43 (ddd, *J* = 7.6, 4.9, 1.1 Hz, 1H), 2.39 (s, 3H), 1.94 (s, 3H). **^{13}C NMR**

(101 MHz, DMSO-*d6*) δ 169.1, 157.4, 149.3, 144.6, 139.1, 136.9, 136.6, 130.3, 129.2, 125.0, 124.1, 122.8, 23.4, 20.1. **HRMS** (ESI): m/z (M + H^+) calcd for $C_{14}H_{15}O_3N_2S$, 291.0798, found: 291.0794.

***N-((3-methyl-4-(pyrimidin-2-yl)phenyl)sulfonyl)acetamide* (1k)**

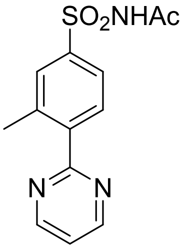

Method B, (70%, white powder), R_f = 0.3 (EtOAc/Petroleum ether = 1:1). **m.p.**: 177-179°C. **^{1}H NMR** (400 MHz, DMSO-*d6*) δ 12.15 (s, 1H), 8.98 (d, *J* = 4.9 Hz, 2H), 7.99 – 7.94 (m, 1H), 7.89 – 7.85 (m, 2H), 7.54 (t, *J* = 4.9 Hz, 1H), 2.55 (s, 3H), 1.96 (s, 3H). **^{13}C NMR** (101 MHz, DMSO-*d6*) δ 168.9, 165.0, 157.5, 142.5, 139.6, 137.9, 131.0, 129.7, 125.0, 120.0, 23.3, 20.8. **HRMS** (ESI): m/z (M + H^+) calcd for $C_{13}H_{14}O_3N_3S$, 292.0750, found: 292.0738.

***N*-((2-methyl-4-(pyrazin-2-yl)phenyl)sulfonyl)acetamide (1l)**

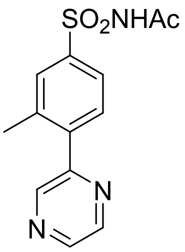

Method B, (77%, white powder), R_f = 0.4 (EtOAc/Petroleum ether = 1:1). **m.p.**: 228-230°C. **^{1}H NMR** (400 MHz, DMSO-*d6*) δ 12.16 (s, 1H), 8.91 (d, J = 1.5 Hz, 1H), 8.79 (dd, J = 2.5, 1.6 Hz, 1H), 8.71 (d, J = 2.5 Hz, 1H), 7.90 – 7.84 (m, 2H), 7.72 (d, J = 8.0 Hz, 1H), 2.43 (s, 3H), 1.96 (s, 3H). **^{13}C NMR** (101 MHz, DMSO-*d6*) δ 168.9, 153.0, 144.9, 144.1, 143.8, 141.4, 139.6, 137.4, 130.7, 129.4, 125.2, 23.3, 20.0. **HRMS** (ESI): m/z (M + H^+) calcd for $C_{13}H_{14}O_3N_3S$, 292.0750, found: 292.0743.

***N*-((5-(pyridin-2-yl)thiophen-2-yl)sulfonyl)acetamide (1m)**

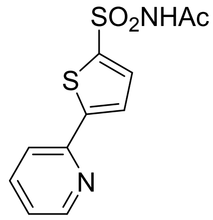

Method B, (81%, white powder), R_f = 0.3 (EtOAc/Petroleum ether = 1:2). **m.p.**: 211 – 213°C. **^{1}H NMR** (400 MHz, DMSO-*d6*) δ 12.51 (s, 1H), 8.58 (d, *J* = 4.4 Hz, 1H), 8.03 (d, *J* = 8.0 Hz, 1H), 7.90 (td, *J* = 7.8, 1.6 Hz, 1H), 7.80 (d, *J* = 4.0 Hz, 1H), 7.70 (d, *J* = 4.0 Hz, 1H), 7.38 (dd, *J* = 6.9, 5.0 Hz, 1H), 1.92 (s, 3H). **^{13}C NMR** (101 MHz, DMSO-*d6*) δ 170.0, 150.7, 150.4, 149.7, 142.0, 137.5, 134.0, 124.4, 123.8, 119.4, 23.9. **HRMS** (ESI): m/z (M + H^+) calcd for $C_{11}H_{11}O_3N_2S_2$, 283.0206, found: 283.0199.

***N*-((5-(2-methylthiazol-4-yl)thiophen-2-yl)sulfonyl)acetamide (1n)**

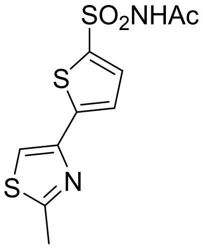

Method B, (87%, white powder), R_f = 0.3 (EtOAc/Petroleum ether = 1:2). **m.p.**: 236-238°C. **^{1}H NMR** (400 MHz, DMSO-*d6*) δ 12.30 (s, 1H), 8.11 (s, 1H), 7.74 (d, *J* = 4.0 Hz, 1H), 7.60 (d, *J* = 4.0 Hz, 1H), 2.70 (s, 3H), 1.97 (s, 3H). **^{13}C NMR** (101 MHz, DMSO-*d6*) δ 168.9, 166.9, 146.4, 145.3, 137.6, 135.0, 123.3, 116.1, 23.3, 18.7. **HRMS** (ESI): m/z (M + H^+) calcd for $C_{10}H_{11}O_3N_2S_3$, 302.9926, found: 302.9919.

***N*-((4-(thiazol-2-yl)phenyl)sulfonyl)acetamide (1o)**

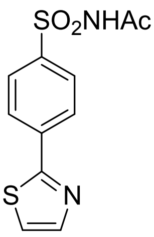

Method B, (82%, white powder), R_f = 0.3 (EtOAc/Petroleum ether = 1:1). **m.p.**: 201-203°C. **^{1}H NMR** (400 MHz, DMSO-*d6*) δ 12.20 (s, 1H), 8.21 – 8.17 (m, 2H), 8.05 – 8.00 (m, 3H), 7.94 (d, *J* = 3.2 Hz, 1H), 1.95 (s, 3H). **^{13}C NMR** (101 MHz, DMSO-*d6*) δ 168.9, 165.1, 144.5, 140.1, 137.3, 128.6, 126.7, 122.4, 23.2. **HRMS** (ESI): m/z (M + H^+) calcd for $C_{11}H_{11}O_3N_2S_2$, 283.0206, found: 283.0197.

N-((4-(pyridin-2-yloxy)phenyl)sulfonyl)acetamide (1p)

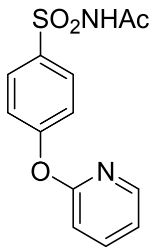

Method B, (88%, white powder), $\boldsymbol{R_f}$ = 0.4 (EtOAc/Petroleum ether = 1:1). **m.p.**: 161-163°C. ^{1}**H NMR** (400 MHz, DMSO-*d6*) δ 12.09 (s, 1H), 8.21 (ddd, *J* = 4.9, 2.0, 0.8 Hz, 1H), 7.97 – 7.91 (m, 3H), 7.36 – 7.32 (m, 2H), 7.23 (ddd, *J* = 7.2, 4.9, 0.9 Hz, 1H), 7.18 (dt, *J* = 8.2, 0.8 Hz, 1H), 1.95 (s, 3H). ^{13}**C NMR** (101 MHz, DMSO-*d6*) δ 168.8, 161.9, 158.1, 147.6, 140.7, 134.6, 129.7, 120.9, 120.2, 112.5, 23.3. **HRMS** (ESI): m/z (M + H$^+$) calcd for $C_{13}H_{13}O_4N_2S$, 293.0591, found: 293.0584.

N-((4-(5-(p-tolyl)-3-(trifluoromethyl)-1H-pyrazol-1-yl)phenyl)sulfonyl)acetamide (1q)

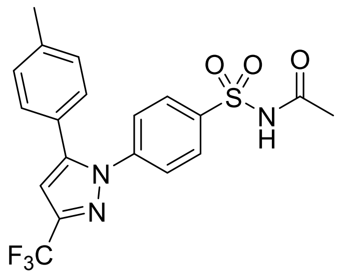

Method A, (90%, white powder), $\boldsymbol{R_f}$ = 0.4 (EtOAc/Petroleum ether = 1:1). **m.p.**: 169 – 170°C. ^{1}**H NMR** (400 MHz, DMSO-*d6*) δ 12.24 (s, 1H), 8.00 – 7.94 (m, 2H), 7.62 – 7.56 (m, 2H), 7.27 – 7.18 (m, 5H), 2.32 (s, 3H), 1.94 (s, 3H). ^{13}**C NMR** (101 MHz, DMSO-*d6*) δ 169.0, 145.4, 142.5, 142.4 (*J* = 37.9 Hz), 139.2, 139.1, 129.5, 128.8, 128.7, 126.0, 125.2, 121.2 (*J* = 270.1 Hz), 106.3, 23.2, 20.8. **MS** (ESI): m/z (M + H$^+$) 424.2.

N-((4-(1H-pyrazol-1-yl)phenyl)sulfonyl)acetamide (1r)

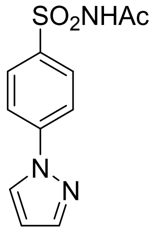

Method B, (83%, white powder), $\boldsymbol{R_f}$ = 0.3 (EtOAc/Petroleum ether = 1:1). **m.p.**: 240-242°C. ^{1}**H NMR** (400 MHz, DMSO-*d6*) δ 12.13 (s, 1H), 8.63 (d, *J* = 2.6 Hz, 1H), 8.12 – 8.07 (m, 2H), 8.04 – 8.00 (m, 2H), 7.84 (d, *J* = 1.7 Hz, 1H), 6.62 (dd, *J* = 2.5, 1.7 Hz, 1H), 1.94 (s, 3H). ^{13}**C NMR** (101 MHz, DMSO-*d6*) δ 168.9, 143.0, 142.3, 136.2, 129.4, 128.5, 118.3, 109.0, 23.2. **HRMS** (ESI): m/z (M + H$^+$) calcd for $C_{11}H_{12}O_3N_3S$, 266.0594, found: 266.0586

Dimethyl 2-diazomalonate (2a)

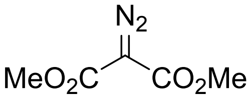

Method C, (78%, yellow oil), $\boldsymbol{R_f}$ = 0.4 (EtOAc/Petroleum ether = 1:4). ^{1}**H NMR** (400 MHz, $CDCl_3$) δ 3.79 (s, 1H). ^{13}**C NMR** (101 MHz, $CDCl_3$) δ 161.6, 52.6. **MS** (ESI): m/z (M + H$^+$) 159.2.

Dibenzyl 2-diazomalonate (2b)

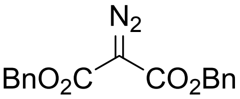

Method C, (75%, yellow oil), $\boldsymbol{R_f}$ = 0.4 (EtOAc/Petroleum ether = 1:4). ^{1}**H NMR** (400 MHz, $CDCl_3$) δ 7.41 – 7.30 (m, 10H), 5.28 (s, 4H). ^{13}**C NMR** (101 MHz, $CDCl_3$) δ 160.9, 135.4, 128.7, 128.6, 128.4, 67.2. **MS** (ESI): m/z (M + H$^+$) 311.2.

Methyl 2-diazo-2-(diethoxyphosphoryl)acetate (2c)

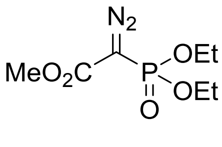

Method C, (76%, yellow oil), $\boldsymbol{R_f}$ = 0.3 (EtOAc/Petroleum ether = 1:1). ^{1}**H NMR** (400 MHz, $CDCl_3$) δ 4.28 – 4.05 (m, 4H), 3.77 (s, 3H), 1.35 – 1.30 (m, 6H). ^{13}**C NMR** (101 MHz, $CDCl_3$) δ 163.4, 63.5, 63.4, 52.3, 15.9, 15.8. **MS** (ESI): m/z (M + H$^+$) 237.2.

Methyl 2-diazo-2-tosylacetate (2d)

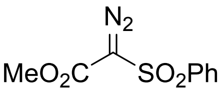

Method C, (75%, yellow powder, $\boldsymbol{R}_f$ = 0.3 (EtOAc/Petroleum ether = 1:2). **m.p.**: 68 – 69°C. **^{1}H NMR** (400 MHz, $CDCl_3$) δ 8.03 – 7.99 (m, 2H), 7.67 – 7.61 (m, 1H), 7.57 – 7.52 (m, 2H), 3.74 (s, 3H). **^{13}C NMR** (101 MHz, $CDCl_3$) δ 159.2, 141.8, 134.3, 129.4, 128.0, 53.0. **MS** (ESI): m/z (M + H^+) 254.1.

General procedure for the Rh-catalyzed sulfonamide directed C-H bond carbenoid founctionalization: A 50 mL tube equipped with a magnetic stir bar was charged with $[RhCp^*Cl_2]_2$ (5.0 mol%), AgOAc (20 mol%), sulfonamide containing *N*-heterocycle (0.125 mmol) and 25 mL toluene, then diazo compound (1.1 equivlent) was added. The tube was sealed, and the reaction mixture was stirred at 60°C over night. Toluene was removed under *vacuo*, and 20 mL DCM was added. The mixture was then filtered, the filtrate was concentrated, and the residue was purified by preparative TLC on silica gel to afford desired compound.

General procedure for the Rh-catalyzed heterocycle directed C-H bond carbenoid founctionalization: A 15 mL tube equipped with a magnetic stir bar was charged with $[RhCp^*Cl_2]_2$ (2.5 mol%), AgOAc (60 mol%), sulfonamide containing *N*-heterocycle (0.125 mmol) and 2.5 mL DCE, then diazo compound (1.1 equivlent) was added. The tube was sealed, and the reaction mixture was stirred at 60°C over night. DCE was removed under *vacuo*, and 20 mL DCM was added. The mixture was then filtered, the filtrate was concentrated, and the residue was purified by preparative TLC on silica gel to afford desired compound.

Dimethyl 2-(2-(*N*-acetylsulfamoyl)-4-methyl-5-(thiazol-2-yl)phenyl)malonate (3)

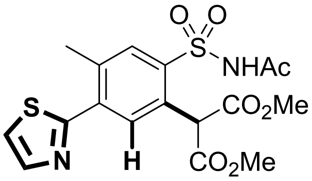

$[RhCp^*Cl_2]_2$ (3.9 mg, 5.0 mol%), AgOAc (4.2 mg, 20 mol%), ***N-((3-methyl-4-(thiazol-2-yl)phenyl)sulfonyl)acetamide*** (37 mg, 0.125 mmol), **2a** (22 mg, 1.1 equiv), 25 mL toluene, 60°C overnight. 43 mg **3** was obtained (81% yield, white powder, EAOAc/ Petroleum ether = 1:1, $\boldsymbol{R}_f$ = 0.3), **m.p.**: 152 – 154°C. **^{1}H NMR** (400 MHz, DMSO-*d6*) δ 12.54 (s, 1H), 8.10 (d, *J* = 3.2 Hz, 1H), 8.02 (d, *J* = 3.2 Hz, 1H), 8.01 (s, 1H), 7.98 (s, 1H), 5.81 (s, 1H), 3.71 (s, 6H), 2.66 (s, 3H), 1.91 (s, 3H). **^{13}C NMR** (101 MHz, DMSO-*d6*) δ 169.2, 167.8, 163.9, 143.8, 137.9, 136.7, 136.2, 133.6, 131.4, 129.0, 122.7, 53.2, 51.9, 23.2, 21.1. **HRMS** (ESI): m/z (M + H^+) calcd for $C_{17}H_{19}O_7N_2S_2$, 427.0628, found: 427.0616. IR (KBr): 3116, 2954, 1734, 1584, 1434, 1307, 1263, 1123, 1028, 909, 733, 703, 657 cm^{-1}. **IR** (neat): 3116, 2954, 1734, 1584, 1434, 1307, 1263, 1123, 1028, 909, 733, 703, 657 cm^{-1}.

Dimethyl 2-(5-(*N*-acetylsulfamoyl)-3-methyl-2-(thiazol-2-yl)phenyl)malonate (3')

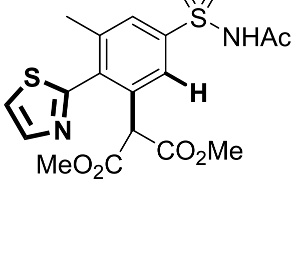

$[RhCp^*Cl_2]_2$ (2.0 mg, 2.5 mol%), AgOAc (12.6 mg, 60 mol%), ***N-((3-methyl-4-(thiazol-2-yl)phenyl)sulfonyl)acetamide*** (37 mg, 0.125 mmol),

2a (22 mg, 1.1 equiv), 2.5 mL DCE, 60°C overnight. 46.7 mg **3'** was obtained (88% yield, white powder, EAOAc/ Petroleum ether = 1:1, ***R*****f** = 0.3), **m.p.**: 164 – 166°C. **^{1}H NMR** (400 MHz, DMSO-*d6*) δ 12.22 (s, 1H), 8.03 (s, 2H), 7.83 (d, *J* = 1.2 Hz, 1H), 7.78 (d, *J* = 1.2 Hz, 1H), 4.49 (s, 1H), 3.61 (s, 6H), 2.14 (s, 3H), 1.88 (s, 3H). **^{13}C NMR** (101 MHz, DMSO-*d6*) δ 170.0, 167.4, 161.8, 143.5, 141.5, 139.1, 137.9, 133.5, 128.4, 125.3, 123.3, 54.1, 53.1, 23.8, 20.2. **HRMS** (ESI): m/z (M + H$^+$) calcd for $C_{17}H_{19}O_7N_2S_2$, 427.0628, found: 427.0618. **IR** (neat): 3111, 2955, 1736, 1585, 1437, 1300, 1257, 1119, 1004, 921, 754,699, 653 cm^{-1}.

Dimethyl 2-(2-(*N*-acetylsulfamoyl)-3-methyl-5-(thiazol-2-yl)phenyl)malonate (4)

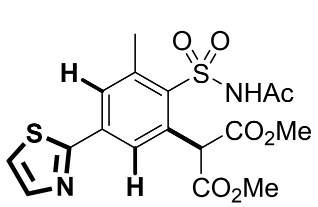

[RhCp*Cl$_2$]$_2$ (3.9 mg, 5.0 mol%), AgOAc (4.2 mg, 20 mol%), ***N-((2-methyl-4-(thiazol-2-yl)phenyl)sulfonyl)acetamide*** (37 mg, 0.125 mmol), **2a** (22 mg, 1.1 equiv), 25 mL toluene, 60°C overnight. 44 mg **4** was obtained (83% yield, white powder, EAOAc/ Petroleum ether = 1:1, ***R*****f** = 0.3), **m.p.**: 193 – 194°C. **^{1}H NMR** (400 MHz, DMSO-*d6*) δ 12.60 (s, 1H), 8.04 (d, *J* = 3.2 Hz, 1H), 7.97 – 7.95 (m, 2H), 7.83 (d, *J* = 1.8 Hz, 1H), 6.17 (s, 1H), 3.71 (s, 6H), 2.75 (s, 3H), 1.96 (s, 3H). **^{13}C NMR** (101 MHz, DMSO-*d6*) δ 169.9, 168.5, 164.3, 144.6, 141.7, 137.0, 136.0, 135.2, 130.4, 125.5, 122.7, 54.0, 52.9, 23.0, 22.2. **HRMS** (ESI): m/z (M + H$^+$) calcd for $C_{17}H_{19}O_7N_2S_2$, 427.0628, found: 427.0616. **IR:** 3234, 2923, 1737, 1435, 1312, 1266, 1221, 1160, 1092, 1035, 857, 654, 527 cm^{-1}.

Dimethyl 2-(2-(*N*-acetylsulfamoyl)-5-(thiazol-2-yl)-3-(trifluoromethoxy)phenyl)malonate (5)

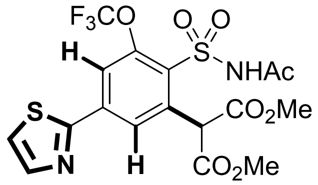

[RhCp*Cl$_2$]$_2$ (3.9 mg, 5.0 mol%), AgOAc (4.2 mg, 20 mol%), ***N-((4-(thiazol-2-yl)-2-(trifluoromethoxy)phenyl)sulfonyl)acetamide*** (45.7 mg, 0.125 mmol), **2a** (22 mg, 1.1 equiv), 25 mL toluene, 60°C overnight. 53.5 mg **5** was obtained (86% yield, white powder, EAOAc/ Petroleum ether = 1:1, ***R*****f** = 0.3), **m.p.**: 195 – 196°C. **^{1}H NMR** (400 MHz, DMSO-*d6*) δ 12.85 (s, 1H), 8.10 (d, *J* = 3.2 Hz, 1H), 8.05 (d, *J* = 3.2 Hz, 1H), 8.02 (s, 1H), 7.92 (d, *J* = 1.5 Hz, 1H), 6.25 (s, 1H), 3.74 (s, 6H), 1.95 (s, 3H). **^{13}C NMR** (151 MHz, DMSO-*d6*) δ 170.3, 167.9, 162.8, 147.4, 145.0, 137.8, 137.1, 131.5, 126.1, 123.8, 119.9 (q, *J* = 260.0 Hz), 118.2, 53.3, 53.1, 23.0. **HRMS** (ESI): m/z (M + H$^+$) calcd for $C_{17}H_{16}O_8N_2F_3S_2$, 497.0295, found: 497.0281. **IR** (neat): 3225, 2922, 1719, 1435, 1365, 1314, 1259, 1204, 1160, 1087, 1042, 736, 618, 522 cm^{-1}.

Dimethyl 2-(1-(*N*-acetylsulfamoyl)-4-(thiazol-2-yl)naphthalen-2-yl)malonate (6)

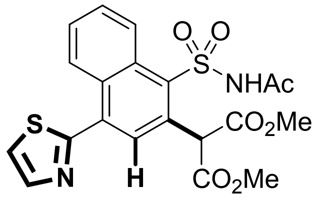

[RhCp*Cl$_2$]$_2$ (3.9 mg, 5.0 mol%), AgOAc (4.2 mg, 20 mol%), ***N-((4-(thiazol-2-yl)naphthalen-1-yl)sulfonyl)acetamide*** (41.5 mg, 0.125 mmol), **2a** (22 mg, 1.1 equiv), 25 mL toluene, 60°C overnight. 47.5 mg **6** was obtained (82% yield, white powder, EAOAc/ Petroleum ether = 1:1, ***R*****f** = 0.3), **m.p.**: 153 – 154°C.**^{1}H NMR** (400 MHz, DMSO-*d6*) δ 12.96

(s, 1H), 8.93 (d, *J* = 8.2 Hz, 1H), 8.78 (dd, *J* = 8.4, 1.4 Hz, 1H), 8.20 (d, *J* = 3.3 Hz, 1H), 8.09 (d, *J* = 3.3 Hz, 1H), 7.85 – 7.75 (m, 2H), 7.70 (s, 1H), 6.66 (s, 1H), 3.73 (s, 6H), 1.93 (s, 3H). **^{13}C NMR** (101 MHz, DMSO-*d6*) δ 171.0, 169.0, 164.7, 144.9, 135.6, 134.5, 130.4 (X2), 129.5, 129.0, 128.7, 126.7, 126.4, 126.3, 123.6, 54.7, 53.4, 23.7. **HRMS** (ESI): m/z (M + H$^+$) calcd for $C_{20}H_{19}O_7N_2S_2$, 463.0628, found: 463.0620. **IR** (neat): 3220, 2953, 1732, 1488, 1434, 1337, 1222, 1158, 1144, 1029, 857, 758, 659, 526 cm^{-1}.

Dimethyl 2-(2-(*N*-acetylsulfamoyl)-3-methyl-5-(1H-pyrazol-1-yl)phenyl)malonate (7)

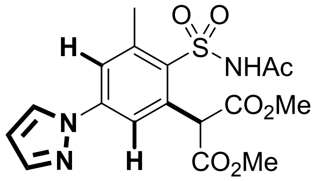

[RhCp*Cl$_2$]$_2$ (3.9 mg, 5.0 mol%), AgOAc (4.2 mg, 20 mol%), ***N*-((2-methyl-4-(1H-pyrazol-1-yl)phenyl)sulfonyl)acetamide** (34.9 mg, 0.125 mmol), **2a** (22 mg, 1.1 equiv), 25 mL toluene, 60°C overnight. 40 mg **7** was obtained (78% yield, white powder, EAOAc/ Petroleum ether = 2:1, ***R*$_f$** = 0.4), **m.p.**: 155 – 156°C. **^{1}H NMR** (400 MHz, DMSO-*d6*) δ 12.52 (s, 1H), 8.63 (d, *J* = 2.6 Hz, 1H), 7.94 (d, *J* = 2.2 Hz, 1H), 7.84 (d, *J* = 1.7 Hz, 1H), 7.75 (d, *J* = 2.3 Hz, 1H), 6.63 (dd, *J* = 2.5, 1.8 Hz, 1H), 6.16 (s, 1H), 3.70 (s, 6H), 2.74 (s, 3H), 1.94 (s, 3H). **^{13}C NMR** (101 MHz, DMSO-*d6*) δ 169.9, 168.4, 142.5, 142.5, 141.5, 136.1, 133.3, 128.5, 121.2, 117.9, 109.1, 54.1, 52.9, 23.0, 22.5. **HRMS** (ESI): m/z (M + H$^+$) calcd for $C_{17}H_{20}O_7N_3S$, 410.1016, found: 410.1010. **IR** (neat): 3228, 2954, 1718, 1599, 1435, 1390, 1160, 1137, 1041, 859, 735, 649, 518 cm^{-1}.

Dimethyl 2-(2-(*N*-acetylsulfamoyl)-5-(3,5-dimethyl-1H-pyrazol-1-yl)-4-methylphenyl)malonate (8)

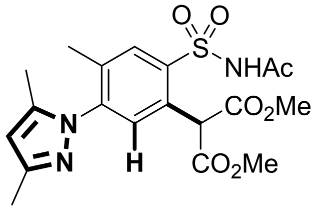

[RhCp*Cl$_2$]$_2$ (3.9 mg, 5.0 mol%), AgOAc (4.2 mg, 20 mol%), ***N*-((4-(3,5-dimethyl-1H-pyrazol-1-yl)-3-methylphenyl)sulfonyl)acetamide** (38.4 mg, 0.125 mmol), **2a** (22 mg, 1.1 equiv), 25 mL toluene, 60°C overnight. 31.5 mg **8** was obtained (57% yield, white powder, EAOAc/ Petroleum ether = 2:1, ***R*$_f$** = 0.4), **m.p.**: 159 – 160°C. **^{1}H NMR** (400 MHz, DMSO-*d6*) δ 12.56 (s, 1H), 8.03 (s, 1H), 7.29 (s, 1H), 6.10 (s, 1H), 5.81 (s, 1H), 3.70 (s, 6H), 2.18 (s, 3H), 2.16 (s, 3H), 2.06 (s, 3H), 1.93 (s, 3H). **^{13}C NMR** (151 MHz, DMSO-*d6*) δ 169.0, 167.6, 148.4, 142.0, 140.1, 137.4, 136.2, 133.2, 129.7, 129.5, 106.2, 53.2, 51.8, 23.1, 17.2, 13.3, 10.9. **HRMS** (ESI): m/z (M + H$^+$) calcd for $C_{19}H_{24}O_7N_3S$, 438.1329, found: 438.1312. **IR** (neat): 3225, 2955, 1720, 1591, 1431, 1397, 1156, 1136, 1037, 864, 753, 621 cm^{-1}.

Dimethyl 2-(2-(*N*-acetylsulfamoyl)-4-methyl-5-(1H-pyrazol-1-yl)phenyl)malonate (9)

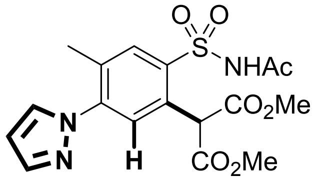

[RhCp*Cl$_2$]$_2$ (3.9 mg, 5.0 mol%), AgOAc (4.2 mg, 20 mol%), ***N*-((3-methyl-4-(1H-pyrazol-1-yl)phenyl)sulfonyl)acetamide** (34.7 mg, 0.125 mmol), **2a** (22 mg, 1.1 equiv), 25 mL toluene, 60°C overnight. 41.3 mg **9** was obtained (80% yield, white powder, EAOAc/ Petroleum ether = 2:1, ***R*$_f$** = 0.4), **m.p.**: 83 – 84°C. **^{1}H NMR** (400 MHz, DMSO-*d6*) δ 12.54 (s, 1H), 8.21 (d, *J* = 2.3 Hz, 1H), 8.03 (s, 1H), 7.82 (d, *J* = 1.5 Hz, 1H), 7.50 (s, 1H), 6.60 – 6.58 (m, 1H), 5.82 (s, 1H), 3.70 (s, 6H), 2.39 (s, 3H), 1.90 (s, 3H). **^{13}C NMR** (101 MHz, DMSO-*d6*) δ 169.3, 167.7, 142.8, 141.3, 136.7, 133.9, 132.0,

131.7, 129.8, 127.1, 107.5, 53.2, 51.9, 23.3, 18.3. **HRMS** (ESI): m/z (M + H+) calcd for $C_{17}H_{20}O_7N_3S$, 410.1016, found: 410.1005. **IR** (neat): 3226, 2956, 1733, 1563, 1434, 1400, 1257, 1219, 902, 964, 734, 630, 522 cm^{-1}.

Dimethyl 2-(7-(*N*-acetylsulfamoyl)-4,5-diphenylpyrazolo[1,5-a]quinolin-8-yl)malonate (10)

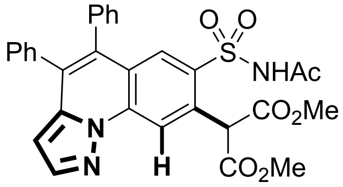

[RhCp*Cl$_2$]$_2$ (3.9 mg, 5.0 mol%), AgOAc (4.2 mg, 20 mol%), ***N*-((4,5-diphenylpyrazolo[1,5-a]quinolin-7-yl)sulfonyl)acetamide** (55.1 mg, 0.125 mmol), **2a** (22 mg, 1.1 equiv), 25 mL toluene, 60°C overnight. 53.2 mg **10** was obtained (75% yield, white powder, EAOAc/ Petroleum ether = 2:1, R_f = 0.3), **m.p.**: 223 – 224°C. **^{1}H NMR** (400 MHz, $CDCl_3$) δ 8.95 (s, 1H), 8.86 (s, 1H), 8.47 (s, 1H), 8.07 (d, *J* = 2.1 Hz, 1H), 7.36 – 7.29 (m, 5H), 7.24 – 7.18 (m, 4H), 6.44 (d, *J* = 2.0 Hz, 1H), 6.00 (s, 1H), 3.83 (s, 6H), 1.98 (s, 3H). **^{13}C NMR** (101 MHz, $CDCl_3$) δ 168.2, 168.1, 143.2, 140.4, 136.7, 136.4, 135.2, 133.5, 133.2, 132.8, 131.6, 131.4, 131.2, 130.0, 128.6, 128.4, 128.3, 128.1, 123.6, 119.4, 102.7, 53.8 (X2), 23.5. **HRMS** (ESI): m/z (M + H+) calcd for $C_{30}H_{26}O_7N_3S$, 572.1486, found: 572.1480. **IR** (neat): 3021, 2954, 1741, 1591, 1489, 1437, 1148, 994, 854, 764, 699 cm^{-1}.

Dimethyl 2-(2-(*N*-acetylsulfamoyl)-4-methyl-5-(1-methyl-1H-benzo[d]imidazol-2-yl)phenyl)malonate (11)

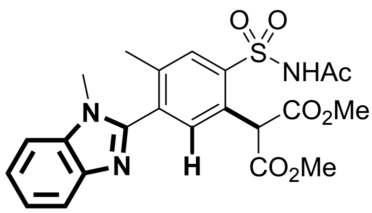

[RhCp*Cl$_2$]$_2$ (3.9 mg, 5.0 mol%), AgOAc (4.2 mg, 20 mol%), ***N*-((3-methyl-4-(1-methyl-1H-benzo[d]imidazol-2-yl)phenyl)sulfonyl)acetamide** (42.8 mg, 0.125 mmol), **2a** (22 mg, 1.1 equiv), 25 mL toluene, 60°C overnight. 42.2 mg **11** was obtained (71% yield, white powder, EAOAc/ Petroleum ether = 3:1, R_f = 0.3), **m.p.**: 236 – 238°C. **^{1}H NMR** (400 MHz, DMSO-*d6*) δ 12.55 (s, 1H), 8.02 (s, 1H), 7.69 (d, *J* = 7.7 Hz, 1H), 7.60 (d, *J* = 7.8 Hz, 1H), 7.52 (s, 1H), 7.33 – 7.29 (m, 1H), 7.27 – 7.23 (m, 1H), 5.82 (s, 1H), 3.66 (s, 6H), 3.62 (s, 3H), 2.35 (s, 3H), 1.90 (s, 3H). **^{13}C NMR** (151 MHz, DMSO-*d6*) δ 169.2, 167.7, 150.5, 142.3, 138.7, 138.6, 135.6, 134.4, 132.9, 132.3, 128.5, 122.8, 122.2, 119.2, 110.8, 53.2, 51.9, 30.8, 23.2, 19.4. **HRMS** (ESI): m/z (M + H+) calcd for $C_{22}H_{24}O_7N_3S$, 474.1329, found: 474.1319. **IR** (neat): 3061, 2953, 1734, 1647, 1461, 1270, 1148, 1019, 878, 733, 660, 637, 582 cm^{-1}.

Dimethyl 2-(2-(*N*-acetylsulfamoyl)-4-methyl-5-(pyridin-2-yl)phenyl)malonate (12)

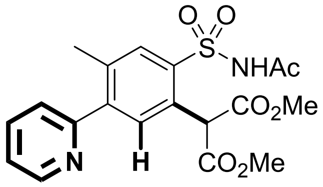

[RhCp*Cl$_2$]$_2$ (3.9 mg, 5.0 mol%), AgOAc (4.2 mg, 20 mol%), ***N*-((3-methyl-4-(pyridin-2-yl)phenyl)sulfonyl)acetamide** (36.2 mg, 0.125 mmol), **2a** (22 mg, 1.1 equiv), 25 mL toluene, 60°C overnight. 38.5 mg **12** was obtained (73% yield, white powder, EAOAc/ Petroleum ether = 2:1, R_f = 0.4), **m.p.**: 161 – 162°C. **^{1}H NMR** (400 MHz, DMSO-*d6*) δ 12.50 (s, 1H), 8.75 – 8.71 (m, 1H), 7.98 – 7.90 (m, 2H), 7.59 (d, *J* = 7.8 Hz, 1H), 7.50 (s, 1H), 7.46 (ddd, *J* = 7.6, 4.8, 0.9 Hz, 1H), 5.82 (s, 1H), 3.69 (s, 6H), 2.42 (s, 3H), 1.91 (s, 3H). **^{13}C NMR** (151 MHz, DMSO-*d6*) δ 169.2, 167.9, 156.6, 149.5,

144.4, 137.4, 137.0, 136.1, 132.6, 131.8, 128.6, 124.2, 123.0, 53.0, 52.0, 23.2, 19.8. **HRMS** (ESI): m/z (M + H+) calcd for $C_{19}H_{21}O_7N_2S$, 421.1064, found: 421.1056. **IR** (neat): 3239, 2955, 1736, 1435, 1343, 1263, 1221, 1197, 996, 866, 735, 628, 526 cm^{-1}.

Dimethyl 2-(2-(*N*-acetylsulfamoyl)-4-methyl-5-(pyrimidin-2-yl)phenyl)malonate (13)

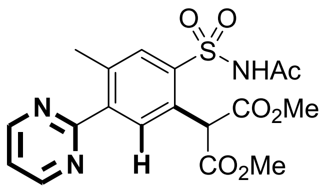

$[RhCp^*Cl_2]_2$ (3.9 mg, 5.0 mol%), AgOAc (4.2 mg, 20 mol%), ***N-((3-methyl-4-(pyrimidin-2-yl)phenyl)sulfonyl)acetamide*** (36.3 mg, 0.125 mmol), **2a** (22 mg, 1.1 equiv), 25 mL toluene, 60°C overnight. 39.5 mg **13** was obtained (75% yield, white powder, EAOAc/ Petroleum ether = 2:1, $\boldsymbol{R_f}$ = 0.3), **m.p.**: 180 – 181°C. **^{1}H NMR** (400 MHz, DMSO-*d6*) δ 12.52 (s, 1H), 9.00 (d, *J* = 4.2 Hz, 2H), 7.96 (d, *J* = 10.0 Hz, 2H), 7.56 (s, 1H), 5.82 (s, 1H), 3.70 (s, 6H), 2.59 (s, 3H), 1.91 (s, 3H). **^{13}C NMR** (101 MHz, DMSO-*d6*) δ 169.1, 167.9, 164.4, 157.6, 142.0, 138.1, 137.5, 133.2, 132.6, 131.5, 128.5, 120.1, 53.1, 52.0, 23.2, 20.7. **HRMS** (ESI): m/z (M + H+) calcd for $C_{18}H_{20}O_7N_3S$, 422.1016, found: 422.1009. **IR** (neat): 3232, 2956, 1735, 1565, 1434, 1344, 1310, 1267, 1221, 996, 866, 735, 653, 526 cm^{-1}.

Dimethyl 2-(2-(*N*-acetylsulfamoyl)-4-methyl-5-(pyrazin-2-yl)phenyl)malonate (14)

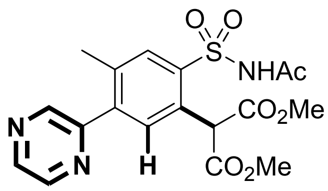

$[RhCp^*Cl_2]_2$ (3.9 mg, 5.0 mol%), AgOAc (4.2 mg, 20 mol%), ***N-((3-methyl-4-(pyrazin-2-yl)phenyl)sulfonyl)acetamide*** (36.4 mg, 0.125 mmol), **2a** (22 mg, 1.1 equiv), 25 mL toluene, 60°C overnight. 27 mg **14** was obtained (51% yield, white powder, EAOAc/ Petroleum ether = 2:1, $\boldsymbol{R_f}$ = 0.4), **m.p.**: 204 – 206°C. **^{1}H NMR** (400 MHz, DMSO-*d6*) δ 12.54 (s, 1H), 8.90 (d, *J* = 1.5 Hz, 1H), 8.83 – 8.81 (m, 1H), 8.73 (d, *J* = 2.5 Hz, 1H), 8.00 (s, 1H), 7.57 (s, 1H), 5.83 (s, 1H), 3.70 (s, 6H), 2.46 (s, 3H), 1.92 (s, 3H). **^{13}C NMR** (101 MHz, DMSO-*d6*) δ 169.5, 167.8, 152.3, 144.8, 144.4, 144.0, 141.0, 138.4, 136.6, 132.7, 132.1, 128.8, 53.1, 52.0, 23.4, 19.7. **HRMS** (ESI): m/z (M + H+) calcd for $C_{18}H_{20}O_7N_3S$, 422.1016, found: 422.1011. **IR** (neat): 3244, 2955, 1741, 1566, 1477, 1351, 1287, 1211, 998, 853, 746, 654 cm^{-1}.

Dimethyl 2-(2-(*N*-acetylsulfamoyl)-5-(pyridin-2-yl)thiophen-3-yl)malonate (15)

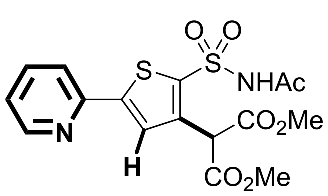

$[RhCp^*Cl_2]_2$ (3.9 mg, 5.0 mol%), AgOAc (4.2 mg, 20 mol%), ***N-((5-(pyridin-2-yl)thiophen-2-yl)sulfonyl)acetamide*** (35.2 mg, 0.125 mmol), **2a** (22 mg, 1.1 equiv), 25 mL toluene, 60°C overnight. 37.5 mg **15** was obtained (72% yield, white powder, EAOAc/ Petroleum ether = 2:1, $\boldsymbol{R_f}$ = 0.3), **m.p.**: 204 – 206°C. **^{1}H NMR** (400 MHz, DMSO-*d6*) δ 12.50 (s, 1H), 8.56 (ddd, *J* = 4.9, 1.7, 1.0 Hz, 1H), 8.07 (d, *J* = 8.0 Hz, 1H), 7.88 (td, *J* = 7.8, 1.7 Hz, 1H), 7.74 (s, 1H), 7.39 (ddd, *J* = 7.6, 4.9, 1.0 Hz, 1H), 5.64 (s, 1H), 3.70 (s, 6H), 1.89 (s, 3H). ^{13}C NMR (151 MHz, DMSO-*d6*) δ 169.3, 167.0, 149.8, 149.6, 149.3, 141.9, 137.7, 134.2, 126.2, 124.3, 119.6, 53.1,

50.3, 23.3. **HRMS** (ESI): m/z (M + H+) calcd for $C_{16}H_{17}O_7N_2S_2$, 413.0472, found: 413.0458. **IR** (neat): 3245, 3109, 2952, 1731, 1568, 1470, 1301, 1217, 1155, 979, 785, 665, 414 cm-1.

Dimethyl 2-(2-(*N*-acetylsulfamoyl)-5-(2-methylthiazol-4-yl)thiophen-3-yl)malonate (16)

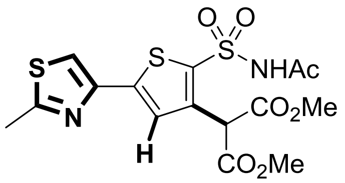

[RhCp*Cl2]2 (3.9 mg, 5.0 mol%), AgOAc (4.2 mg, 20 mol%), ***N-((5-(2-methylthiazol-4-yl)thiophen-2-yl)sulfonyl)acetamide*** (37.7 mg, 0.125 mmol), **2a** (22 mg, 1.1 equiv), 25 mL toluene, 60°C overnight. 40 mg **16** was obtained (74% yield, white powder, EAOAc/ Petroleum ether = 1:1, ***R*f** = 0.3), **m.p.**: 139 – 140°C. **1H NMR** (400 MHz, DMSO-*d6*) δ 12.49 (s, 1H), 8.24 (s, 1H), 7.58 (s, 1H), 5.65 (s, 1H), 3.73 (s, 6H), 2.71 (s, 3H), 1.94 (s, 3H). **13C NMR** (151 MHz, DMSO-*d6*) δ 169.0, 167.0, 167.0, 145.9, 143.0, 137.8, 134.6, 125.1, 116.6, 53.2, 50.2, 23.1, 18.6. **HRMS** (ESI): m/z (M + H+) calcd for $C_{15}H_{17}O_7N_2S_3$, 433.0192, found: 433.0185. **IR** (neat): 3240, 2955, 1736, 1435, 1315, 1219, 1141, 1065, 854, 646, 528 cm-1.

Methyl 2-(2-(*N*-acetylsulfamoyl)-3-methyl-5-(thiazol-2-yl)phenyl)-2-(diethoxyphosphoryl)acetate (17)

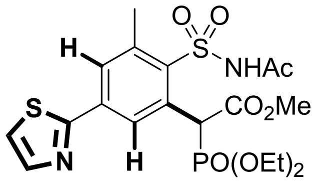

[RhCp*Cl2]2 (3.9 mg, 5.0 mol%), AgOAc (4.2 mg, 20 mol%), ***N-((2-methyl-4-(thiazol-2-yl)phenyl)sulfonyl)acetamide*** (37mg, 0.125 mmol), **2d** (33 mg, 1.1 equiv), 25 mL toluene, 60°C overnight. 50 mg **17** was obtained (80% yield, white powder, EAOAc/ Petroleum ether = 2:1, ***R*f** = 0.4), **m.p.**: 185 – 186°C. **1H NMR** (400 MHz, DMSO-*d6*) δ 12.56 (s, 1H), 8.39 (t, *J* = 1.9 Hz, 1H), 8.04 (d, *J* = 3.2 Hz, 1H), 7.95 (d, *J* = 3.2 Hz, 2H), 6.07 (d, *J* = 28.2 Hz, 1H), 4.17 – 4.08 (m, 2H), 3.96 – 3.79 (m, 2H), 3.70 (s, 3H), 2.74 (s, 3H), 1.92 (s, 3H), 1.26 (t, *J* = 7.1 Hz, 3H), 1.00 (t, *J* = 7.0 Hz, 3H). **13C NMR** (151 MHz, DMSO-*d6*) δ 169.7, 167.2 (d, *J* = 4.7 Hz), 164.6, 144.5, 141.1, 137.1, 135.2, 134.3 (d, *J* = 4.5 Hz), 129.6, 128.5 (d, *J* = 5.4 Hz), 122.5, 62.8 (dd, *J* = 6.9, 24.3 Hz), 52.7, 46.9, 46.0, 23.0, 22.7, 16.2 (d, *J* = 5.7 Hz), 15.9 (d, *J* = 5.4 Hz). **HRMS** (ESI): m/z (M + H+) calcd for $C_{19}H_{26}O_8N_2PS_2$, 505.0863, found: 505.0851. **IR** (neat): 3445, 2991, 1744, 1601, 1488, 1457, 1342, 1238, 1159, 1020, 856, 745, 645 cm-1.

Methyl 2-(2-(*N*-acetylsulfamoyl)-3-methyl-5-(thiazol-2-yl)phenyl)-2-(phenylsulfonyl)acetate (18)

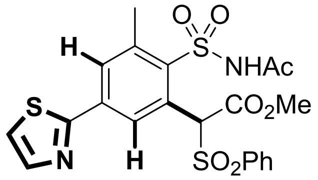

[RhCp*Cl2]2 (3.9 mg, 5.0 mol%), AgOAc (4.2 mg, 20 mol%), ***N-((2-methyl-4-(thiazol-2-yl)phenyl)sulfonyl)acetamide*** (37mg, 0.125 mmol), **2f** (33 mg, 1.1 equiv), 25 mL toluene, 60°C overnight. 51.5 mg **18** was obtained (81% yield, white powder, EAOAc/ Petroleum ether = 2:1, ***R*f** = 0.4), **m.p.**: 226 – 228°C. **1H NMR** (400 MHz, DMSO-*d6*) δ 12.67 (s, 1H), 8.52 (d, *J* = 1.9 Hz, 1H), 8.09 – 8.06 (m, 2H), 7.99 (d, *J* = 3.2 Hz, 1H), 7.94 – 7.90 (m, 2H), 7.83 – 7.78 (m, 1H), 7.70 (dt, *J* = 7.3, 1.6 Hz, 2H), 7.37 (s, 1H), 3.60 (s, 3H), 2.74 (s, 3H), 1.92 (s, 3H). **13C NMR** (101 MHz, DMSO-*d6*) δ 170.1, 164.5, 164.3, 144.7, 141.4, 138.7, 137.8, 135.4, 134.6, 131.2, 130.5, 129.3, 128.8, 127.9, 122.8, 68.4,

53.1, 23.0, 22.4. HRMS (ESI): m/z (M + H+) calcd for $C_{21}H_{21}O_7N_2S_3$, 509.0505, found: 509.0493. IR **(neat):** 3446, 2955, 1719, 1588, 1459, 1426, 1348, 1222, 1153, 1002, 852, 774, 675 cm^{-1}.

Tetramethyl 2,2'-(2-(*N*-acetylsulfamoyl)-5-(thiazol-2-yl)-1,3-phenylene)dimalonate (19)

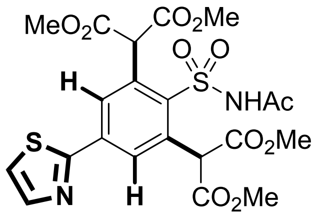

$[RhCp^*Cl_2]_2$ (3.9 mg, 5.0 mol%), AgOAc (4.2 mg, 20 mol%), ***N-((4-(thiazol-2-yl)phenyl)sulfonyl)acetamide*** (35.2 mg, 0.125 mmol), **2a** (22 mg, 1.1 equiv), 25 mL toluene, 60°C overnight. 30.5 mg **19** was obtained (45% yield, white powder, EAOAc/ Petroleum ether = 3:1, R_f = 0.3), **m.p.**: 195 – 197°C. **^{1}H NMR** (400 MHz, DMSO-*d6*) δ 12.99 (s, 1H), 8.05 (d, *J* = 3.2 Hz, 1H), 7.97 (d, *J* = 3.1 Hz, 1H), 7.92 (s, 2H), 6.23 (s, 2H), 3.71 (s, 12H), 1.90 (s, 3H). **^{13}C NMR** (101 MHz, DMSO) δ 171.1, 168.4, 163.9, 144.8, 138.9, 135.2, 129.6, 127.8, 122.8, 53.8, 53.0, 23.5. **HRMS** (ESI): m/z (M + H+) calcd for $C_{21}H_{23}O_{11}N_2S_2$, 543.0738, found: 543.0737. **IR** (neat): 3212, 2955, 1734, 1603, 1434, 1312, 1253, 1221, 1091, 1037, 860, 734, 652, 529 cm^{-1}.

Tetramethyl 2,2'-(2-(*N*-acetylsulfamoyl)-5-(pyridin-2-yloxy)-1,3-phenylene)dimalonate (20)

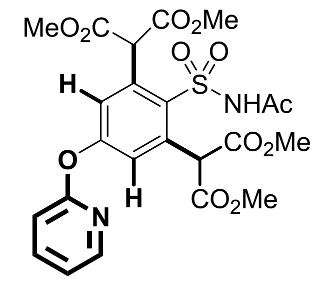

$[RhCp^*Cl_2]_2$ (3.9 mg, 5.0 mol%), AgOAc (4.2 mg, 20 mol%), ***N-((4-(pyridin-2-yloxy)phenyl)sulfonyl)acetamide*** (36.5 mg, 0.125 mmol), **2a** (44 mg, 2.2 equiv), 25 mL toluene, 60°C overnight. 59 mg **20** was obtained (85% yield, white powder, EAOAc/ Petroleum ether = 3:1, R_f = 0.3), **m.p.**: 183 – 185°C. **^{1}H NMR** (400 MHz, DMSO-*d6*) δ 12.85 (s, 1H), 8.24 (dd, *J* = 4.9, 1.4 Hz, 1H), 7.96 (td, *J* = 8.2, 2.0 Hz, 1H), 7.27 (dd, *J* = 6.9, 5.2 Hz, 1H), 7.19 (d, *J* = 8.2 Hz, 1H), 7.03 (s, 2H), 6.20 (s, 2H), 3.66 (s, 12H), 1.87 (s,3H). **^{13}C NMR** (101 MHz, DMSO-*d6*) δ 171.7, 168.4, 161.2, 155.8, 147.6, 140.9, 135.9, 129.3, 121.4, 120.7, 112.9, 54.0, 52.8, 23.7. **HRMS** (ESI): m/z (M + H+) calcd for $C_{23}H_{25}O_{12}N_2S$, 553.1123, found: 553.1114. **IR** (neat): 3224, 2956, 1736, 1589, 1431, 1302, 1267, 1223, 1064, 859, 735, 655, 526 cm^{-1}.

Tetramethyl 2,2'-(2-(*N*-acetylsulfamoyl)-5-(5-(p-tolyl)-3-(trifluoromethyl)-1H-pyrazol-1-yl)-1,3-phenylene)dimalonate (21)

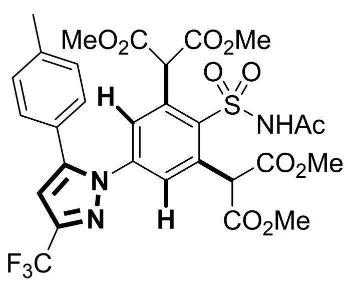

$[RhCp^*Cl_2]_2$ (3.9 mg, 5.0 mol%), AgOAc (4.2 mg, 20 mol%), ***N*-((4-(5-(p-tolyl)-3-(trifluoromethyl)-1H-pyrazol-1-yl)phenyl)sulfonyl)acetamide** (52.8 mg, 0.125 mmol), **2a** (44 mg, 2.2 equiv), 25 mL toluene, 60°C overnight. 73 mg **21** was obtained (86% yield, white powder, DCM/ MeOH = 30:1, R_f = 0.3, **m.p.**: 207 – 208°C. **^{1}H NMR** (400 MHz, DMSO-*d6*) δ 7.23 (d, *J* = 8.1 Hz, 2H), 7.18 (d, *J* = 8.2 Hz, 2H), 7.16 (s, 2H), 7.13 (s, 1H), 6.60 (s, 1H), 3.53 (s, 12H), 2.31 (s, 3H), 1.62 (s, 3H). **^{13}C NMR** (151 MHz, DMSO-*d6*) δ 170.8, 169.1, 145.5, 142.7 (q, 36.4 Hz), 139.4, 134.9, 130.0, 128.9, 126.2, 125.6, 124.4, 122.6, 120.8, 107.0, 54.1, 52.9, 21.3. **HRMS** (ESI): m/z (M + H+) calcd for $C_{29}H_{29}O_{11}N_3F_3S$,

684.1469, found: 684.1452. **IR** (neat): 3221, 2956, 1738, 1601, 1435, 1374, 1246, 1179, 1038, 973, 861, 736, 649, 525 cm^{-1}.

Tetramethyl 2,2'-(2-(*N*-acetylsulfamoyl)-5-(1H-pyrazol-1-yl)-1,3-phenylene)dimalonate (22)

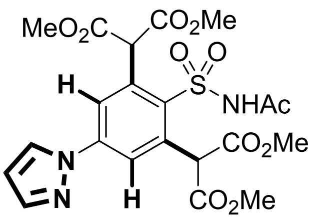

$[RhCp^*Cl_2]_2$ (3.9 mg, 5.0 mol%), AgOAc (4.2 mg, 20 mol%), ***N-((4-(1H-pyrazol-1-yl)phenyl)sulfonyl)acetamide*** (33.1 mg, 0.125 mmol), **2a** (22 mg, 1.1 equiv), 25 mL toluene, 60°C overnight. 26 mg **22** was obtained (40% yield, white powder, EAOAc/ Petroleum ether = 3:1, R_f = 0.3), **m.p.**: 205 – 207°C. **^{1}H NMR** (400 MHz, DMSO-*d6*) δ 12.91 (s, 1H), 8.60 (s, 1H), 7.87 (d, *J* = 1.5 Hz, 1H), 7.82 (s, 2H), 6.65 – 6.63 (m, 1H), 6.19 (s, 2H), 3.71 (s, 12H), 1.91 (s, 3H). **^{13}C NMR** (101 MHz, DMSO-*d6*) δ 170.8, 168.2, 142.8, 141.3, 136.3, 129.6, 128.7, 120.0, 109.4, 54.0, 53.0, 23.3. **HRMS** (ESI): m/z (M + H$^+$) calcd for $C_{21}H_{24}O_{11}N_3S$, 526.1126, found: 526.1120. **IR** (neat): 3220, 2955, 1718, 1601, 1434, 1392, 1265, 1137,1039, 956, 859, 735, 649, 524 cm^{-1}.

Dimethyl 2-(5-(*N*-acetylsulfamoyl)-2-(thiazol-2-yl)-4-(trifluoromethoxy)phenyl)malonate (23)

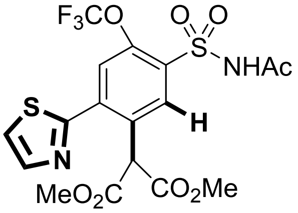

$[RhCp^*Cl_2]_2$ (2.0 mg, 2.5 mol%), AgOAc (12.6 mg, 60 mol%), ***N-((4-(thiazol-2-yl)-2-(trifluoromethoxy)phenyl)sulfonyl)acetamide*** (45.7 mg, 0.125 mmol), **2a** (22 mg, 1.1 equiv), 0.5 mL DCE, 60°C overnight. 54 mg **23** was obtained (87% yield, white powder, EAOAc/ Petroleum ether = 1:1, R_f = 0.3), **m.p.**: 170 – 172°C. **^{1}H NMR** (400 MHz, DMSO-*d6*) δ 12.71 (s, 1H), 8.13 (s, 1H), 8.09 (q, *J* = 3.3 Hz, 2H), 7.87 – 7.85 (m, 1H), 5.93 (s, 1H), 3.70 (s, 6H), 1.97 (s, 3H). **^{13}C NMR** (101 MHz, DMSO-*d6*) δ 169.1, 167.6, 163.0, 144.4, 144.3, 138.5, 134.2, 131.8, 129.9, 124.0, 122.5, 119.8 (q, J = 261.5 Hz), 53.4, 53.1, 23.1. **HRMS** (ESI): m/z (M + H$^+$) calcd for $C_{17}H_{16}O_8N_2F_3S_2$, 497.0295, found: 497.0286. **IR** (neat): 3123, 2954, 1743, 1507, 1367, 1262, 1208, 1161, 997, 860, 760, 644 cm^{-1}.

Dimethyl 2-(4-(*N*-acetylsulfamoyl)-1-(thiazol-2-yl)naphthalen-2-yl)malonate (24)

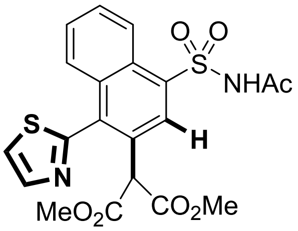

$[RhCp^*Cl_2]_2$ (2.0 mg, 2.5 mol%), AgOAc (12.6 mg, 60 mol%), ***N-((4-(thiazol-2-yl)naphthalen-1-yl)sulfonyl)acetamide*** (41.5 mg, 0.125 mmol), **2a** (22 mg, 1.1 equiv), 0.5 mL DCE, 60°C overnight. 52 mg **24** was obtained (90% yield, white powder, EAOAc/ Petroleum ether = 1:1, R_f = 0.3), **m.p.**: 122 – 123°C. **^{1}H NMR** (400 MHz, $CDCl_3$) δ 9.66 (s, 1H), 8.69 (s, 1H), 8.67 (d, *J* = 8.6 Hz, 1H), 8.16 (d, *J* = 3.3 Hz, 1H), 7.73 (d, *J* = 3.3 Hz, 1H), 7.62 (ddd, *J* = 8.5, 5.5, 2.7 Hz, 1H), 7.54 – 7.51 (m, 2H), 4.75 (s, 1H), 3.74 (s, 6H), 1.98 (s, 3H). **^{13}C NMR** (101 MHz, $CDCl_3$) δ 169.0, 167.7, 162.3, 144.0, 138.0, 135.6, 134.1, 132.2, 130.1, 129.4, 128.2, 127.6, 127.4, 124.2, 122.6, 54.8, 53.5, 23.3. **HRMS** (ESI): m/z (M + H$^+$) calcd for $C_{20}H_{19}O_7N_2S_2$, 463.0628, found:463.0623. **IR** (neat): 3221, 2955, 1741, 1499, 1418, 1357, 1245, 1198, 1154, 991, 865, 745, 649, 535 cm^{-1}.

Dimethyl 2-(5-(*N*-acetylsulfamoyl)-4-methyl-2-(1H-pyrazol-1-yl)phenyl)malonate (25)

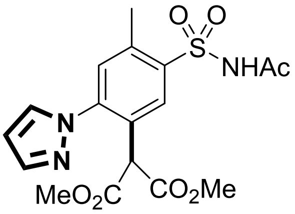

$[RhCp^*Cl_2]_2$ (2.0 mg, 2.5 mol%), AgOAc (12.6 mg, 60 mol%), ***N*-((2-methyl-4-(1H-pyrazol-1-yl)phenyl)sulfonyl)acetamide** (34.9 mg, 0.125 mmol), **2a** (22 mg, 1.1 equiv), 0.5 mL DCE, 60°C overnight. 43.2 mg **25** was obtained (85% yield, white powder, EAOAc/ Petroleum ether = 2:1, R_f = 0.4), **m.p.**: 181 – 183°C. **^{1}H NMR** (400 MHz, DMSO-*d6*) δ 12.34 (s, 1H), 8.28 (d, *J* = 2.5 Hz, 1H), 8.03 (s, 1H), 7.82 (d, *J* = 1.9 Hz, 1H), 7.62 (s, 1H), 6.60 (t, *J* = 2.4 Hz, 1H), 5.22 (s, 1H), 3.67 (s, 6H), 2.64 (s, 3H), 1.96 (s, 3H). **^{13}C NMR** (101 MHz, DMSO-*d6*) δ 168.7, 167.6, 142.0, 141.9, 138.6, 136.3, 132.7, 131.8, 128.3, 124.6, 108.0, 52.9, 52.4, 23.2, 19.1. **HRMS** (ESI): m/z (M + H^+) calcd for $C_{17}H_{20}O_7N_3S$, 410.1016, found: 410.1007. **IR** (neat): 3241, 2953, 1737, 1435, 1332, 1269, 1225, 1199, 1179, 995, 859, 764, 610, 519 cm^{-1}.

Dimethyl 2-(5-(*N*-acetylsulfamoyl)-3-methyl-2-(1H-pyrazol-1-yl)phenyl)malonate (26)

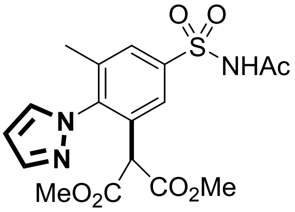

$[RhCp^*Cl_2]_2$ (2.0 mg, 2.5 mol%), AgOAc (12.6 mg, 60 mol%), ***N*-((3-methyl-4-(1H-pyrazol-1-yl)phenyl)sulfonyl)acetamide** (34.7 mg, 0.125 mmol), **2a** (22 mg, 1.1 equiv), 0.5 mL DCE, 60°C overnight. 45.2 mg **26** was obtained (88% yield, white powder, EAOAc/ Petroleum ether = 2:1, R_f = 0.4), **m.p.**: 157 – 158°C. **^{1}H NMR** (400 MHz, DMSO-*d6*) δ 12.28 (s, 1H), 8.10 – 7.80 (m, 4H), 6.59 (s, 1H), 4.24 (s, 1H), 3.66 (s, 6H), 2.07 (s, 3H), 1.97 (s, 3H). **^{13}C NMR** (101 MHz, DMSO-*d6*) δ 169.0, 167.1, 142.7, 141.4, 139.8, 137.6, 132.8, 131.9, 129.3, 126.3, 107.1, 53.1, 51.9, 23.3, 17.3. **HRMS** (ESI): m/z (M + H^+) calcd for $C_{17}H_{20}O_7N_3S$, 410.1016, found: 410.1013. **IR** (neat): 3332, 2956, 1747, 1683, 1635, 1518, 1436, 1299, 1140, 1019, 898, 852, 761, 655 cm^{-1}.

Dibenzyl 2-(2-(*N*-acetylsulfamoyl)-3-methyl-5-(1H-pyrazol-1-yl)phenyl)malonate (27)

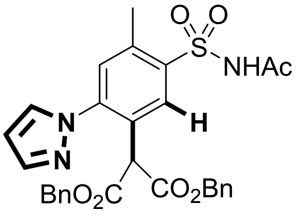

$[RhCp^*Cl_2]_2$ (2.0 mg, 2.5 mol%), AgOAc (12.6 mg, 60 mol%), ***N*-((2-methyl-4-(1H-pyrazol-1-yl)phenyl)sulfonyl)acetamide** (34.9 mg, 0.125 mmol), **2b** (43 mg, 1.1 equiv), 0.5 mL DCE, 60°C overnight. 64 mg **27** was obtained (91% yield, white powder, EAOAc/ Petroleum ether = 2:1, R_f = 0.4), **m.p.**: 175 – 177°C. **^{1}H NMR** (400 MHz, DMSO-*d6*) δ 12.36 (s, 1H), 8.26 (d, *J* = 2.5 Hz, 1H), 8.17 (s, 1H), 7.73 (d, *J* = 1.7 Hz, 1H), 7.64 (s, 1H), 7.36 – 7.27 (m, 10H), 6.59 – 6.57 (m, 1H), 5.34 (s, 1H), 5.21 – 5.12 (m, 4H), 2.65 (s, 3H), 1.97 (s, 3H). **^{13}C NMR** (101 MHz, DMSO-*d6*) δ 168.7, 167.0, 142.0, 141.9, 138.7, 136.3, 135.3, 132.8, 131.7, 128.4, 128.2, 128.2, 127.9, 124.4, 108.0, 67.1, 52.8, 23.2, 19.2. **HRMS** (ESI): m/z (M + H^+) calcd for $C_{29}H_{28}O_7N_3S$, 562.1642, found: 562.1649. **IR** (neat): 3234, 2955, 1733, 1522, 1455, 1376, 1335, 1216, 1157, 994, 854, 751, 618, 519 cm^{-1}.

Dimethyl 2-(5-(*N*-acetylsulfamoyl)-3-methyl-2-(pyridin-2-yl)phenyl)malonate (28)

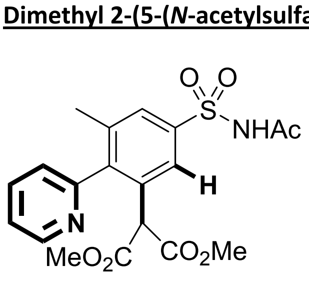

$[RhCp^*Cl_2]_2$ (2.0 mg, 2.5 mol%), AgOAc (12.6 mg, 60 mol%), ***N*-((3-**

methyl-4-(pyridin-2-yl)phenyl)sulfonyl)acetamide (36.2 mg, 0.125 mmol), **2a** (22 mg, 1.1 equiv), 0.5 mL DCE, 60°C overnight. 47.2 mg **28** was obtained (90% yield, white powder, EAOAc/ Petroleum ether = 2:1, $\boldsymbol{R}_f$ = 0.4),**m.p.**: 189 – 191°C. **^{1}H NMR** (400 MHz, DMSO-*d6*) δ 12.18 (s, 1H), 8.71 (d, *J* = 4.6 Hz, 1H), 7.97 (td, *J* = 7.7, 1.7 Hz, 1H), 7.86 (d, *J* = 1.5 Hz, 1H), 7.82 (d, *J* = 1.7 Hz, 1H), 7.51 – 7.47 (m, 1H), 7.40 (d, *J* = 7.8 Hz, 1H), 4.41 (s, 1H), 3.63 (s, 6H), 2.10 (s, 3H), 1.96 (s, 3H). **^{13}C NMR** (101 MHz, DMSO-*d6*) δ 168.9, 167.5, 155.5, 145.0, 145.2, 139.0, 137.6, 137.1, 132.2, 128.3, 125.4, 124.8, 123.3, 54.1, 52.9, 23.3, 20.2. **HRMS** (ESI): m/z (M + H$^+$) calcd for $C_{19}H_{21}O_7N_2S$, 421.1064, found: 421.1053. **IR** (neat): 3249, 2955, 1732, 1586, 1433, 1339, 1264, 1197, 1180, 996, 844, 734, 628, 513 cm^{-1}.

Dimethyl 2-(5-(*N*-acetylsulfamoyl)-3-methyl-2-(pyrimidin-2-yl)phenyl)malonate (29)

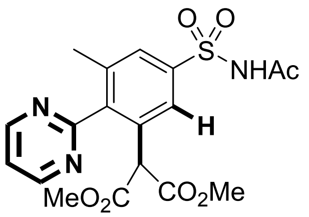

$[RhCp^*Cl_2]_2$ (2.0 mg, 2.5 mol%), AgOAc (12.6 mg, 60 mol%), ***N-((3-methyl-4-(pyrimidin-2-yl)phenyl)sulfonyl)acetamide*** (36.3 mg, 0.125 mmol), **2a** (22 mg, 1.1 equiv), 0.5 mL DCE, 60°C overnight. 47 mg **29** was obtained (89% yield, white powder, EAOAc/ Petroleum ether = 2:1, $\boldsymbol{R}_f$ = 0.3), **m.p.**: 183 – 185°C. **^{1}H NMR** (400 MHz, DMSO-*d6*) δ 12.20 (s, 1H), 9.00 (s, 1H), 8.99 (s, 1H), 7.88 – 7.86 (m, 1H), 7.80 (d, *J* = 1.8 Hz, 1H), 7.61 (t, *J* = 5.0 Hz, 1H), 4.48 (s, 1H), 3.64 (s, 6H), 2.15 (s, 3H), 1.96 (s, 3H). **^{13}C NMR** (101 MHz, DMSO-*d6*) δ 168.9, 167.5, 164.1, 157.8, 143.4, 139.3, 137.6, 132.1, 128.5, 125.7, 120.6, 54.2, 52.9, 23.3, 19.9. **HRMS** (ESI): m/z (M + H$^+$) calcd for $C_{18}H_{20}O_7N_3S$, 422.1016, found: 422.1005. **IR** (neat): 3239, 2955, 1735, 1560, 1435, 1412, 1347, 1258, 1154, 994, 909, 733, 645, 595, 513 cm^{-1}.

Dimethyl 2-(5-(*N*-acetylsulfamoyl)-2-(pyridin-2-yl)thiophen-3-yl)malonate (30)

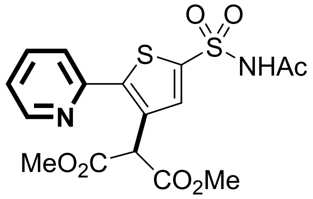

$[RhCp^*Cl_2]_2$ (2.0 mg, 2.5 mol%), AgOAc (12.6 mg, 60 mol%), ***N-((5-(pyridin-2-yl)thiophen-2-yl)sulfonyl)acetamide*** (35.2 mg, 0.125 mmol), **2a** (22 mg, 1.1 equiv), 0.5 mL DCE, 60°C overnight. 46 mg **30** was obtained (90% yield, white powder, EAOAc/ Petroleum ether = 2:1, $\boldsymbol{R}_f$ = 0.3), **m.p.**: 184 – 186°C. **^{1}H NMR** (400 MHz, $CDCl_3$) δ 9.73 (s, 1H), 8.71 (d, *J* = 4.4 Hz, 1H), 8.01 (s, 1H), 7.86 (t, *J* = 7.7 Hz, 1H), 7.68 (d, *J* = 7.8 Hz, 1H), 7.39 (dd, *J* = 7.5, 4.9 Hz, 1H), 5.59 (s, 1H), 3.77 (s, 6H), 2.03 (s, 3H). **^{13}C NMR** (101 MHz, $CDCl_3$) δ 168.5, 167.9, 150.2, 149.7, 147.4, 138.5, 138.4, 137.4, 130.5, 124.3, 124.0, 53.5, 51.6, 23.6. **HRMS** (ESI): m/z (M + H$^+$) calcd for $C_{16}H_{17}O_7N_2S_2$, 413.0472, found: 413.0461. **IR** (neat): 3243, 3107, 2955, 1733, 1580, 1447, 1297, 1208, 1163, 996, 801, 737, 661, 409 cm^{-1}.

Intermolecular competition experiments between X-type sulfonamide and L-type *N*-heterocycles

Method A: $[RhCp^*Cl_2]_2$ (3.9 mg, 5.0 mol%), AgOAc (4.2 mg, 20 mol%), sulfonamide (0.125 mmol), heterocycles (0.125 mmol), **2a** (22 mg, 1.1 equiv), 25 mL toluene, 60°C overnight.

Method B: $[RhCp^*Cl_2]_2$ (2.0 mg, 2.5 mol%), AgOAc (12.6 mg, 60 mol%), sulfonamide (0.125 mmol), heterocycles (0.125 mmol), **2a** (22 mg, 1.1 equiv), 0.5 mL DCE, 60°C overnight.

Dimethyl 2-(2-(N-acetylsulfamoyl)-3-methylphenyl)malonate (31)

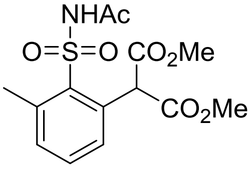

Scheme 2A, *Method A*: 28 mg **31** (66% yield); ***Scheme 2B***, *Method A*: 29 mg **31** (68% yield); ***Scheme 2C***, *Method A*: 34 mg **31** (79% yield). (white powder, EAOAc/ Petroleum ether = 1:1, $\mathbf{R_f}$ = 0.4), m.p.: 159 – 161°C.^{1}**H NMR** (400 MHz, DMSO-*d6*) δ 7.55 (t, *J* = 7.7 Hz, 1H), 7.41 (d, *J* = 7.4 Hz, 1H), 7.19 (d, *J* = 7.7 Hz, 1H), 6.10 (s, 1H), 3.67 (s, 6H), 2.66 (s, 3H), 1.93 (s, 3H). ^{13}**C NMR** (101 MHz, DMSO-*d6*) δ 169.9, 168.8, 140.1, 136.4, 134.2, 133.2, 132.8, 128.7, 54.2, 52.7, 23.1, 22.2. **HRMS** (ESI): m/z (M + H$^+$) calcd for $C_{14}H_{18}O_7NS$, 344.0798, found: 344.0791. **IR** (neat): 3245, 2955, 1734, 1435, 1348, 1265, 1229, 1151, 995, 903, 845, 734, 639, 600, 507 cm^{-1}.

Dimethyl 2-(3-methyl-2-(pyridin-2-yl)phenyl)malonate (32)

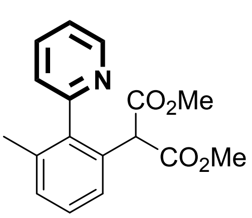

Method B: 25 mg **32** (68% yield, oil, EAOAc/ Petroleum ether = 1:2, $\mathbf{R_f}$ = 0.4). ^{1}**H NMR** (400 MHz, DMSO-*d6*) δ 8.68 (d, *J* = 4.8 Hz, 1H), 7.91 (td, *J* = 7.7, 1.7 Hz, 1H), 7.43 – 7.34 (m, 2H), 7.32 – 7.28 (m, 2H), 7.22 (d, *J* = 7.5 Hz, 1H), 4.36 (s, 1H), 3.60 (s, 6H), 2.01 (s, 3H). ^{13}**C NMR** (101 MHz, DMSO-*d6*) δ 168.2, 157.1, 149.7, 140.6, 136.7, 135.8, 131.0, 129.7, 128.1, 126.0, 125.0, 122.6, 54.3, 52.6, 20.1. **HRMS** (ESI): m/z (M + H$^+$) calcd for $C_{14}H_{18}O_4N$, 300.1230, found: 300.1228. **IR** (neat): 3059, 2952, 1717, 1599, 1456, 1433, 1309, 1263, 1238, 1135, 1027, 989, 788, 752, 730, 622, 528 cm^{-1}.

Dimethyl 2-(3-methyl-2-(1H-pyrazol-1-yl)phenyl)malonate (33)

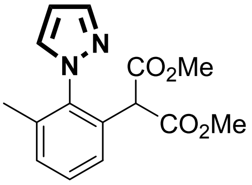

Method B: 24 mg **33** (66% yield, oil, EAOAc/ Petroleum ether = 1:3, $\mathbf{R_f}$ = 0.3). ^{1}**H NMR** (400 MHz, DMSO-*d6*) δ 7.85 (d, *J* = 2.3 Hz, 1H), 7.76 (d, *J* = 1.6 Hz, 1H), 7.48 (t, *J* = 7.7 Hz, 1H), 7.41 (d, *J* = 6.9 Hz, 1H), 7.33 (d, *J* = 7.7 Hz, 1H), 6.53 (t, *J* = 2.1 Hz, 1H), 4.16 (s, 1H), 3.63 (s, 6H), 1.96 (s, 3H). ^{13}**C NMR** (101 MHz, DMSO-*d6*) δ 167.7, 140.6, 138.9, 136.0, 132.5, 130.9, 130.6, 129.2, 126.8, 106.5, 52.8, 52.0, 17.0. **HRMS** (ESI): m/z (M + H$^+$) calcd for $C_{15}H_{17}O_4N_2$, 289.1183, found: 289.1182. **IR** (neat): 3118, 2954, 1734, 1518, 1434, 1392, 1311, 1242, 1146, 1021, 937, 759, 701, 625 cm^{-1}.

Dimethyl 2-(3-methyl-2-(thiazol-2-yl)phenyl)malonate (34)

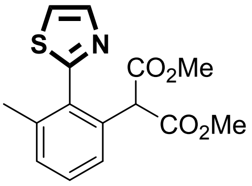

Method B: 22 mg **33** (58% yield, oil, EAOAc/ Petroleum ether = 1:3, $\mathbf{R_f}$ = 0.3). ^{1}**H NMR** (400 MHz, DMSO-*d6*) δ 8.02 (d, *J* = 3.3 Hz, 1H), 7.99 (d, *J* = 3.3 Hz, 1H), 7.46 (t, *J* = 7.7 Hz, 1H), 7.37 (d, *J* = 7.5 Hz, 1H), 7.27 (d, *J* = 7.7 Hz, 1H), 4.46 (s, 1H), 3.63 (s, 6H), 2.09 (s, 3H). ^{13}**C NMR** (101 MHz, DMSO-*d6*) δ 167.9, 163.5, 143.2, 137.7, 133.5, 132.5, 130.0, 129.7, 126.3, 122.6, 54.2, 52.8, 20.1. **HRMS** (ESI): m/z (M + H$^+$) calcd for $C_{15}H_{16}O_4NS$, 306.0795, found: 306.0791. **IR** (neat): 3116, 2952, 1722, 1463, 1433, 1307, 1246, 127, 1121, 1052, 968, 787, 730, 657, 554 cm^{-1}.

Dimethyl 2-(2-(N-acetylsulfamoyl)thiophen-3-yl)malonate (35)

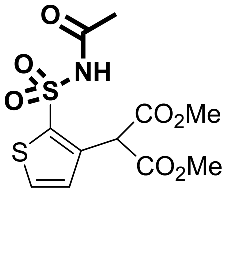

Method A: 23mg **35** was obtained (55% yield, white powder, EAOAc/ Petroleum ether = 2:1, $\mathbf{\mathit{R_f}}$ = 0.4), **m.p.**: 122 – 123°C. ^{1}**H NMR** (400 MHz, DMSO-

d6) δ 7.97 (d, *J* = 5.2 Hz, 1H), 7.15 (d, *J* = 5.2 Hz, 1H), 5.68 (s, 1H), 3.70 (s, 6H), 1.89 (s, 3H). **^{13}C NMR** (101 MHz, DMSO-*d6*) δ 169.6, 167.2, 137.5, 136.5, 132.4, 129.3, 53.1, 50.1, 23.5. **HRMS** (ESI): m/z (M + H$^+$) calcd for $C_{11}H_{14}O_7NS_2$, 336.0206, found: 336.0199. **IR** (neat): 3245, 2955, 1731, 1418, 1337, 1269, 1139, 1017, 917, 851, 762, 653 cm^{-1}.

Dimethyl 2-(2-(pyridin-2-yl)thiophen-3-yl)malonate (36)

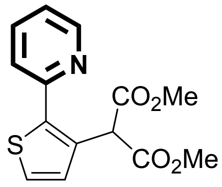

Method B: 27 mg **36** (75% yield, oil, EAOAc/ Petroleum ether = 1:3, **R_f** = 0.4). **^{1}H NMR** (400 MHz, DMSO-*d6***)** δ 8.62 – 8.59 (m, 1H), 7.87 (td, *J* = 7.8, 1.8 Hz, 1H), 7.66 – 7.63 (m, 2H), 7.33 (dd, *J* = 7.5, 4.9 Hz, 1H), 7.13 (d, *J* = 5.3 Hz, 1H), 5.97 (s, 1H), 3.69 (s, 6H). **^{13}C NMR** (101 MHz, DMSO-*d6*) δ 168.3, 151.7, 149.3, 139.2, 137.7, 130.5, 130.0, 126.3, 122.5, 122.1, 52.7, 51.3. **HRMS** (ESI): m/z (M + H$^+$) calcd for $C_{14}H_{14}O_4NS$, 292.0638, found: 292.0635. **IR** (neat): 3104, 2951, 1718, 1584, 1470, 1435, 1293, 1249, 1193, 1145, 1026, 977, 783, 714, 669, 404 cm^{-1}.

Compound 3’

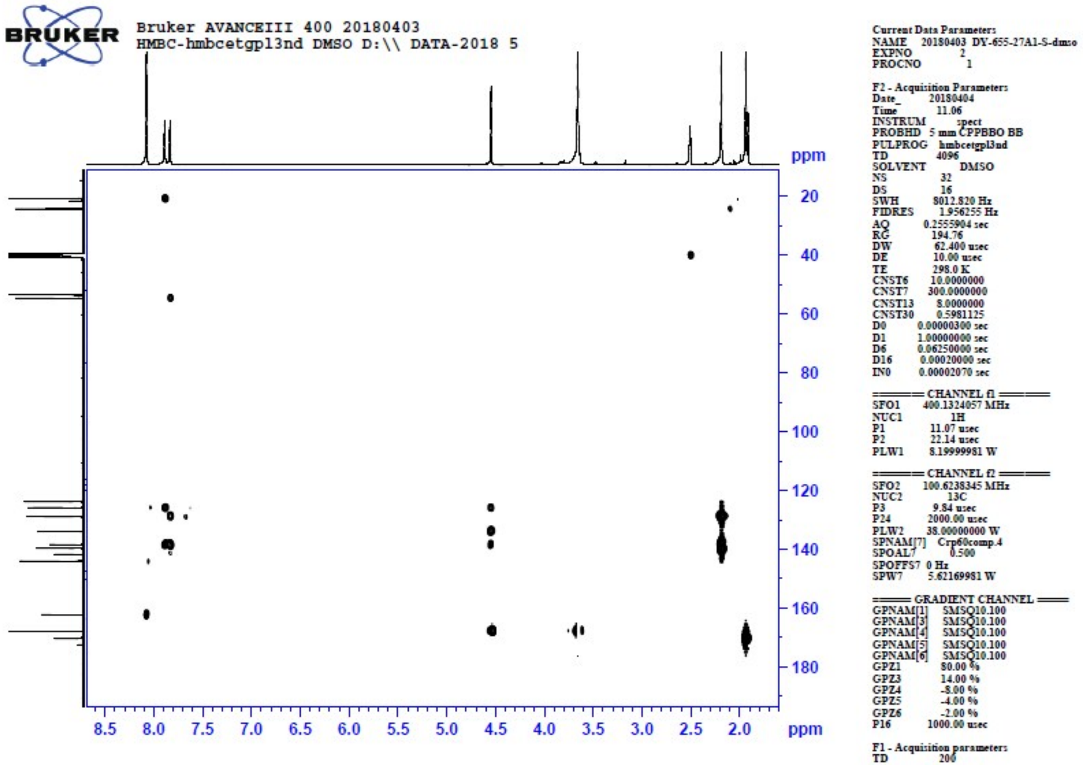

Compound 3’

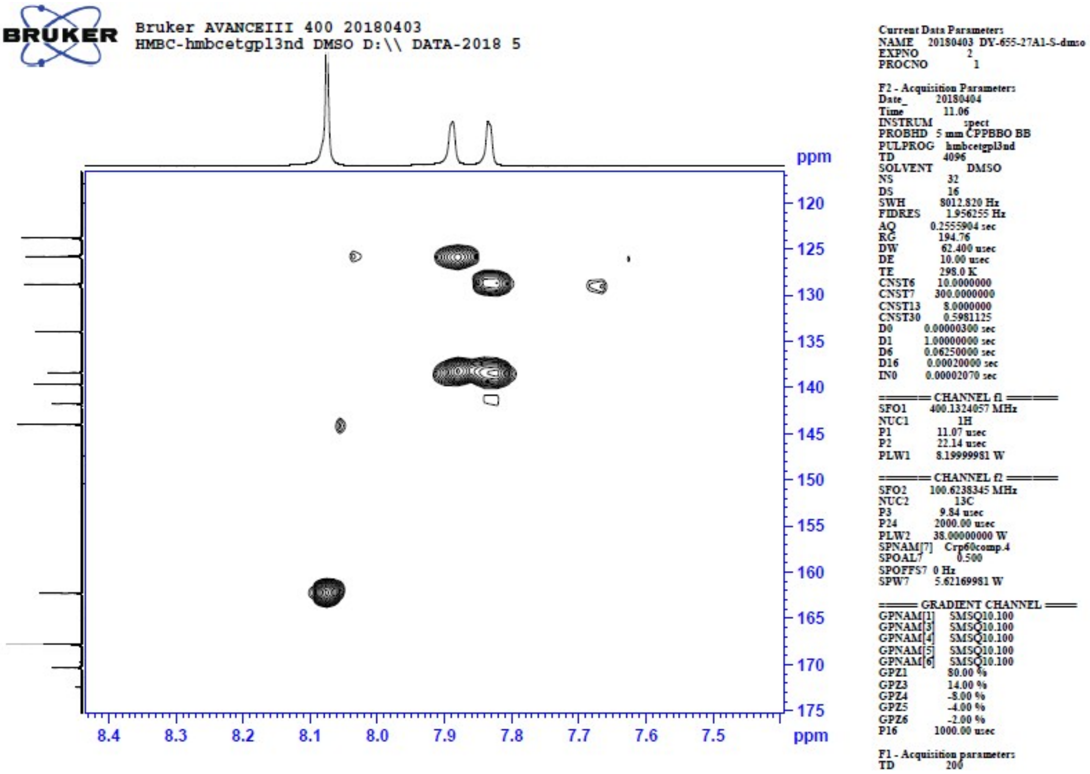

Compound 3’

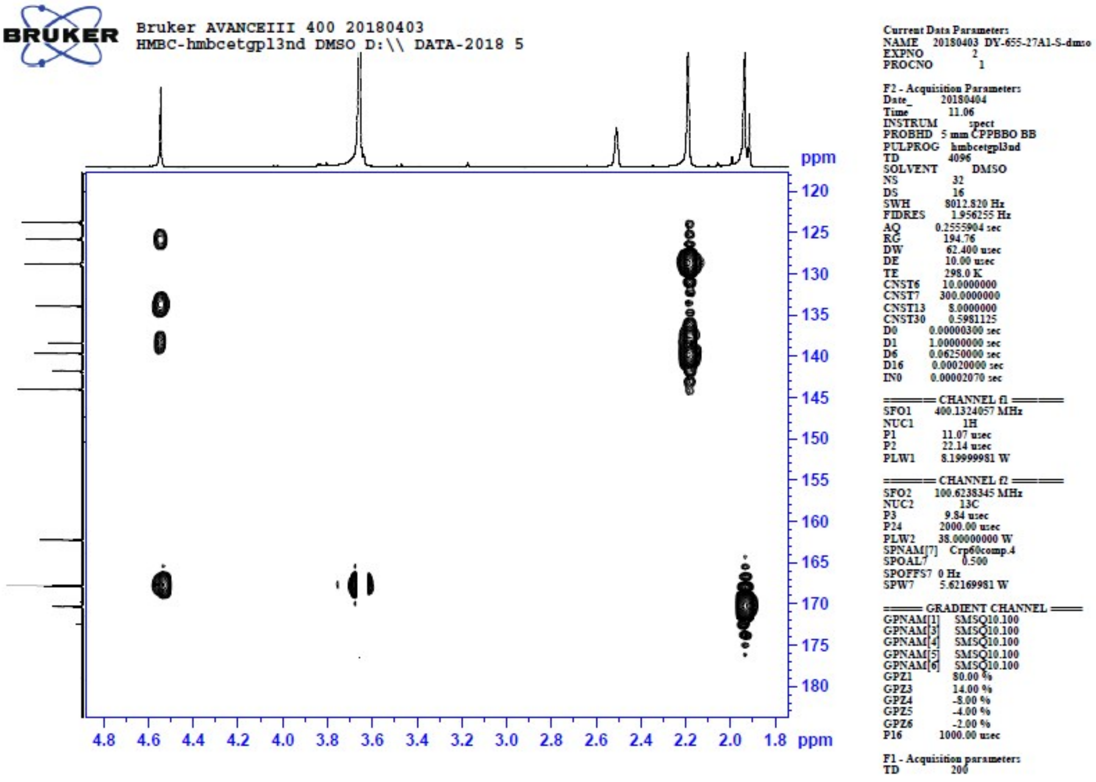

Compound 3’

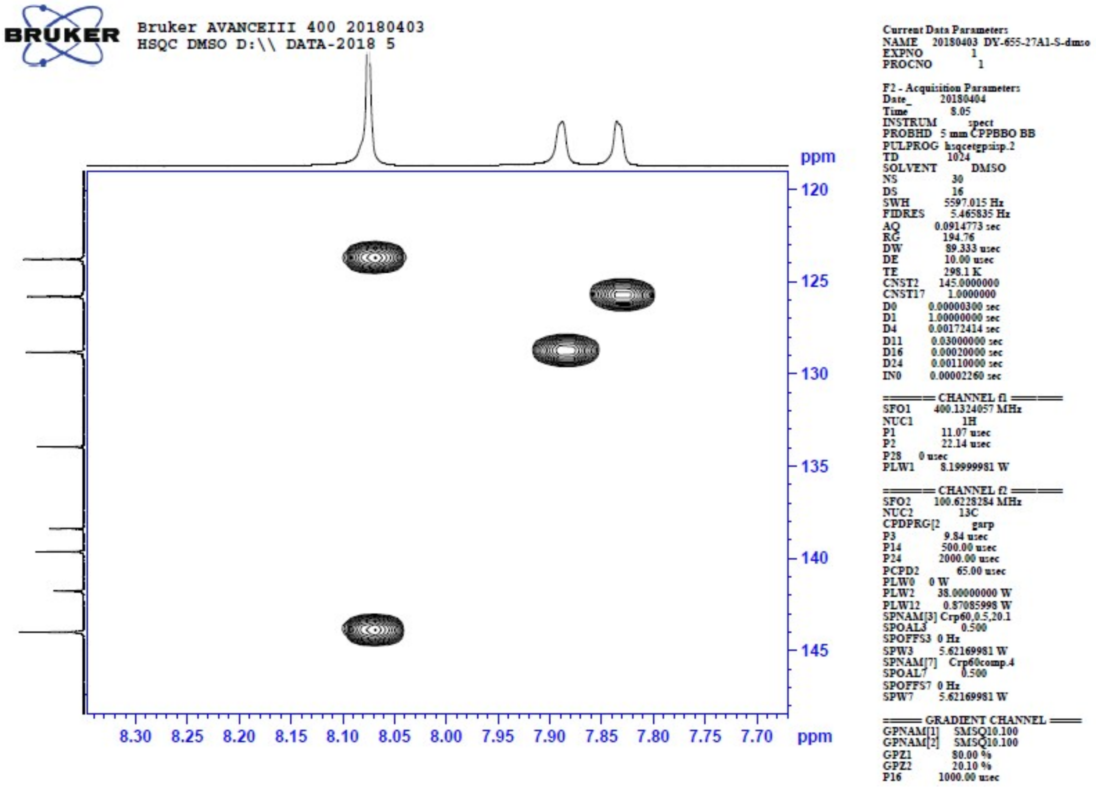

Compound 3’

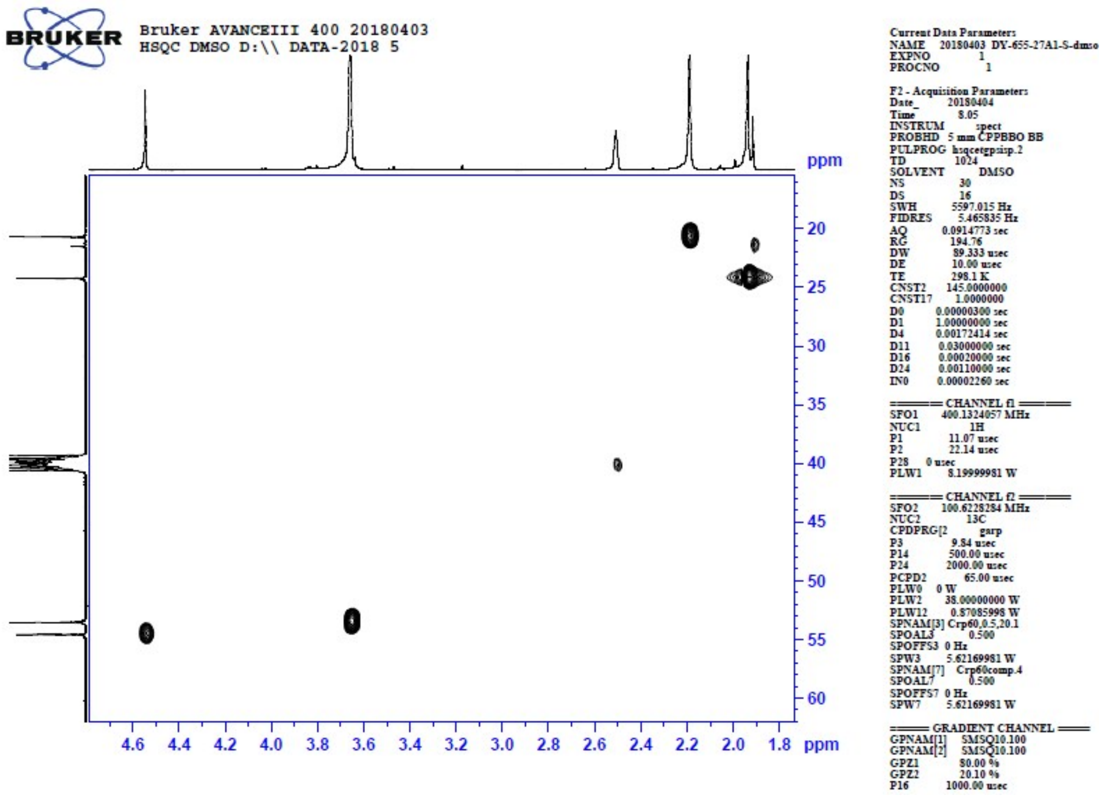

Compound 3’

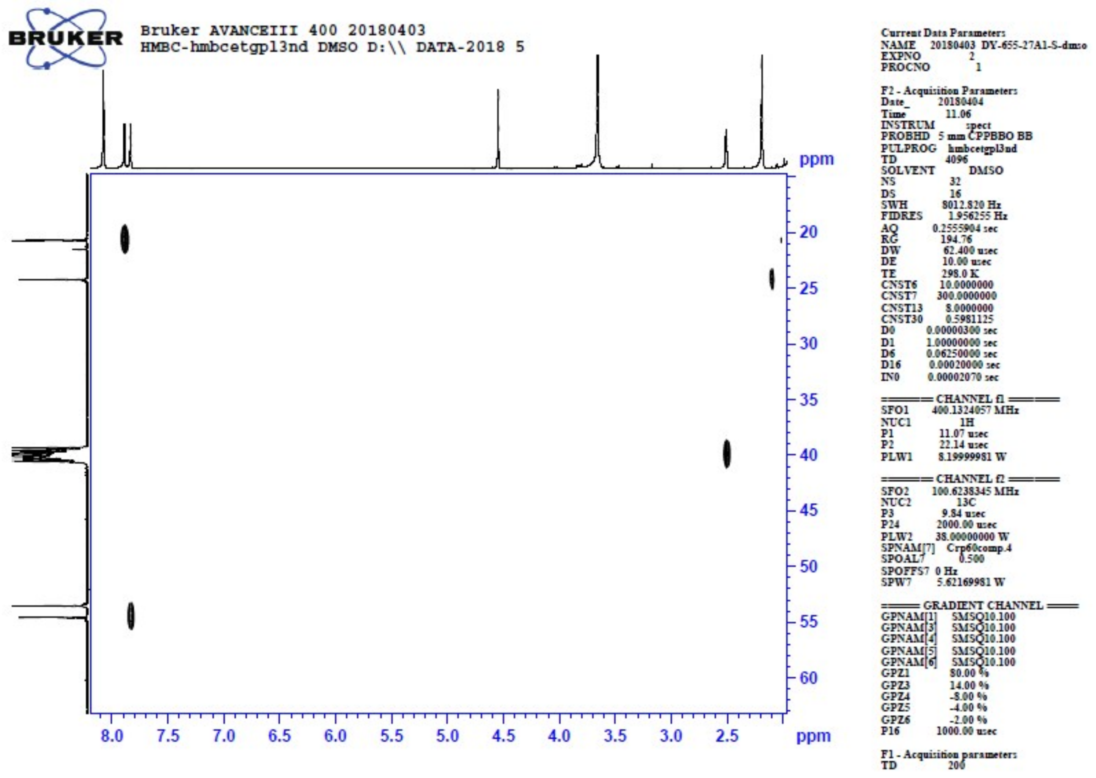

Compound 3'

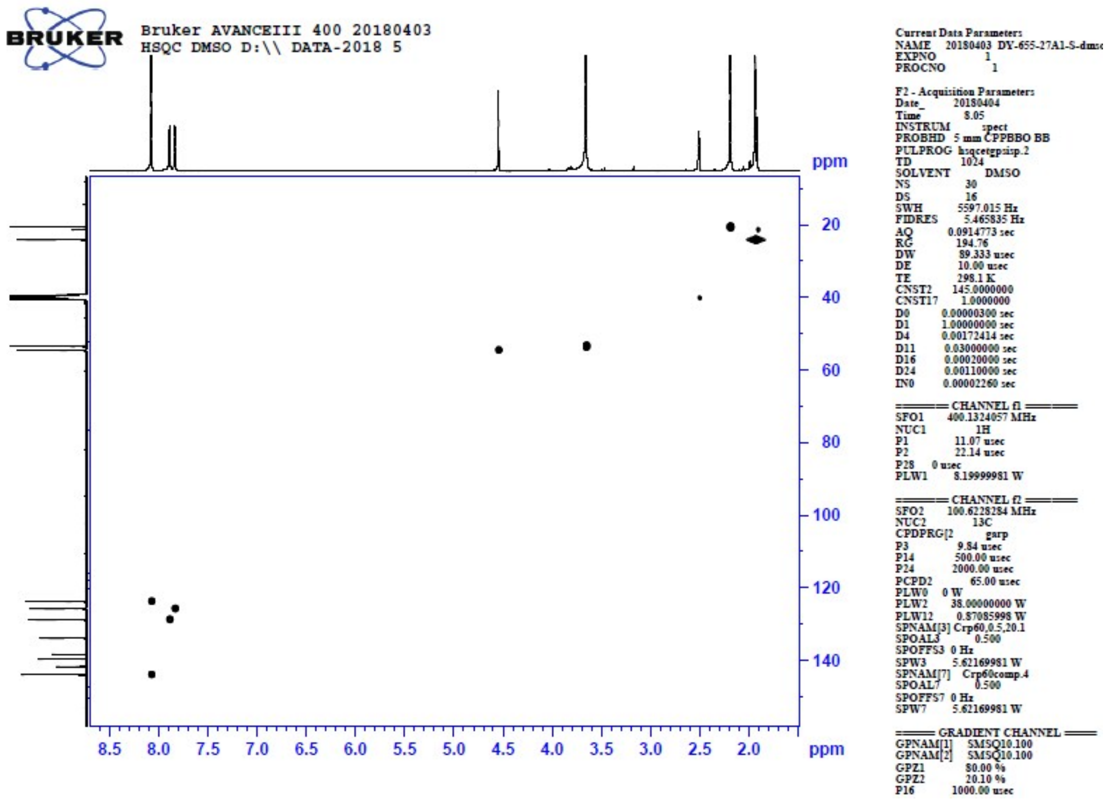

Compound 3

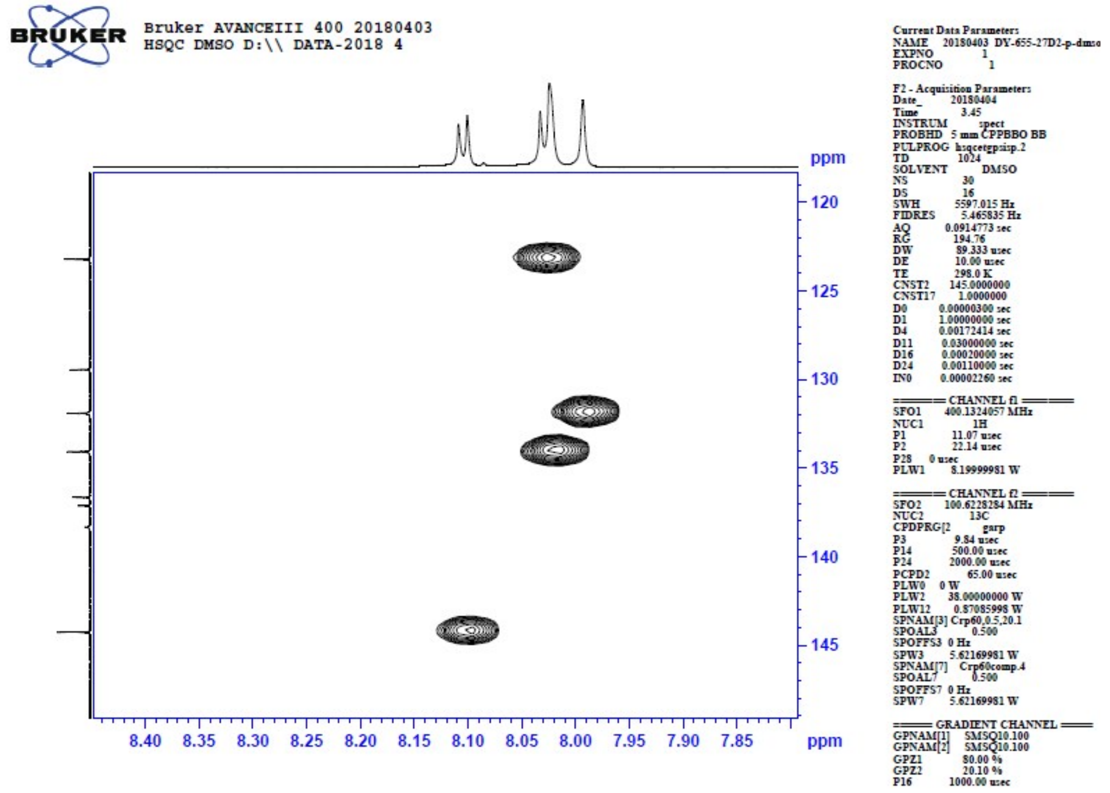

Compound 3

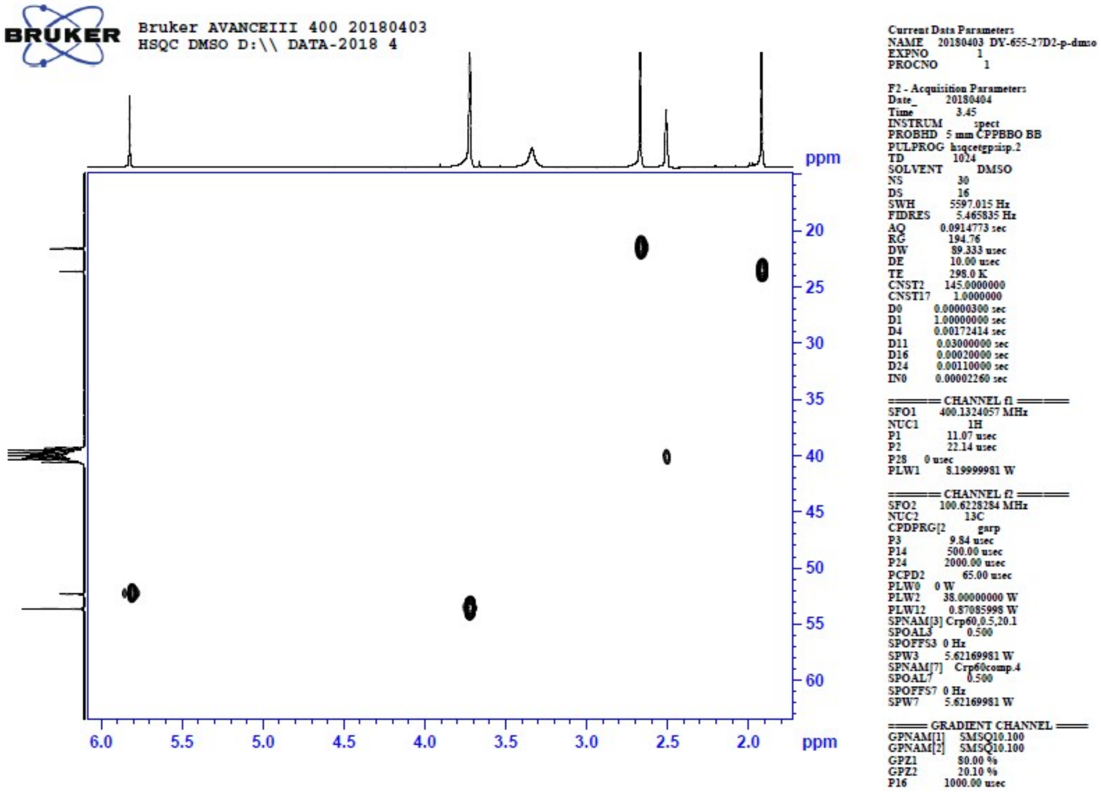

Compound 3

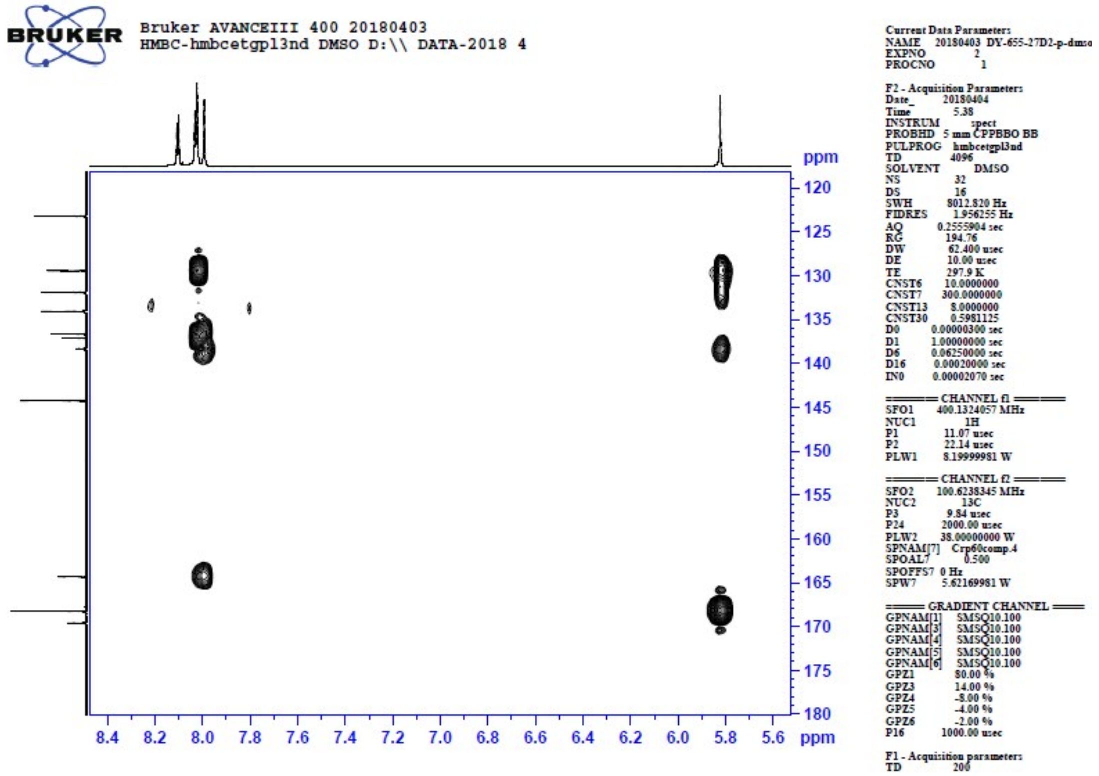

Compound 3

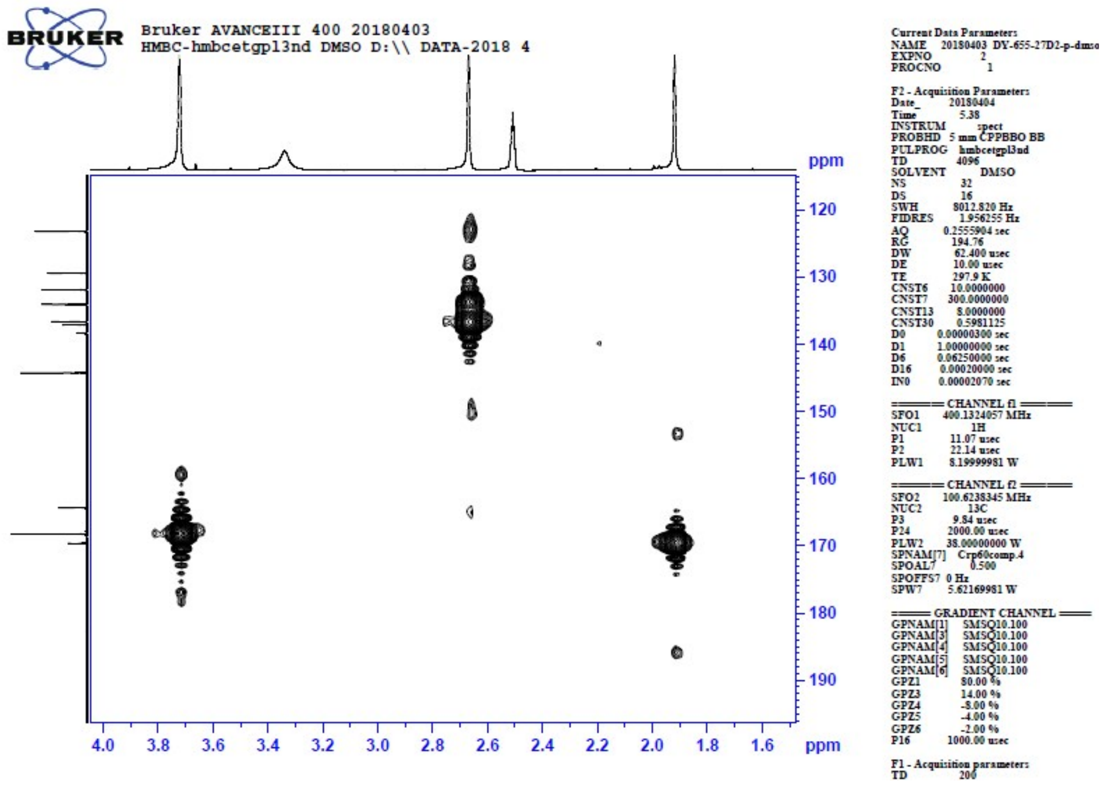

Compound 3

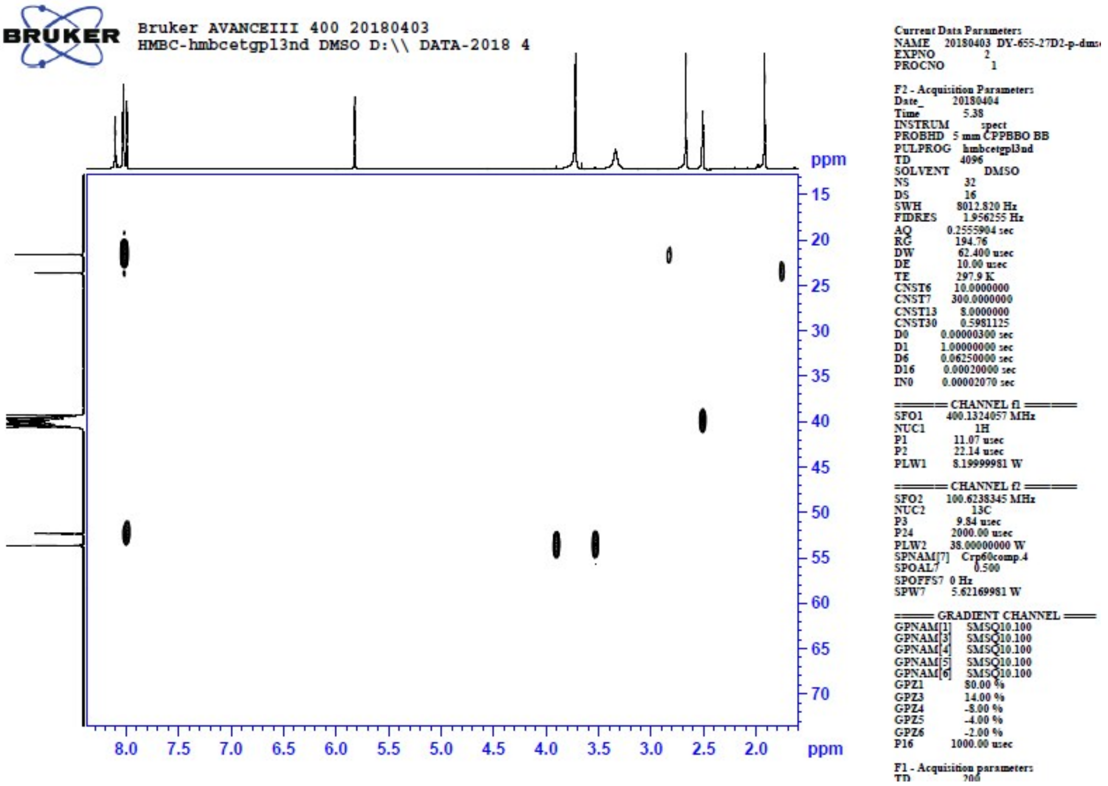

Compound 3

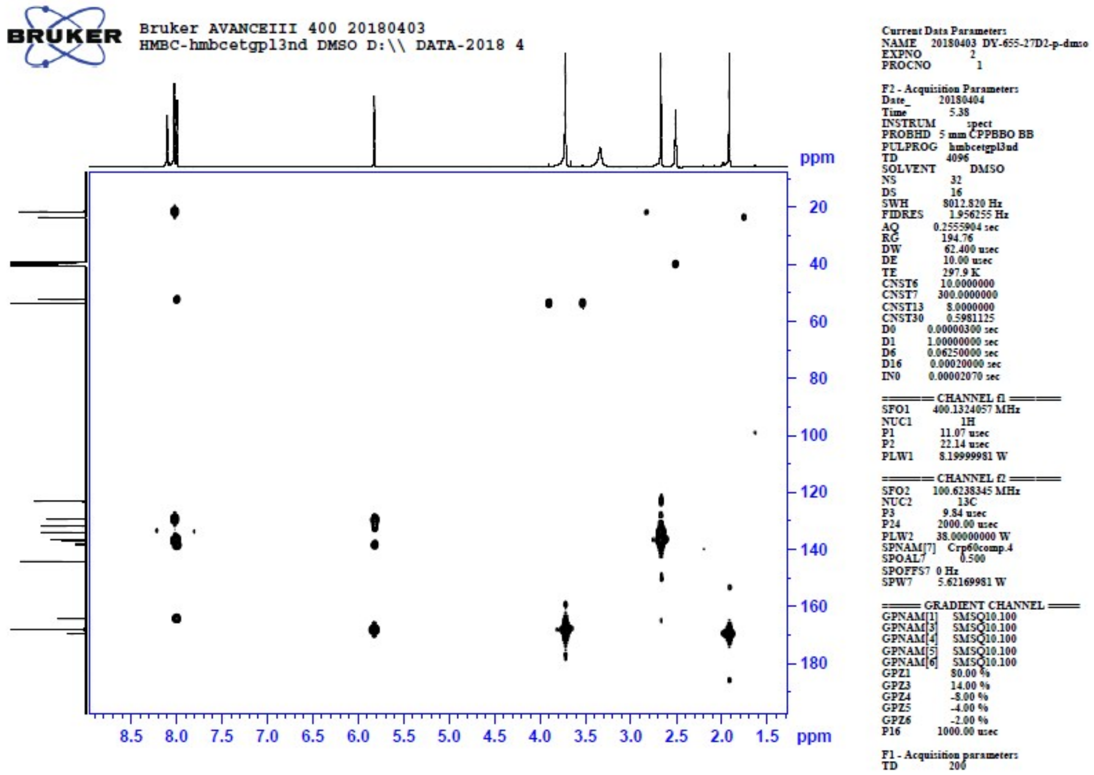

Compound 3

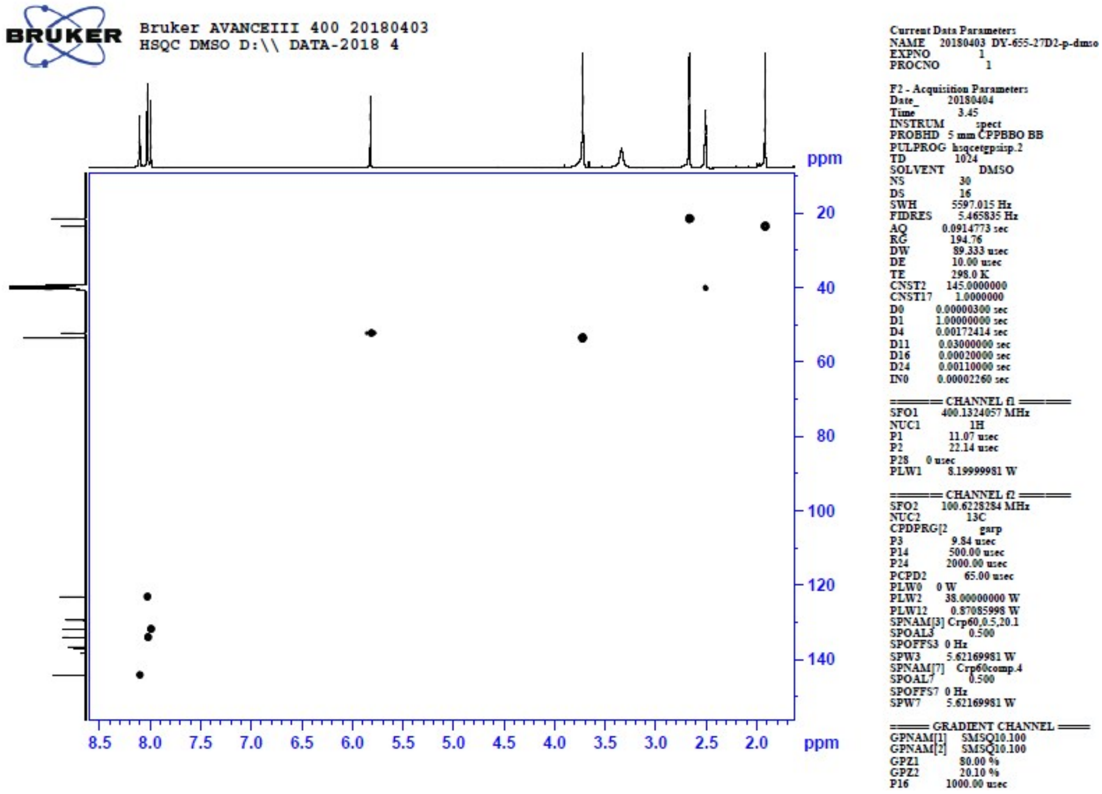

^{1}H NMR of compound **3**

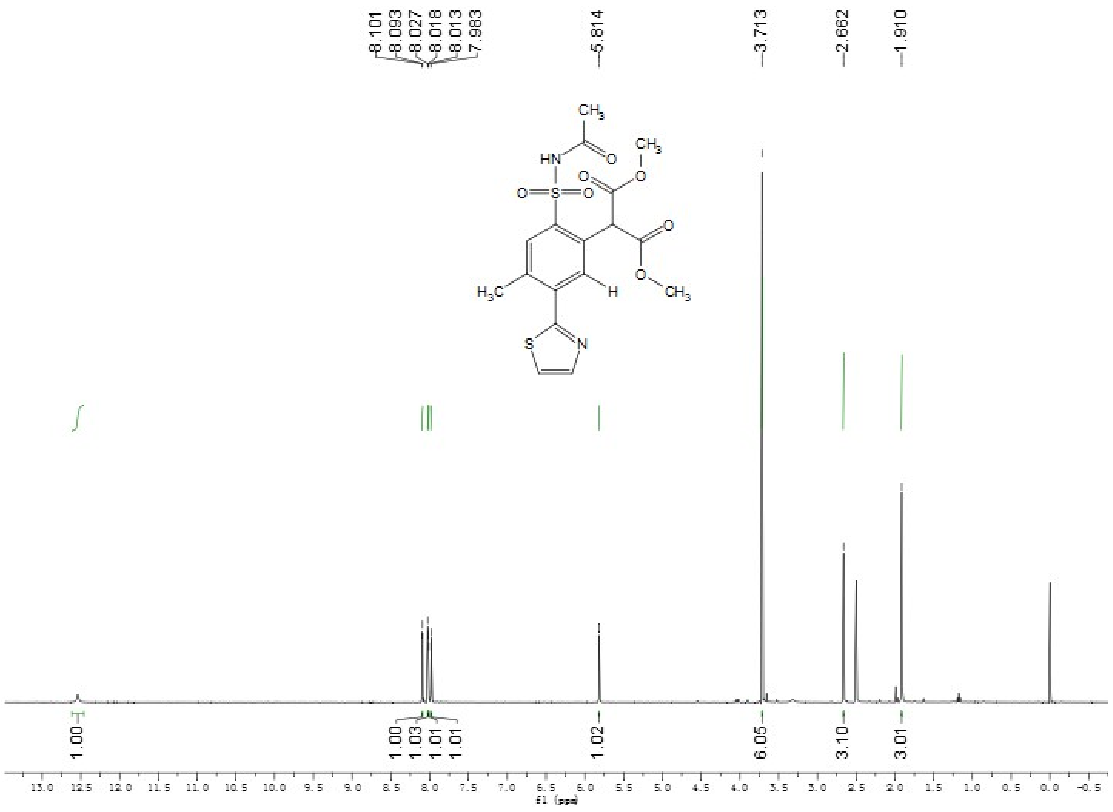

^{13}C NMR of compound **3**

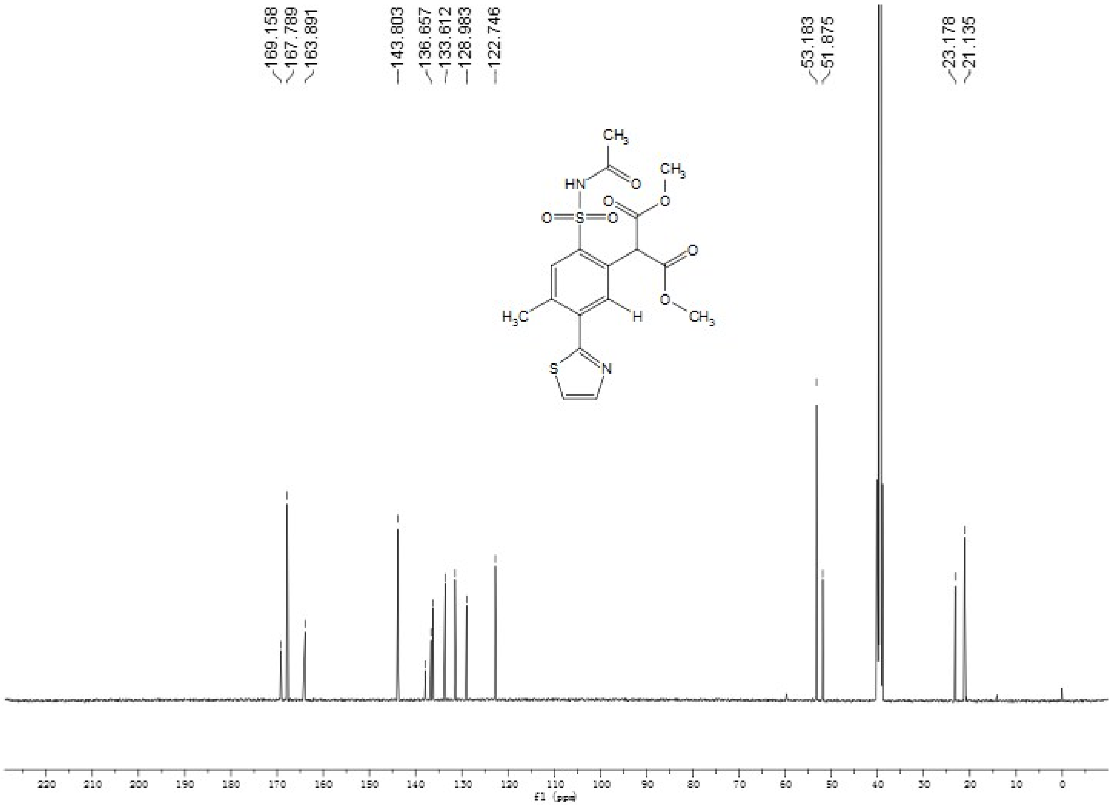

^{1}H NMR of compound **3’**

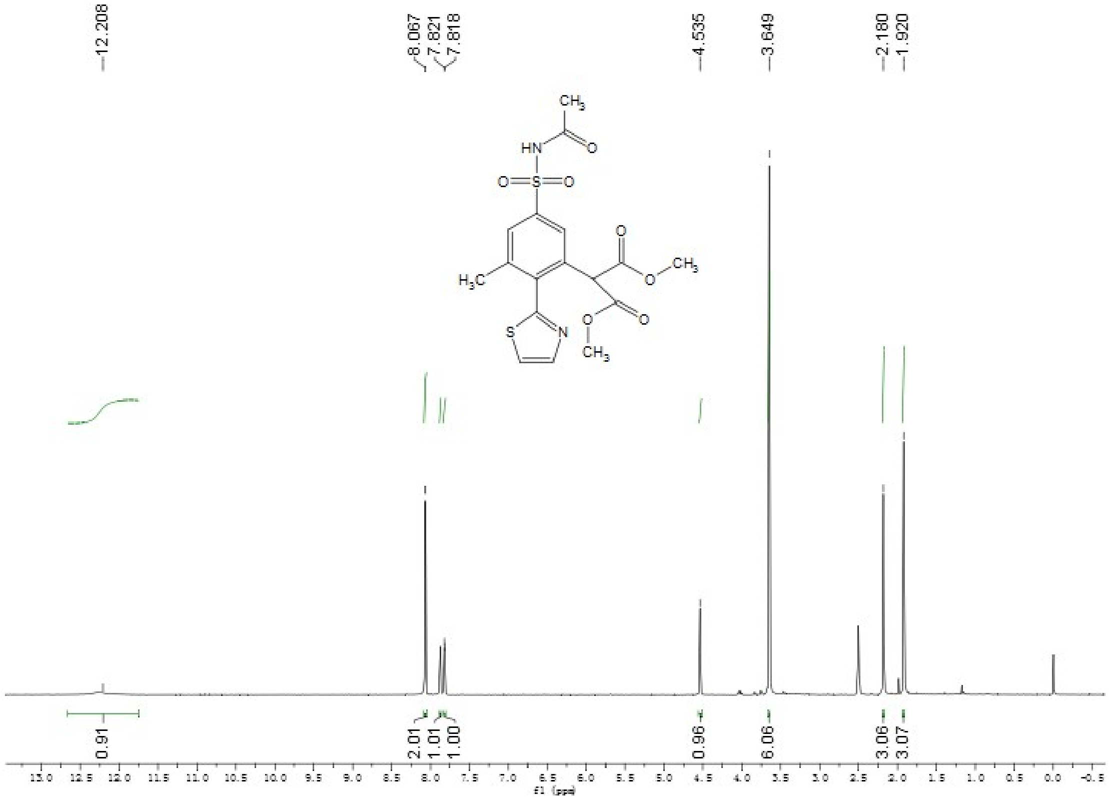

^{13}C NMR of compound **3’**

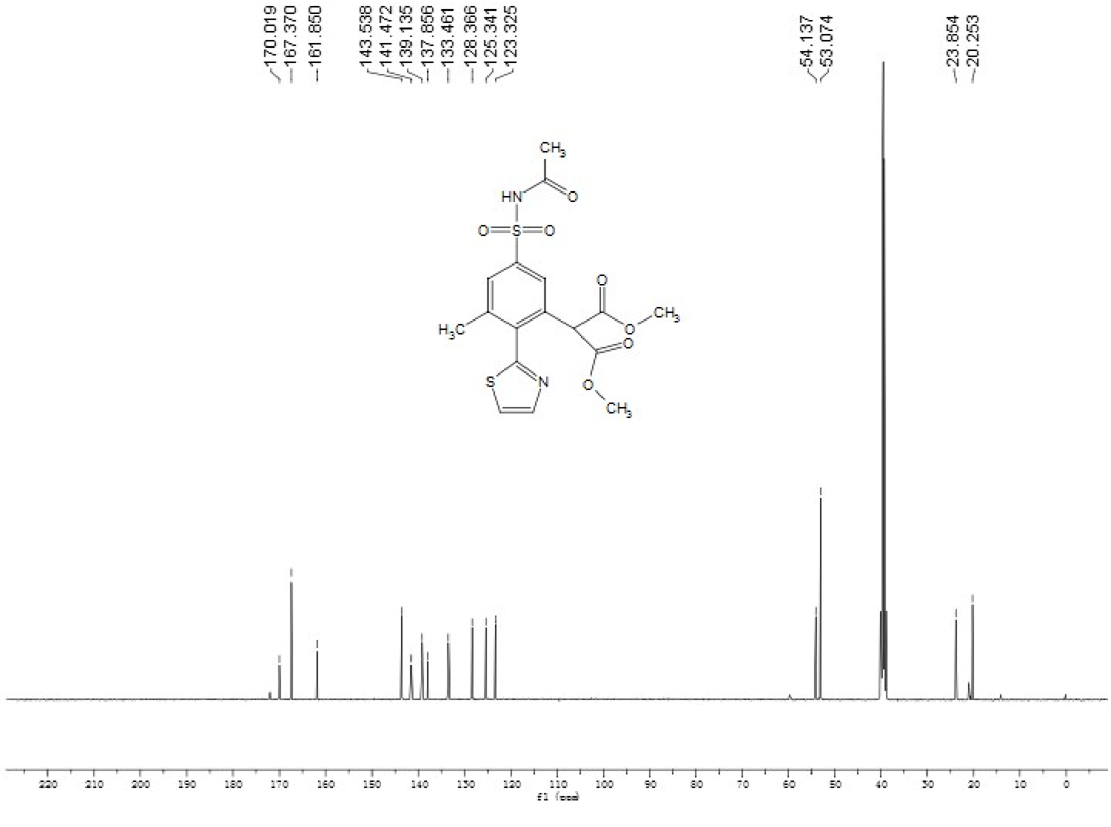

^{1}H NMR of compound **4**

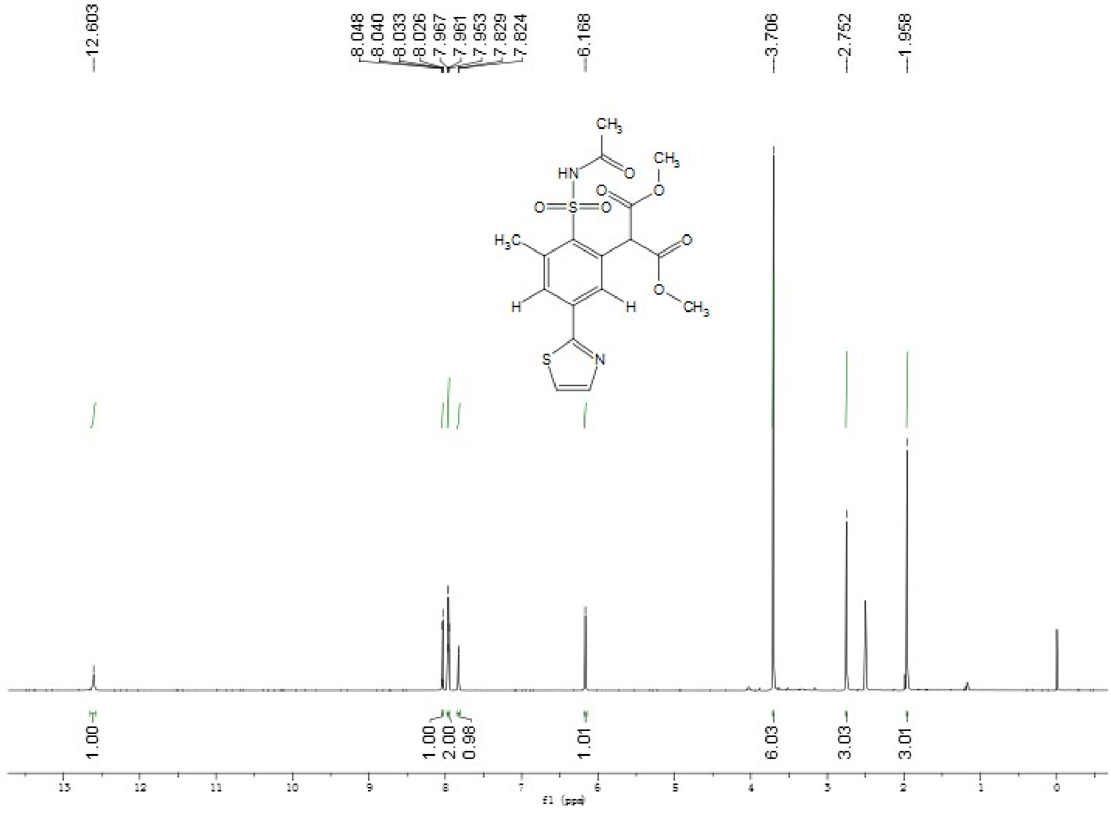

^{13}C NMR of compound **4**

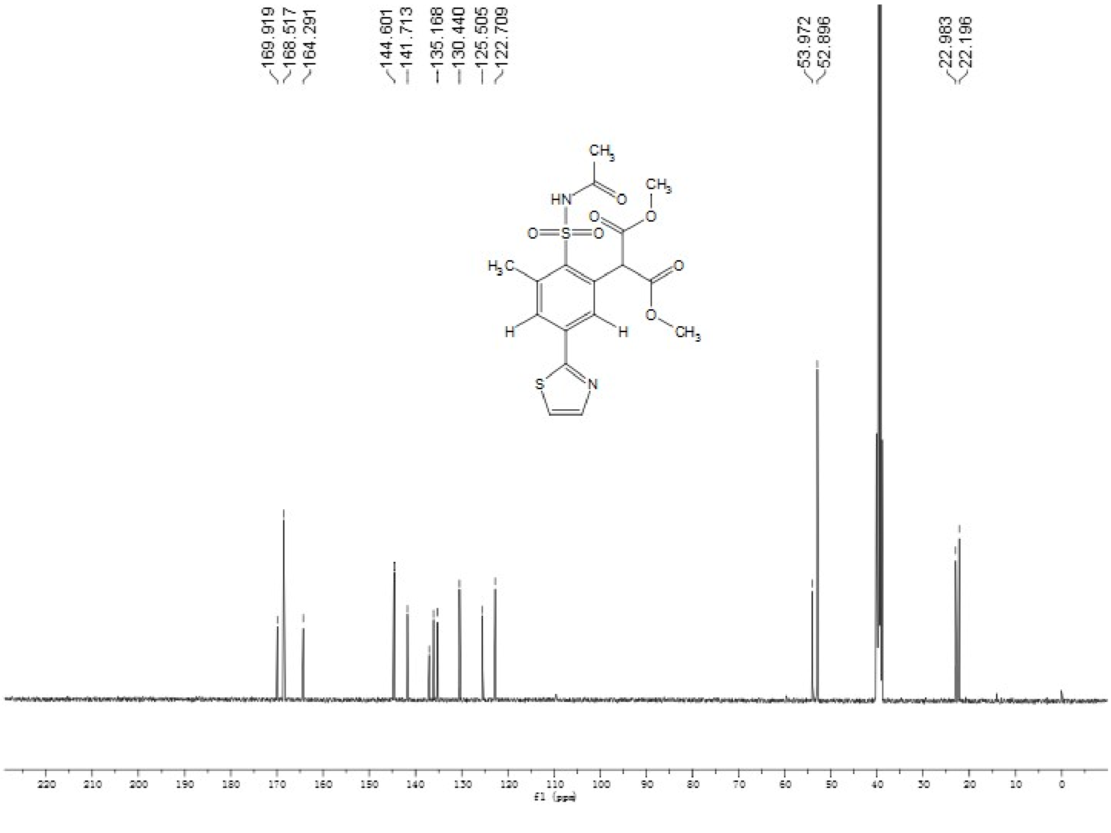

^{1}H NMR of compound **5**

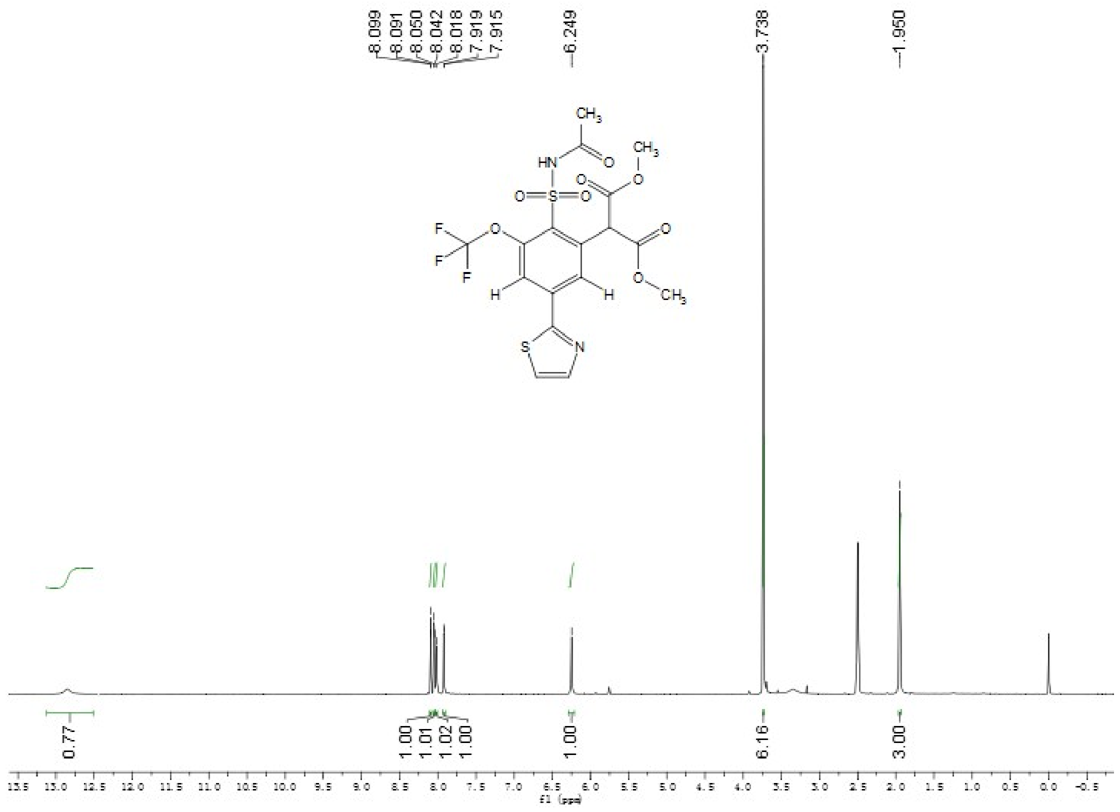

^{13}C NMR of compound **5**

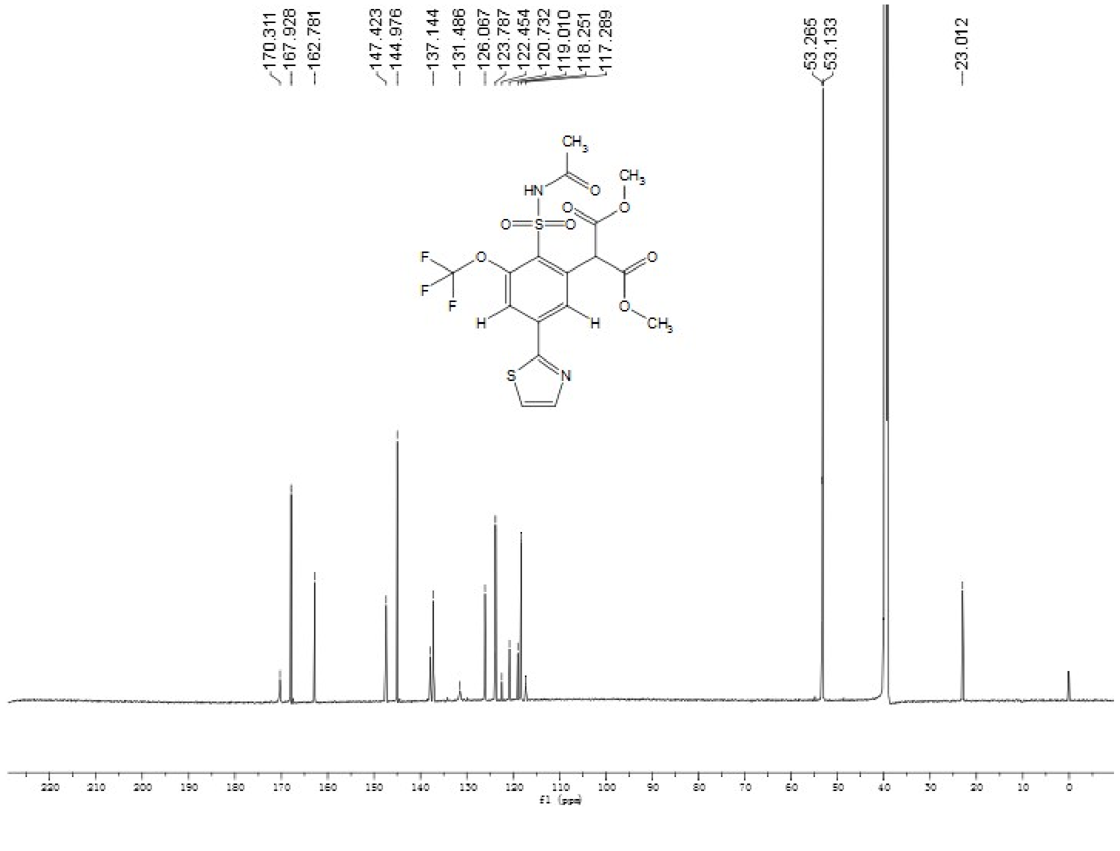

^{1}H NMR of compound **6**

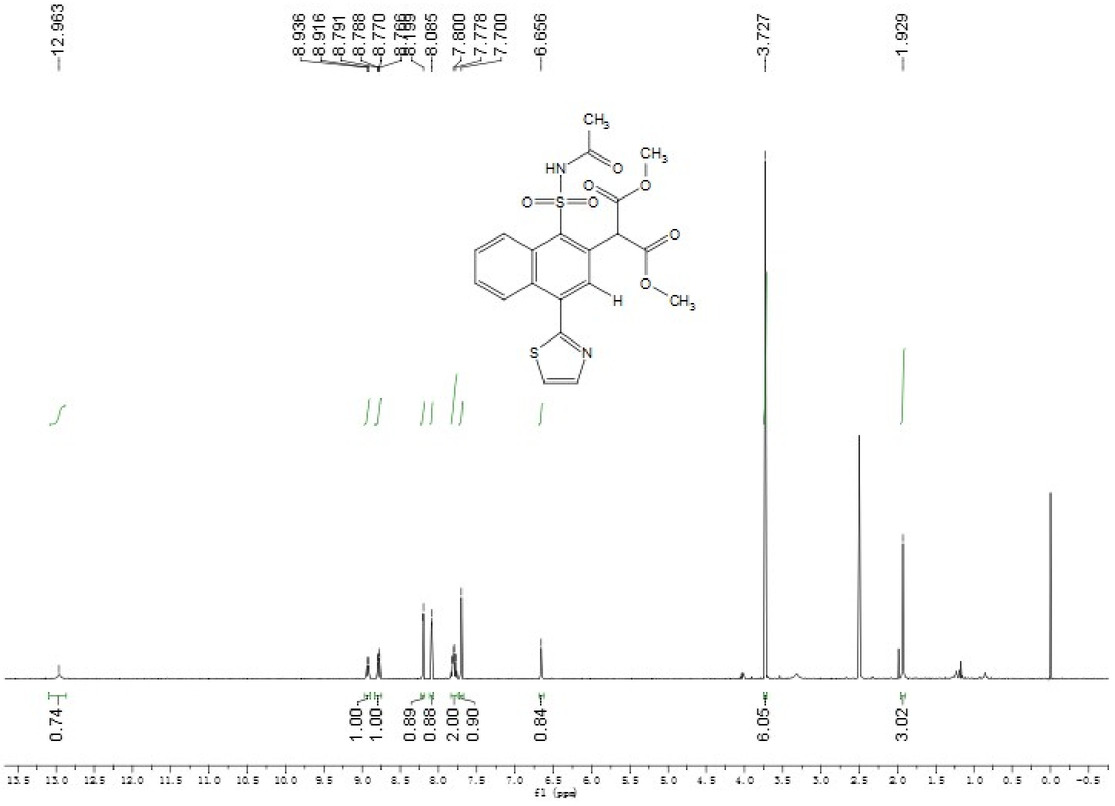

^{13}C NMR of compound **6**

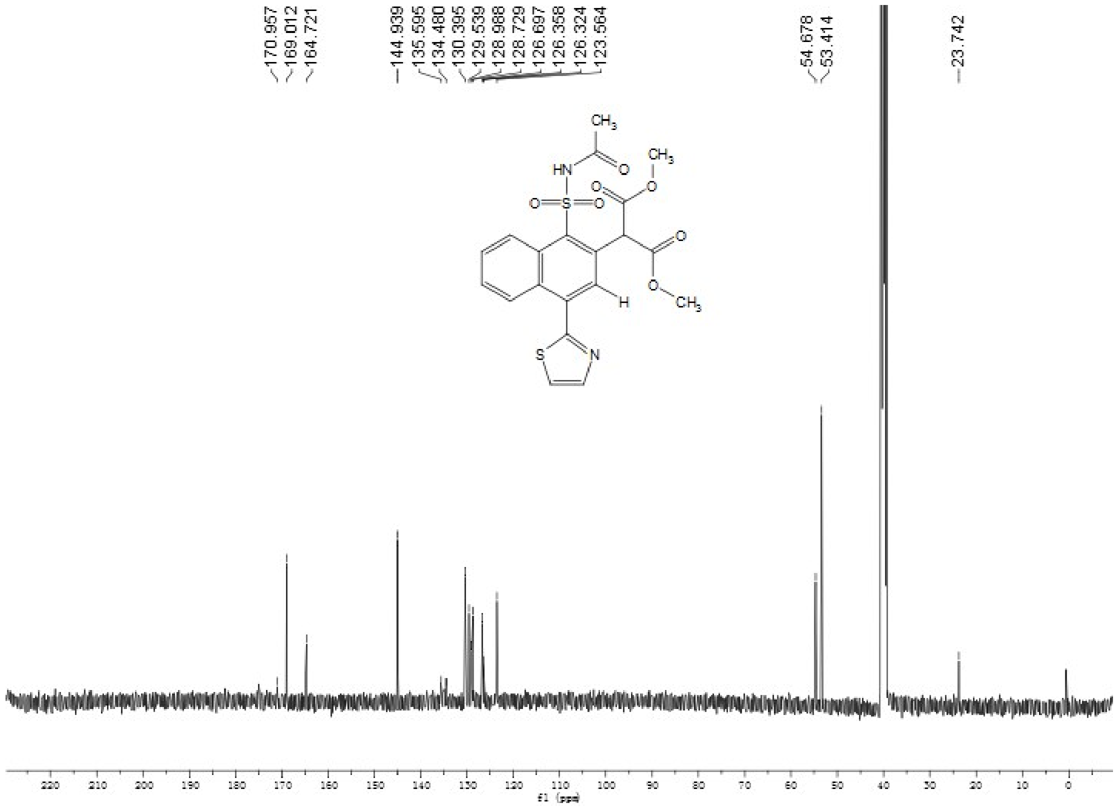

^{1}H NMR of compound **7**

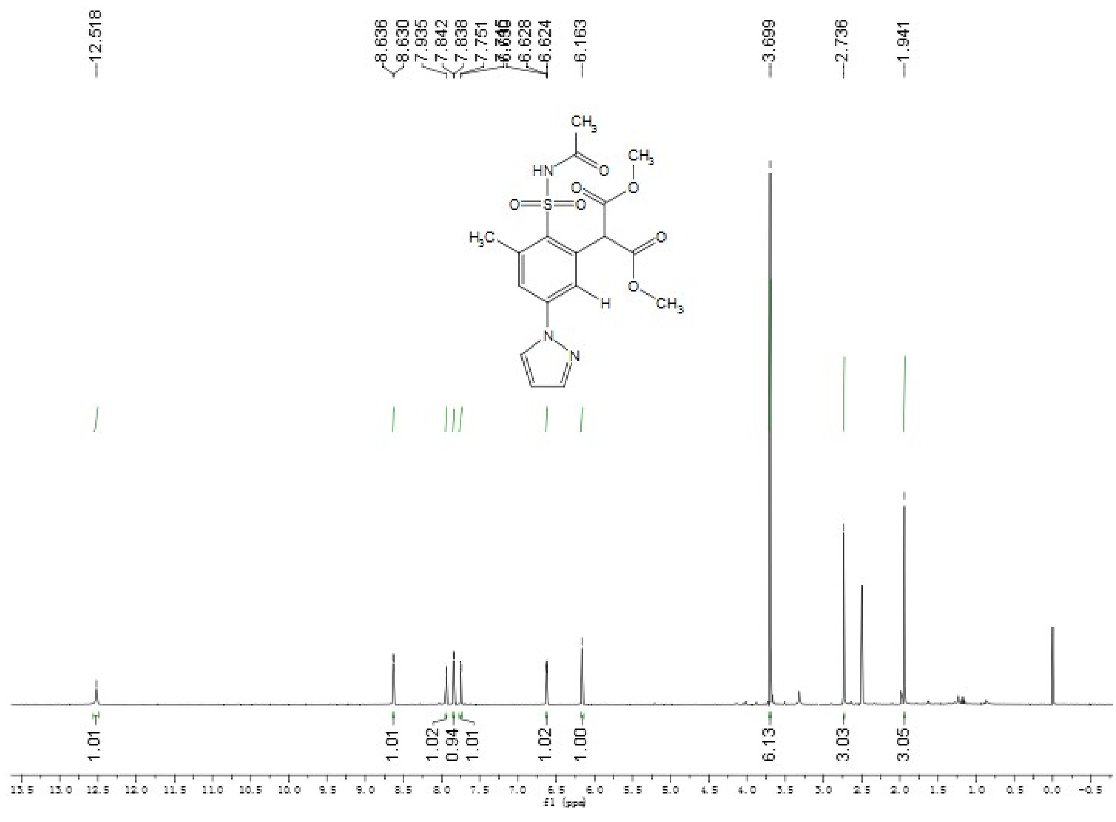

^{1}H NMR of compound **7**

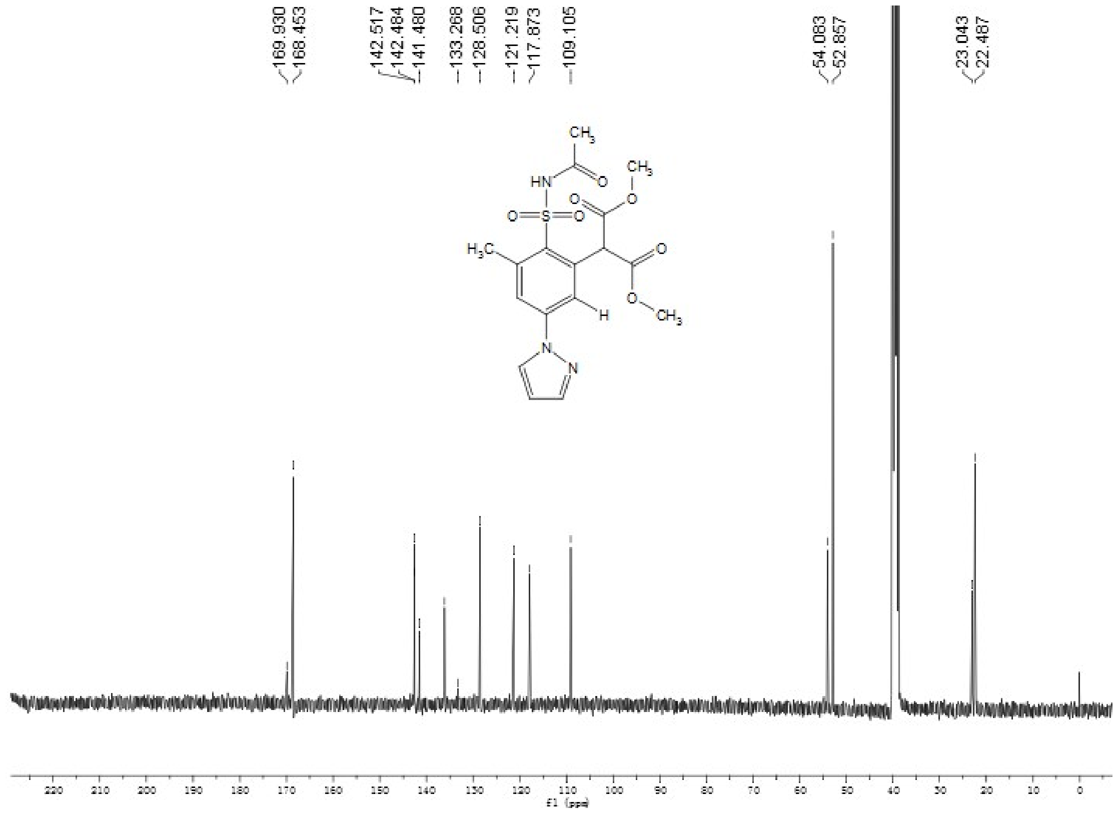

^{1}H NMR of compound **8**

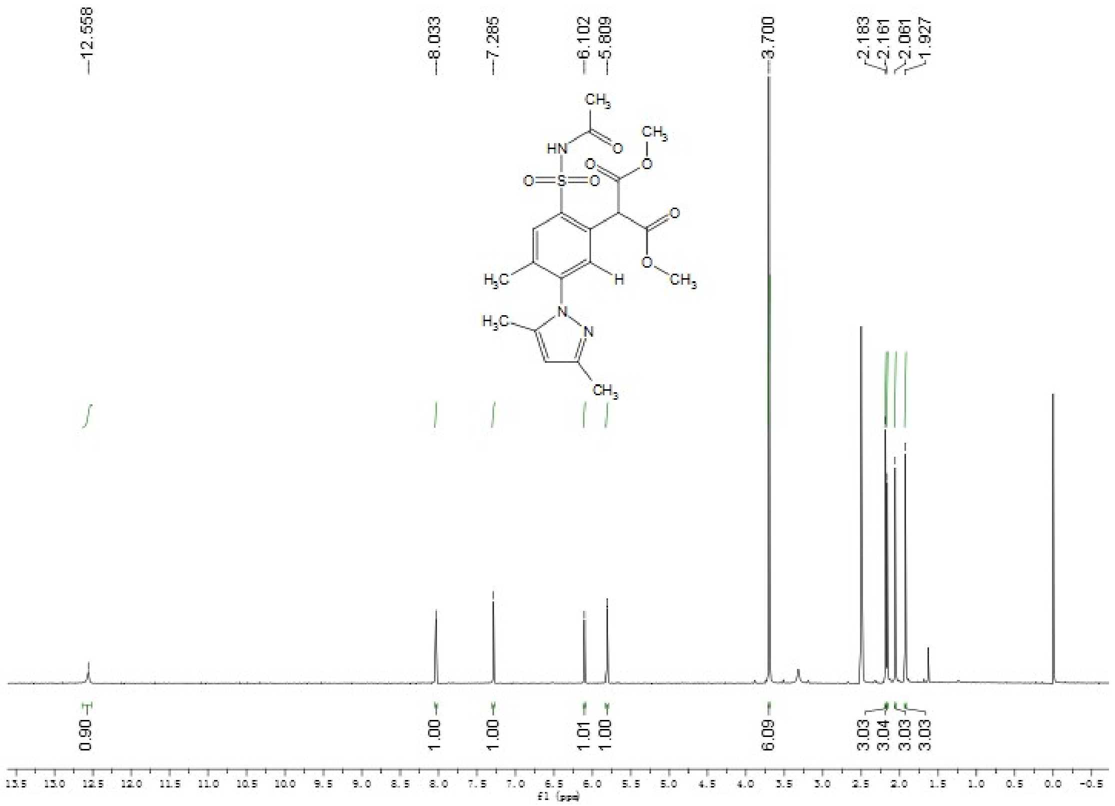

^{13}C NMR of compound **8**

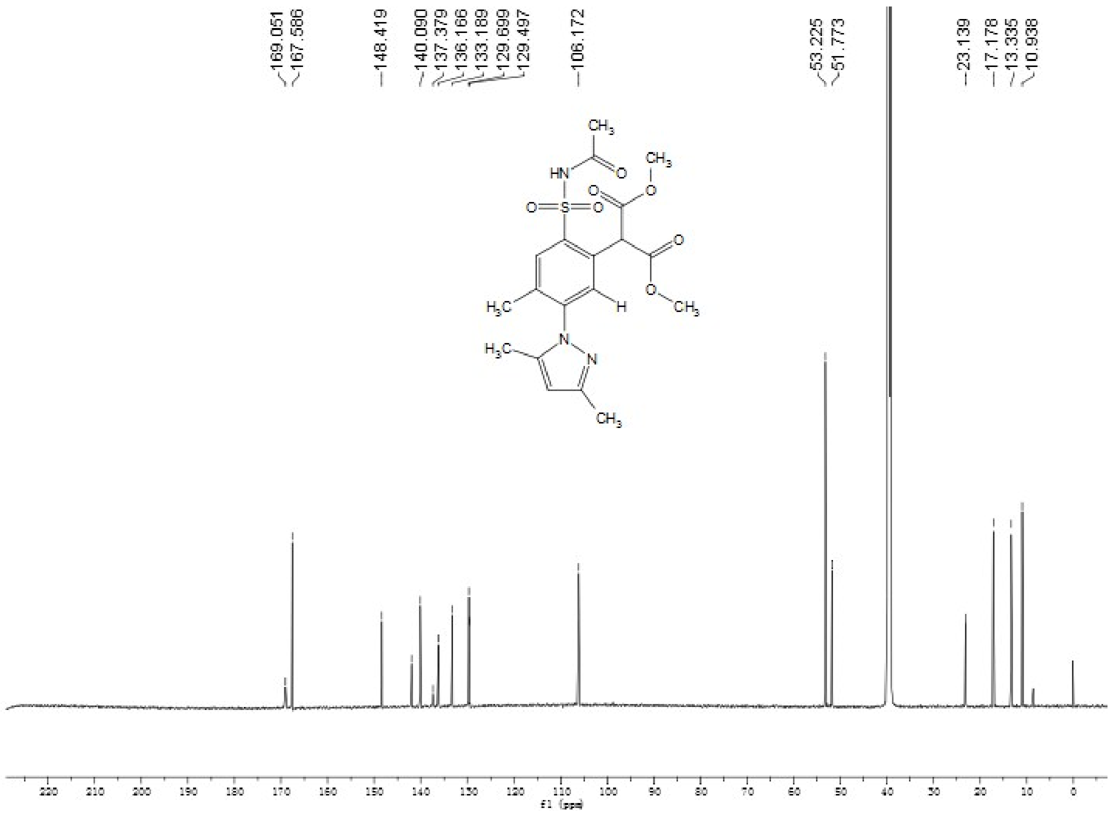

^{1}H NMR of compound **9**

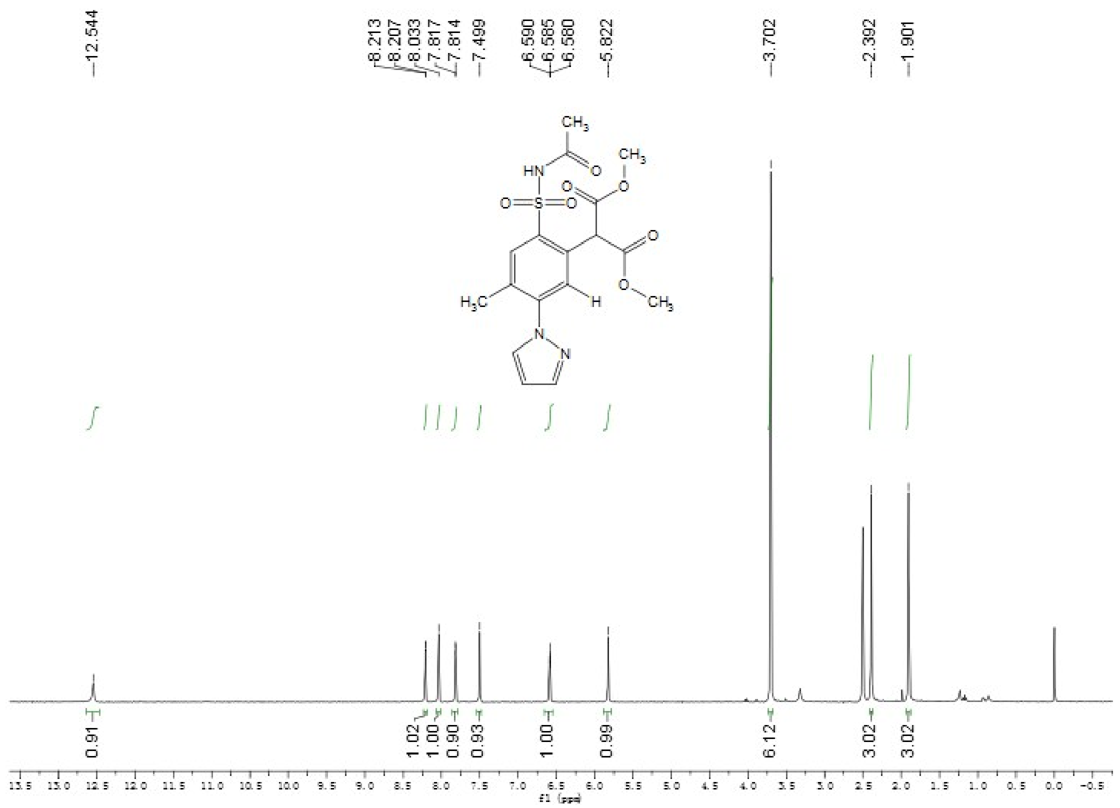

^{13}C NMR of compound **9**

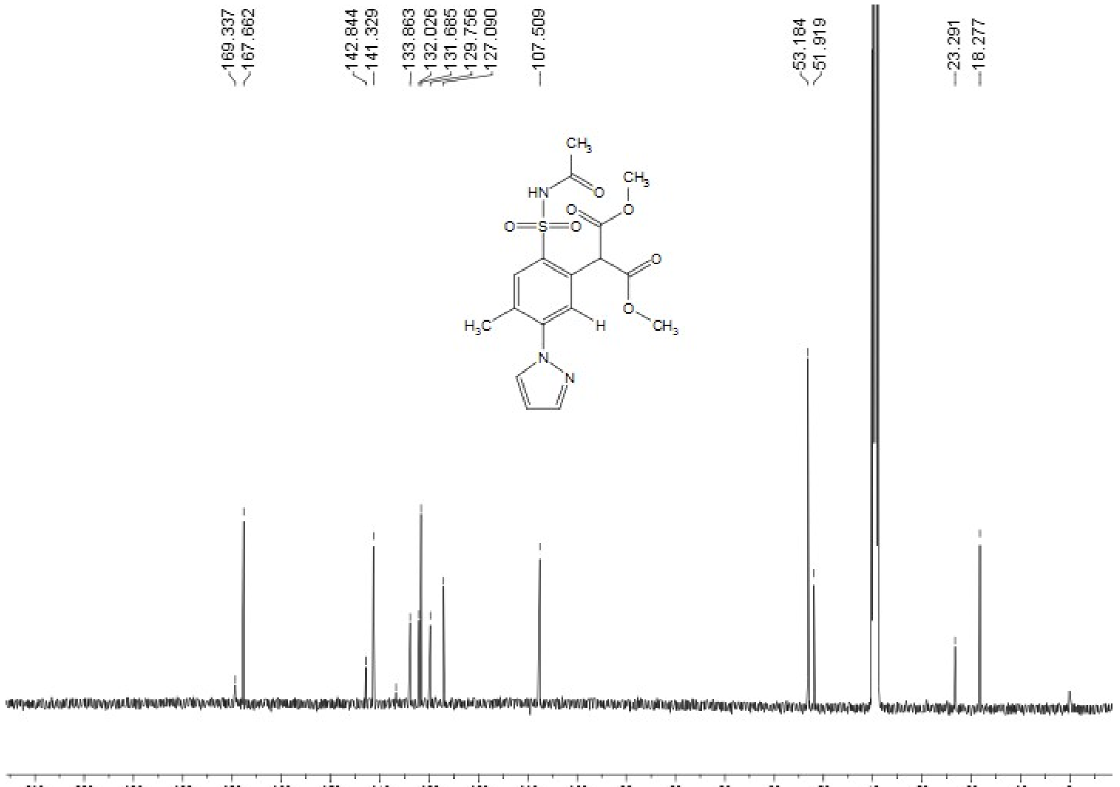

^{1}H NMR of compound **10**

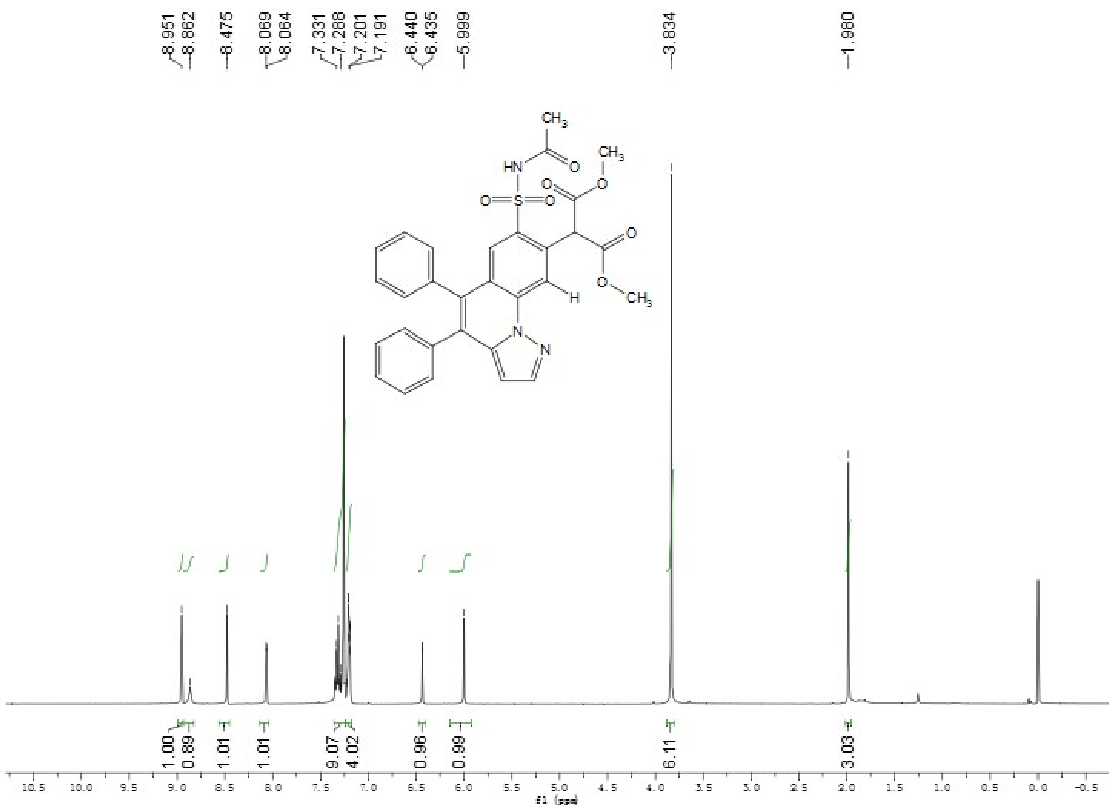

^{13}C NMR of compound **10**

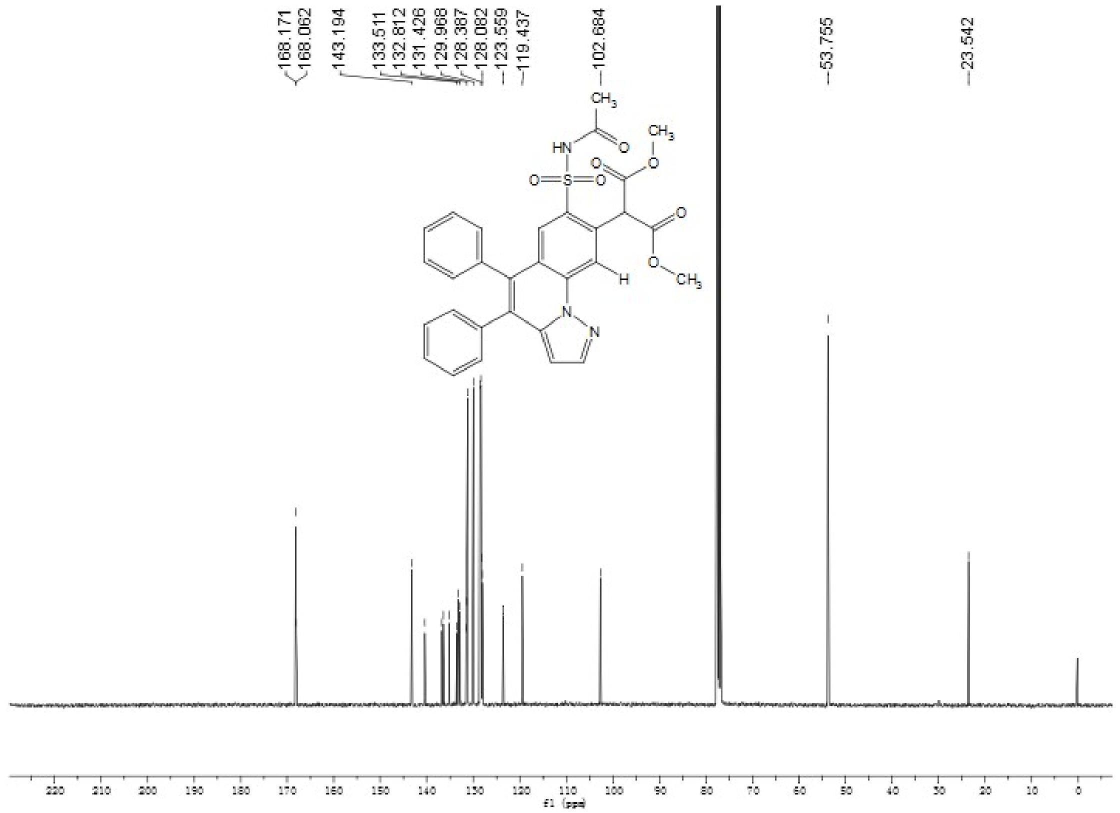

^{1}H NMR of compound **11**

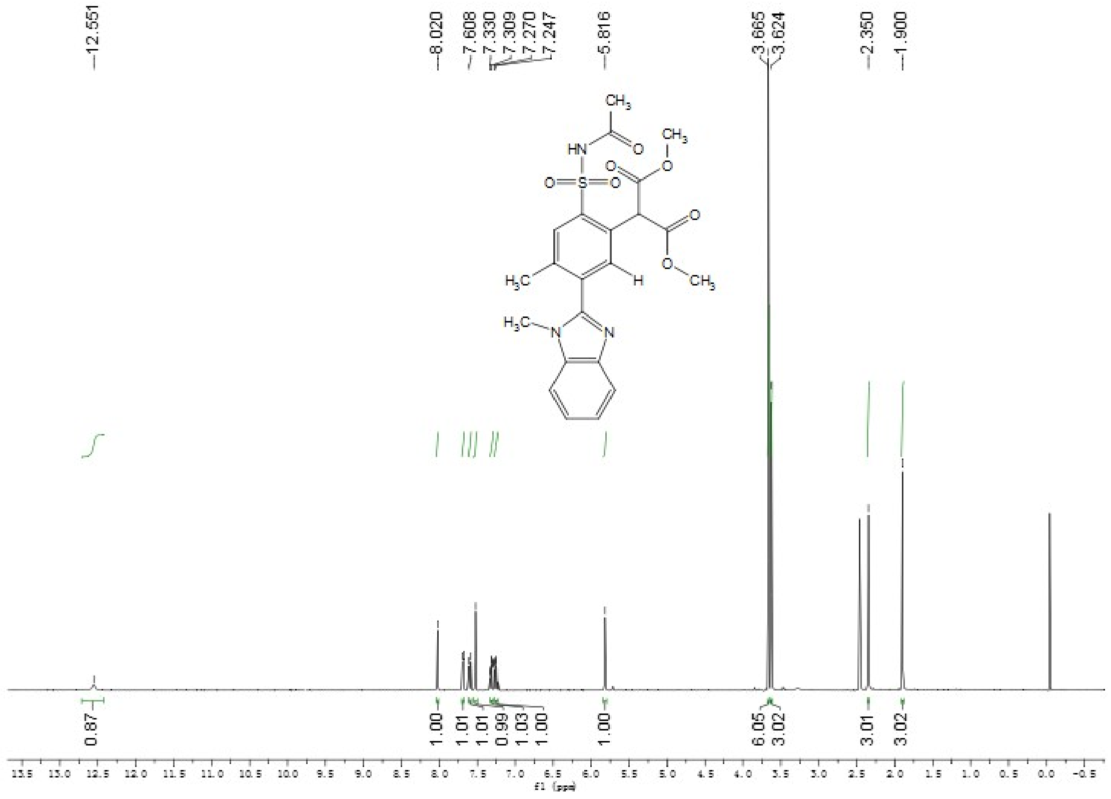

^{13}C NMR of compound **11**

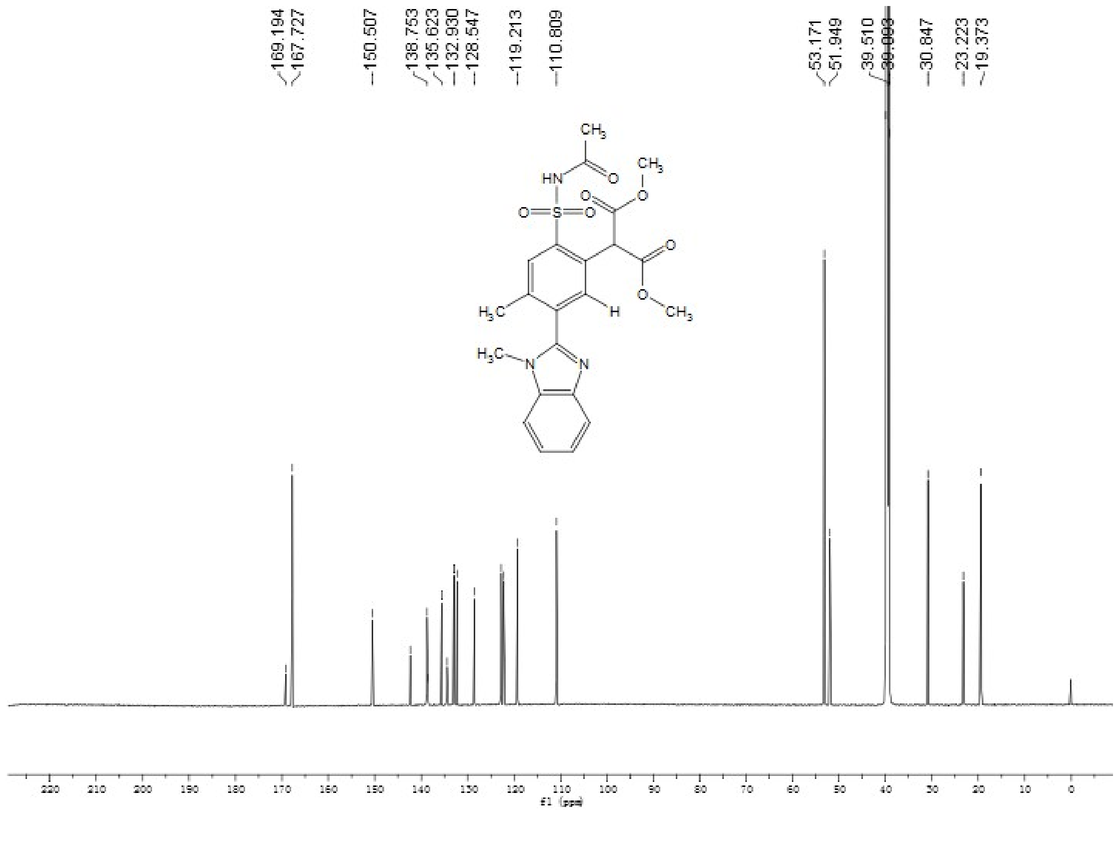

^{1}H NMR of compound **12**

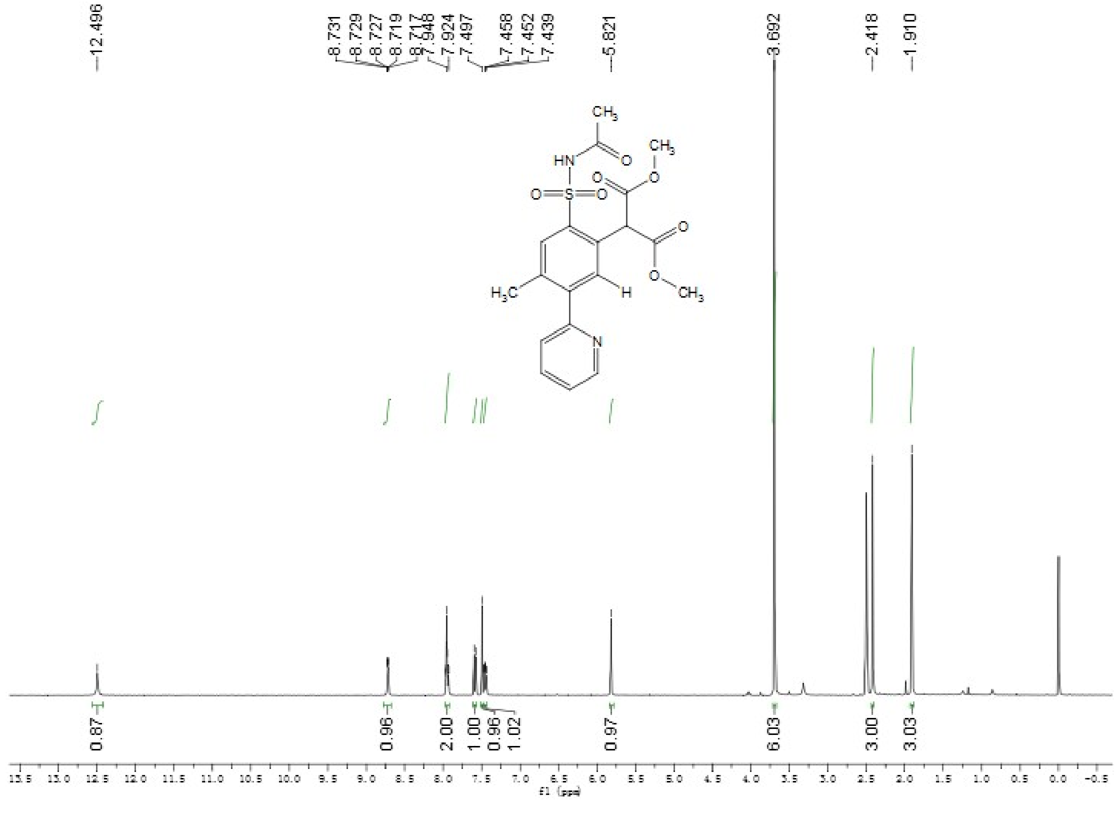

^{13}C NMR of compound **12**

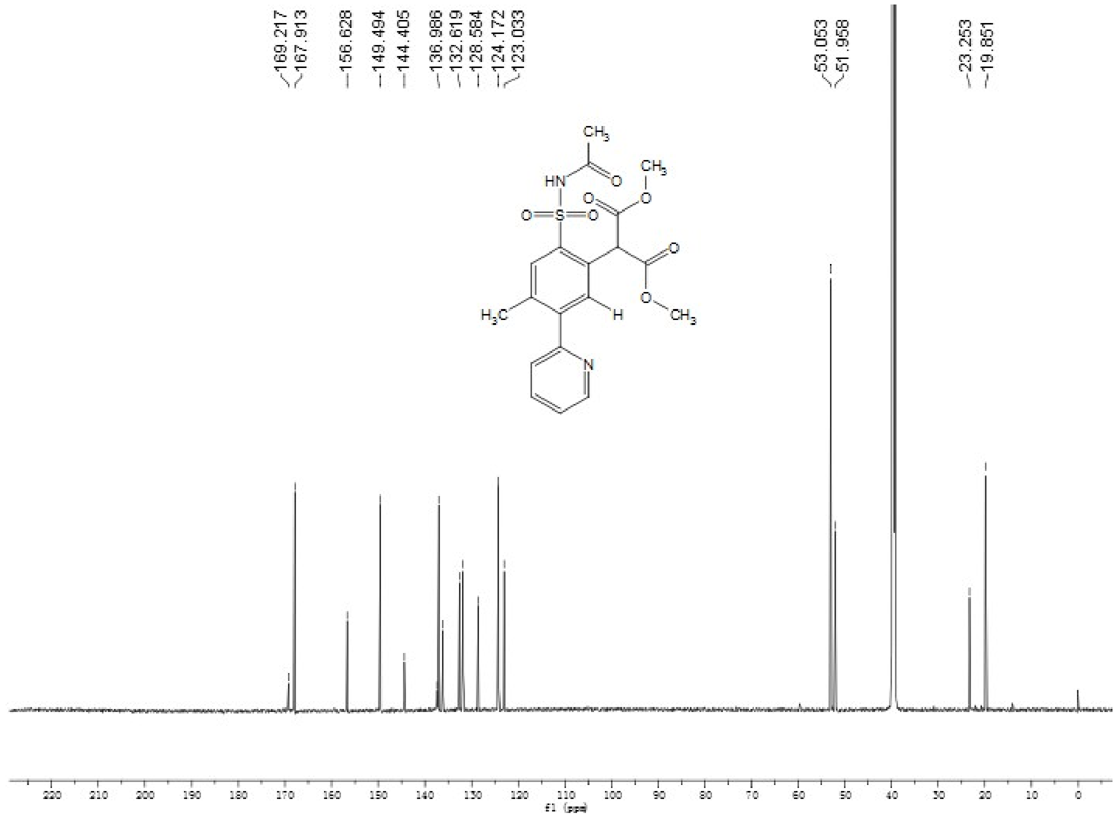

^{1}H NMR of compound **13**

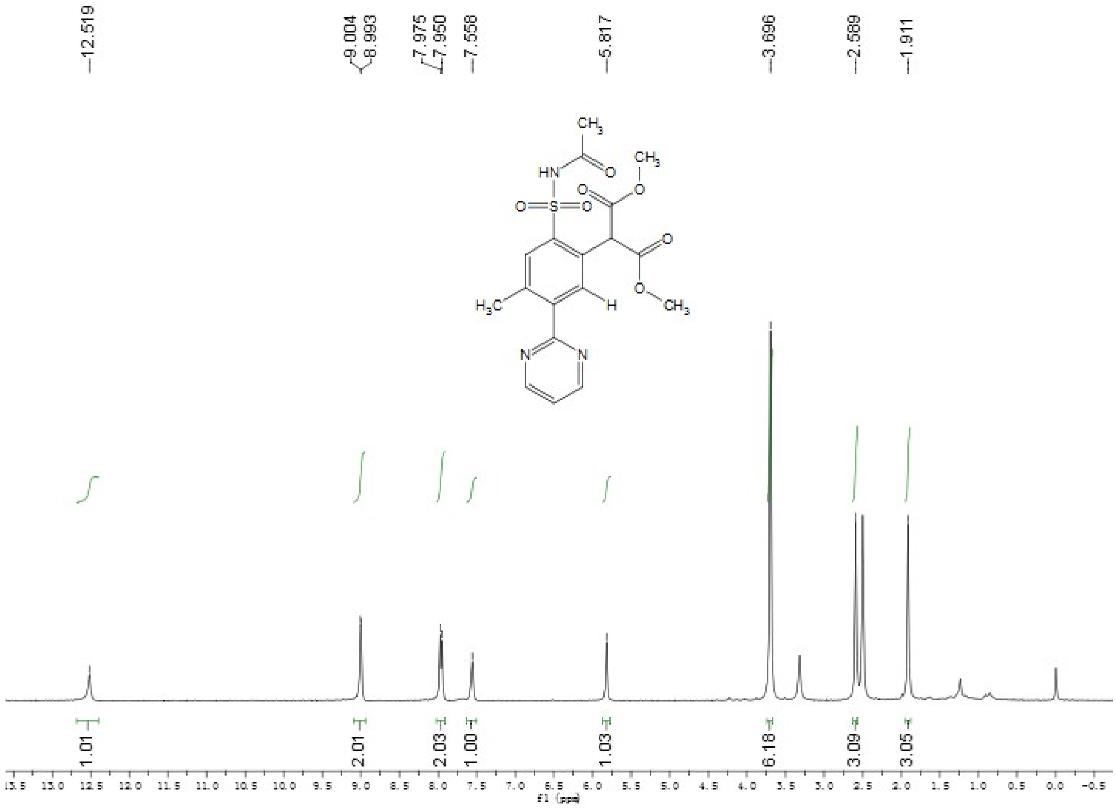

^{13}C NMR of compound **13**

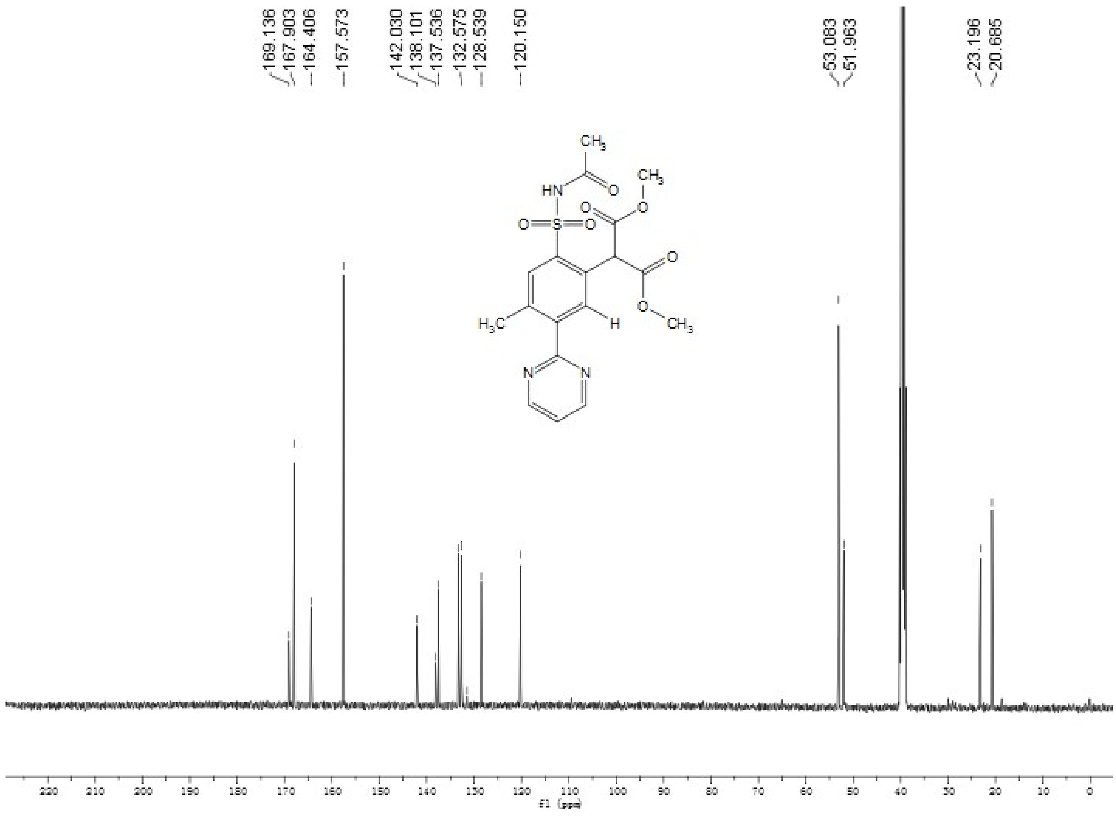

^{1}H NMR of compound **14**

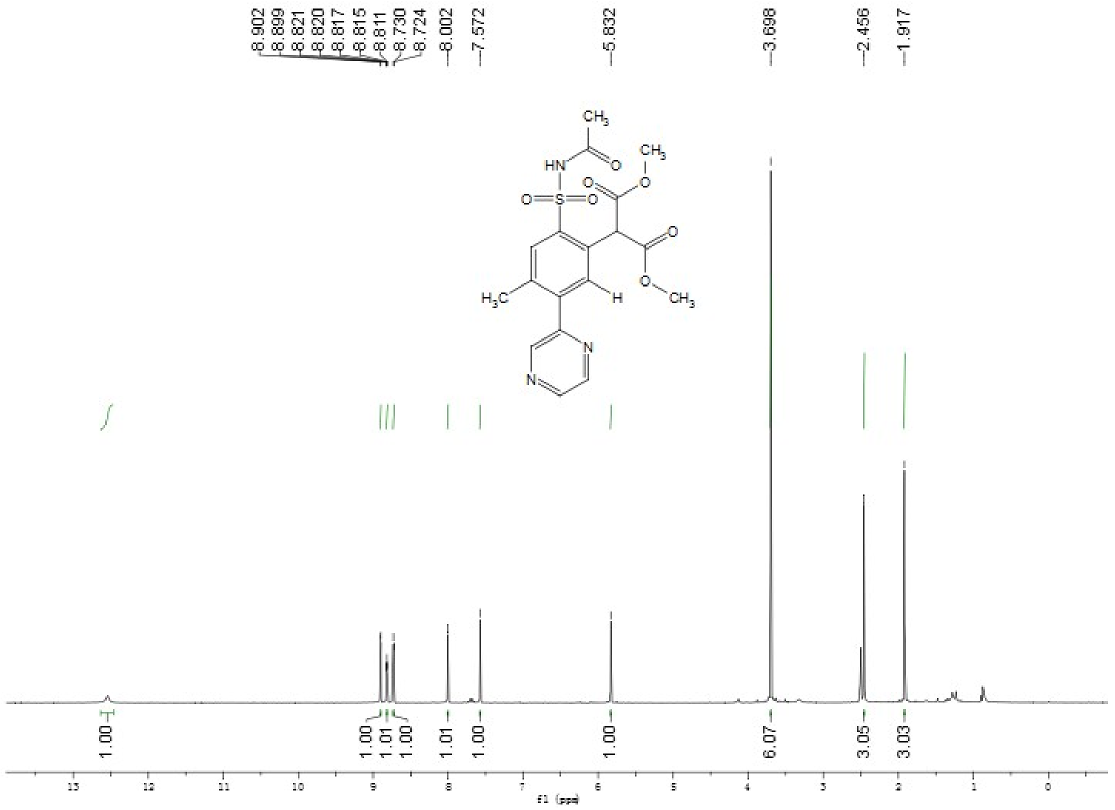

^{13}C NMR of compound **14**

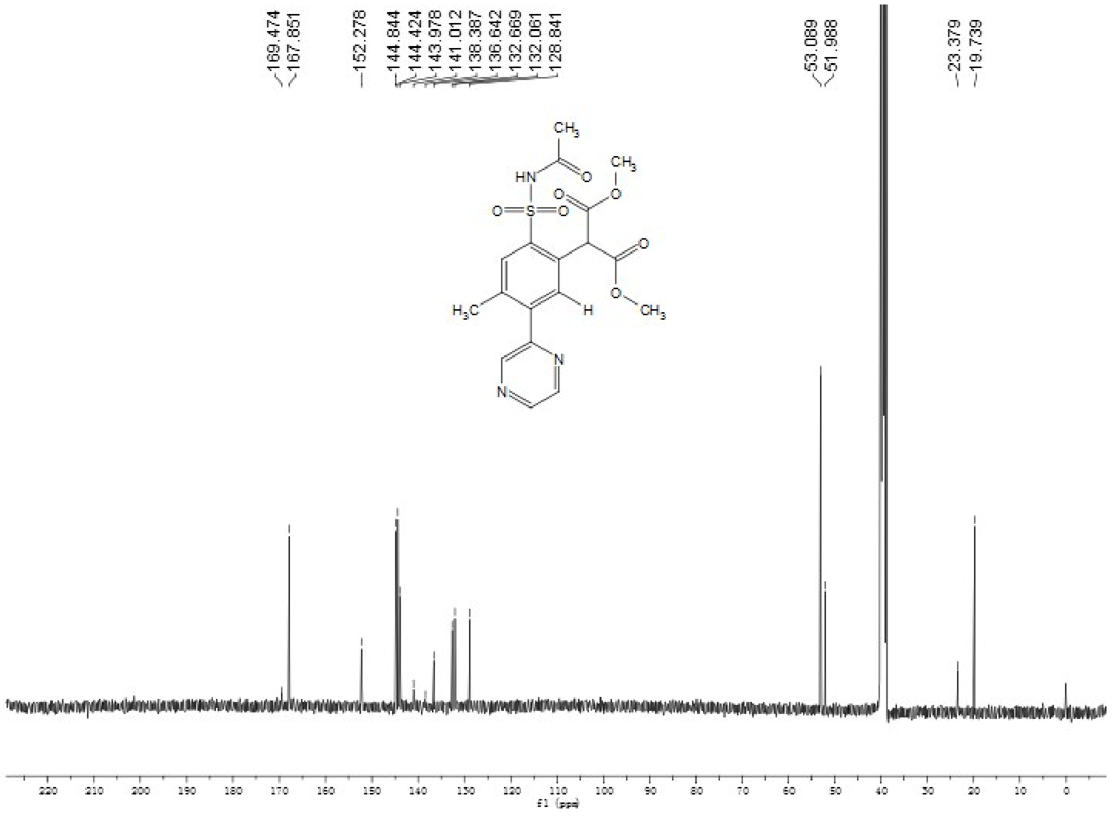

^{1}H NMR of compound **15**

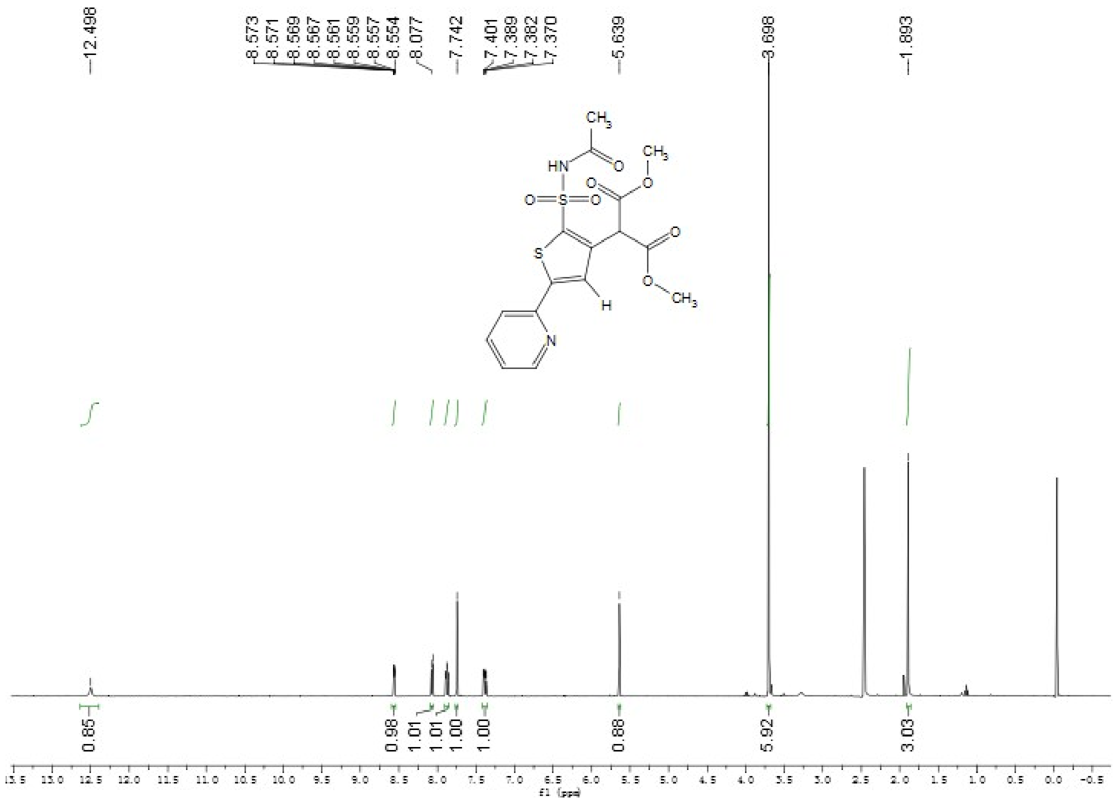

^{13}C NMR of compound **15**

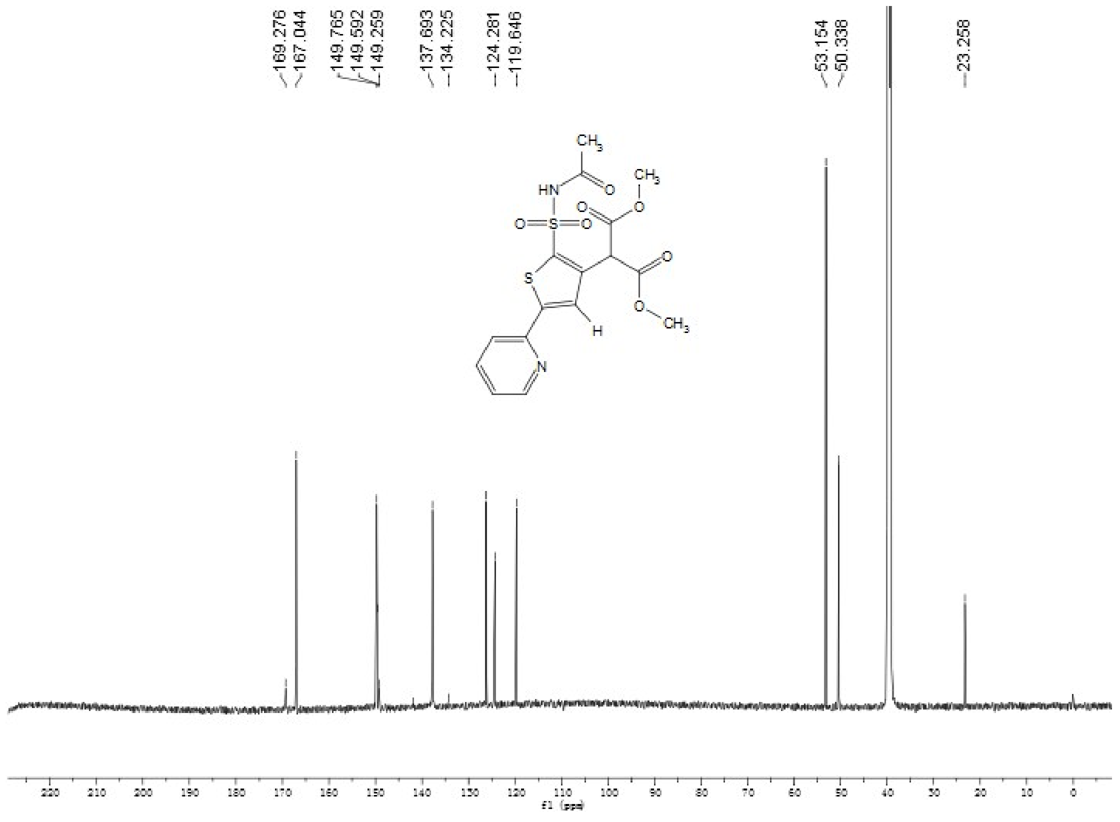

^{1}H NMR of compound **16**

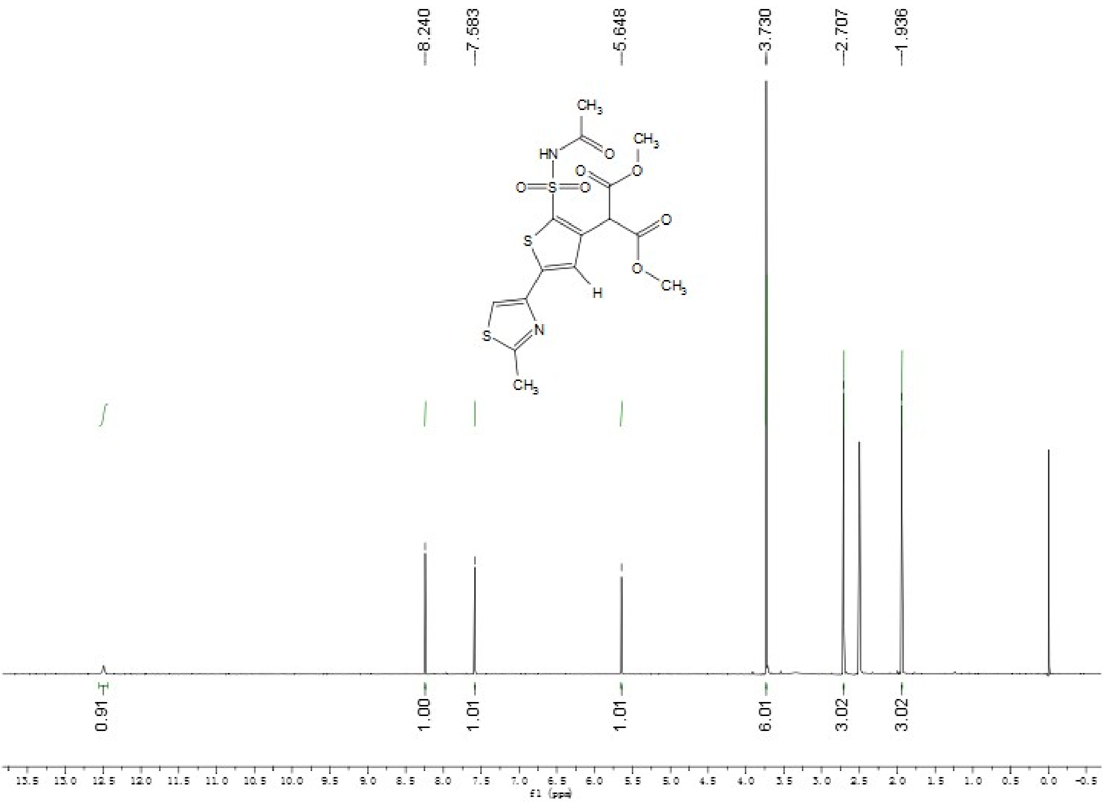

^{13}C NMR of compound **16**

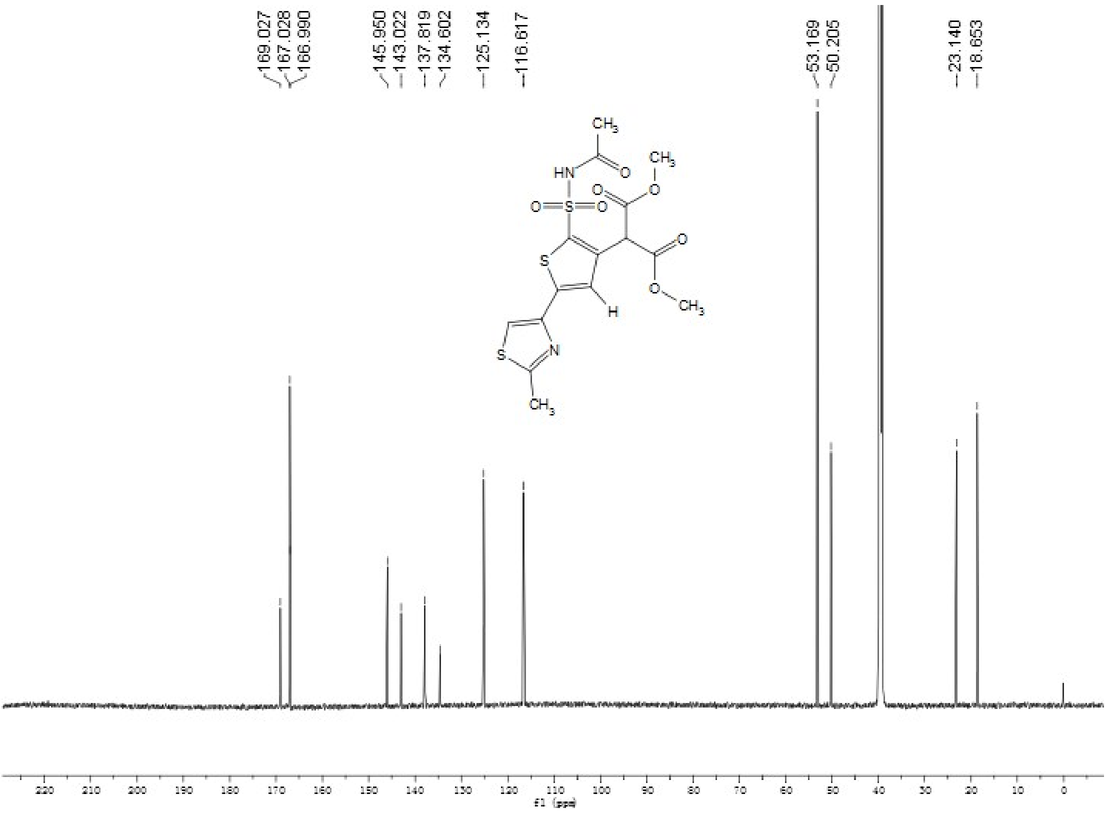

^{1}H NMR of compound **17**

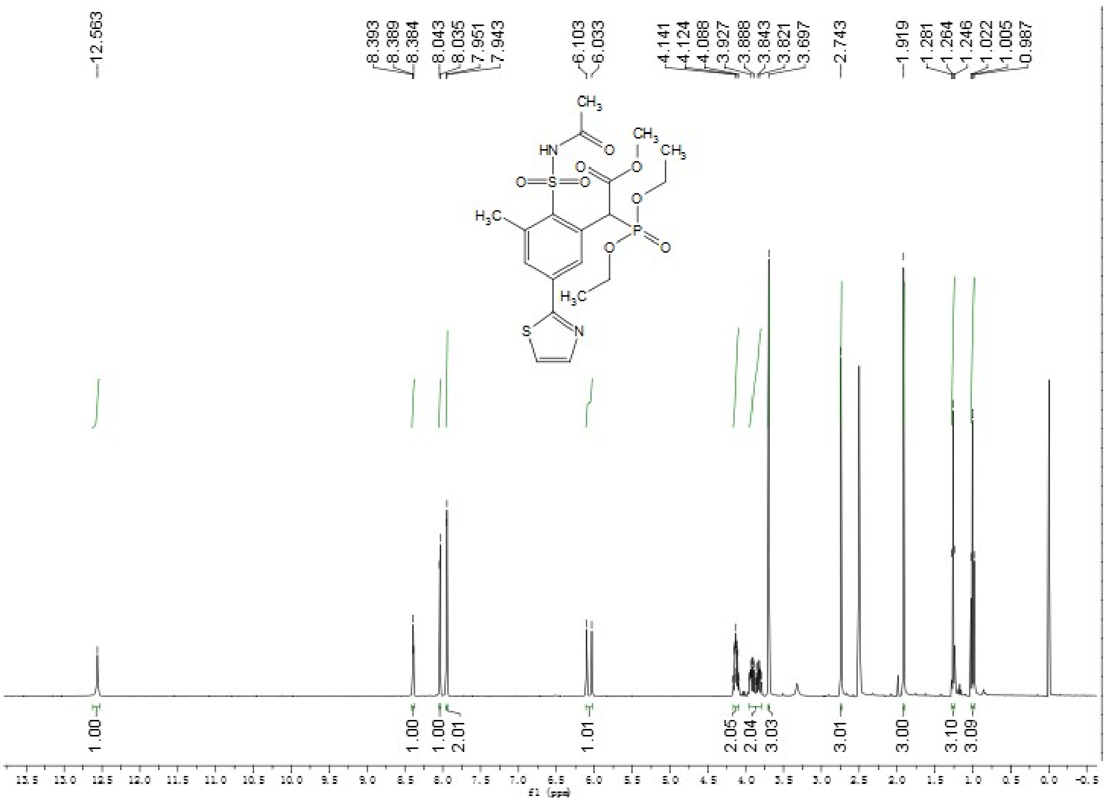

^{13}C NMR of compound **17**

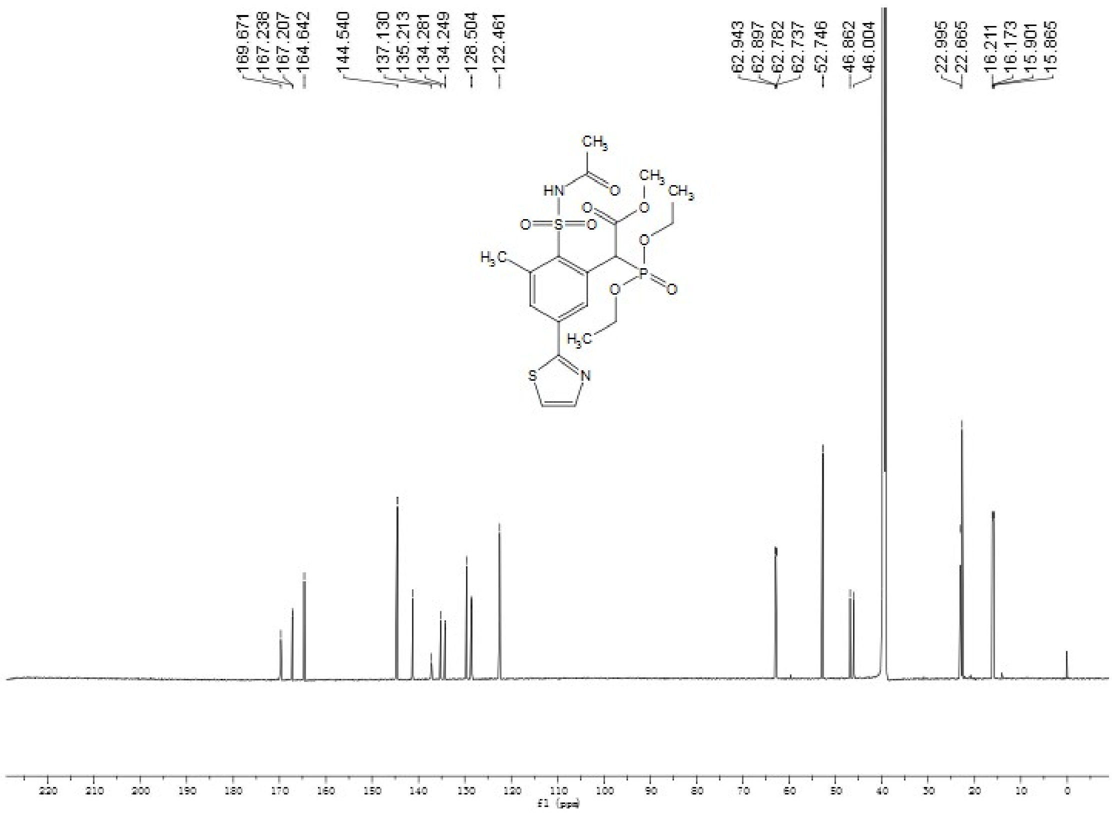

^{1}H NMR of compound **18**

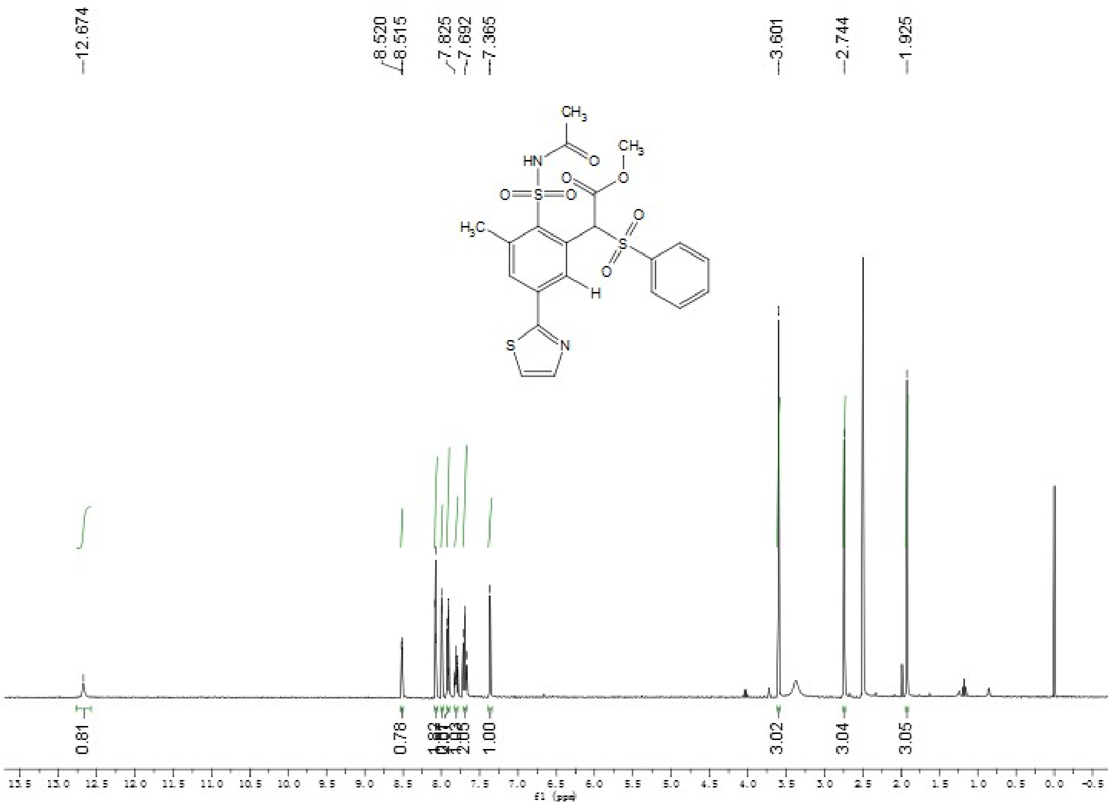

^{13}C NMR of compound **18**

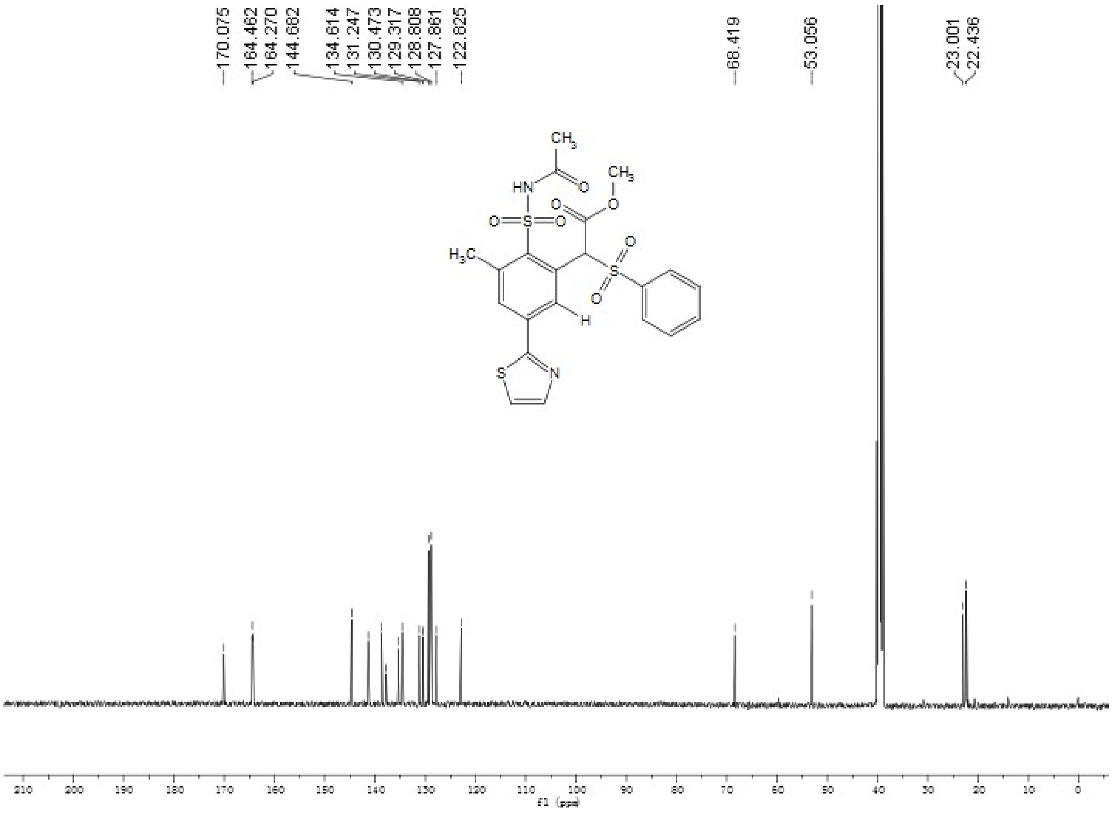

^{1}H NMR of compound **19**

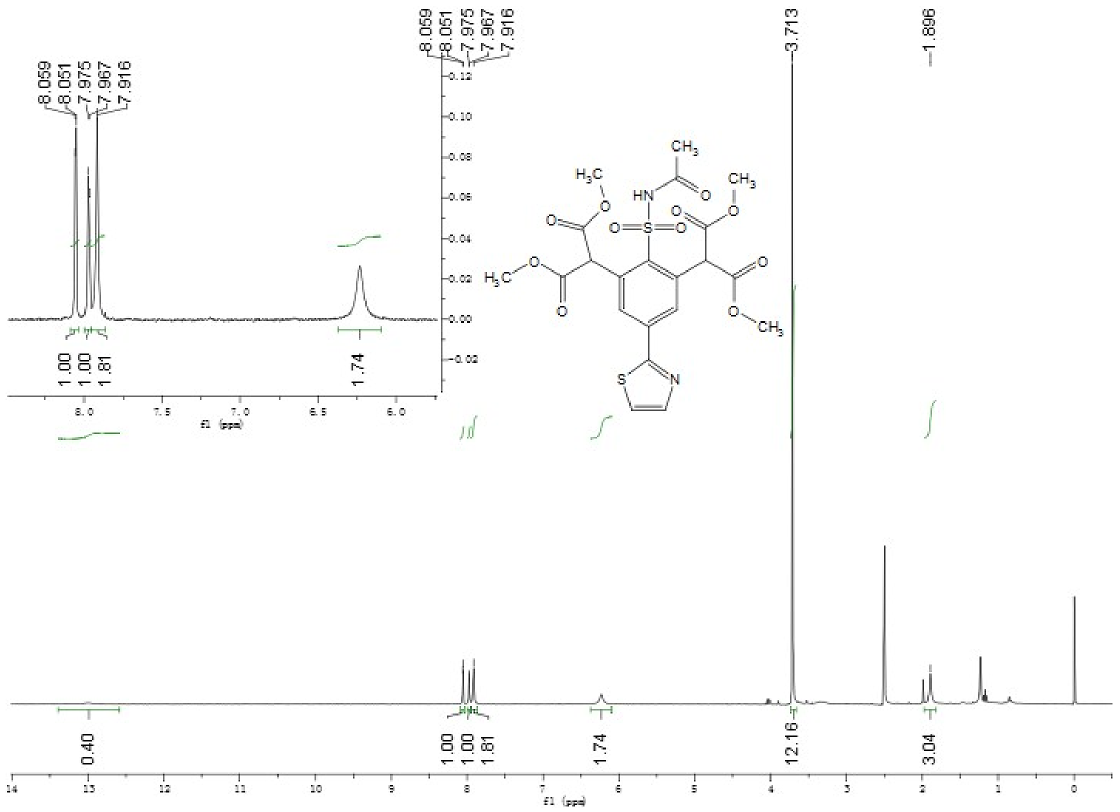

^{13}C NMR of compound **19**

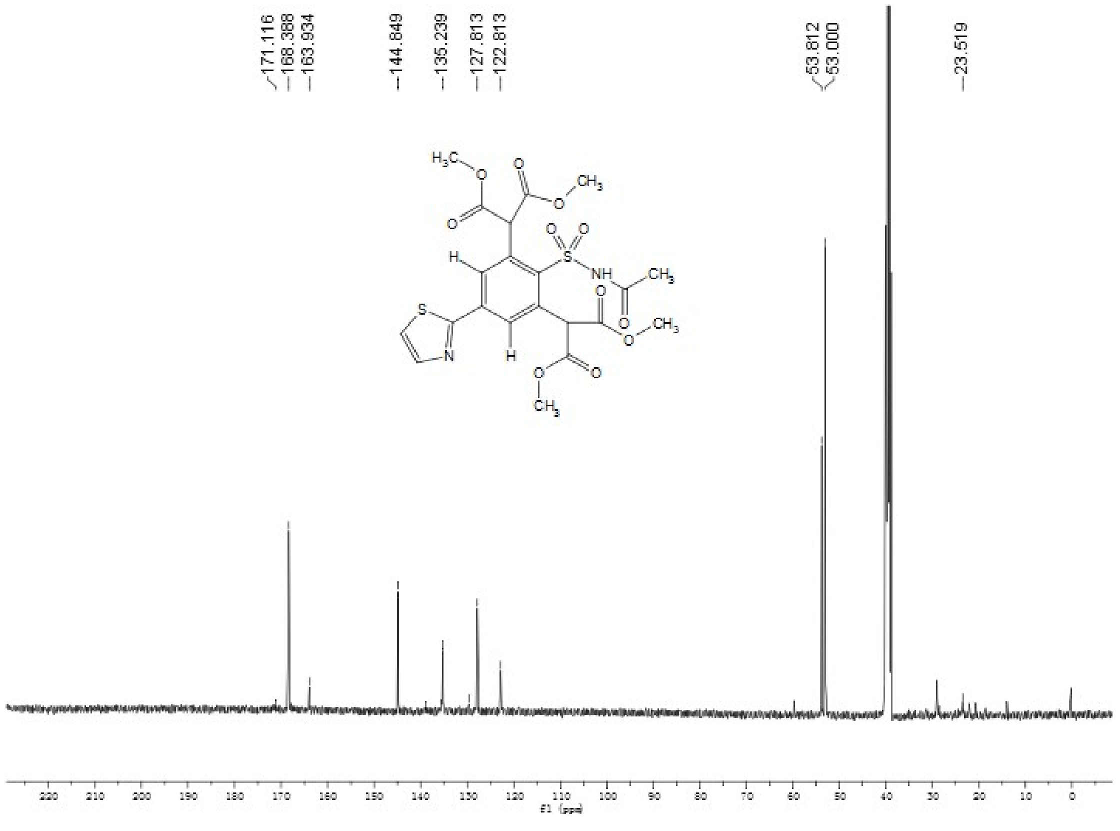

^{1}H NMR of compound **20**

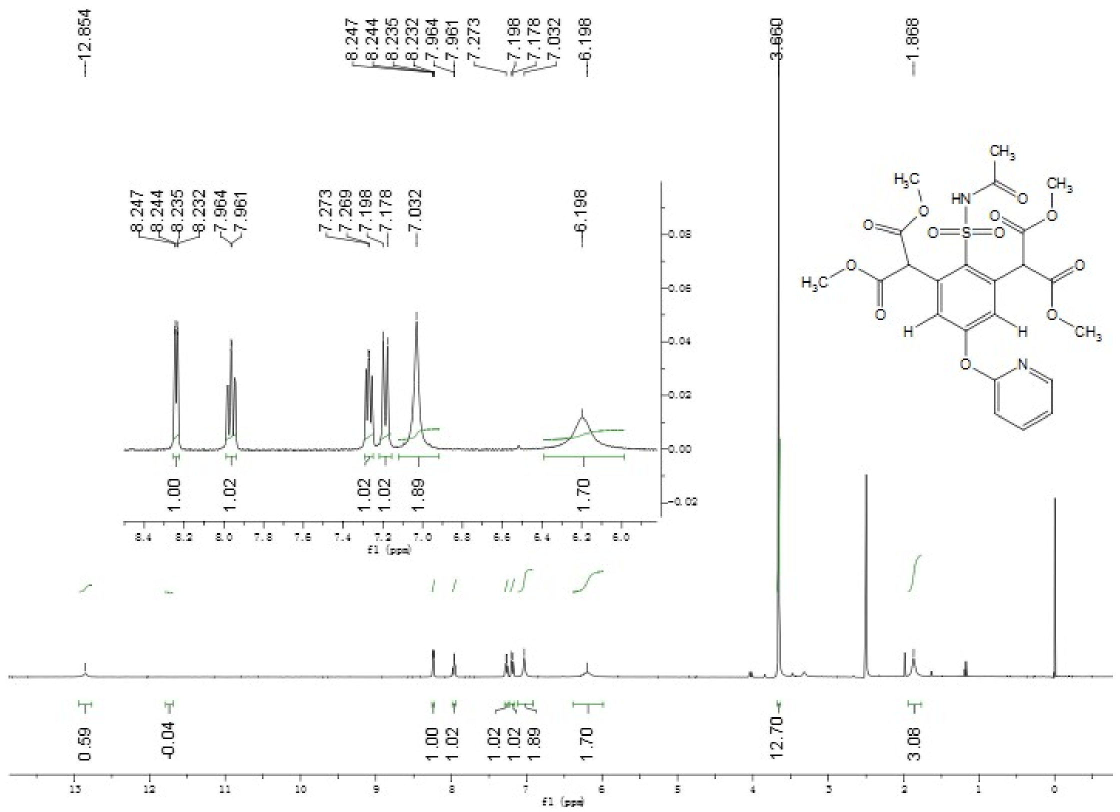

^{13}C NMR of compound **20**

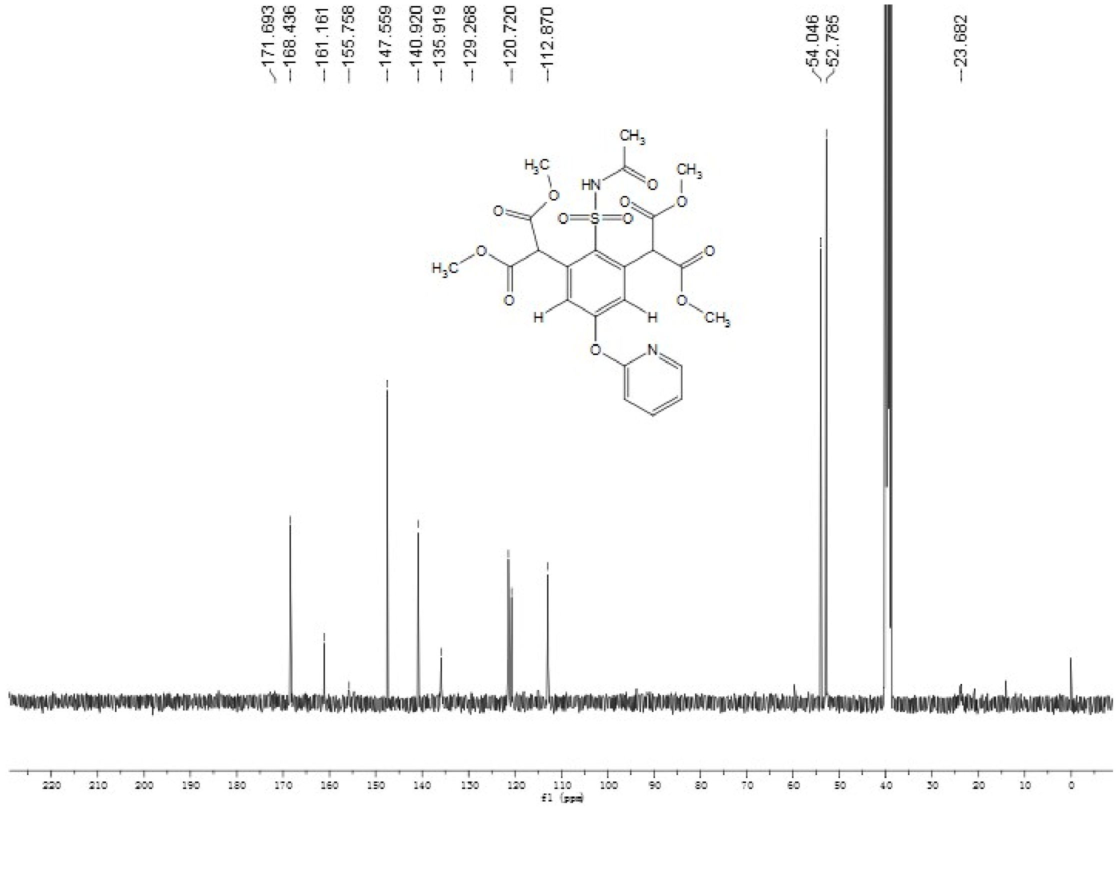

^1H NMR of compound **21**

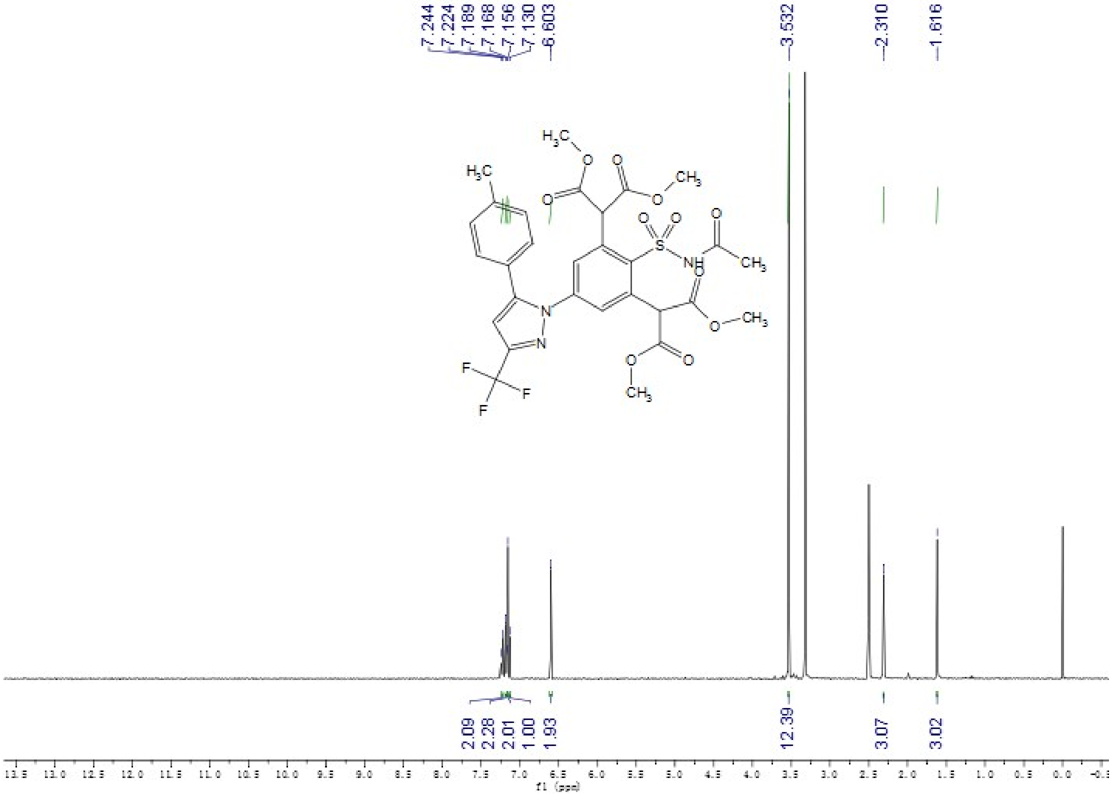

^{13}C NMR of compound **21**

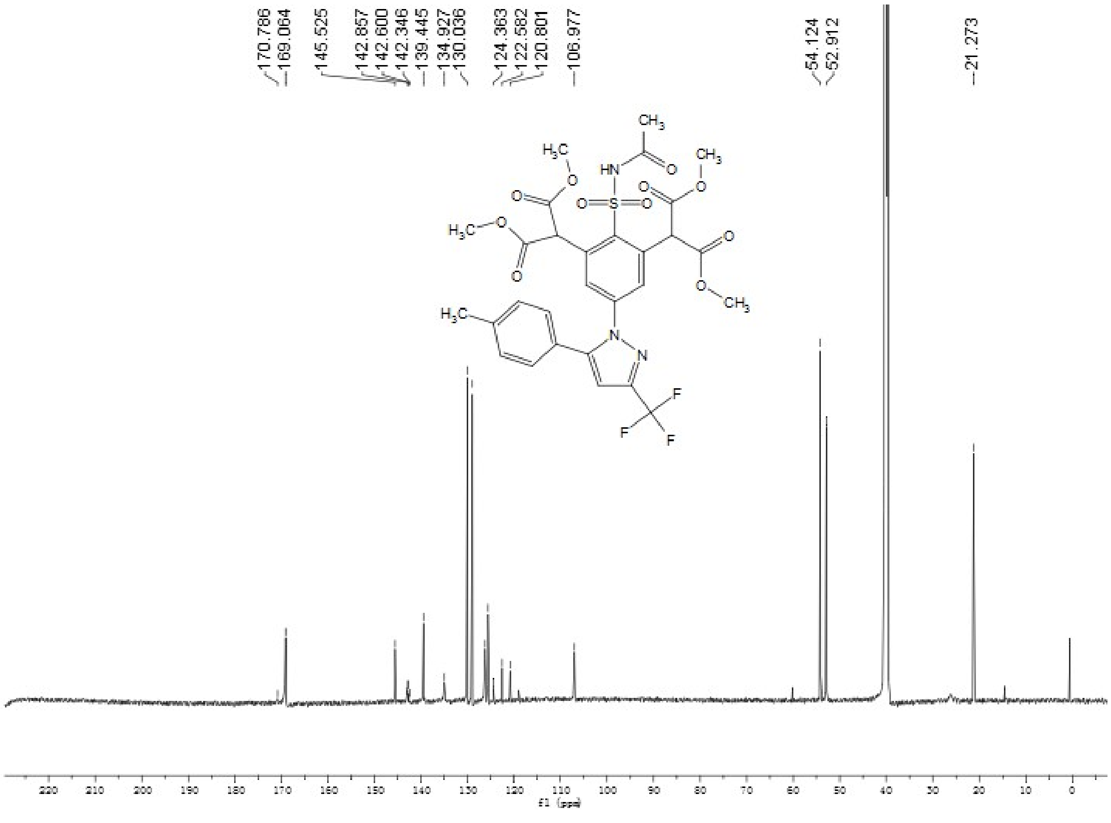

^{1}H NMR of compound **22**

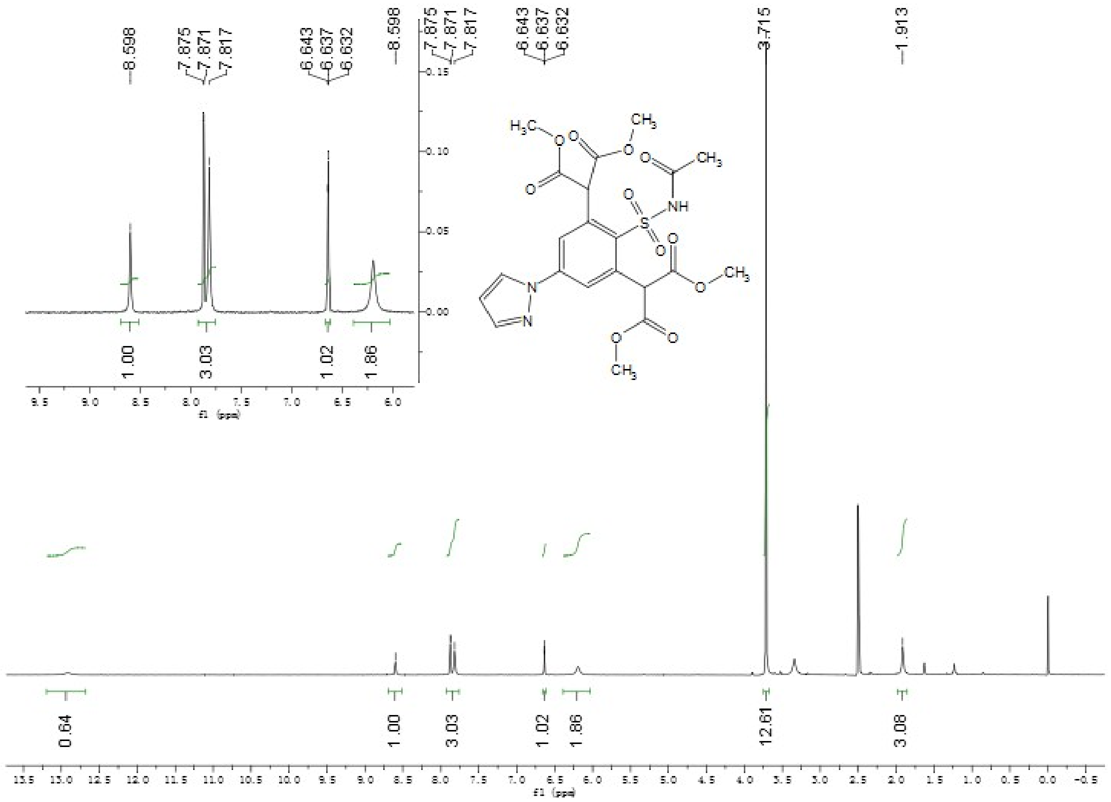

^{13}C NMR of compound **22**

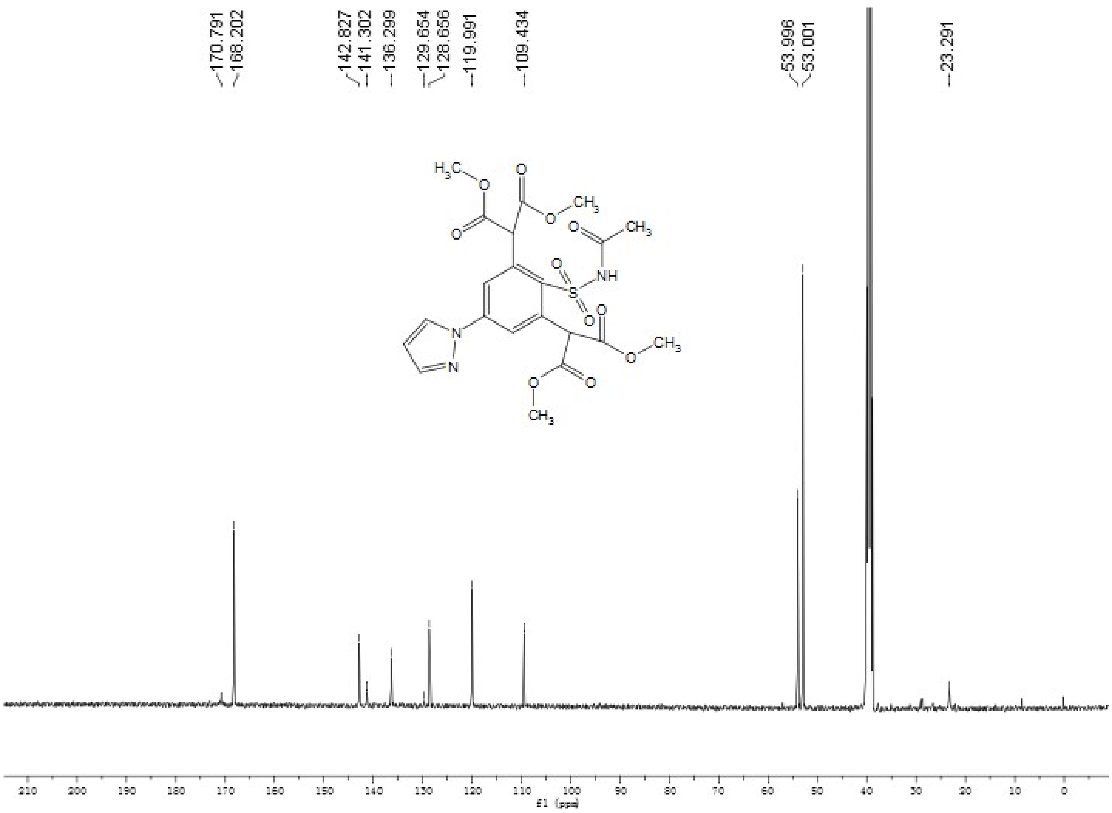

^{1}H NMR of compound **23**

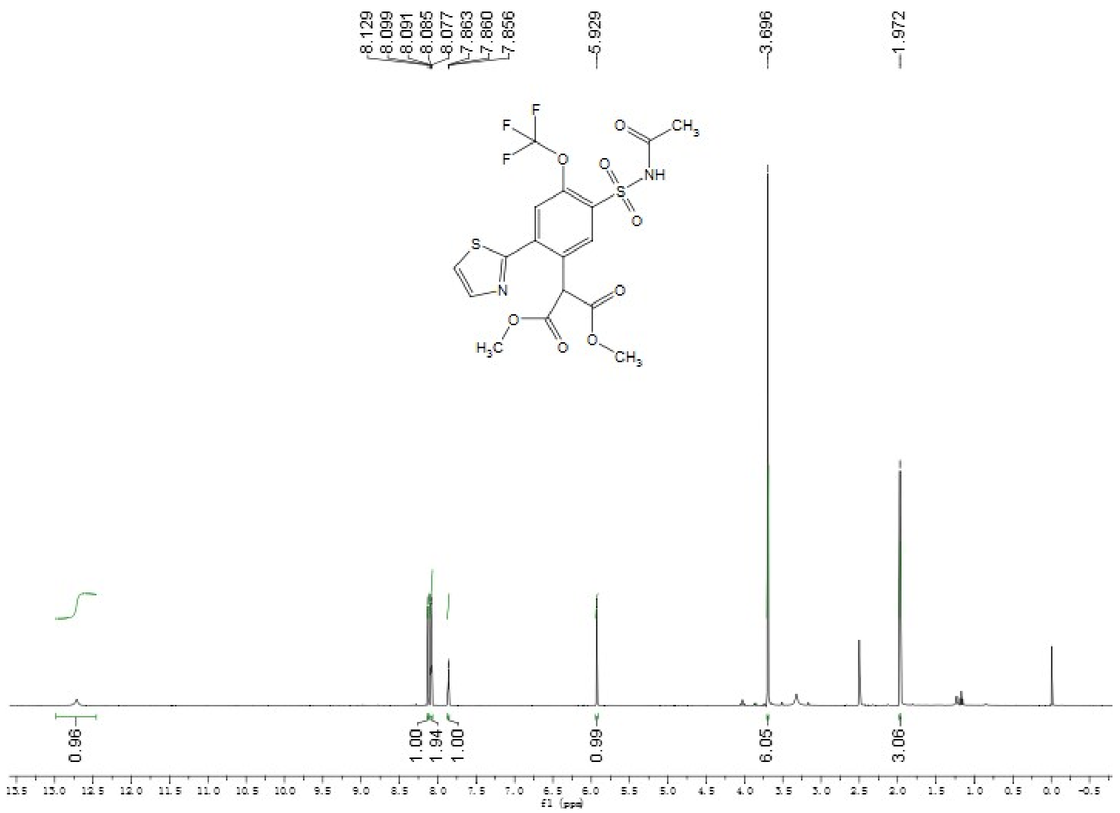

^{13}C NMR of compound **23**

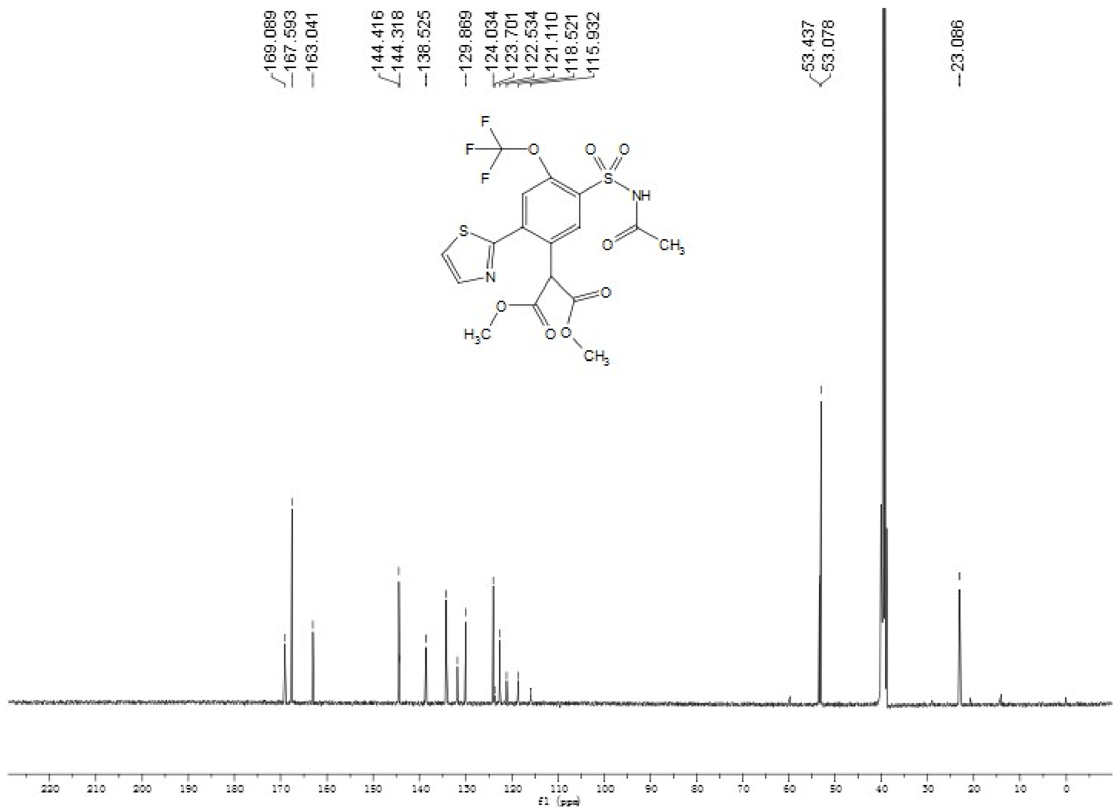

^{1}H NMR of compound **24**

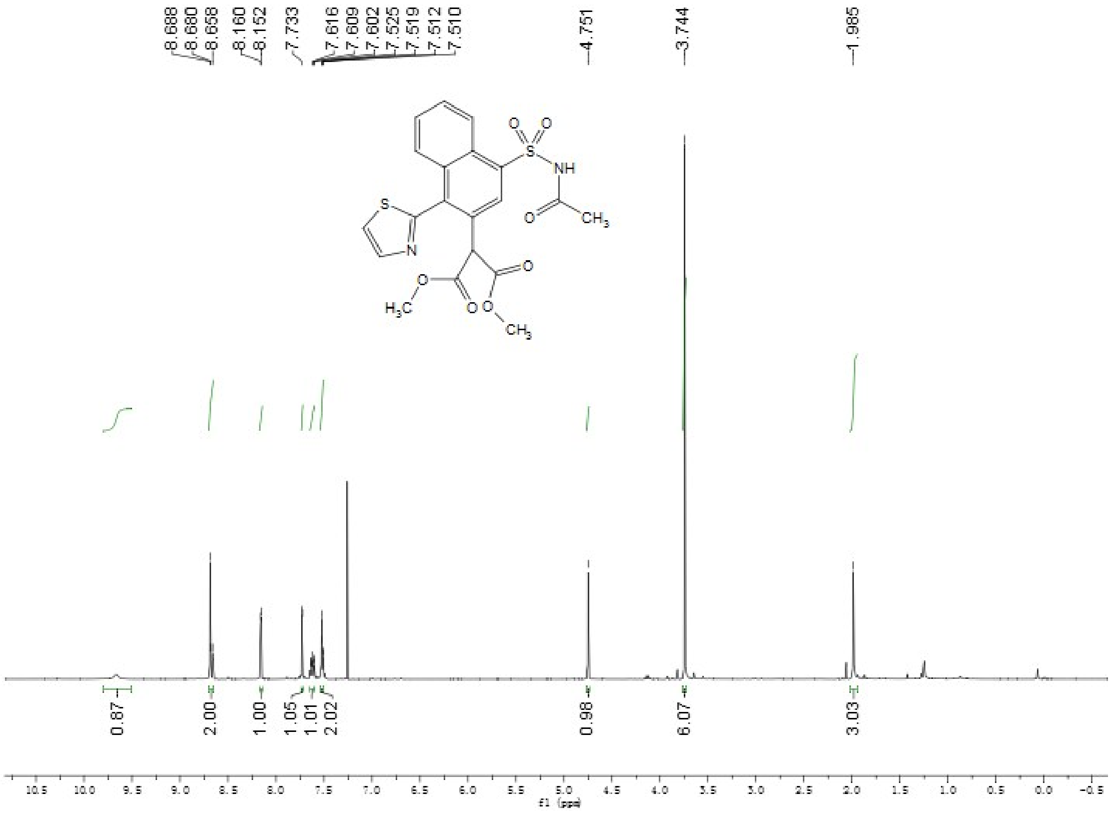

^{13}C NMR of compound **24**

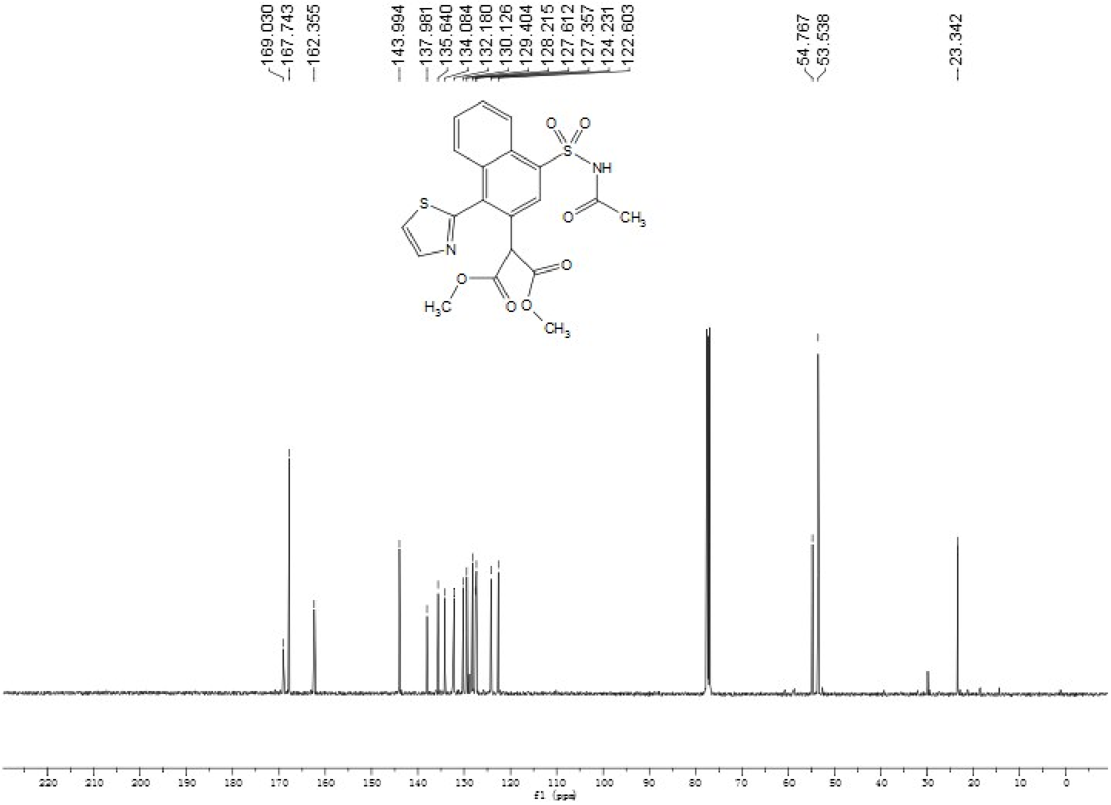

^{1}H NMR of compound **25**

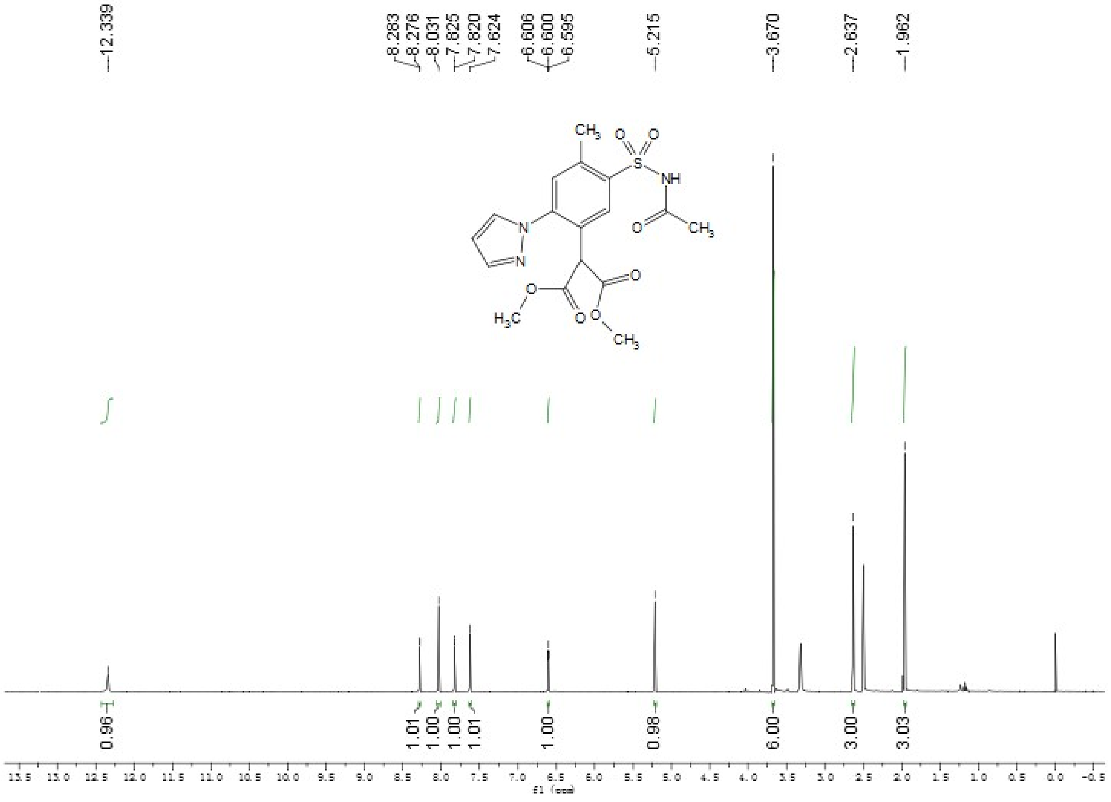

^{13}C NMR of compound **25**

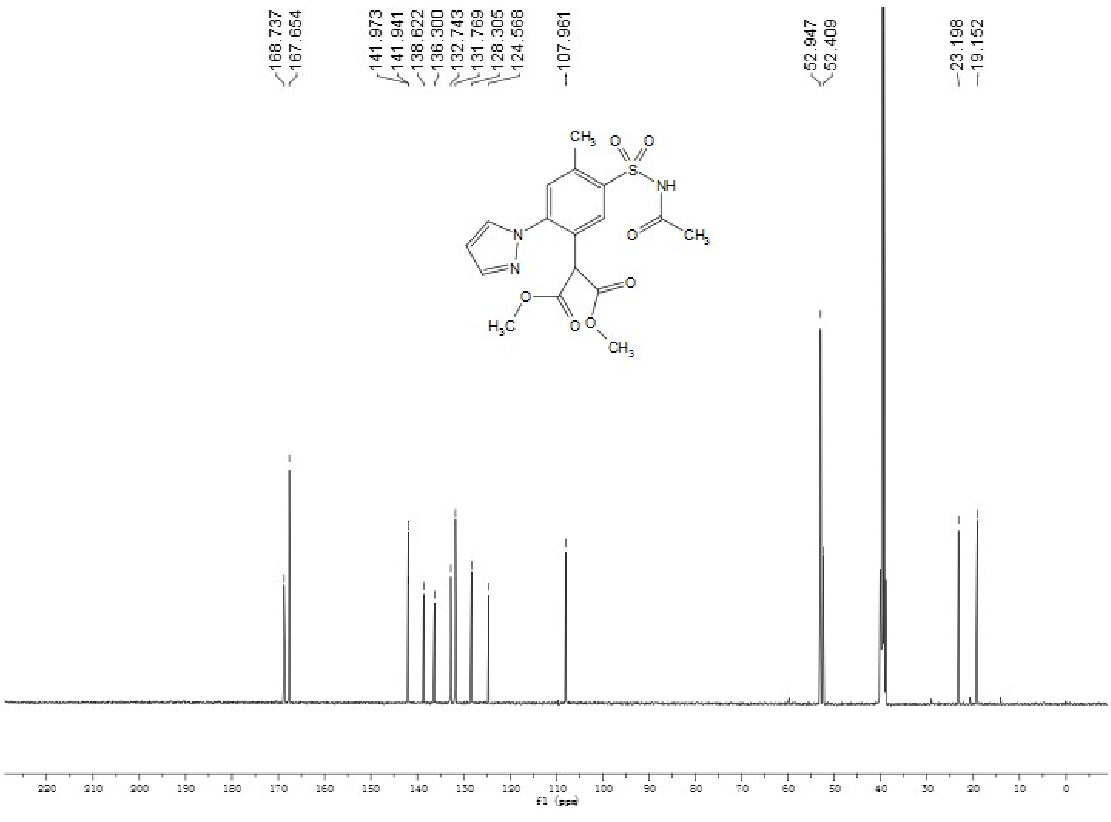

^{1}H NMR of compound **26**

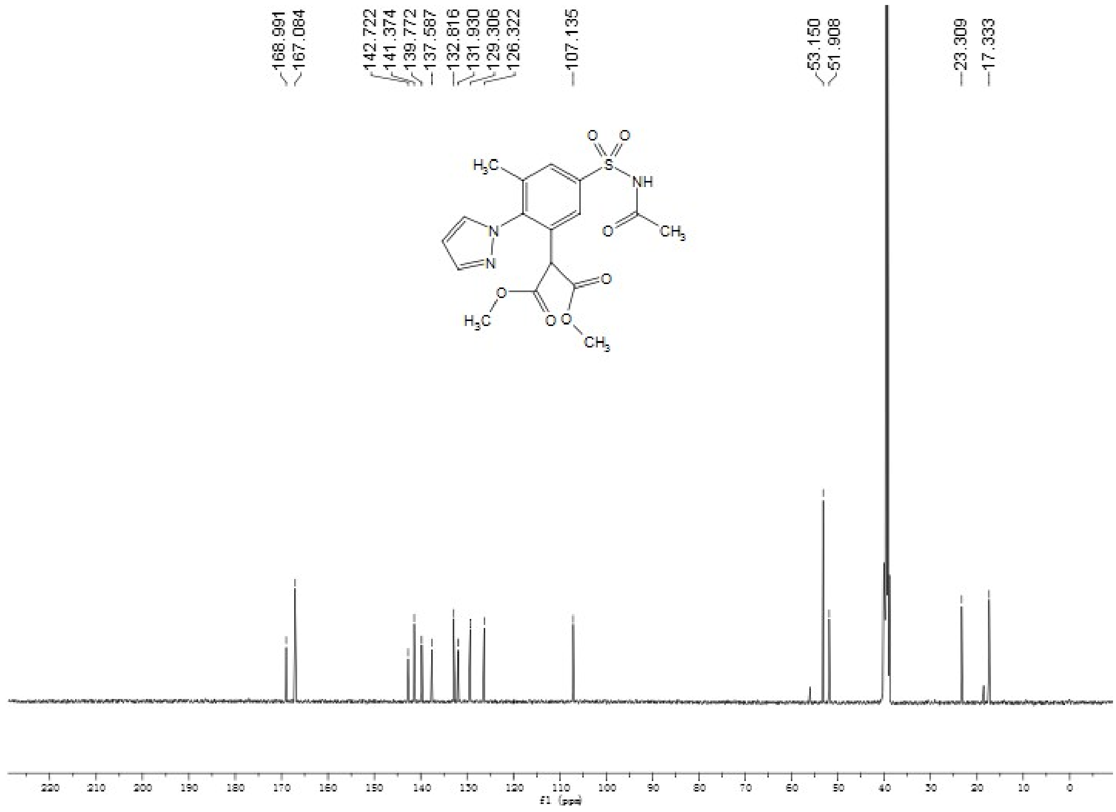

^{13}C NMR of compound **26**

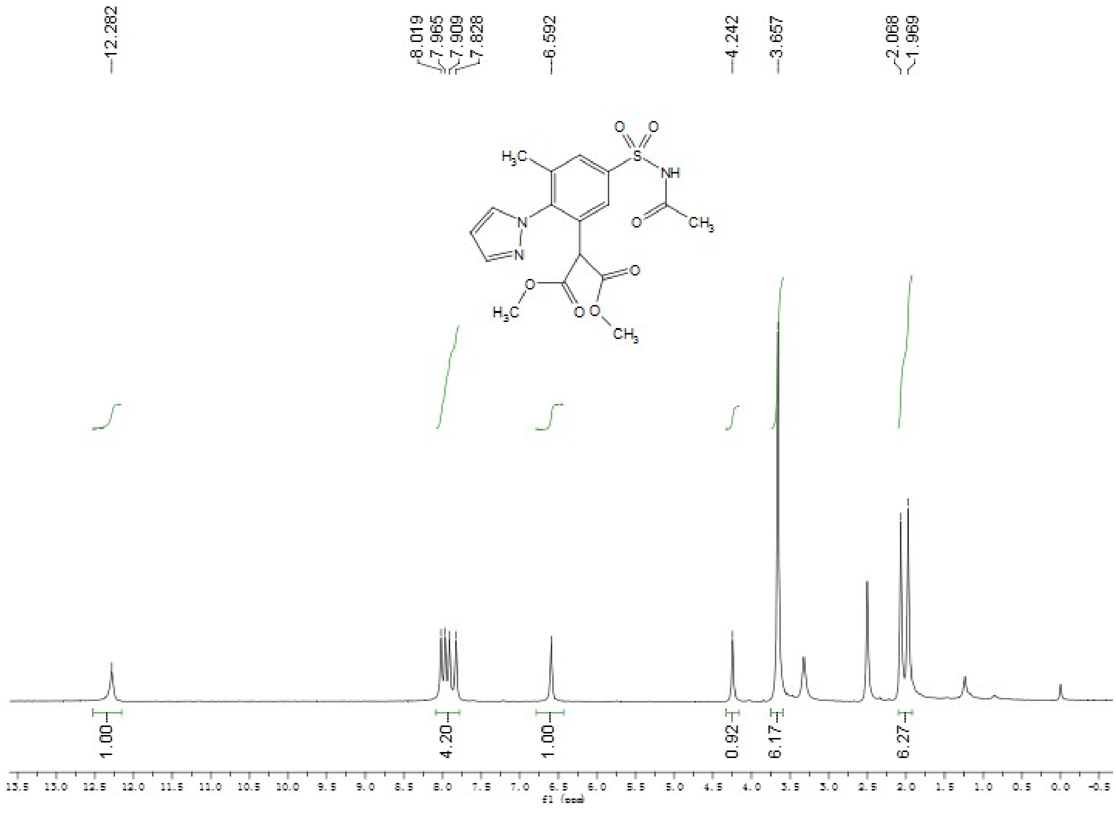

^{1}H NMR of compound **27**

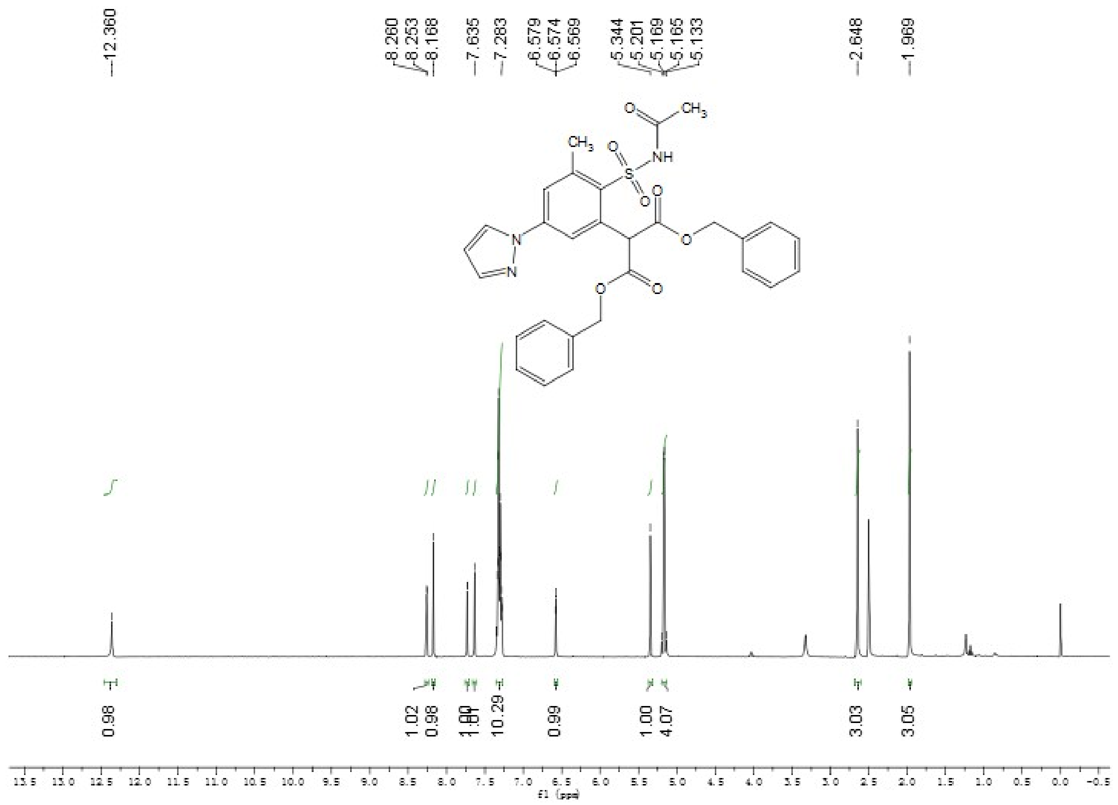

^{13}C NMR of compound **27**

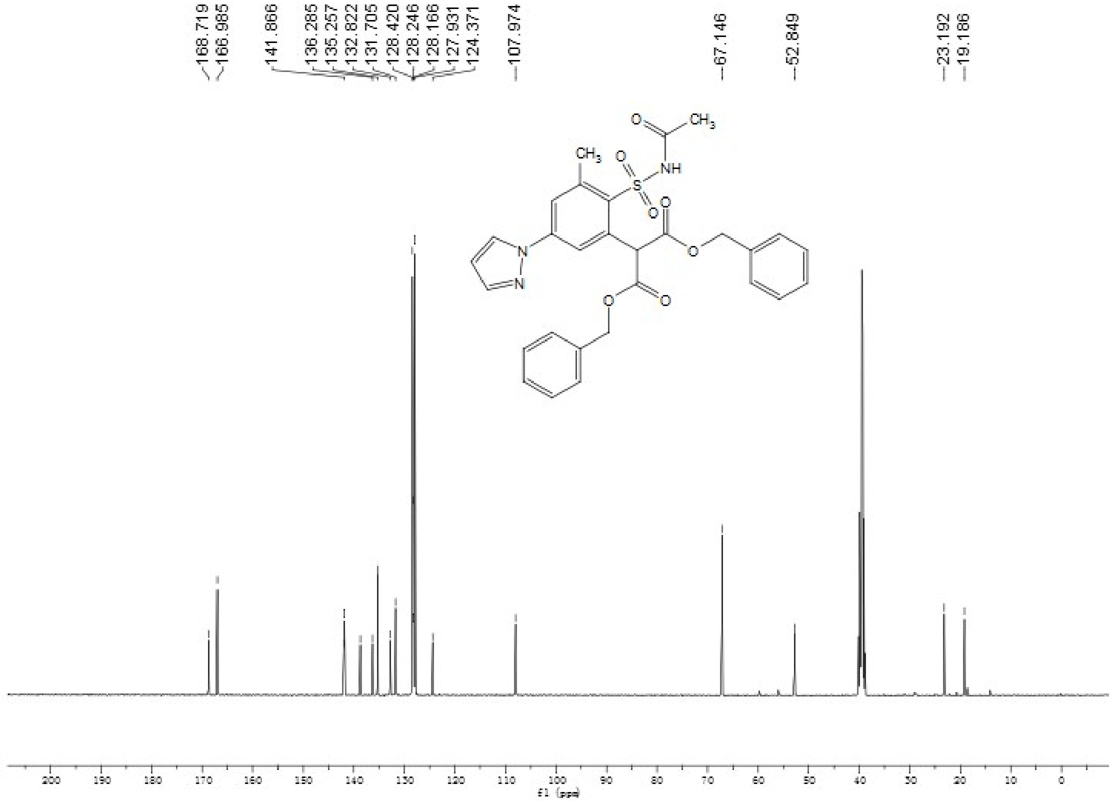

^{1}H NMR of compound **28**

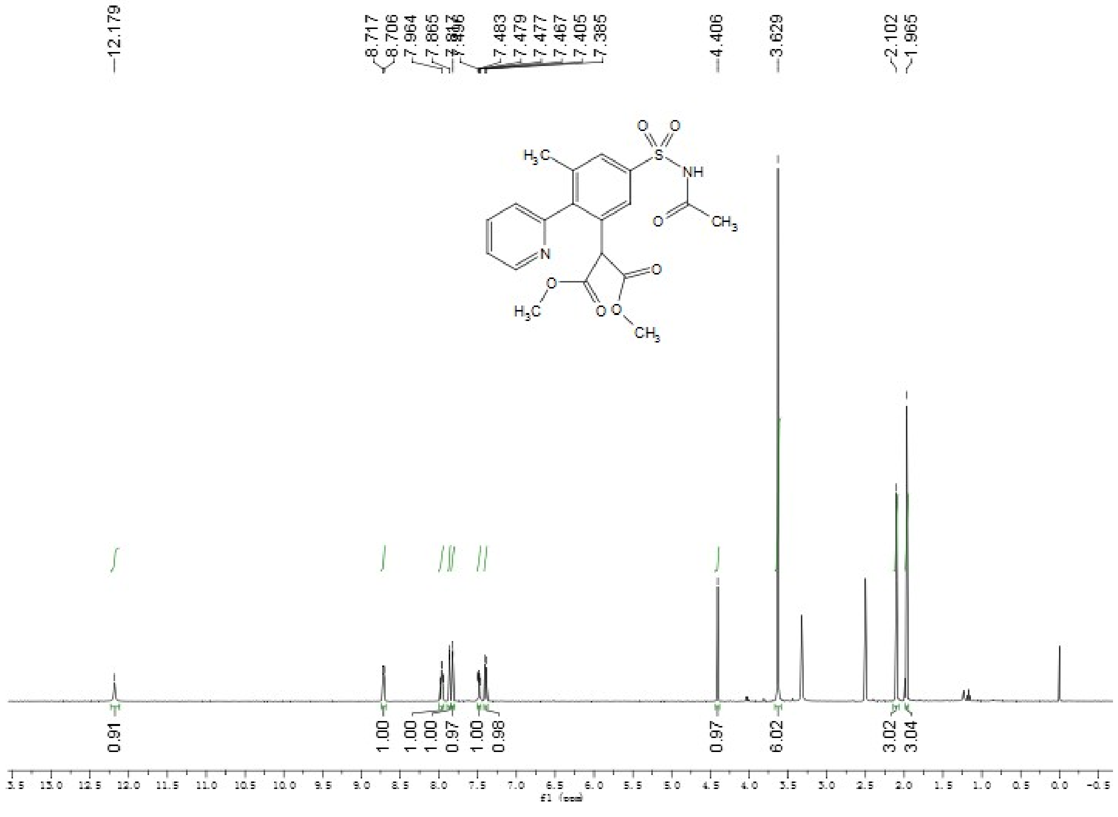

^{13}C NMR of compound **28**

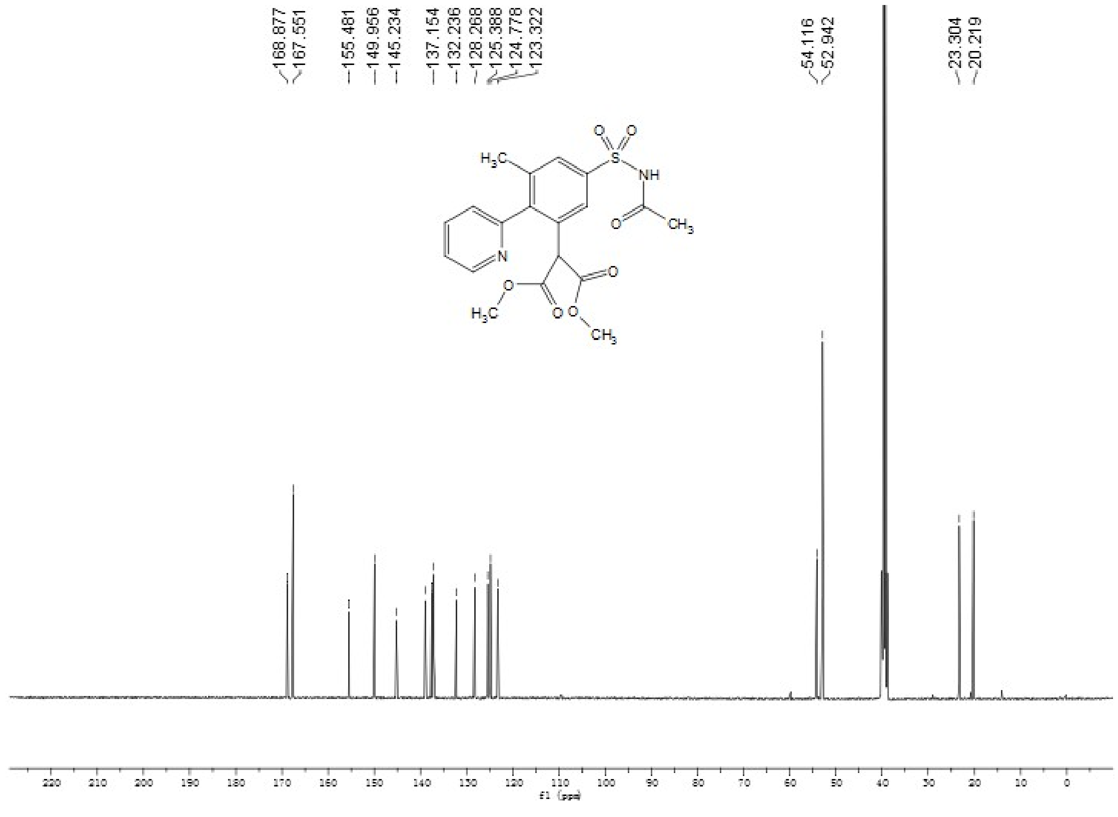

^{1}H NMR of compound **29**

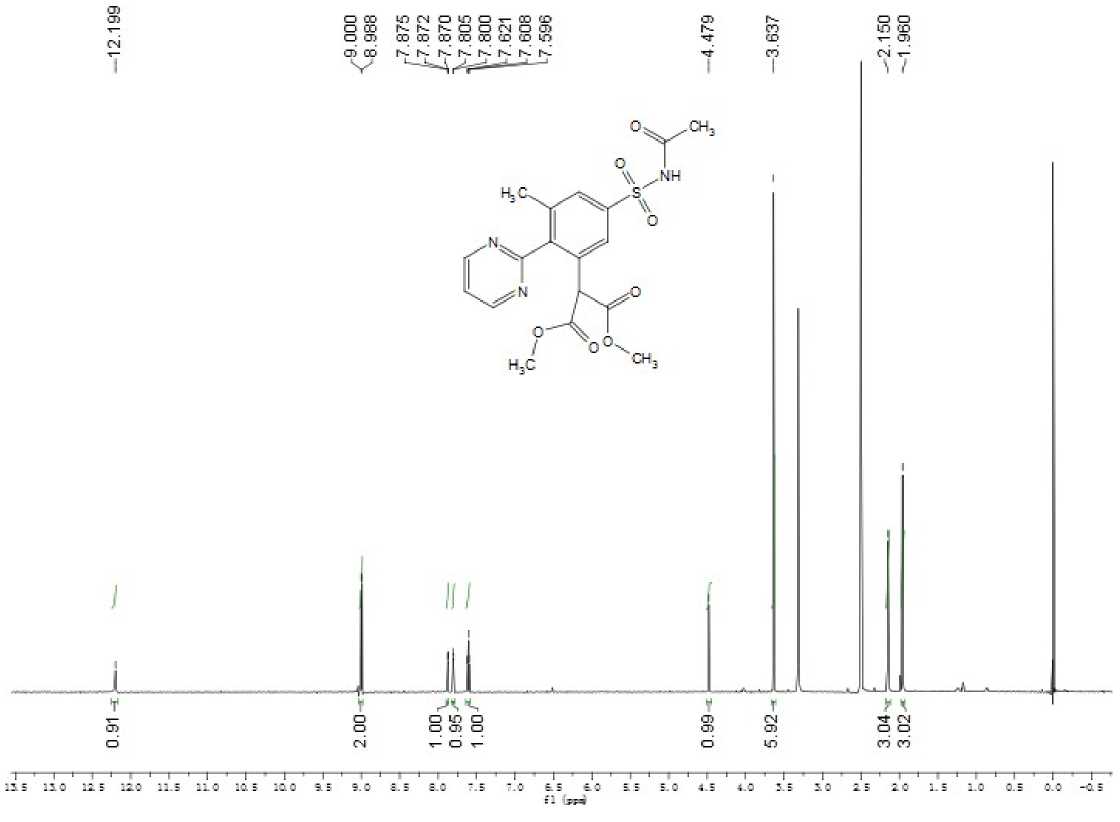

^{13}C NMR of compound **29**

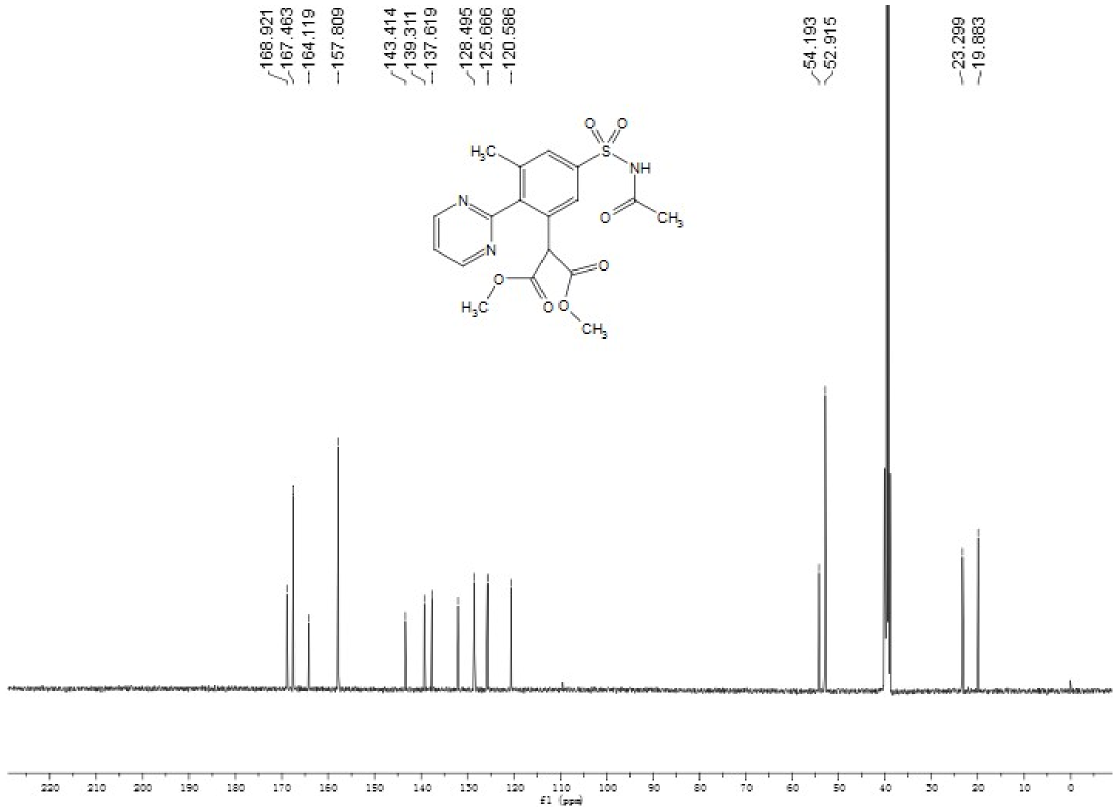

^{1}H NMR of compound **30**

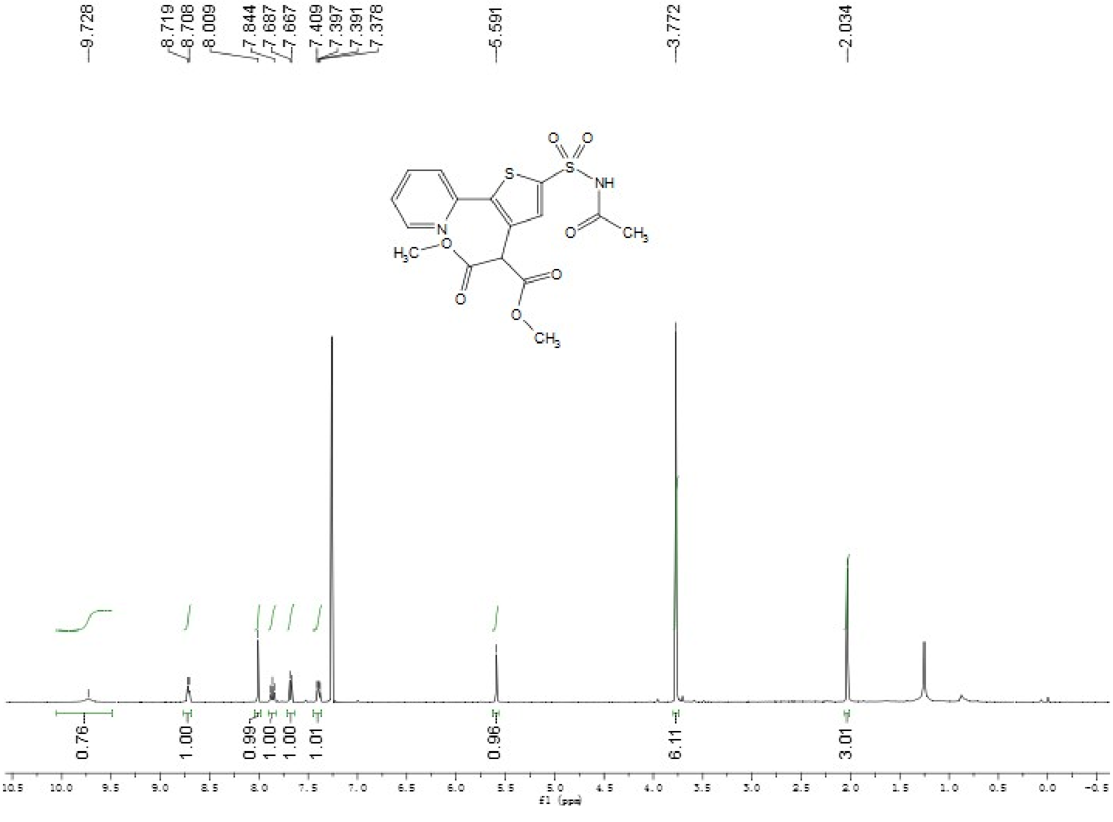

^{13}C NMR of compound **30**

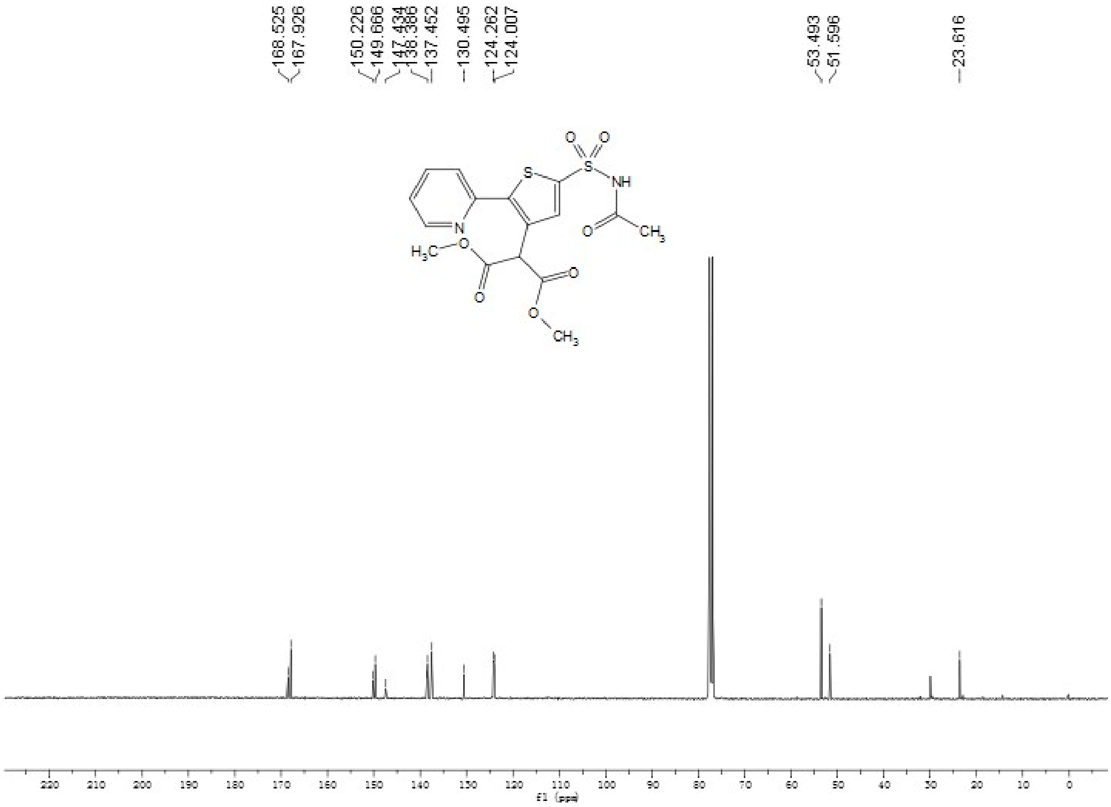

^{1}H NMR of compound **31**

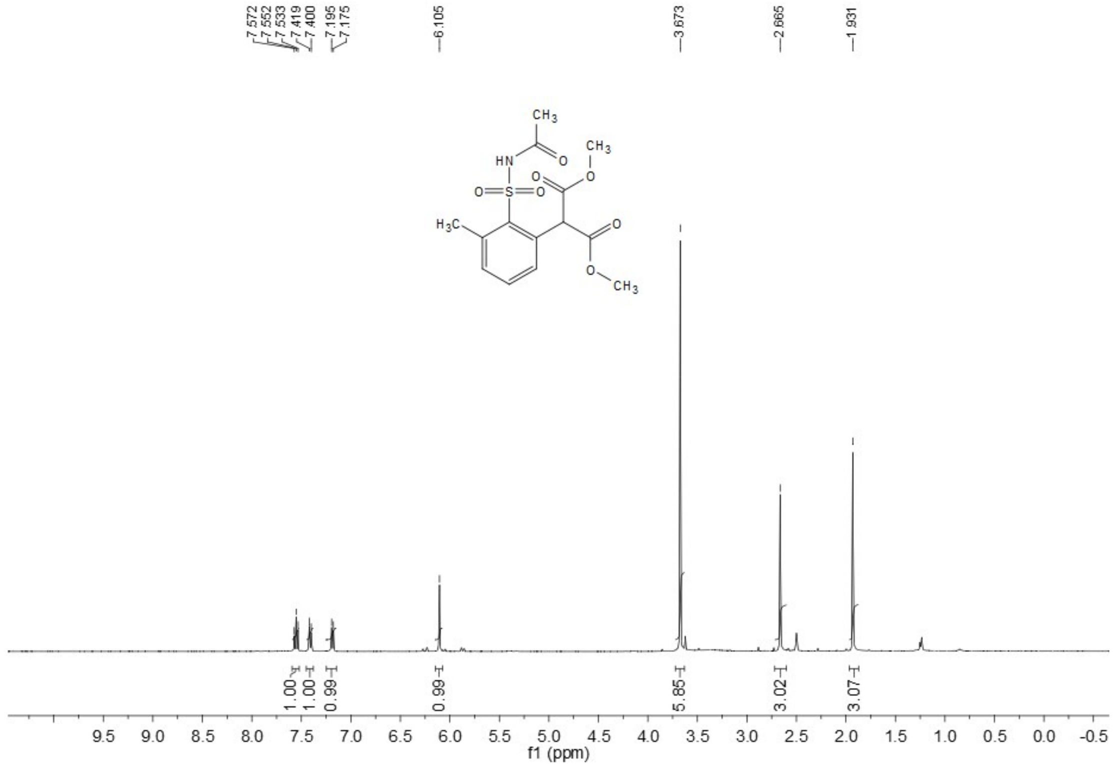

^{13}C NMR of compound **31**

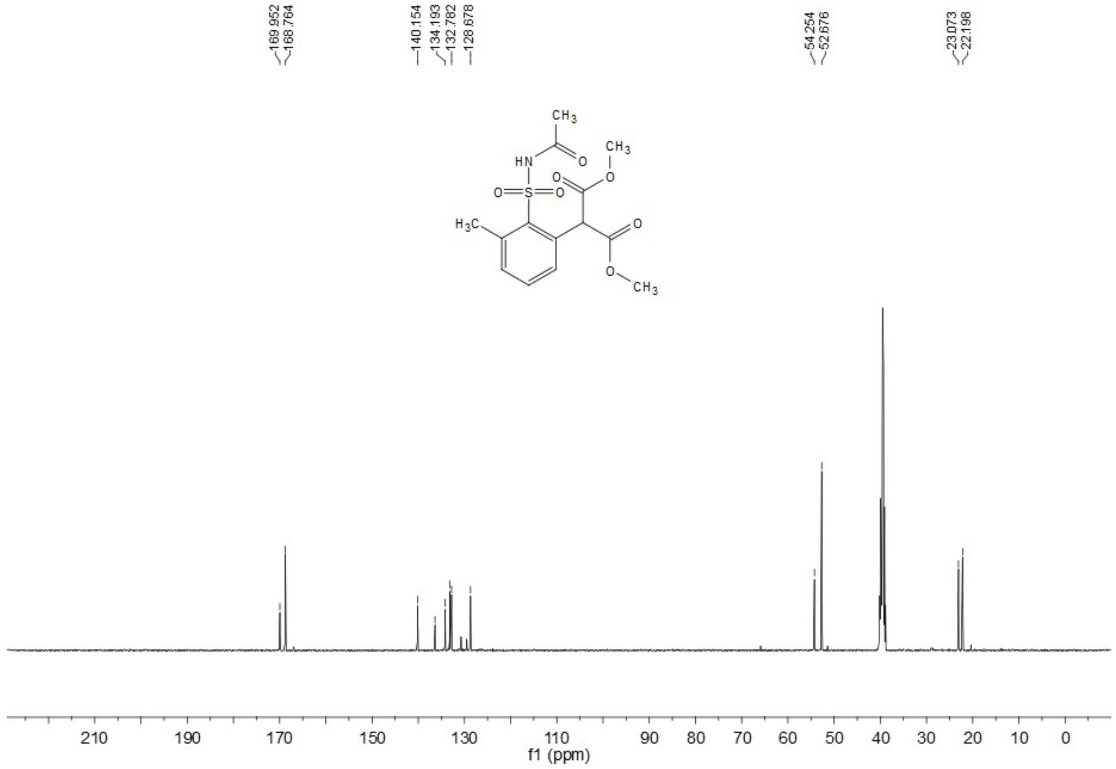

^{1}H NMR of compound **32**

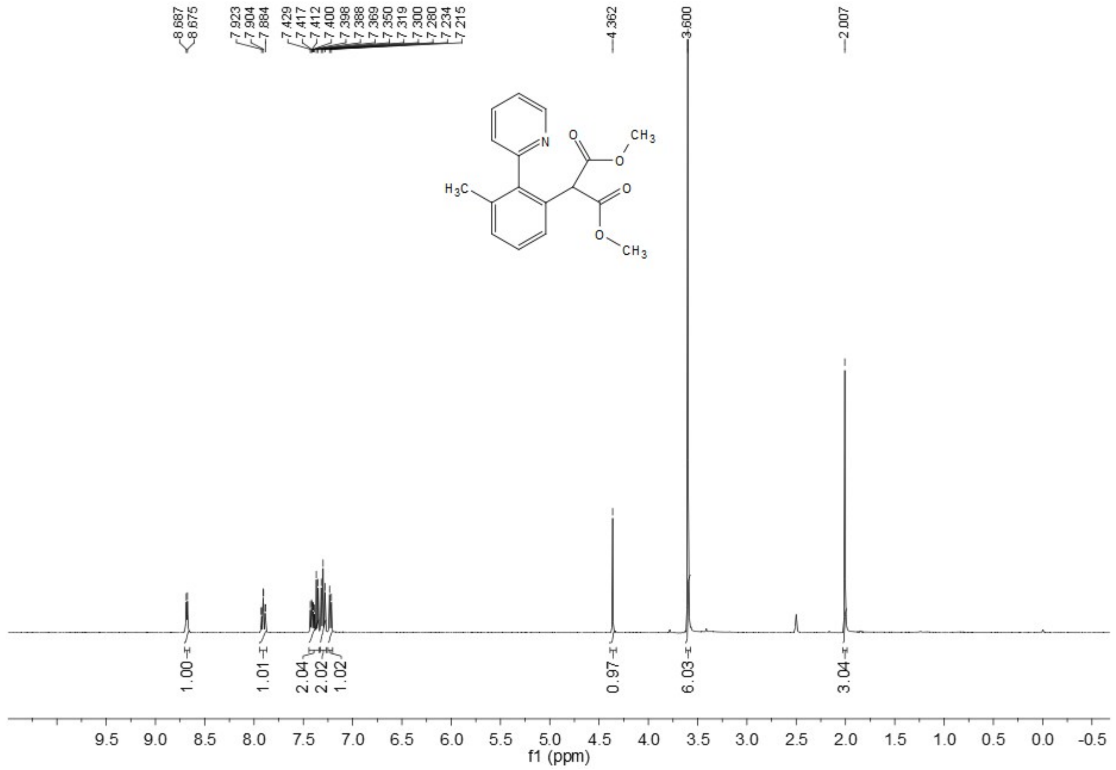

^{13}C NMR of compound **32**

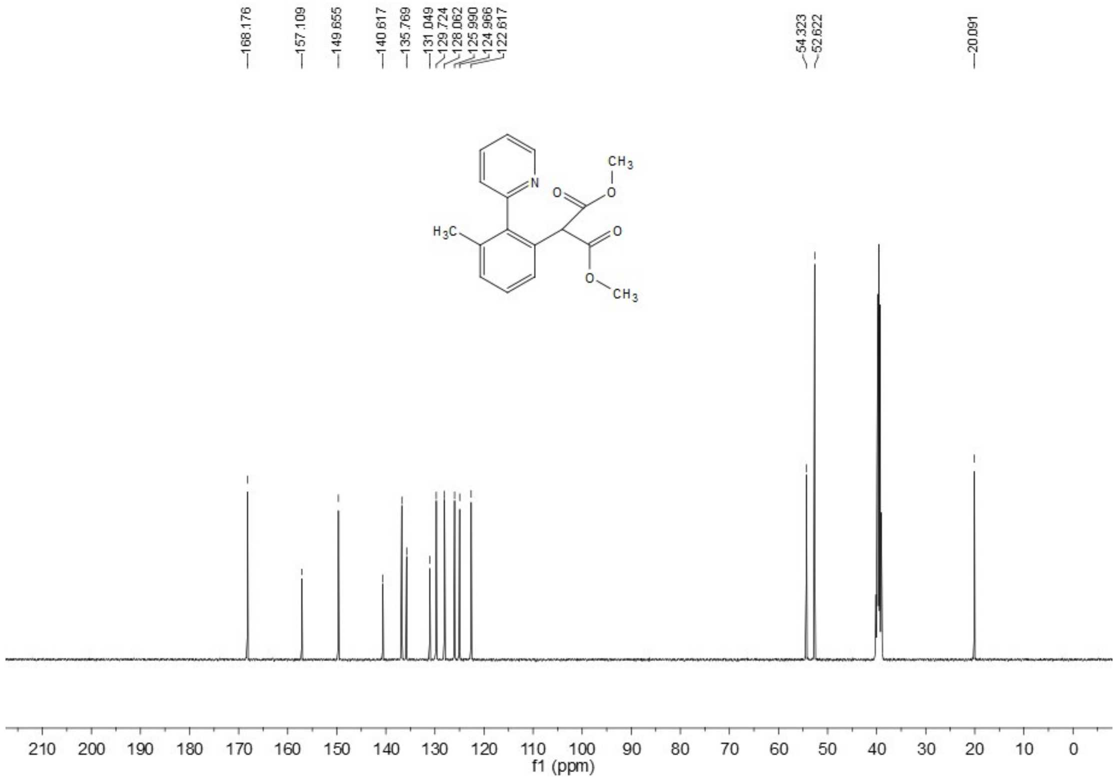

^{1}H NMR of compound **33**

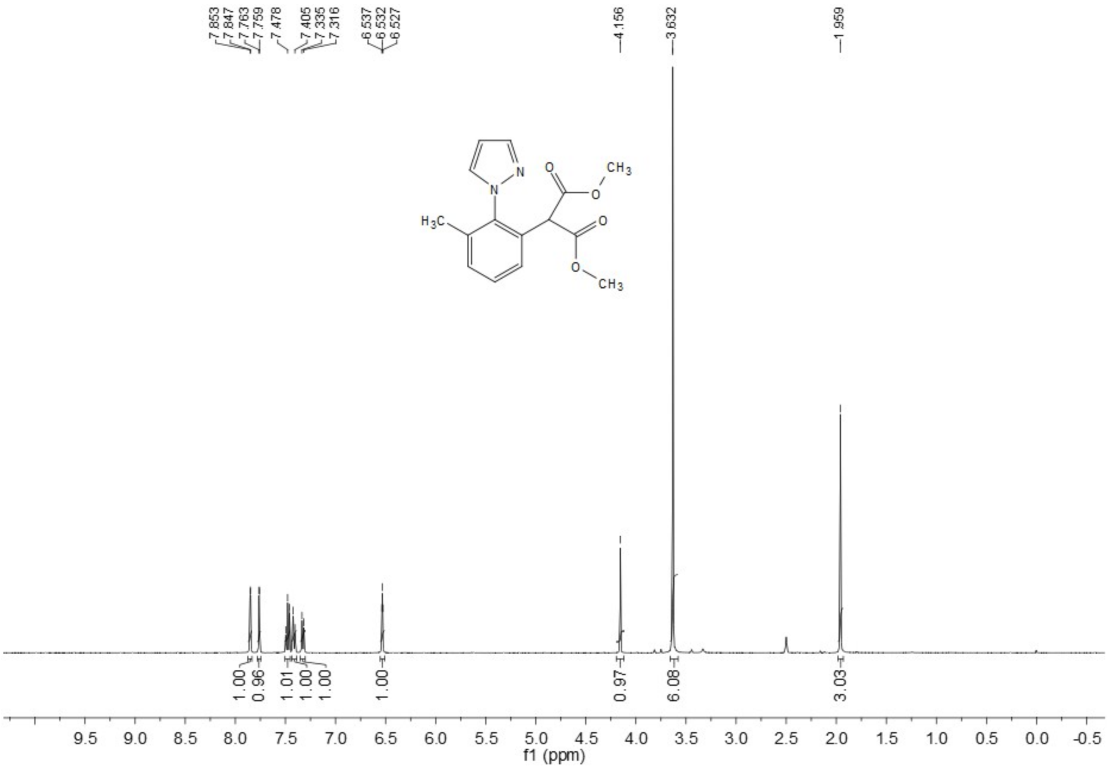

^{13}C NMR of compound **33**

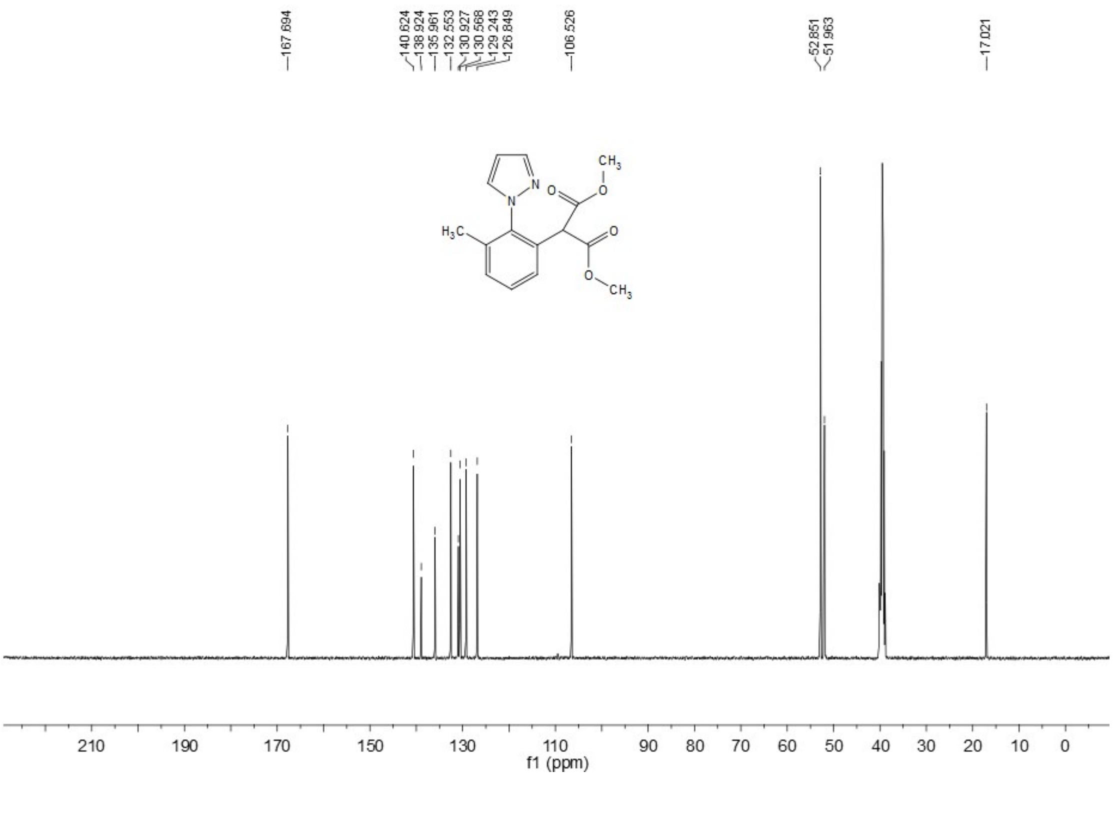

^{1}H NMR of compound **34**

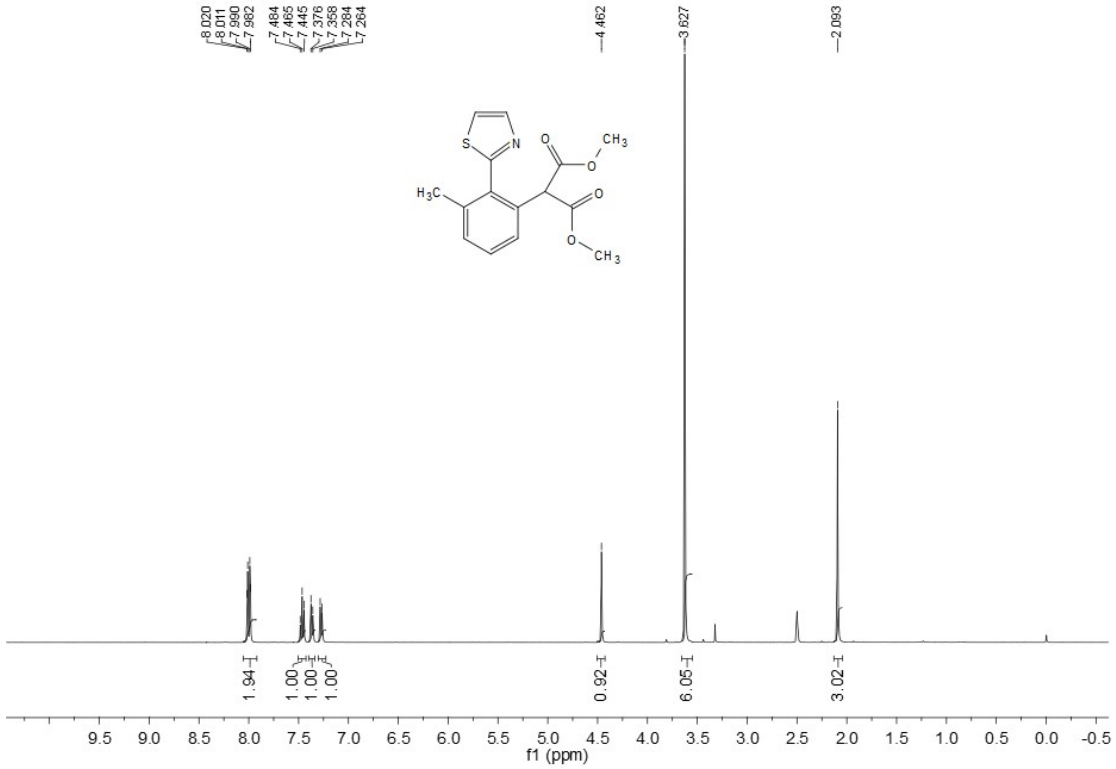

^{13}C NMR of compound **34**

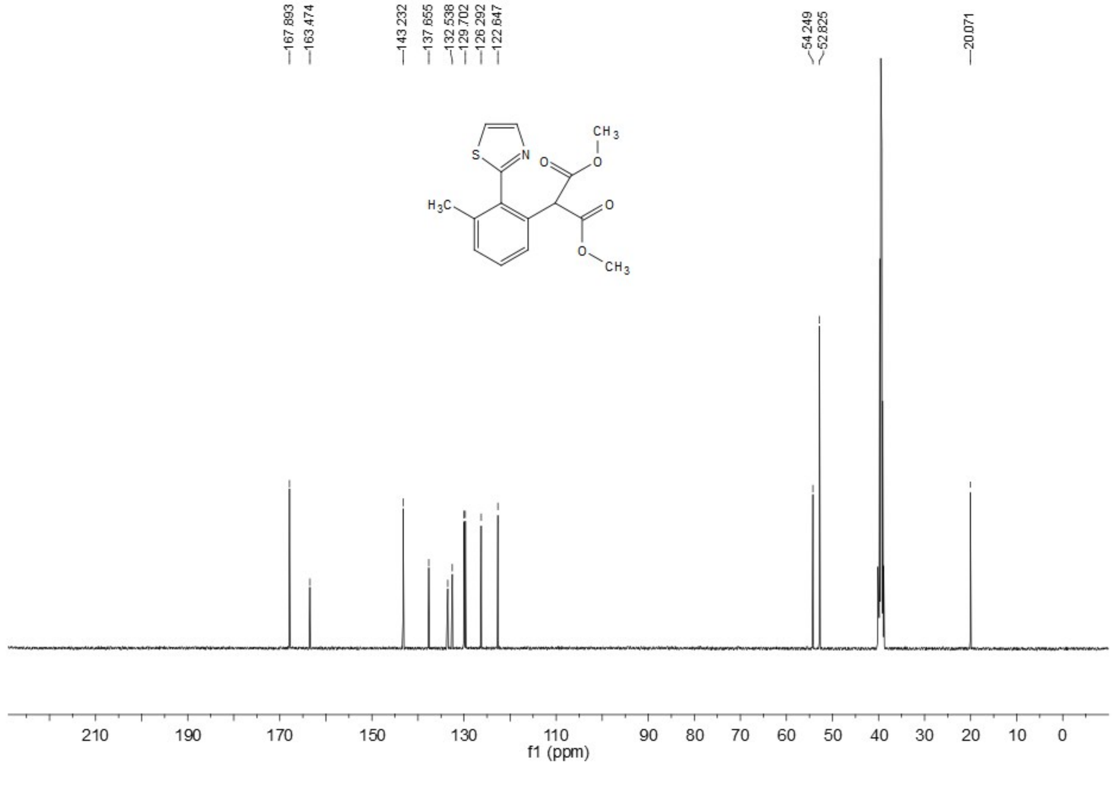

^{1}H NMR of compound **35**

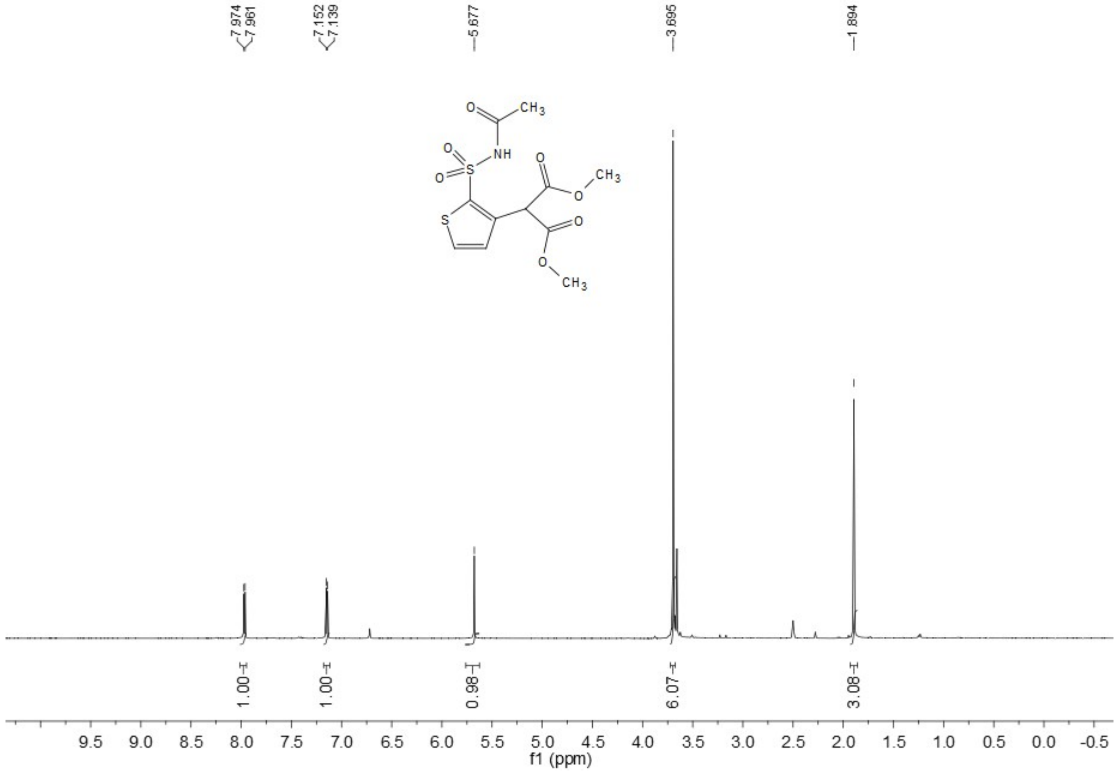

^{13}C NMR of compound **35**

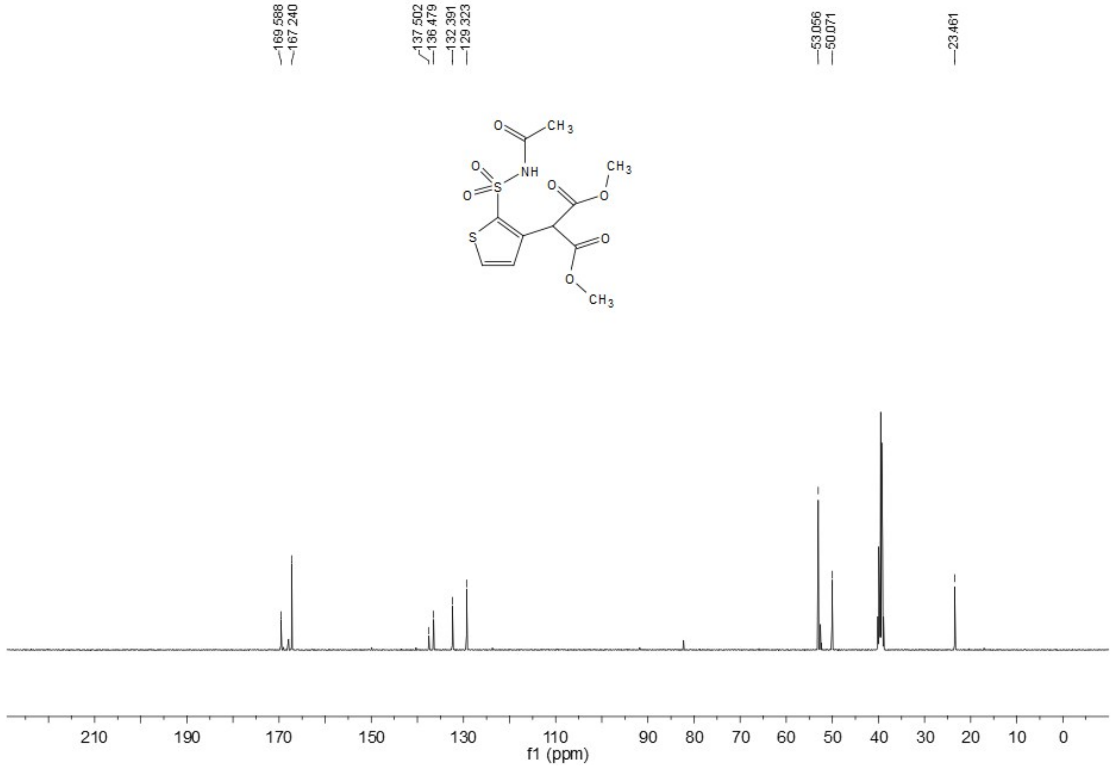

^{1}H NMR of compound **36**

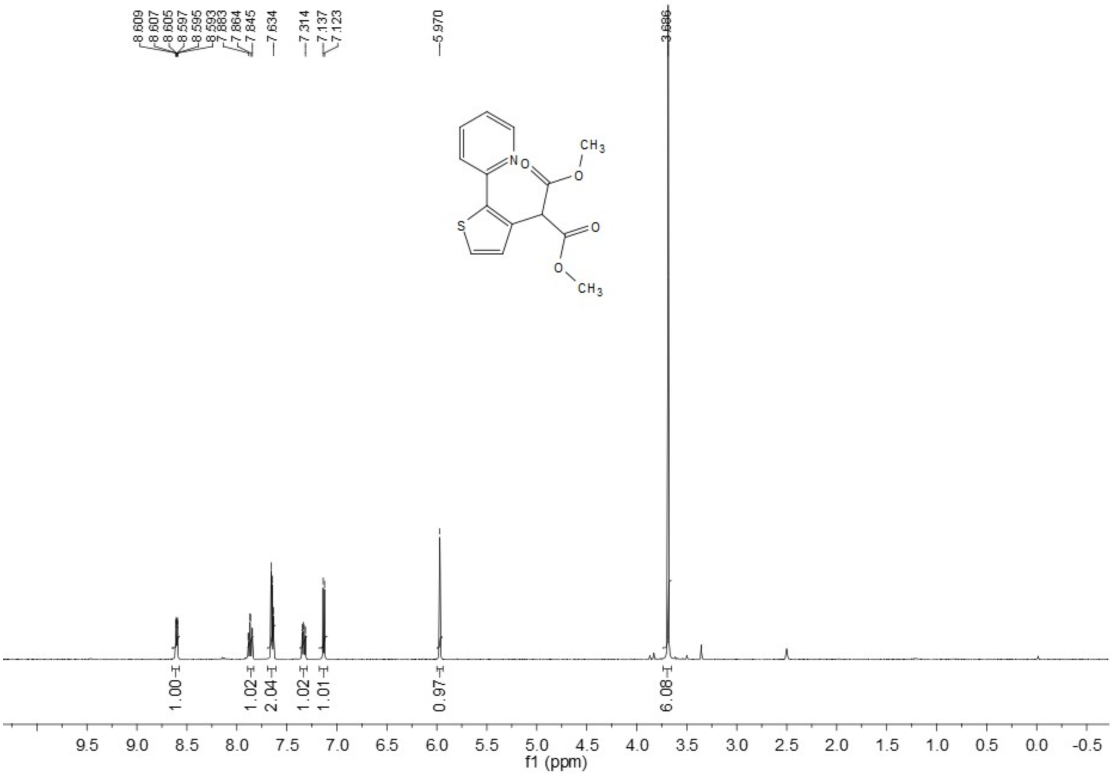

^{13}C NMR of compound **36**

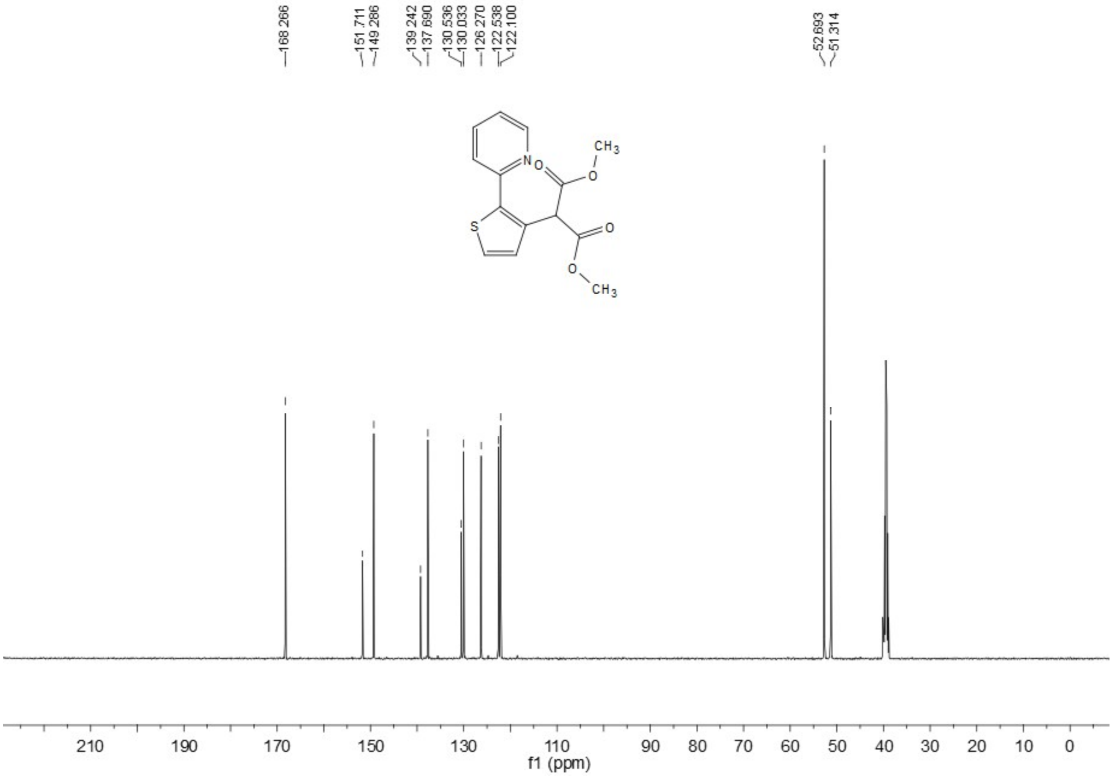

Table 1. Crystal Data and Structure Refinement for CCDC 1874184

Identification code	
Empirical formula	$C_{17}H_{15}F_3N_2O_8S_2$
Formula weight	496.43
Temperature/K	296
Crystal system	triclinic
Space group	P-1
a/Å	8.0043(3)
b/Å	10.7993(4)
c/Å	12.4643(5)
α/°	95.266(3)
β/°	100.867(3)
γ/°	92.113(3)
Volume/Å^3	1052.02(7)
Z	2
ρ_{calc}/g cm^{-3}	1.567
μ/mm^{-1}	2.994
F(000)	508.0
Crystal size/mm^3	0.5 × 0.35 × 0.2
Radiation	CuKα (λ = 1.54184)
2Θ range for data collection/°	7.258 to 142.632
Index ranges	-9 <= h <= 9, -13 <= k <= 12, -9 <= l <= 15
Reflections collected	6999
Independent reflections	3965 [R_{int} = 0.0208, R_{sigma} = 0.0289]
Data/restraints/parameters	3965/0/292
Goodness-of-fit on F^2	1.058
Final R indexes [I>=2σ (I)]	R_1 = 0.0459, wR_2 = 0.1222
Final R indexes [all data]	R_1 = 0.0500, wR_2 = 0.1269
Largest diff. peak/hole / e Å^{-3}	0.325 / -0.526

Table 2 Fractional Atomic Coordinates and Equivalent Isotropic Displacement Parameters (Å^2) for CCDC 1874184 . U_{eq} is defined as 1/3 of of the trace of the orthogonalised U_{IJ} tensor.

Atom	*x*	*y*	*z*	U(eq)
S1	0.02623(7)	0.14071(4)	0.63125(4)	0.04358(17)
S2	0.33572(9)	0.51841(6)	1.12185(5)	0.0603(2)
O1	-0.0473(2)	0.65852(14)	0.73040(15)	0.0521(4)
O2	0.1527(2)	0.80843(13)	0.73465(14)	0.0535(4)
C3	0.1924(2)	0.45977(17)	0.90229(16)	0.0354(4)
O4	0.0809(3)	0.12178(14)	0.87593(14)	0.0583(4)
C5	0.1344(3)	0.37945(17)	0.71170(16)	0.0365(4)
N6	0.1759(2)	0.04143(15)	0.64239(15)	0.0419(4)
C7	0.1106(3)	0.70203(17)	0.74056(16)	0.0380(4)
C8	0.0986(3)	0.26100(17)	0.73879(17)	0.0376(4)
C9	0.1845(2)	0.47945(17)	0.79123(15)	0.0333(4)
C10	0.2415(2)	0.60413(17)	0.75802(15)	0.0344(4)
O11	0.2157(2)	0.60060(19)	0.56617(13)	0.0631(5)
O12	0.0147(3)	0.19552(15)	0.53099(13)	0.0614(5)
O13	-0.1190(2)	0.07580(16)	0.65382(17)	0.0627(5)
C14	0.1548(3)	0.34031(19)	0.92863(17)	0.0422(5)
C15	0.3225(2)	0.58484(18)	0.65673(16)	0.0368(4)
C16	0.2452(3)	0.55786(19)	0.99384(16)	0.0384(4)
O17	0.3847(3)	0.17118(16)	0.60981(17)	0.0661(5)
F18	0.1538(4)	-0.04651(15)	0.94479(17)	0.1092(8)
N19	0.2301(3)	0.67674(17)	0.98826(16)	0.0539(5)
C20	0.1147(3)	0.24218(18)	0.84863(18)	0.0422(5)
O21	0.4636(2)	0.5549(2)	0.65996(15)	0.0760(6)
C22	0.3399(3)	0.0682(2)	0.6273(2)	0.0496(5)
F23	0.2501(4)	0.12213(18)	1.03902(16)	0.1279(10)
F24	0.3440(3)	0.0646(2)	0.8950(2)	0.1098(7)
C25	0.3549(4)	0.6714(3)	1.1703(2)	0.0590(6)
C26	0.2928(4)	0.7399(2)	1.0893(2)	0.0611(7)
C27	0.2066(5)	0.0682(2)	0.9386(2)	0.0747(9)
C28	-0.1765(4)	0.7493(3)	0.7061(3)	0.0705(8)
C29	0.2846(4)	0.5872(4)	0.4664(2)	0.0865(11)
C1	0.4527(4)	-0.0401(3)	0.6360(3)	0.0774(9)

Table 3 Anisotropic Displacement Parameters (Å^2) for CCDC 1874184 . The Anisotropic displacement factor exponent takes the form: $-2\pi^2[h^2a^{*2}U_{11}+2hka^*b^*U_{12}+...]$.

Atom	U11	U22	U33	U23	U13	U12
S1	0.0478(3)	0.0269(3)	0.0507(3)	0.0003(2)	-0.0018(2)	0.0003(2)
S2	0.0828(5)	0.0521(4)	0.0400(3)	0.0090(2)	-0.0054(3)	0.0048(3)
O1	0.0463(8)	0.0369(8)	0.0770(11)	0.0158(7)	0.0160(8)	0.0076(6)
O2	0.0713(11)	0.0249(7)	0.0680(10)	0.0102(7)	0.0199(8)	0.0036(7)
C3	0.0397(10)	0.0296(9)	0.0380(10)	0.0059(7)	0.0084(8)	0.0055(7)
O4	0.0889(13)	0.0286(8)	0.0615(10)	0.0153(7)	0.0209(9)	-0.0013(8)
C5	0.0433(10)	0.0287(9)	0.0369(9)	0.0070(7)	0.0036(8)	0.0033(8)
N6	0.0497(10)	0.0229(7)	0.0533(10)	0.0046(7)	0.0105(8)	0.0000(7)
C7	0.0521(11)	0.0273(9)	0.0370(10)	0.0040(7)	0.0138(8)	0.0035(8)
C8	0.0432(10)	0.0262(9)	0.0425(10)	0.0031(8)	0.0063(8)	0.0033(7)
C9	0.0368(9)	0.0258(9)	0.0379(10)	0.0058(7)	0.0069(7)	0.0044(7)
C10	0.0412(10)	0.0269(9)	0.0348(9)	0.0044(7)	0.0060(8)	0.0008(7)
O11	0.0584(10)	0.0989(14)	0.0358(8)	0.0111(8)	0.0099(7)	0.0388(10)
O12	0.0893(13)	0.0376(8)	0.0470(9)	0.0028(7)	-0.0126(8)	0.0049(8)
O13	0.0447(9)	0.0448(9)	0.0932(14)	-0.0031(9)	0.0058(9)	-0.0052(7)
C14	0.0568(12)	0.0335(10)	0.0390(10)	0.0095(8)	0.0129(9)	0.0050(9)
C15	0.0388(10)	0.0324(9)	0.0393(10)	0.0062(8)	0.0069(8)	0.0024(7)
C16	0.0440(11)	0.0352(10)	0.0367(10)	0.0060(8)	0.0078(8)	0.0041(8)
O17	0.0707(11)	0.0444(9)	0.0874(13)	0.0083(9)	0.0278(10)	-0.0114(8)
F18	0.208(3)	0.0362(8)	0.0901(13)	0.0279(9)	0.0348(15)	0.0165(11)

N19	0.0814(14)	0.0350(9)	0.0418(10)	0.0002(8)	0.0043(9)	0.0055(9)
C20	0.0542(12)	0.0260(9)	0.0491(11)	0.0102(8)	0.0136(9)	0.0027(8)
O21	0.0458(10)	0.137(2)	0.0503(10)	0.0191(11)	0.0122(8)	0.0267(11)
C22	0.0556(13)	0.0395(11)	0.0548(13)	0.0017(10)	0.0160(10)	-0.0026(10)
F23	0.246(3)	0.0628(11)	0.0585(11)	0.0093(9)	-0.0176(14)	0.0354(15)
F24	0.1069(16)	0.1094(17)	0.1211(18)	0.0469(14)	0.0184(14)	0.0410(13)
C25	0.0702(16)	0.0610(15)	0.0400(12)	-0.0045(10)	0.0029(11)	-0.0087(12)
C26	0.0877(19)	0.0409(13)	0.0494(13)	-0.0059(10)	0.0057(13)	-0.0009(12)
C27	0.135(3)	0.0376(13)	0.0553(15)	0.0170(11)	0.0199(17)	0.0170(15)
C28	0.0571(15)	0.0588(16)	0.103(2)	0.0234(15)	0.0225(15)	0.0247(13)
C29	0.085(2)	0.144(3)	0.0381(13)	0.0123(16)	0.0199(13)	0.056(2)
C1	0.0636(17)	0.0634(18)	0.116(3)	0.0196(17)	0.0365(17)	0.0170(14)

Table 4 Bond Lengths for CCDC 1874184.

Atom	Atom	Length/Å
S1	N6	1.6320(18)
S1	C8	1.775(2)
S1	O12	1.4201(17)
S1	O13	1.4218(18)
S2	C16	1.722(2)
S2	C25	1.697(3)
O1	C7	1.312(3)
O1	C28	1.460(3)
O2	C7	1.198(2)
C3	C9	1.410(3)
C3	C14	1.396(3)
C3	C16	1.472(3)
O4	C20	1.403(2)
O4	C27	1.337(4)
C5	C8	1.386(3)
C5	C9	1.390(3)
N6	C22	1.385(3)
C7	C10	1.517(3)
C8	C20	1.385(3)
C9	C10	1.523(2)
C10	C15	1.526(3)
O11	C15	1.310(3)
O11	C29	1.450(3)
C14	C20	1.371(3)
C15	O21	1.180(3)
C16	N19	1.301(3)
O17	C22	1.207(3)
F18	C27	1.308(3)
N19	C26	1.372(3)
C22	C1	1.503(4)
F23	C27	1.309(4)
F24	C27	1.315(4)
C25	C26	1.338(4)

Table 5 Bond Angles for CCDC 1874184.

Atom	Atom	Atom	Angle/°
N6	S1	C8	105.00(9)
O12	S1	N6	110.03(11)
O12	S1	C8	106.88(9)
O12	S1	O13	119.73(12)
O13	S1	N6	105.33(10)
O13	S1	C8	108.97(11)
C25	S2	C16	89.66(11)
C7	O1	C28	115.13(18)
C9	C3	C16	123.52(17)
C14	C3	C9	119.04(18)
C14	C3	C16	117.40(17)
C27	O4	C20	117.8(2)
C8	C5	C9	121.99(18)
C22	N6	S1	123.92(15)
O1	C7	C10	114.10(16)
O2	C7	O1	124.8(2)
O2	C7	C10	121.12(19)
C5	C8	S1	118.69(15)
C20	C8	S1	122.57(15)
C20	C8	C5	118.70(18)
C3	C9	C10	121.54(17)
C5	C9	C3	118.48(17)
C5	C9	C10	119.83(17)
C7	C10	C9	117.72(16)
C7	C10	C15	109.24(15)
C9	C10	C15	110.56(15)
C15	O11	C29	115.60(19)
C20	C14	C3	121.01(19)
O11	C15	C10	112.25(17)
O21	C15	C10	123.44(19)
O21	C15	O11	124.27(19)
C3	C16	S2	119.83(14)
N19	C16	S2	114.44(16)
N19	C16	C3	125.73(18)
C16	N19	C26	109.6(2)
C8	C20	O4	118.60(19)
C14	C20	O4	120.72(19)
C14	C20	C8	120.60(18)
N6	C22	C1	114.2(2)
O17	C22	N6	121.3(2)
O17	C22	C1	124.5(2)
C26	C25	S2	109.58(18)

C25	C26	N19	116.7(2)
F18	C27	O4	107.6(3)
F18	C27	F23	107.5(2)
F18	C27	F24	108.0(3)
F23	C27	O4	114.0(3)
F23	C27	F24	108.0(3)
F24	C27	O4	111.5(2)

Table 6 Hydrogen Atom Coordinates (Å) and Isotropic Displacement Parameters (Å^2) for CCDC 1874184.

Atom	*x*	*y*	*z*	U(eq)
H5	0.1246	0.3924	0.6381	0.044
H6	0.1515	-0.0319	0.6586	0.05
H10	0.3323	0.6401	0.8181	0.041
H14	0.157	0.3271	1.0016	0.051
H25	0.4014	0.7027	1.2422	0.071
H26	0.2923	0.8262	1.1009	0.073
H28A	-0.1778	0.7739	0.6339	0.106
H28B	-0.1502	0.8211	0.759	0.106
H28C	-0.2862	0.7127	0.7094	0.106
H29A	0.1979	0.6018	0.405	0.13
H29B	0.3223	0.5044	0.4556	0.13
H29C	0.3791	0.6464	0.4725	0.13
H1A	0.4503	-0.0752	0.7037	0.116
H1B	0.5673	-0.0121	0.6343	0.116
H1C	0.4124	-0.1022	0.5755	0.116

Datablock 20180911dy-655-430 - ellipsoid plot

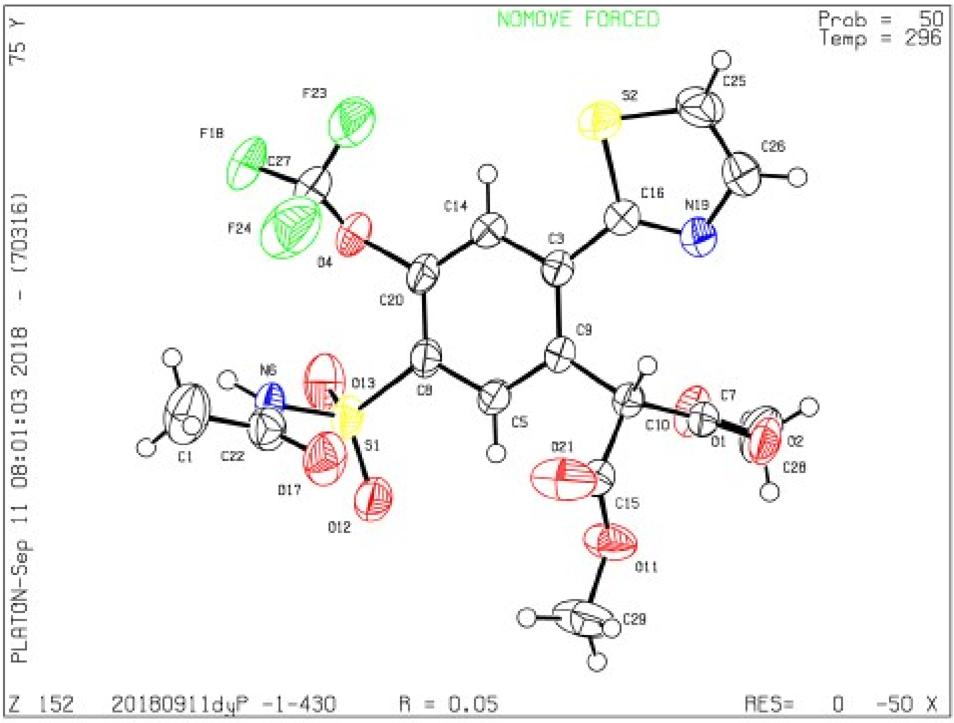

Table 1. Crystal Data and Structure Refinement for CCDC 1874186

Identification code	
Empirical formula	$C_{17}H_{18}F_3N_2O_9S_2$
Formula weight	515.45
Temperature/K	110
Crystal system	triclinic
Space group	P-1
a/Å	9.3787(4)
b/Å	10.6208(5)
c/Å	11.4671(6)
α/°	85.869(4)
β/°	72.343(4)
γ/°	71.018(4)
Volume/Å^3	1028.77(9)
Z	2
ρ_{calc}/g cm^{-3}	1.664
μ/mm^{-1}	3.121
F(000)	530.0
Crystal size/mm^3	0.28 × 0.22 × 0.08
Radiation	CuKα (λ = 1.54184)
2Θ range for data collection/°	8.094 to 142.538
Index ranges	-11 <= h <= 11, -12 <= k <= 12, -14 <= l <= 11
Reflections collected	6843
Independent reflections	3854 [R_{int} = 0.0305, R_{sigma} = 0.0406]
Data/restraints/parameters	3854/1/304
Goodness-of-fit on F^2	1.027
Final R indexes [I>=2σ (I)]	R_1 = 0.0449, wR_2 = 0.1186
Final R indexes [all data]	R_1 = 0.0523, wR_2 = 0.1260
Largest diff. peak/hole / e Å^{-3}	0.454 / -0.704

Table 2 Fractional Atomic Coordinates and Equivalent Isotropic Displacement Parameters (Å^2) for CCDC 1874186 . U_{eq} is defined as 1/3 of of the trace of the orthogonalised U_{IJ} tensor.

**Atom**	***x***	***y***	***z***	**U(eq)**
S1	0.05887(7)	0.5345(6)	0.06837(6)	0.02160(17)
S2	-0.01830(8)	0.90168(7)	1.20508(6)	0.02501(18)
F1	-0.39715(19)	0.62231(18)	0.96646(16)	0.0346(4)
O2	-0.1890(2)	0.6799(2)	0.88818(17)	0.0273(4)
O3	0.6146(2)	0.62662(18)	0.68125(18)	0.0247(4)
O4	0.5268(2)	0.45234(18)	0.73672(19)	0.0263(4)
O5	0.4554(2)	0.71714(19)	0.46683(17)	0.0261(4)
O6	0.3301(2)	0.86916(19)	0.62139(17)	0.0270(4)
O7	-0.0106(2)	0.44240(19)	0.7557(2)	0.0324(5)
O8	0.2025(2)	0.4827(2)	0.5878(2)	0.0355(5)
O9	0.0637(2)	0.7714(2)	0.53696(18)	0.0297(5)
F10	-0.3744(2)	0.7815(2)	1.0529(2)	0.0596(7)
N11	-0.0760(2)	0.6328(2)	0.62511(19)	0.0191(4)
C12	0.1057(3)	0.7883(2)	0.9683(2)	0.0166(5)
N13	0.2419(3)	0.9081(2)	1.05011(19)	0.0204(5)
C14	0.2236(3)	0.9732(2)	1.1551(2)	0.0226(5)
C15	0.2323(3)	0.7406(2)	0.8646(2)	0.0174(5)
C16	0.1216(3)	0.8640(2)	1.0636(2)	0.0169(5)
C17	0.2225(3)	0.6700(2)	0.7713(2)	0.0168(5)
C18	0.5172(3)	0.5719(2)	0.6927(2)	0.0168(5)
C19	0.3785(3)	0.7561(3)	0.5828(2)	0.0204(5)
C20	0.0826(3)	0.6416(2)	0.7844(2)	0.0170(5)
C21	-0.0464(3)	0.6946(2)	0.8883(2)	0.0183(5)
C22	-0.0576(3)	0.7436(3)	0.5584(2)	0.0218(5)
C23	-0.0365(3)	0.7669(2)	0.9794(2)	0.0191(5)
F24	-0.2296(3)	0.5858(3)	1.0675(2)	0.0690(8)
C25	0.3653(3)	0.6342(2)	0.6581(2)	0.0185(5)
C26	0.6740(3)	0.3779(3)	0.7602(3)	0.0268(6)
C27	0.4825(4)	0.8201(3)	0.3826(3)	0.0330(7)
C28	-0.2944(3)	0.6669(3)	0.9932(3)	0.0246(6)
C29	0.0908(3)	0.9803(3)	1.2474(2)	0.0251(6)
C30	-0.1989(4)	0.8220(3)	0.5197(3)	0.0298(6)
O1	0.4452(4)	0.9656(3)	0.8084(3)	0.0596(8)

Table 3 Anisotropic Displacement Parameters (Å^2) for CCDC 1874186 . The Anisotropic displacement factor exponent takes the form: $-2\pi^2[h^2a^{*2}U_{11}+2hka^*b^*U_{12}+...]$.

Atom	U11	U22	U33	U23	U13	U12
S1	0.0161(3)	0.0194(3)	0.031(4)	-0.0076(2)	-0.0098(3)	-0.0035(2)
S2	0.0257(3)	0.0279(3)	0.0191(3)	-0.0062(3)	-0.0013(3)	-0.0089(3)
F1	0.0227(8)	0.0504(11)	0.0355(10)	-0.0083(8)	-0.0011(7)	-0.0228(8)
O2	0.0170(9)	0.0473(12)	0.0217(10)	-0.0017(8)	-0.0037(7)	-0.0168(8)
O3	0.0181(9)	0.0283(10)	0.0308(11)	0.0089(8)	-0.0101(8)	-0.0105(8)
O4	0.0182(9)	0.0191(9)	0.0426(12)	0.0016(8)	-0.0126(8)	-0.0041(7)
O5	0.0255(10)	0.0296(10)	0.0159(9)	-0.0020(7)	-0.0038(8)	-0.0007(8)
O6	0.0313(11)	0.0257(10)	0.0212(10)	-0.0022(8)	-0.0040(8)	-0.0081(8)
O7	0.0326(11)	0.0202(9)	0.0556(14)	0.0057(9)	-0.0268(10)	-0.0115(8)
O8	0.0158(9)	0.0404(12)	0.0475(13)	-0.0318(10)	-0.0060(9)	-0.0016(8)
O9	0.0290(11)	0.0407(11)	0.0238(10)	-0.0052(8)	-0.0020(8)	-0.0209(9)
F10	0.0232(10)	0.0748(15)	0.0744(15)	-0.0489(13)	0.0042(9)	-0.0138(10)
N11	0.0138(10)	0.0250(11)	0.0208(11)	-0.0020(8)	-0.0055(8)	-0.0082(8)
C12	0.0177(12)	0.0151(11)	0.0178(12)	0.0022(9)	-0.0081(10)	-0.0039(9)
N13	0.0244(11)	0.0185(10)	0.0154(10)	-0.0007(8)	-0.0053(9)	-0.0035(9)
C14	0.0315(14)	0.0174(12)	0.0233(13)	0.0034(10)	-0.0134(11)	-0.0091(11)
C15	0.0145(11)	0.0183(11)	0.0194(12)	-0.0010(9)	-0.0057(10)	-0.0044(9)

C16	0.0188(12)	0.0158(11)	0.0151(12)	0.0022(9)	-0.0066(9)	-0.0031(9)
C17	0.0143(11)	0.0151(11)	0.0194(12)	-0.0010(9)	-0.0048(10)	-0.0024(9)
C18	0.0121(11)	0.0210(12)	0.0145(11)	-0.0060(9)	-0.0007(9)	-0.0034(9)
C19	0.0157(12)	0.0272(13)	0.0174(12)	-0.0032(10)	-0.0068(10)	-0.0031(10)
C20	0.0153(12)	0.0168(11)	0.0198(12)	-0.0004(9)	-0.0080(10)	-0.0035(9)
C21	0.0139(11)	0.0230(12)	0.0191(12)	0.0019(10)	-0.0043(9)	-0.0083(10)
C22	0.0249(14)	0.0272(13)	0.0141(12)	-0.0061(10)	-0.0017(10)	-0.0115(11)
C23	0.0157(12)	0.0241(12)	0.0164(12)	0.0002(10)	-0.0033(9)	-0.0060(10)
F24	0.0584(14)	0.1017(19)	0.0766(17)	0.0693(15)	-0.0436(13)	-0.0567(14)
C25	0.0136(12)	0.0227(12)	0.0188(12)	-0.0061(10)	-0.0051(10)	-0.0034(9)
C26	0.0190(13)	0.0209(13)	0.0379(16)	0.0013(11)	-0.0109(12)	-0.0008(10)
C27	0.0361(16)	0.0386(16)	0.0189(14)	0.0043(12)	-0.0071(12)	-0.0064(13)
C28	0.0190(13)	0.0330(14)	0.0234(14)	0.0005(11)	-0.0038(10)	-0.0126(11)
C29	0.0351(15)	0.0203(12)	0.0197(13)	-0.0032(10)	-0.0086(11)	-0.0076(11)
C30	0.0350(16)	0.0290(14)	0.0297(15)	0.0077(12)	-0.0136(13)	-0.0135(12)
O1	0.075(2)	0.0659(1	0.0503(1	-	-	-

8)	7)	0.0005(13)	0.0145(15)	0.0423(16)

Table 4 Bond Lengths for CCDC 1874186 .

Atom	Atom	Length/Å
S1	O7	1.427(2)
S1	O8	1.426(2)
S1	N11	1.642(2)
S1	C20	1.785(2)
S2	C16	1.724(3)
S2	C29	1.701(3)
F1	C28	1.321(3)
O2	C21	1.396(3)
O2	C28	1.338(3)
O3	C18	1.205(3)
O4	C18	1.317(3)
O4	C26	1.445(3)
O5	C19	1.328(3)
O5	C27	1.440(4)
O6	C19	1.201(3)
O9	C22	1.216(3)
F10	C28	1.314(3)
N11	C22	1.387(3)
C12	C15	1.386(3)
C12	C16	1.472(3)
C12	C23	1.391(3)
N13	C14	1.369(3)
N13	C16	1.321(3)
C14	C29	1.352(4)
C15	C17	1.391(3)
C17	C20	1.403(3)
C17	C25	1.519(3)
C18	C25	1.522(3)
C19	C25	1.521(4)
C20	C21	1.407(3)
C21	C23	1.382(4)
C22	C30	1.494(4)
F24	C28	1.306(3)

Table 5 Bond Angles for CCDC 1874186 .

Atom	Atom	Atom	Angle/°
O7	S1	N11	105.99(11)
O7	S1	C20	108.34(12)
O8	S1	O7	118.20(13)
O8	S1	N11	109.02(13)
O8	S1	C20	110.13(11)
N11	S1	C20	104.19(11)
C29	S2	C16	89.56(13)
C28	O2	C21	120.6(2)
C18	O4	C26	116.1(2)
C19	O5	C27	116.3(2)
C22	N11	S1	123.06(18)
C15	C12	C16	119.8(2)
C15	C12	C23	119.4(2)
C23	C12	C16	120.8(2)
C16	N13	C14	110.2(2)
C29	C14	N13	115.8(2)
C12	C15	C17	121.9(2)
C12	C16	S2	121.84(19)
N13	C16	S2	114.10(18)
N13	C16	C12	124.0(2)
C15	C17	C20	119.2(2)
C15	C17	C25	116.3(2)
C20	C17	C25	124.4(2)
O3	C18	O4	125.1(2)
O3	C18	C25	123.5(2)
O4	C18	C25	111.4(2)
O5	C19	C25	108.8(2)
O6	C19	O5	125.1(3)
O6	C19	C25	126.1(2)
C17	C20	S1	124.86(19)
C17	C20	C21	118.0(2)
C21	C20	S1	117.03(18)
O2	C21	C20	115.6(2)
C23	C21	O2	121.9(2)
C23	C21	C20	122.2(2)
O9	C22	N11	121.1(3)
O9	C22	C30	125.3(3)
N11	C22	C30	113.6(2)
C21	C23	C12	119.1(2)
C17	C25	C18	111.1(2)
C17	C25	C19	111.5(2)
C19	C25	C18	108.4(2)

F1	C28	O2	108.0(2)
F10	C28	F1	107.1(2)
F10	C28	O2	112.1(2)
F24	C28	F1	109.6(2)
F24	C28	O2	112.8(2)
F24	C28	F10	107.2(3)
C14	C29	S2	110.4(2)

Table 6 Hydrogen Atom Coordinates (Å) and Isotropic Displacement Parameters (Å^2) for CCDC 1874186 .

Atom	*x*	*y*	*z*	U(eq)
H11	-0.1621	0.6147	0.6363	0.023
H13	0.3205	0.8975	0.9848	0.024
H14	0.2978	1.0103	1.1626	0.027
H15	0.3266	0.7563	0.8572	0.021
H23	-0.1237	0.8009	1.0471	0.023
H25	0.3542	0.5696	0.6073	0.022
H26A	0.7573	0.3572	0.6841	0.04
H26B	0.6651	0.2968	0.7996	0.04
H26C	0.6974	0.4303	0.8123	0.04
H27A	0.5601	0.7803	0.3075	0.05
H27B	0.5197	0.8778	0.4178	0.05
H27C	0.3856	0.8708	0.3662	0.05
H29	0.063	1.0222	1.3232	0.03
H30A	-0.1847	0.9036	0.4846	0.045
H30B	-0.2912	0.842	0.5897	0.045
H30C	-0.2115	0.7709	0.4601	0.045
H1A	0.4046	0.9298	0.7686	0.089
H1B	0.3865	1.0465	0.8191	0.089

Datablock 20181015dongyi - ellipsoid plot

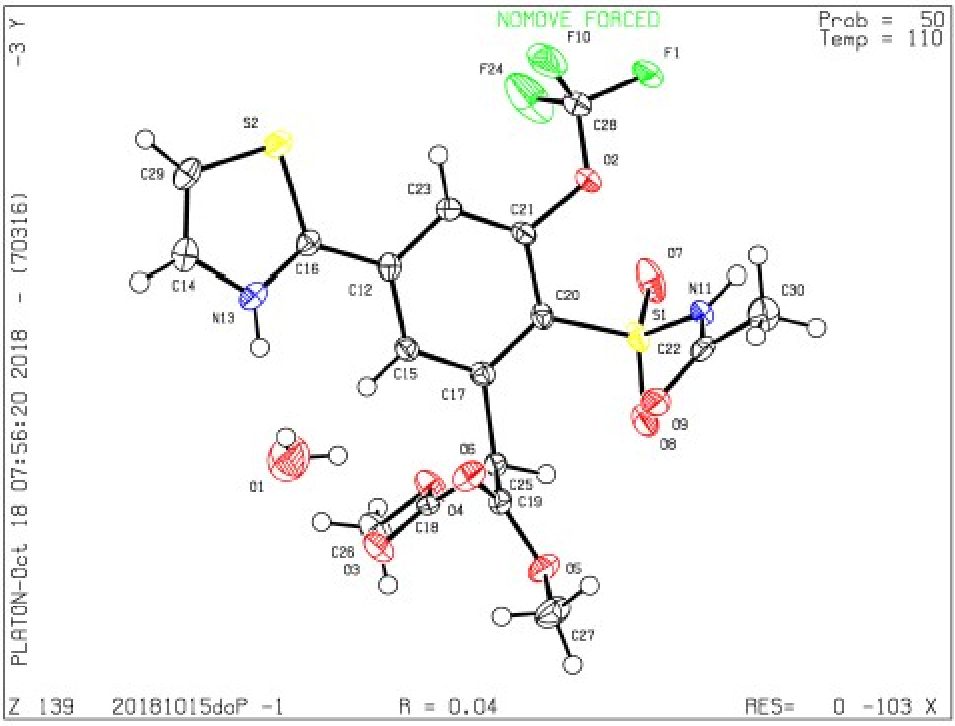

Supplement: Supplementary file 1 [file SC-010-C9SC03691A-s001.pdf]
